# Lifting the curse from high-dimensional data: automated projection pursuit clustering for a variety of biological data modalities

**DOI:** 10.1093/gigascience/giaf052

**Published:** 2025-05-29

**Authors:** Claire Simpson, Evgeniy Tabatsky, Zainab Rahil, Devon J Eddins, Sasha Tkachev, Florian Georgescauld, Derek Papalegis, Martin Culka, Tyler Levy, Ivan Gregoretti, Connor Meehan, Chiara Schiller, Kresimir Bestak, Denis Schapiro, Andrei Chernyshev, Guenther Walther, Eliver E B Ghosn, Darya Orlova

**Affiliations:** Cell Signaling Technology, Danvers, MA 01915, USA; Independent researcher, Komsomolsk-on-Amur 681021, Russia; Genentech, South San Francisco, CA 94080, USA; Division of Immunology and Rheumatology, Department of Medicine, Lowance Center for Human Immunology, Emory University School of Medicine, Atlanta, GA 30322, USA; Cell Signaling Technology, Danvers, MA 01915, USA; Cell Signaling Technology, Danvers, MA 01915, USA; Cell Signaling Technology, Danvers, MA 01915, USA; Department of Systems Biology, Columbia University, New York, NY 10032, USA; Cell Signaling Technology, Danvers, MA 01915, USA; Cell Signaling Technology, Danvers, MA 01915, USA; Independent researcher, Surrey, British Columbia V3T 3V4, Canada; Institute for Computational Biomedicine, Heidelberg University, Faculty of Medicine, Heidelberg University Hospital, Heidelberg 69120, Germany; Translational Spatial Profiling Center (TSPC), Heidelberg 69120, Germany; Institute for Computational Biomedicine, Heidelberg University, Faculty of Medicine, Heidelberg University Hospital, Heidelberg 69120, Germany; Institute for Computational Biomedicine, Heidelberg University, Faculty of Medicine, Heidelberg University Hospital, Heidelberg 69120, Germany; Translational Spatial Profiling Center (TSPC), Heidelberg 69120, Germany; Institute of Pathology, Heidelberg University Hospital, Heidelberg 69120, Germany; Voevodsky Institute of Chemical Kinetics and Combustion SB RAS, Novosibirsk 630090, Russia; Department of Statistics, Stanford University, Stanford, CA 94305, USA; Division of Immunology and Rheumatology, Department of Medicine, Lowance Center for Human Immunology, Emory University School of Medicine, Atlanta, GA 30322, USA; Cell Signaling Technology, Danvers, MA 01915, USA

**Keywords:** curse of dimensionality, clustering, high-dimensional data, projection pursuit, unsupervised machine learning

## Abstract

Unsupervised clustering is a powerful machine-learning technique widely used to analyze high-dimensional biological data. It plays a crucial role in uncovering patterns, structures, and inherent relationships within complex datasets without relying on predefined labels. In the context of biology, high-dimensional data may include transcriptomics, proteomics, and a variety of single-cell omics data. Most existing clustering algorithms operate directly in the high-dimensional space, and their performance may be negatively affected by the phenomenon known as the curse of dimensionality. Here, we show an alternative clustering approach that alleviates the curse by sequentially projecting high-dimensional data into a low-dimensional representation. We validated the effectiveness of our approach, named automated projection pursuit (APP), across various biological data modalities, including flow and mass cytometry data, scRNA-seq, multiplex imaging data, and T-cell receptor repertoire data. APP efficiently recapitulated experimentally validated cell-type definitions and revealed new biologically meaningful patterns.

## Introduction

The well-known phrase “the curse of dimensionality” was coined by Richard Bellman [1], who used it to describe the fact that the computational complexity of many numerical methods increases exponentially with the dimension. But there is also a statistical version of the curse of dimensionality, which is also called the “empty space phenomenon” and which refers to the fact that data become sparse in high dimensions. For example, if data are sampled from a 10-dimensional cube {*x* : |*x_i_*| ≤ 1 for all *i*}, then only about 1% of the data will fall into the cube {*x* : |*x_i_*| ≤ 0.63 for all *i*}. As a consequence, density estimators that use local averaging will not work well because most local neighborhoods will be empty. In order to obtain the same number of observations in the neighborhood given by a subcube as in the univariate case, the number of observations needs to increase exponentially with the dimension (see, e.g., [2]). This is the reason why, for example, multivariate density estimation is a notoriously difficult problem.

Modern biological data can be quite complex and high-dimensional, making it challenging to uncover meaningful insights from the data. Clustering is often used to discover interesting patterns in the data by partitioning it into clusters, where data points within the same cluster are more similar to each other than to those in other clusters. High-dimensional clustering and projection pursuit [3–5] aim to address the problem of discovering patterns in the data. However, they approach the problem from different angles.

High-dimensional clustering, such as HDBSCAN [6], KMeans [7], Phenograph [8], FlowSOM [9], and SPADE [10], aim to group similar data points together based on a similarity measure or fit to a posited generative model directly in the high-dimensional space. For example, PhenoGraph represents the dataset as a mathematical graph built using the *k*-nearest neighbors of each point, then runs a Louvain community detection algorithm to cluster the graph. While these approaches retain the full information of the original data, they are susceptible to the curse of dimensionality [2, 11, 12], which can lead to data sparsity and uninformative distance metrics. As a result, traditional clustering methods may struggle to accurately uncover biological patterns [13] (see also Supplementary Tables 1 and 2 in [14]). To address these limitations, dimensionality reduction techniques (e.g., PCA [15], t-SNE [16], UMAP [17]) are often used before clustering to enhance interpretability and mitigate high-dimensional challenges. However, these methods come with trade-offs because they may distort global structures or obscure biologically relevant variations.

An alternative approach is projection pursuit, which seeks lower-dimensional projections that reveal meaningful structures while preserving key data characteristics. This is motivated by the fact that in many situations the relevant information (such as cluster relationships) is contained in a lower-dimensional subspace [2], with the remaining dimensions being uninformative. Projection pursuit involves finding projections that maximize some criterion or interesting property. Once an interesting set of projections has been found, existing structures (clusters) can be extracted and analyzed separately. Projection pursuit can reveal hidden structures and relationships in the data that might be difficult to detect in the original high-dimensional space due to the curse of dimensionality. However, the choice of criterion will determine the types of patterns that the optimization will search for, and many choices exist. Additionally, identifying the right projection can be computationally intensive, since one needs to explore approximately *d^N^*/*N*! projections, where *d* is the dataset dimensionality, and *N* is the dimensionality of the low-dimensional projection. While this challenge is primarily computational rather than a fundamental scientific limitation, it can pose a significant practical obstacle.

The concept of exhaustively exploring low-dimensional projections of high-dimensional data has existed for a few decades. Historically, efforts have been made to systematically explore low-dimensional projections, known as the “grand tour” [18], or to optimize specific criteria for identifying informative projections, such as those that reveal structure in the data by deviating from normality or uniformity [3]. However, challenges in determining the optimal criterion and the computational complexities associated with processing numerous low-dimensional projections have hindered the widespread adoption of projection pursuit methods for data clustering tasks.

We developed automated projection pursuit (APP) clustering, combining projection pursuit principles [3–5] with clustering to uncover structures in high-dimensional data. Unlike traditional projection pursuit, APP automates the search for low-dimensional projections with minimal density between clusters, recursively refining clusters until no further splits are detected. This enhances reproducibility and mitigates the curse of dimensionality. APP was applied to diverse data modalities, including flow/mass cytometry, scRNA-seq, multiplex imaging, and TCR repertoire data, accurately recapitulating known cell types and providing new biological insights. Notably, it enabled the assessment of a potential binding motif in the CDR3b region of TCRs and a charged amino acid pattern stabilizing CDR3a–CDR3b interactions. To evaluate APP’s performance against other high-dimensional clustering methods, we applied it to biological data with known ground truth and implemented a label transfer pipeline using supervised UMAP [19]. This approach facilitated quantitative comparisons while preserving data topology. As an example, our analysis uncovered a novel myeloid cell population enriched in hospitalized COVID-19 patients.

## Materials and methods

### Data overview

#### Flow cytometry data

##### Dataset with a functionally validated ground truth

Mice lacking the RAG1 gene (i.e., RAG-KO) are deficient in immune cells known as B and T lymphocytes, while still developing all other major immune lineages, including myeloid cells and NK cells. Since the wild-type (WT) mice, expressing the RAG1 gene, can develop both B and T lymphocytes, we intentionally mixed WT cells from a GFP+ mouse, which expresses green fluorescent protein on lymphocytes, with cells from the RAG-KO mouse, which does not contain lymphocytes (i.e., WT-GFP mixed with RAG-KO). We used this experimental approach to define a biological and technical ground truth. For example, any B and T cells identified by our new APP pipeline should express the green (GFP) protein because they can only come from the WT-GFP mice and not from the RAG-KO mice. If the pipeline detects any B and T lymphocytes lacking the green/GFP protein, these events would be considered as “misclassification” by the APP pipeline.

##### COVID dataset

Whole blood from consenting COVID-19 patients and healthy donors were collected as part of our previous study (see [20] for Emory Institutional Review Board (IRB) protocol numbers) by standard venipuncture, then samples were processed as previously described [20]. Peripheral blood mononuclear cells (PBMCs) were isolated from whole blood after serum collection using an EasySep Direct Human PBMC Isolation Kit (StemCell Technologies, Vancouver, Canada) following the manufacturer’s instructions. We then performed either a custom monocyte-enrichment procedure (via negative selection) utilizing Mojosort anti-PE Nanobeads (BioLegend, San Diego, CA, USA) and PBMCs stained with CD3ε::PE (clone: UCHT1), CD19::PE (SJ25C1), CD56::PE (5.1H11), and CD57::PE (HNK-1; all from BioLegend, San Diego, CA, USA) for COVID-19 samples or an EasySep Human Monocyte Enrichment Kit without a CD16 Depletion kit (StemCell Technologies, Vancouver, Canada) for health donor samples.

An aliquot of monocyte-enriched PBMCs (<10^7^ total) was resuspended in fluorescence-activated cell sorter (FACS) buffer in 5 ml FACS tubes and preincubated with Human TruStain FcX (BioLegend, San Diego, CA, USA). The 28-color extracellular staining master mix included: CD86::BB515 (clone: FUN-1; titration 1:10), CD45-RA::BB630-P2 (HI100; 1:160), CD19::BB660-P2 (HIB19; 1:50), CD45::BB700 (HI30; 1:640), CD4::BB755-P (RPA-T4; 1:100), HLA-DR::BB790-P (G46-6; 1:50), CD1c::BV480 (746677; 1:20), HLA-ABC::BV650 (G46-2.6; 1:320), CD11b::BV750 (ICRF44; 1:20), CD56::BUV563 (NCAM16.2; 1:160), CD123::BUV661 (9F5; 1:40), CD14::BUV737 (M5E2; 1:50), CD8α::BUV805 (SK1; 1:80) from BD Biosciences (Milpitas, CA, USA); CD163::BV421 (GHI/61; 1:50), CD16::BV570 (3GB; 1:100), CD169::BV605 (7-239; 1:100), CD141::BV711 (1A4; 1:100), CD197::BV785 (G043H7; 1:50), XCR1::PE (S15046E; 1:50), CD206::PE-Dazzle594 (15-2; 1:200), CD10::PE-Cy5.5 (HI10a; 1:20), CD3ε::PE-Cy5 (UCHT1; 1:100), CD172a/b::PE-Cy7 (SE5A5; 1:200), CD66b::APC (QA17A51; 1:200), CD11c::AF700 (Bu15; 1:100), and CD32::APC-Fire750 (FUN-2; 1:50) from BioLegend (San Diego, CA, USA); and the amine-reactive viability stain GhostDye UV450 (1:100) from Tonbo Biosciences (San Diego, CA, USA). Fluorophores marked with -P(2) denote prototype reagents that are custom conjugations from BD Biosciences (Milpitas, CA, USA); purified CD10 was conjugated in-house using a Lightning-Link PE-Cy5.5 Antibody Labeling Kit (catalog no. 761-0010) from Novus Biologicals (Centennial, CO, USA) (Abbreviations: AF: AlexaFluor, APC: Allophycocyanin, BB: Brilliant Blue, BUV: Brilliant Ultraviolet, BV: Brilliant Violet, FITC: Fluorescein isothiocyanate, PE: Phycoerythrin). Titrations of all reagents were determined empirically for each lot independently. The staining master mix was prepared 2× in BD Horizon Brilliant Stain Buffer (BD Biosciences, Milpitas, CA, USA) and added 1:1 to cells. After staining, cells were fixed with 4% paraformaldehyde, then washed with FACS buffer, and resuspended in 200–1,000 µl FACS buffer for acquisition using BD FACSDiva Software on and Emory Pediatric/Winship Flow Cytometry Core BD FACSymphony A5.

All 6 COVID-19 samples analyzed in this study were collected from patients with severe disease in the intensive care unit (ICU). To minimize biological variability, we intentionally avoided mixing samples from COVID-19 patients with different disease severities.

#### Mass cytometry (CyTOF) data

Whole blood was collected from consenting healthy human donors (*N* = 10), and PBMCs were isolated and stained with a metal-conjugated 38-parameter mAb panel (see Table S1 in [21]), enabling the comparison of 28 immune cell subset frequencies [21]. Data were acquired using a Helios CyTOF® system (Fluidigm, South San Francisco, CA, USA).

#### RNAseq data

##### PBMC dataset

Publicly available scRNA-seq counts data from 2,700 single PBMCs were accessed from 10x Genomics, Pleasanton, CA, USA (see Data Availability).

##### Wild-type and 5XFAD mouse model dataset

Single-nucleus RNA-seq counts data for 3 wild-type and 3 5XFAD 7-month-old mouse brains was downloaded from the Gene Expression Omnibus (GEO) database (see Data Availability) [22]. Droplet-based 5′ end massively parallel single-cell RNA sequencing had been performed on the samples, and data processing was done using the Cell Ranger Single-Cell Software Suite from 10x Genomics, Pleasanton, CA, USA by the originators of the data.

#### Multiplex imaging data

CD11c (D3V1E), SIRPɑ (D6I3M), CD163 (D6U1J), CD206/MRC1 (E2L9N), CD68 (D4B9C), CD45 (D9M8I), HLA-DRA (E9R2Q), and Pan-Keratin (C11) antibodies were conjugated to oligonucleotides (oligos) and then validated in a SignalStar Multiplex IHC assay to assess the myeloid compartment of the tumor microenvironment. Paraffin-embedded human squamous cell carcinoma tissue was tested using an 8-plex panel Pan-Keratin (C11) and CO-0003-488 SignalStar Oligo-Antibody Pair #63566 (0.25 μg/ml), a CD68 (D4B9C) and CO-0007-594 SignalStarM Oligo-Antibody Pair #77318 (0.5 μg/ml), a CD206/MRC1 (E2L9N) and CO-0035-488 SignalStar Oligo-Antibody Pair #99626 (0.25 μg/ml), a CD163 (D6U1J) and CO-0022-750 SignalStar Oligo-Antibody Pair #71043 (0.7 μg/ml), an SIRPɑ/SHPS1 (D6I3M) and CO-0034-647 SignalStar Oligo-Antibody Pair #80150 (0.625 μg/ml), a CD45 (Intracellular Domain) (D9M8I) and CO-0013-647 SignalStar Oligo-Antibody Pair #32740 (0.1 μg/ml), a CD11c (D3V1E) and CO-0017-594 SignalStar Oligo- Antibody Pair #85384 (2.0 μg/ml), and an HLA-DRA (E9R2Q) and CO-0023- 750 SignalStar Oligo-Antibody Pair #58446 (0.05 μg/ml) using SignalStarTM mIHC technology.

All 8 primary antibodies are applied at once in one primary incubation step. A network of complementary oligonucleotides with fluorescent channels 488, 594, 647, and 750 nm amplifies the signal of up to 4 oligo-conjugated antibodies in the first round of imaging, followed by removal and amplification of 4 additional antibodies in the second round of imaging. Images were acquired on a PhenoImager HT (Akoya Biosciences, Menlo Park, CA, USA). The antibodies were quantitatively validated by SignalStar assay to ensure maximum fluorescent signal with minimal background, and compared against the chromogenic gold standard.

#### TCR repertoire data

The TCR repertoire data utilized in this study were obtained from the McPAS-TCR database [23]. McPAS-TCR is a manually curated resource containing human and mouse TCR sequences associated with various pathologies and their cognate antigens. We downloaded the September 10, 2022 version of McPAS-TCR (latest available), providing over 13,000 TCR CDR3-beta chain and epitope pairs.

The TCR molecule is a heterodimer that primarily interacts with pMHC through its CDR3a and CDR3b chains. While using paired CDR3a and CDR3b data points is ideal, the high cost of sequencing has led most studies to focus solely on the CDR3b chain. Despite this limitation, the field continues to rely on the CDR3b chain as the primary determinant of TCR specificity because it is the most highly variable region that contacts pMHC. Accordingly, we have maximized the use of the available dataset by leveraging both CDR3b chain data points and those with paired CDR3a sequences whenever possible.

### Data analysis

#### Flow cytometry data

Manual, user-guided analyses were performed using AutoGate [14, 24] and FlowJo v. 10.8 (BD Biosciences, Milpitas, CA, USA).

To assess the performance of the label transfer pipeline, only healthy control samples were used for training (1 or 3 samples randomly selected from a total of 6) because the manual gating strategy was established based on healthy controls. The remaining manually gated healthy control samples, along with all COVID samples, were used as the test set (1 sample at a time). This procedure was repeated 3 times, and representative results are shown.

#### Mass cytometry (CyTOF) data

The comprehensive conventional manual gating strategy for 38-parameter human immunophenotyping is described in [21].

#### RNA-seq data

Both datasets were processed using Seurat v. 3 [25]. Counts were log-normalized and scaled, and UMAP reduction and PCA (principal component analysis) were performed. Seurat’s default graph-based clustering algorithm was used to identify cell-type clusters [26]. Cell types were annotated by comparing known biomarkers with the markers calculated for each cluster. The PBMC dataset was reprocessed using Seurat v. 5 to assess any updates to Seurat’s clustering algorithm, and the clustering results were unchanged between versions.

Principal components (10 for the PBMC dataset and 20 for the brain dataset [22]) were extracted from the Seurat objects to run the APP clustering procedure (using a minimum cluster size of 150 for both datasets and a minimum cluster size of 10 to further cluster the T cells in the PBMC dataset at a more granular level), which produced new cluster identifications for each cell. New cell annotations were identified by comparing known biomarkers with cluster markers calculated using the new identifications. Differently matched and non-matched cells were identified and tabulated. Dimensionality reduction plots and heat maps were produced using Seurat, and other visualizations were produced using ggplot.

#### Multiplex imaging data

To interpret SignalStar data collected in two imaging rounds (rounds 1 and 2) with the PhenoImager HT (Vectra Polaris), several data preprocessing and processing steps were performed. More specifically, whole-slide imaging data collected with PhenoImager HT underwent image stamping and whole-section selection was performed in Phenochart (Akoya Biosciences, Menlo Park, CA, USA), with the further spectral unmixing and autofluorescence removal done in Inform (Akoya Biosciences, Menlo Park, CA, USA) to distinguish true signals from background noise and ensure accurate quantification of each fluorophore signal. We then offer 2 analysis options for downstream data processing: using QuPath or MCMICRO.

#### QuPath analysis

TIFF components were then exported from Inform (Akoya Biosciences, Menlo Park, CA, USA) into the QuPath [27] software where the TIFF component stitching, image alignment co-registration, and image fusion were sequentially performed. These steps allow simultaneous visualization of multiple markers, signals from which were recorded across different cycles or time points.

Nuclear and membrane segmentation was then done using the Cellpose QuPath extension [28] on the preprocessed images. Specifically, the “nuclei’ base model of the Cellpose algorithm was used, with the DAPI nuclear signal from round 1 serving as its input. The expected diameter of the detected nuclei was set to zero to allow for automatic computation by Cellpose. To approximate cell boundaries, a nucleus expansion algorithm implemented in Cellpose was employed, with the cellExpansion parameter set to 5 μm. Cell expansion was constrained to 1.5 times the size of the nucleus, controlled by the cellConstrainScale parameter. Additionally, tile size was set to 2,048 pixels and the setOverlap parameter that accounts for overlaps between the tiles was set to 100 pixels.

Following segmentation, 20 features per marker were extracted for each single cell and used for the subsequent cellular analysis including cell phenotyping. Specifically, measurements of marker mean, median, maximum, minimum, and standard deviation were calculated for the nucleus, cytoplasm, membrane, and the entire cell.

#### MCMICRO analysis

The image-processing pipeline MCMICRO [29, 30] allows scalable and modular analysis of highly multiplexed images. The component data .tif files exported with the Inform software after unmixing were preprocessed to be compatible with the pipeline. In detail, the original per channel and tile .tif files were stacked into one ome-tiff file per cycle (rounds 1 and 2). The signal intensities were normalized across each channel within a cycle by the respective maximum value and converted from float32 to uint16. Further, the metadata were restructured to meet the ome-xml metadata standard. We combined the described preprocessing steps into a phenoimager2mc staging module (see Availability of source code and requirements) that users can apply to analyze their multicyclic PhenoImager data with MCMICRO.

Within MCMICRO, additionally, registration and stitching were performed with ASHLAR (1.18.0) [31] based on the DAPI channels from both cycles (channels 5 and 11 in the stacked image). Cell segmentation was performed with DeepCell Mesmer based on the max projection of DAPI channels (compartment “nuclear,” 0.4.0) [32]. Mean intensities and morphological properties were returned per cell using the MCQuant module (1.6.0) [30]. The pipeline was run using Nextflow [33].

While in some instances the MCMICRO analysis pipeline may demonstrate comparable performance to the QuPath pipeline (see Supporting_Figure_MCMICRO_vs_QuPath in the phenoimager2mc staging module (see Availability of source code and requirements)), MCMICRO offers the advantage of enabling batch processing of multiplex imaging data such as SignalStar.

#### TCR repertoire data

The TCR CDR3b and CDR3a sequences, and associated peptide epitope sequence data, underwent conversion into embeddings using recent techniques in large language model (LLM) technology. Evolutionary Scale Modeling (ESM) [34] has recently harnessed LLMs to create a collection of protein language models. Specifically, we utilized the esm2_t33_650M_UR50D model from ESM to initially generate embeddings for TCR CDR3b and CDR3a, and peptide epitope sequences. These embeddings, characterized by a high dimensionality (1,280 dimensions), were independently created for TCR CDR3b and CDR3a, and peptide epitope sequences. Subsequently, we concatenated the embeddings (1280D for TCR CDR3b and 1280D for peptide; 1280D for TCR CDR3a, 1280D for CDR3b, and 1280D for peptide) to capture the combined information pertaining to TCR-antigen interactions.

The concatenation of these embeddings results in a feature vector (2560D for CDR3b and peptide; 3840D for CDR3a, CDR3b, and peptide) that encapsulates the unique characteristics of both the TCR and antigenic sequences. This combined representation aims to capture the intricacies of TCR–antigen interactions. PCA was then applied to these combined embeddings, and the first 30 principal components were used in APP clustering (minimum cluster size = 100). This approach enabled thorough exploration and analysis of the dataset, unveiling intricate patterns and relationships within the combined TCR–antigen sequence space.

The sequence similarity within each class (CDR3a, CDR3b, and peptide epitope) was calculated by aligning each pair of sequences and computing the Blosum62 score for the alignment (utilizing the Bio.pairwise2 module in the Biopython package [35]). The Blosum62 score offers a quantitative measure of the similarity or dissimilarity between amino acids at specific positions in protein sequences, relying on observed frequencies of substitutions in related proteins. It is commonly utilized in sequence alignment algorithms to assess the evolutionary relationships between proteins and identify regions of conservation or divergence. Within each cluster, an average sequence similarity was calculated by averaging the scores for each unique pair of sequences found in that cluster. Between each pair of clusters, an average sequence similarity score was calculated by averaging the scores for each unique pair of sequences between the two clusters. Unique pairs were used to avoid biasing within-cluster average scores for clusters containing many repeated sequences.

Cluster sequence logos for epitope peptides were generated by selecting all sequences of uniform length. This length was defined as the rounded average sequence length among all sequences in a cluster within a given class of sequences. This approach ensured ∼70–80% coverage (coverage varies among the clusters) for epitope sequences. Cluster sequence logos for CDR3 sequences were calculated by aligning CDR3a and CDR3b sequences using ANARCI, using temporary pseudo sequences to fill in the gaps and simulate full TCR sequences [36]. The distribution of amino acid residues at each position was then calculated for these sequences. The Python package LogoMaker was used to create probability matrices for the sequence logos, which were subsequently utilized for the analysis of amino acid R group properties.

#### TCR-pMHC crystal structure analysis

Crystal structures under PDB accession codes 3GSN, 3PQY, 1OGA, 3O4L, and 5EUO were used to analyze interfaces between antigen and TCRa, antigen and TCRb, and TCRa and TCRb. For each structure the analysis was performed using the PISA service [37, 38]. A manual verification was performed for each structure using the PyMOL Molecular Graphics System, v. 1.2r3pre (Schrödinger, LLC, Portland, OR, USA). Figures were generated with PyMOL.

### Automated projection pursuit clustering based on the best separation score

**Figure 1:**
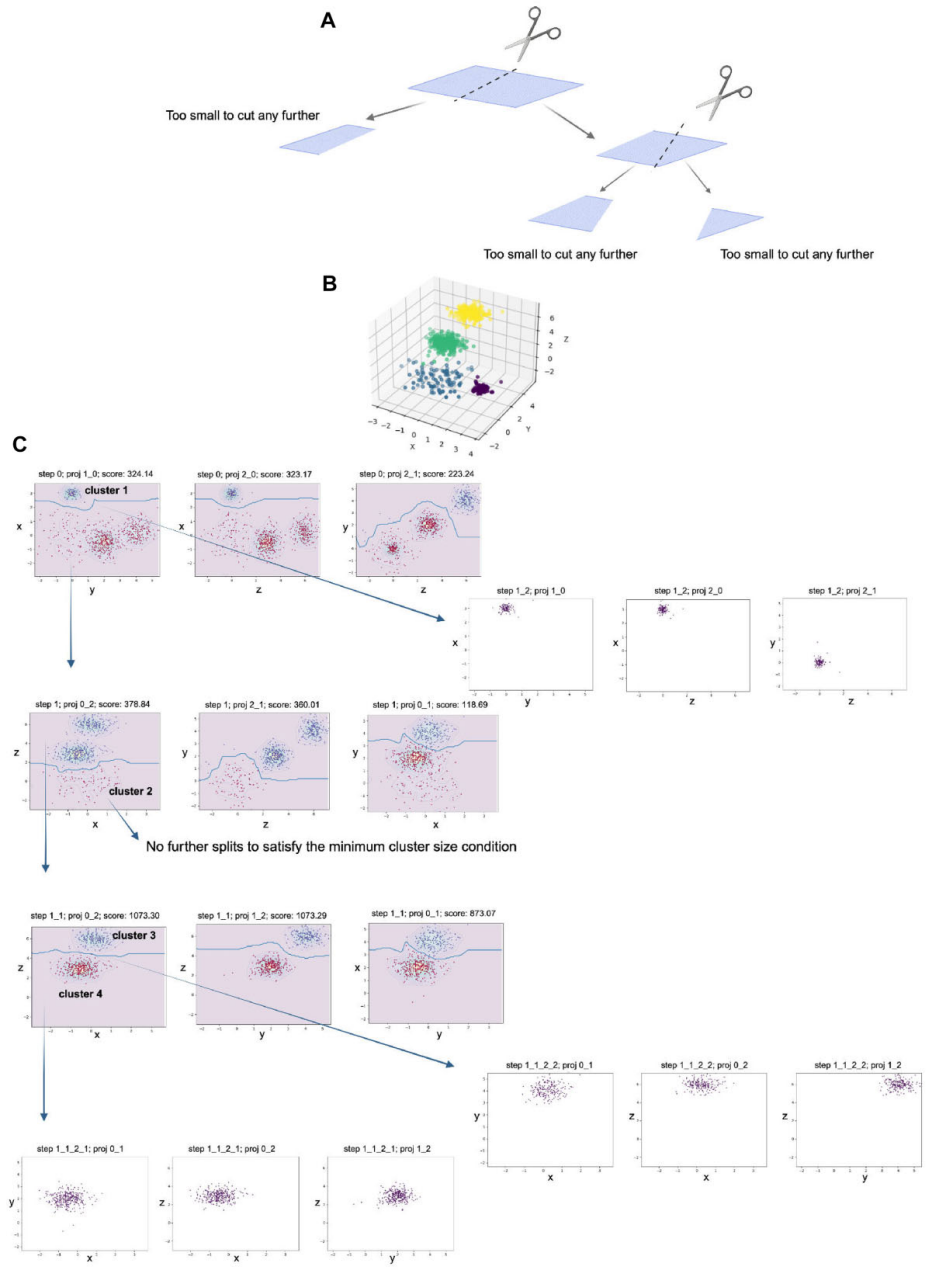
Automated projection pursuit clustering workflow. (A) Metaphorical representation of projection pursuit clustering, where the paper sheet represents multidimensional data projected into a pair of dimensions with the best separation (all other projections are not shown). Scissors represent the process of splitting the data into segments along a decision boundary (dotted line). This step reflects the idea of sequentially finding the most informative projections to separate the data. (B) To illustrate the concept behind the APP algorithm in a simplified manner, we used a 3D synthetic dataset. (C) The APP algorithm systematically explores orthogonal 2D projections, selects the one with the smallest density distribution along the decision boundary (represented by a blue line on the 2D projections), and recursively splits the data until the defined stop criteria are met. This approach helps uncover meaningful patterns and structures in the data. For simplicity, projections with interchanged axes are not displayed (e.g., only the *xy* projection is shown instead of both *xy* and *yx*).

The overall data clustering workflow (Fig. 1) is constructed to unambiguously assign a cluster identification number (ID) to each data point in the dataset by recursively performing the following three steps: (1) presenting the multidimensional data in all its two-dimensional (2D) orthogonal projections; (2) for each 2D projection, finding the decision boundary (i.e., the boundary separating cluster assignments) according to the local minimum density of the data points; (3) choosing the 2D projection that has the decision boundary with the highest Calinski–Harabasz [39] score, and splitting the data along this decision boundary. The Calinski–Harabasz index, which is often used to evaluate the goodness of split, is calculated as a ratio of the sum of intercluster dispersion and the sum of intracluster dispersion for all clusters (where the dispersion is the sum of squared distances). These steps 1 and 2 are repeated recursively and exhaustively until there are no further splits, as defined by the user-input minimum cluster size parameter. This parameter should be set based on the smallest population the user expects to detect in the dataset.

The generalized structure for such an algorithm is as follows.

In each recursive step:

If the number of points at the input to the recursion step is less than 2× min_cluster_size (user-defined parameter), then this piece of data is considered as the final cluster, cluster ID is assigned, and no further splits are performed. The algorithm exits the recursion step.

Otherwise, for each 2D (*x,y*) projection mapped onto a unit square (side length of 1), we build the Gaussian-smoothed histogram *H*(*x,y*) of the data points—i.e., the initial distribution density *H*. To determine the optimal number of bins of the 2D histogram, we use Mann’s formula [40], taking into account the number of data points *n* in the current 2D projection. The challenge of determining the optimal number of histogram bins, contingent on the number of data points, remains an ongoing issue without unanimous consensus in the literature. The optimal choice must strike a balance between having too few bins (resulting in poor resolution) and too many bins (leading to increased noise). The Gaussian smoothing width *σ* is taken as a free parameter of the algorithm. Then, the number of bins *N* for each of the two coordinates is the square root of the total number of 2D bins (as determined by Mann’s formula) multiplied by the width of the Gaussian smoothing:


(1) \begin{eqnarray*}
N = 4\sigma {{\left[ {3{{{\left( {n - 1} \right)}}^2}/4} \right]}^{0.1}}
\end{eqnarray*}

Thus, the use of Gaussian smoothing not only reduces the statistical noise of the data, but also increases the number of histogram bins. Calculating the optimal number of histogram bins depending on the number of data points considered at each recursion step made it possible to significantly speed up the calculations because it reduced the number of algorithm operations due to the reduction in the size of clustered projections during program operation.

To initiate the search for a decision boundary function $y( x )$ (Supplementary Fig. 1) we build ${{H}_1}$, a function that is a sum of the initial distribution density *H* and a parabolic function ${{H}_g}$—the “${{y}_0}$-gravity potential.” The function ${{H}_g}( y )$ is added to partially straighten the decision boundary function $y( x )$ along the *x*-axis. Addition of the ${{H}_g}( y )$ function to the data histogram results in a constraint for the following decision boundary condition: $y( {{{x}_{\min}},\ q} ) = {{y}_0}( q )$, and in a constraint that $y( {x,\ q} )$ will be as close as possible to the ${{y}_0}( q )$ for every $x\ \epsilon\ [ {{{x}_{\mathrm{min}}},\ {{x}_{\mathrm{max}}}} ]$, where *q* is the variable being optimized; writing $y( x )$ instead of $y( {x,\ q} )$ implies that *q* has taken its optimal value.


(2) \begin{eqnarray*}
{{H}_g}\left( {y,\ q} \right) = {{\left( {y - {{y}_o}} \right)}^2};\ {{y}_o}\ = \ q{{y}_{\mathrm{max}}} + \left( {1 - q} \right){{y}_{\mathrm{min}}}
\end{eqnarray*}

Where $q\ \epsilon\ [ {0,\ 1} ]$ is a numeric parameter.


(3) \begin{eqnarray*}
{{H}_1}\left( {x,\ y,\ q} \right) = H\left( {x,y} \right) + k{{H}_g}\left( {y,\ q} \right)
\end{eqnarray*}

Here, ${{k}_{}}$ is a positive constant, the optimal value of which is calculated by the following expression:


(4) \begin{eqnarray*}
k = \beta \cdot\left( {{\mathrm{max}}\left( H \right) - {\mathrm{min}}\left( H \right)} \right)/{\mathrm{max}}\left( {{{H}_g}} \right)
\end{eqnarray*}

The coefficient ${{k}_{}}$ plays a crucial role in achieving a balance between the parabola and the data, particularly in determining the trajectory of the decision boundary. The greater the coefficient *k*, the greater the straightening effect. Нere, the multiplier $\beta $ is a free parameter that varies the degree of influence of the “${{y}_0}$-gravity potential” on the clustering process. Our empirical assessment shows that a value of $\beta $=0.1 (i.e., 10% “gravity”) gives fairly good clustering results in many cases.

To “draw” a decision curve on a 2D plane one needs to know its initial [$y({{x}_{\mathrm{min}}})$] as ${{y}_o}$ and final [$y({{x}_{\mathrm{max}}})$] as ${{y}_1}$ boundary conditions. The explicit, but more computationally intense, solution would be to search for a decision boundary for each possible ${{y}_o}$ and ${{y}_1}$ in a given 2D projection. To optimize this process, we instead made the parabolic function ${{H}_g}$ depend on a parameter *q* that is used to introduce the next incremental step along the axes (i.e., $\ \delta q$ = 0.1).

To find the decision boundary with the smallest data density between the resulting clusters, for every parameter value $q\epsilon[ {0,1} ]$ (Supplementary Fig. 1), we search for extremals ${{f}_q}( x )$ (an analogue of trajectory from analytical mechanics) and the curvilinear integral *S*(*q*) (an analogue of action from analytical mechanics) of the probability density along the decision boundary:


(5) \begin{eqnarray*}
{{f}_q}\left( x \right) = \textit{argmi}{{n}_{y\left( {x,\ q} \right)}}\mathop \int \limits_{{{x}_{\mathrm{min}}}}^{{{x}_{\mathrm{max}}}} {{H}_1}\left( {x,y\left( {x,\ q} \right),q} \right)dx
\end{eqnarray*}

(6) \begin{eqnarray*}
S\left( q \right) = \mathop \int \limits_{{{x}_{\mathrm{min}}}}^{{{x}_{\mathrm{max}}}} H\left( {x,{{f}_q}\left( x \right)} \right)dx
\end{eqnarray*}

Further, we need to find the values ${{q}_0}$ that would satisfy the following condition: $S( {{{q}_0} - \delta q} ) > S( {{{q}_0}} ) < S( {{{q}_0} + \delta q} ).$ For such values, ${{f}_{{{q}_0}}}( x )$ will be a true extremal or decision boundary. Once a decision boundary is found, data are then split along that decision boundary. If both parts of the data obtained after splitting contain more than min_cluster_size cells, then this decision boundary is added to the list of decision boundaries for a given projection. If more than one decision boundary is found on a given projection, these decision boundaries are then ranked according to their Calinski–Harabasz score, and the decision boundary with the maximum Calinski–Harabasz score is chosen to represent the given projection. In our algorithm, the same projections are analyzed twice to determine a 2D cluster boundary in (*x,y*)-space: first to identify the *x*-projection of the boundary and then to determine its *y*-projection.

Then, among all possible 2D projections at a given recursion step, the algorithm chooses the one that contains the decision boundary with the maximum Calinski–Harabasz score. The data are then split along this decision boundary, and the new recursive step is initiated on each of 2 data pieces independently. If, however, there are no candidate decision boundaries (because every boundary leads to a split for which one part of the data contains less than min_cluster_size cells), the algorithm assigns the final cluster ID to this piece of data and recursion stops.

APP has only one required user input parameter: the minimum cluster size, which should be set based on the smallest population the user expects to detect in the dataset. Reducing this value introduces smaller clusters, as expected, while other APP clustering outcomes remain unchanged. All other parameters, such as Gaussian smoothing width, are optional. We have set default values optimized for the Calinski–Harabasz index based on the datasets presented in the manuscript. These parameters are user-adjustable to allow optimization for other data types, guided by the Calinski–Harabasz index.

### Automated label transfer across samples

At a high level, the label transfer pipeline enables the use of a labeled (or partially labeled) set of points to learn a metric on the data. This learned metric is then utilized as a measure of distance between new, unlabeled points. The immediate practical applications of such a pipeline, demonstrated here, include the automation of an expert-defined manual gating strategy, and the assessment of clustering algorithm performance against the ground truth cluster labels. Our concise 4-step pipeline facilitates the visualization and quantification of the misclassification rate between the clustering algorithm and the ground truth labels (see Supplementary Fig. 2).

The first step of the pipeline involves creating a supervised UMAP embedding [19] using labeled or partially labeled training sample(s) with both marker expression data and ground truth cluster labels. UMAP is applied to this training data with the goal of learning a distance metric that best separates the classes while preserving their relationships in the marker space. Using equal weights (target_weight=0.5 as described in [41]) for marker expression and ground truth cluster labels ensures that both data-driven and prior knowledge are considered in the embedding.

This step itself provides an opportunity to assess the quality of the ground truth cluster labels by observing an agreement (or disagreement) between the data topology and clustering decisions. By observing the agreement or disagreement between the data’s topological structure in the UMAP space and the provided ground truth cluster labels, one can gain insight into whether the ground truth labels accurately reflect the underlying structure of the data (Supplementary Fig. 3). Strong agreement between the UMAP topology and the ground truth labels suggests that the ground truth labels are representative of the data’s natural clustering patterns. Conversely, disagreements may indicate issues with the ground truth labels.

In the second step (Supplementary Fig. 2), the set of labeled points is utilized to learn a metric on the data. This learned metric subsequently serves as a distance measure between new unlabeled points, facilitating the projection of an unlabeled test set into the UMAP embedding space constructed using the training set. This ensures that the test set occupies the same reduced-dimensional space as the training set.

In the third step, the test set is subjected to clustering using the Support Vector Clustering (SVC) [42] algorithm directly applied in the supervised UMAP embedding space. Given that clustering in this context is confined to 2 dimensions (UMAP_x and UMAP_y), we opted for an algorithm that refrains from assigning any of the events to noise and, at the same time, offers computational superiority over APP. Subsequently, the QFMatch algorithm [43] is utilized to align the cluster labels between the test set (with cluster IDs defined by SVC or assigned by the clustering algorithm under assessment—see Supplementary Fig. 4) and the training set (with ground truth cluster IDs). The alignment of labels is crucial because it accommodates the following scenarios: (1) transferring cluster labels from the test set to the training set; (2) directly assessing the agreement of clustering decisions made by multiple clustering algorithms (see Supplementary Fig. 4).

In the final step, we compute the number of misclassified events per cluster ID. This quantifies how effectively the clustering algorithm has assigned data points to clusters in comparison to the ground truth.

Beyond clustering algorithm evaluation, this pipeline holds broader applications in supervised learning tasks. As demonstrated here, it can be used to transfer labels from one sample to another or from a partially labeled dataset to the remaining data in a given set. This feature proves particularly valuable in scenarios where labeled data are limited.

## Results

To encompass the range of real-world scenarios for clustering algorithm usage, both with and without ground truth or domain knowledge, we first demonstrate the method’s performance using a dataset with a functionally validated ground truth. We then apply the method to a dataset where expert-defined manual gating serves as the “ground truth.” Next, we test the algorithm on single-cell mRNA expression data and imaging data, which lack predefined ground truth. However, cluster evaluation is enabled using domain knowledge from pathology and immunology, leveraging expression patterns and spatial distributions. Finally, we apply APP clustering in a fully exploratory mode, where no ground truth or pre-existing domain knowledge is available, to assess how TCR receptor embeddings cluster with respect to their cognate antigens.

### Performance on data with functionally validated ground truth labels

**Figure 2:**
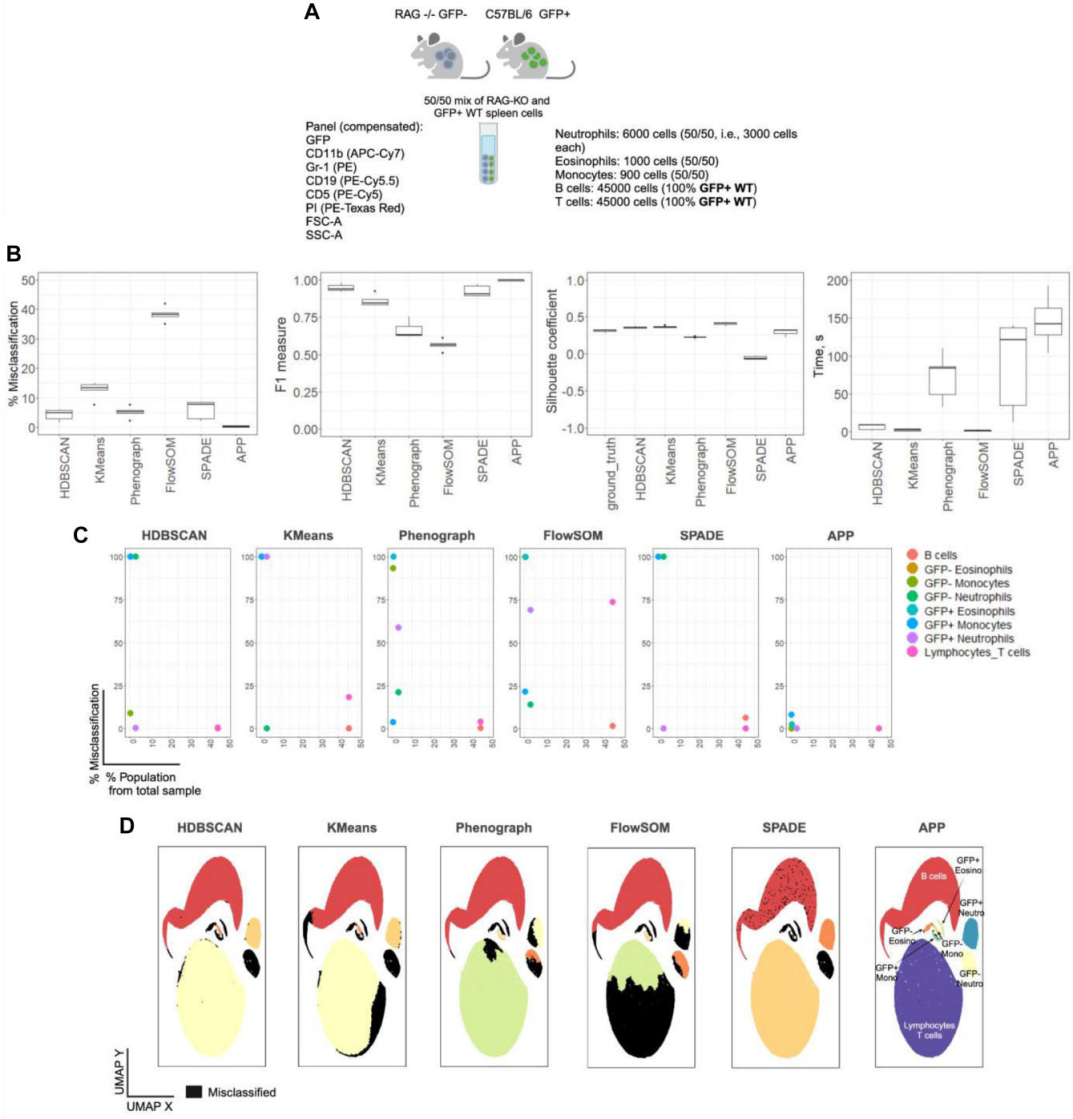
APP outperforms widely used clustering methods when applied to a representative biological dataset that has functionally validated ground truth labels. (A) A representative ground truth sample, selected from several similar types of samples, was generated by mixing cells in equal proportions from GFP+ wild-type spleen and RAG-KO spleen. It is important to note that RAG-KO mice lack B and T cells. (B) The performance of the APP algorithm in cell population classification, evaluated through total misclassification, F1-measure, and Silhouette coefficient, is assessed and compared to state-of-the-art clustering algorithms. The evaluation encompasses the blending of wild-type spleen cells and RAG-KO spleen cells in 5 different proportions (see the RAG-KO immune dataset for more details). Clustering time was evaluated on a laptop equipped with an 11th Gen Intel Core i7-1165G7 @ 2.80 GHz processor and 32.0 GB of RAM, using datasets ranging from ∼27,000 to 98,000 cells with a dimensionality of 7. We used the default input parameters recommended for each clustering algorithm based on the available tutorials. (C) Per-cell-type population misclassification assessment, with cell populations ordered from least to most abundant (left to right). (D) The misclassification for the 50/50 mix is visually represented in black using the automated label transfer pipeline, as detailed in Supplementary Fig. 4. Here, the performance of each of the 6 clustering algorithms is assessed against the ground truth labels (functionally distinct cell types) used to construct the supervised UMAP embedding.

To objectively assess APP’s performance against widely used clustering algorithms when applied to realistic, biologically relevant data, we used ground truth data where each cell population was quantified and functionally validated. To generate such biologically relevant data with a known ground truth, we combined spleen cells from GFP+ wild-type and RAG-KO mice in 5 different proportions (Fig. 2A). RAG-KO mice are deficient in generating lymphoid lineages, while GFP+ mice represent a healthy immune system with constitutive GFP expression in all immune cell lineages. This allows for easy and accurate detection of immune cell populations by flow cytometry, simplifying the identification and quantitation of cell lineages. The latter ensures that the clusters obtained by clustering algorithms can be compared against a known biological truth [44].

To assess the performance of the APP algorithm in comparison to state-of-the-art clustering algorithms, we selected two generally widely used high-dimensional clustering algorithms, irrespective of data origin: HDBSCAN [45] and KMeans [46]. Additionally, we included Phenograph [47], FlowSOM [9], and SPADE [10] as methods that are widely used in the flow cytometry field. Our selection of algorithms reflects a diverse range, encompassing density-based (HDBSCAN), centroid-based (KMeans), graph-based (Phenograph), self-organizing map-based (FlowSOM), and tree-based density-normalized (SPADE) clustering methods. This ensures a comprehensive comparison, considering different clustering paradigms and their suitability for various data structures.

As demonstrated here, HDBSCAN, KMeans (*K*=8), Phenograph, FlowSOM, and SPADE encounter challenges in robustly detecting rare cell populations that coexist with more abundant cell populations in the same sample (Fig. 2B–D). When clustering algorithms operate across multiple dimensions simultaneously, they may face difficulties in effectively detecting and distinguishing sparse populations from more prevalent ones. The increased sparsity within the vast high-dimensional space poses a challenge in identifying clusters that exist in lower-dimensional subspaces. Our findings illustrate that even with 7 dimensions (excluding live/dead PI from clustering), clustering algorithms may encounter challenges when dealing with multiple dimensions simultaneously.

Our findings suggest that the APP clustering method excels in scenarios where there are clear distinctions between the cluster under consideration and the other cells in at least one dimension. The APP method’s proficiency in identifying clusters with evident separations in one or more dimensions makes it well suited for situations in which distinct cell populations exist. In such cases, it can leverage the dimension(s) where the separation is apparent to successfully identify and differentiate clusters, even in the presence of much larger populations and noise in the data. This aligns with scenarios resembling cell phenotyping using flow/mass cytometry, imaging antibody panels, and scRNA-seq data, where there are often identifiable patterns or markers distinguishing cell types. Another application, as demonstrated here, is clustering molecules, such as TCRs and their cognate peptides, based on their sequence similarity and other features.

On the other hand, high-dimensional clustering approaches may potentially outperform the APP method when there is no clear split between clusters in any of the dimensions, and the information about a given cluster is “distributed” across multiple dimensions. High-dimensional clustering algorithms excel in aggregating information from multiple dimensions simultaneously, which can be advantageous in situations where cluster boundaries are less well defined. An example of this is the classification of cells’ activation states.

To mitigate some of the limitations of APP in scenarios with less clear cluster separations, we explored incorporating dimensionality reduction techniques, such as PCA, as a preprocessing step. As detailed below, this approach proved effective in revealing underlying structures in high-dimensional data.

### Application to flow and mass cytometry data

The ability to simultaneously measure multiple cell parameters through high-dimensional flow cytometry has enabled transformative discoveries in medicine, including identifying new cell types and cellular mechanisms critical to disease pathology. As a result, high-dimensional flow cytometry became indispensable for biomedical and clinical research, medical diagnosis, and therapy assessment. The gold-standard approach to analyzing flow cytometry data relies on user-defined manual gating on sequential 2D data projections. However, as the technology evolves and the number of cell parameters that can be measured simultaneously drastically increases, it becomes impractical and often impossible to rely on user-defined manual gates.

To address this limitation, the flow cytometry community has developed new automated methods that simultaneously analyze multiple cell parameters in high-dimensional space [8, 48]. Much effort has been directed to standardizing these automated methods and developing guidelines for the user, including through the flowCAP initiative [49]. However, although these methods proved helpful in some studies, reproducibility has become a major concern because cell types and phenotypes were often not preserved across different methods. As the community recognizes the limitations of the automated high-dimensional analysis, including the curse of dimensionality, the original sequential manual gating in 2D data projections has remained the de facto gold standard. Therefore, there is an emerging need to develop new automated and scalable data analysis approaches that leverage the ground truth of 2D manual gating.

To address this need, we developed APP for unsupervised discovery of cell populations and an automated label transfer pipeline for expert-defined gating automation. We expect APP to facilitate the reproducible analysis of large-scale datasets that rely on automated gating strategies on sequential 2D projections of the data, mitigating user-defined subjective gating and avoiding the curse of dimensionality that affects other methods.

**Figure 3:**
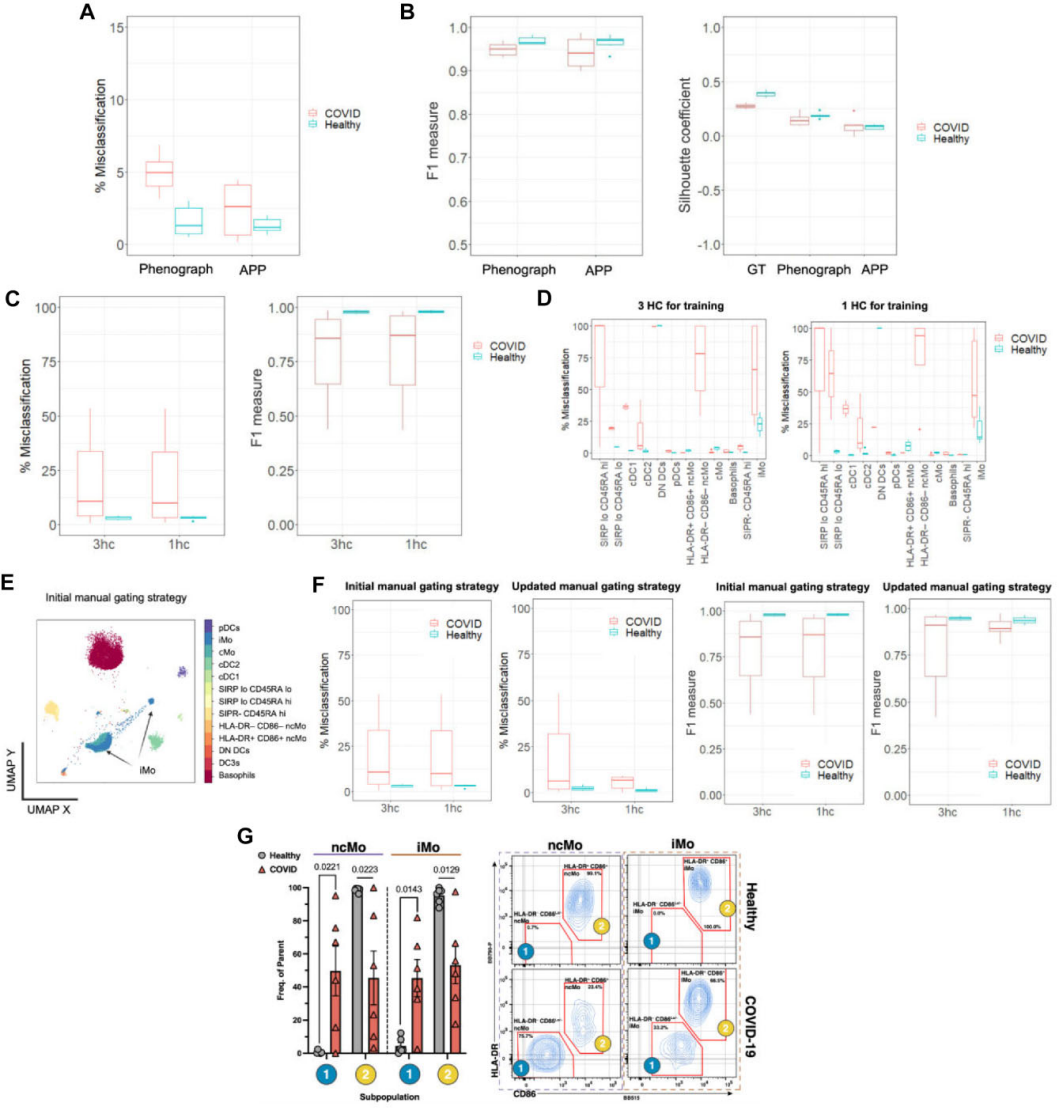
The label transfer pipeline reveals distinct cellular responses in COVID-19 patients and achieves over 99% accuracy in automating the manual gating strategy for healthy control samples, using minimal training data. (A) On average, less than 3% of the data were misclassified when comparing APP clustering decisions to the manual ground truth labels. (B) Comparison of F1-measure and the Silhouette coefficient between Phenograph and APP. “GT” refers to ground truth. (C) The automation of the gating strategy application, implemented via the automated label transfer pipeline, achieves very high accuracy for healthy control (HC or hc) samples. (D) Misclassification evaluation on a per-cell-population basis enables the identification of populations that are most problematic for the automated gating strategy transfer, providing targeted insights for improvement. Cell populations are ordered from most abundant to least abundant (left to right). (E) The label transfer pipeline facilitates the observation of agreement or disagreement between data topology and ground truth cluster labels. It provides insights into the accuracy of ground truth labels and serves as a quality control step. Detected discrepancies (e.g., iMo cell population is more heterogeneous than defined by the original manual gating strategy) may prompt re-evaluation or refinement of annotations (F), resulting in the discovery of biologically meaningful cell populations (G, HLA-DR-CD86lo ncMo and iMo populations that are unique to COVID patients). Minimum cluster size and bin size input parameters of 100 and 50, respectively, were used for both APP and the label transfer pipeline to generate the results presented in (A)–(G).

Using an expert-defined manual gating strategy as the “gold standard” (Supplementary Fig. 5), we evaluated the performance of APP in characterizing PBMCs from healthy donors and COVID-19 patients. APP demonstrated an overall performance accuracy exceeding 95%, significantly outperforming one of the widely used clustering algorithms in the flow cytometry field, Phenograph (Fig. 3A and B). The primary source of misclassification for both algorithms stems from sparse cell populations (Supplementary Figs 6 and 7). A detailed comparison (Supplementary Figs 6, 7) reveals that APP and Phenograph may misclassify different portions of the data, and this discrepancy can be attributed to the distinct clustering logic used by the 2 methods.

Furthermore, we assessed the label transfer pipeline’s capability to automate the expert-defined gating strategy. The pipeline achieved remarkably high performance for the healthy control cohort, exceeding 99%. This level of accuracy was attained even when using just one randomly chosen, manually gated, healthy control sample as a training set (Fig. 3C and D). In the analysis of COVID-19 samples, there were more discrepancies between the manual gating strategy established on healthy control samples and the label transfer pipeline’s outcomes. However, as shown here, this apparent discrepancy may indicate that the original expert-defined gating strategy, established on healthy control samples, might require adjustment when applied to disease samples. For example, as demonstrated here, COVID-19 samples exhibit more complex and diverse cell populations than the healthy control samples used to establish the initial manual gating strategy. COVID-19 induces changes in immune cells, leading to transitional cellular states that are dynamic and vary across patients based on factors such as demographics, treatment regimen, comorbidities, viral load, and the time from infection to analysis. As a result, samples from COVID-19 patients are expected to show greater variability compared with healthy controls. However, we recently demonstrated that despite this variability, ICU patients with severe COVID-19 remain distinguishable from healthy controls using flow cytometry to characterize major immune lineages and cellular states [20].

Our label transfer pipeline includes an immediate sanity check to assess the quality of the “ground truth” labels. This check examines whether the ground truth labels align with the underlying data topology. If there is a disagreement, such as a cell population defined as homogeneous in manual gating but being spread across multiple clusters on the supervised UMAP plot, it suggests a potential issue with the original gating strategy (Fig. 3E). Indeed, revisiting the original expert-defined gating strategy, as illustrated in Fig. 3F and G and Supplementary Fig. 5B, led to the discovery of a COVID-19-specific cell population labeled as HLA-DR–, CD86Lo/– intermediate monocytes (iMo).

Thus, APP identified a new population of myeloid cells specifically enriched in hospitalized COVID-19 patients. Notably, this myeloid cell subset lacks cell-surface expression of key proteins relevant for antigen presentation (HLA-DR) and co-stimulation of T-cells (CD86), likely affecting viral antigen presentation and T-cell activation. Although our new findings might represent a novel mechanism of immune modulation in severe COVID-19, further studies with more patients and different disease states are required to determine whether this is a general mechanism of SARS-CoV-2 infection or a phenotype unique to our small patient cohort.

The label transfer pipeline was also tested in its application to mass cytometry data, achieving 87% accuracy (Supplementary Fig. 8) compared with the manual gating labels described in [21]. Discrepancies between the underlying data topology and manually assigned cell populations (Supplementary Fig. 8) were a source of reduced accuracy for the label transfer pipeline.

### Application to scRNA-seq data

In contrast to flow and mass cytometry, which measure dozens of dimensions for each individual cell, the dimensionality of gene expression data is often on the order of thousands of genes per cell. Given the high-dimensional nature of gene expression data, dimensionality reduction techniques, such as PCA (principal component analysis), are often used as a pre-processing step before clustering to extract meaningful patterns and reduce the computational complexity associated with analyzing a large number of genes. We decided to test APP clustering performance on PCA-preprocessed scRNA-seq data because dealing directly with the combinatorial combinations of all possible pairwise projections from thousands of genes can be computationally prohibitive.

**Figure 4:**
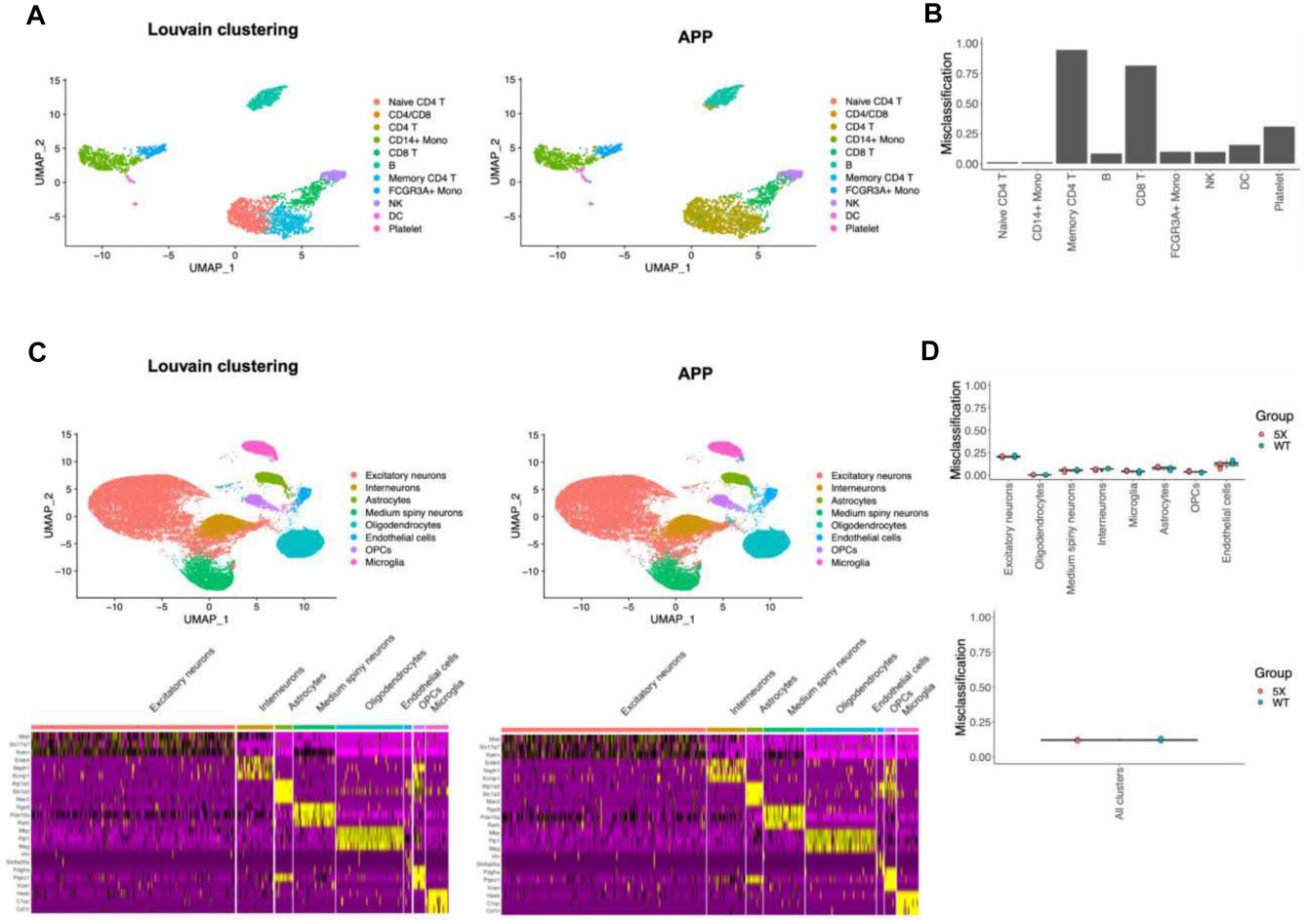
Both Louvain clustering and APP clustering, applied to PCA-reduced scRNA-seq data, exhibit good alignment with each other. The overall misclassification rate between Louvain and APP clustering decisions in the PBMCs dataset is about 29%. The misclassification rates are determined with reference to the “ground truth” clusters, indicating how many cells in each ground truth cluster produced by Louvain were marked as misclassified. APP clustering encounters challenges in distinguishing between CD4 Naive and Memory cells in the dataset (A, B). The Silhouette coefficient is 0.28 for Louvain and 0.23 for APP clustering outcomes. (C) There is a high degree of concordance (approximately 87%) between Louvain and APP clustering when applied to pooled combinations of wildtype (WT) and Alzheimer disease model (5X) mice samples. The Silhouette coefficient is 0.3 for Louvain and 0.2 for APP clustering outcomes. Heatmaps illustrate the concordance of gene expression patterns in each cluster type as identified by the 2 clustering algorithms. (D) Per cell type misclassification, providing insights into specific cell types for which misclassification occurs.

For this purpose we choose a publicly available scRNA-seq dataset generated from human PBMCs and processed by 10x Genomics (Pleasanton, CA, USA). We compared APP clustering performance to the performance of a clustering process using KNN and Louvain algorithms as implemented in the R package Seurat (referred to in this manuscript as “Louvain clustering,” and for our purposes, treated as the ground truth because it is a workflow widely used by the field today). The overall misclassification rate between Louvain clustering and APP clustering decisions in the PBMCs dataset is about 30%, but less than 15% overall when memory CD4 T cells are excluded (Fig. 4A and B). APP clustering faces challenges in resolving the distinction between Naive and Memory CD4 T cells in the dataset (Fig. 4B), likely due to the fact that these cells essentially represent distinct functional states and responses from the same population of cells. There is no clear split between these two populations in any pairwise dimensions explored by APP (see Supplementary Data on Zenodo) because the differentiation between Naive and Memory CD4 T cells may rely on simultaneous changes in several genes, and that gene set can vary based on factors such as the context of the immune response or the microenvironment.

In our analysis, we further examined the group of cells that Louvain clustering identified as B cells and APP as T cells, labeled as “CD4/CD8” in Fig. 4A. We projected this cell population in B cell, T cell, and other marker space to gain insights into the APP algorithm decision logic (see Supplementary Fig. 9). It becomes apparent that while the “CD4/CD8” cell population exhibits high expression of MS4A1, a B-cell-specific marker, it also demonstrates relatively high expression of S100A4, which is a memory CD4 T cell marker, not a B-cell-specific marker. This dual expression pattern likely contributed to the source of confusion for APP clustering.

Louvain clustering and APP clustering were next applied to a snRNA-seq mouse brain dataset with wild-type and Alzheimer disease model samples (Fig. 4C). The overall mismatch rate between the 2 methods was about 13%, indicating a good level of agreement (Fig. 4D). In this dataset, we did not see any “ground truth” clusters that could not be discovered through APP clustering, as we did in the PBMC dataset. All Louvain clusters had equivalent APP clusters with matching marker genes (Fig. 4C). The significant areas of discrepancy tended to be in cells that were assigned to their original Louvain clusters with some level of ambiguity.

To assess the computational complexity of the APP algorithm, which is generally *O*(*N*^2^), where *N* is the number of dimensions, we recorded the clustering time for APP versus Seurat v. 5 using a MacBook Pro with a 2.3 GHz Dual-Core Intel Core i5 and 8 GB memory. For the PBMC dataset (containing 2,700 cells), Seurat clustering (resolution = 0.5) took 1–2 seconds in real time. APP, with 10 PCs and a minimum cluster size of 10, took 11 mim 19.952 sec real time, 26 min 6.587 sec user time, and 2 min 43.988 ses system time. APP, with 10 PCs and a minimum cluster size of 100, took 2 min 16.023 sec real time, 5 mim 47.742 sec user time, and 0 min 34.924 sec system time.

### Application to multiplex imaging data

Recent advancements in multiplex imaging technologies, exemplified by SignalStar, have greatly enhanced our capacity to profile individual cells within the tissue context. These technologies enable the simultaneous visualization of multiple biomolecules at the single-cell or even the subcellular level. This capability provides valuable insights into cellular heterogeneity, spatial organization, and tissue composition.

**Figure 5:**
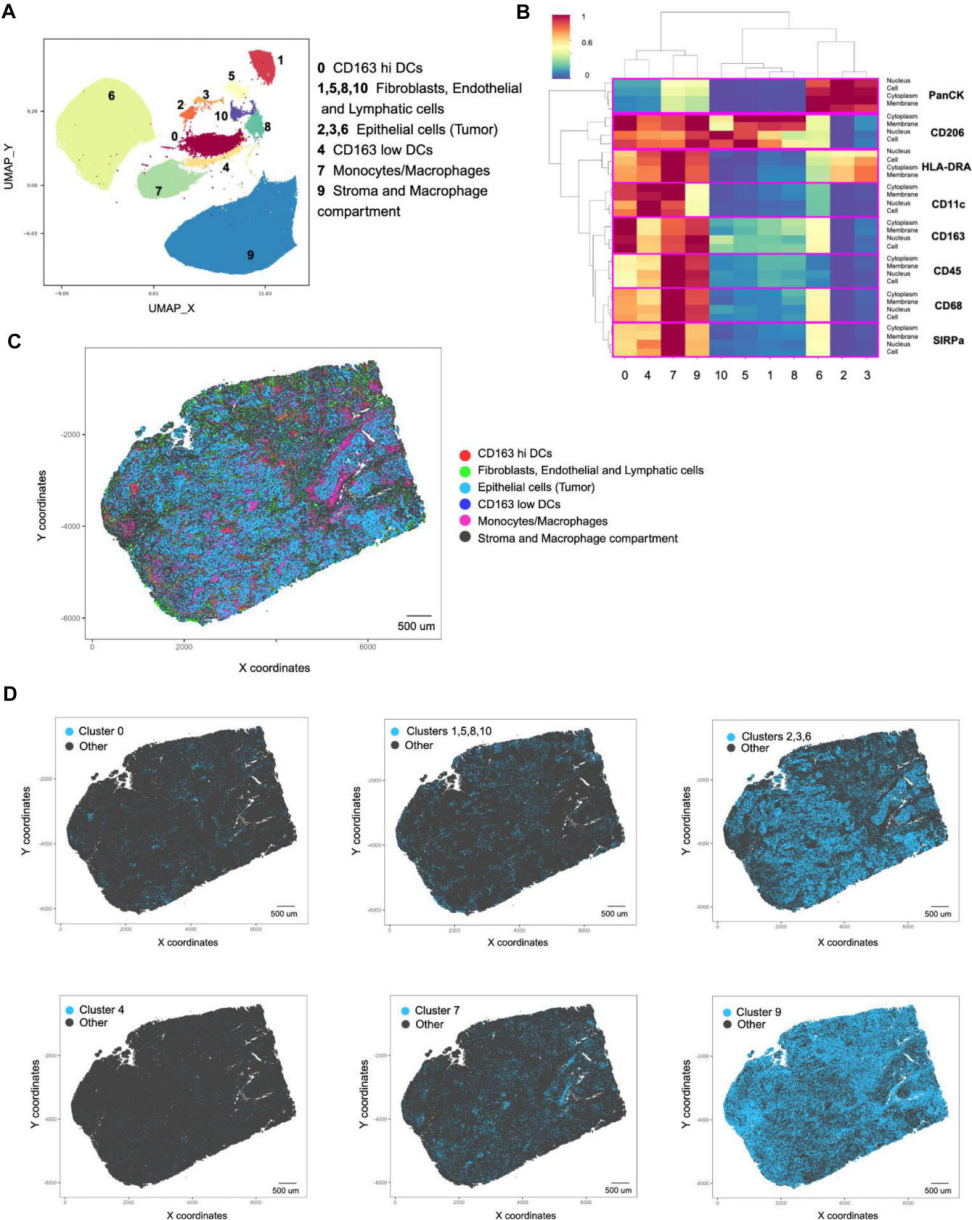
APP clustering successfully leverages multiplexed imaging data to gain insights into biologically meaningful cell populations within the complex tissue context of a human squamous lung carcinoma sample. Eleven cell clusters detected by APP (A) were annotated based on the pattern of median marker expression (B) and their spatial location (C,D). *x,y* Cartesian coordinates represent the position of a tissue sample on a glass slide. The 0–1 scale in (B) represents normalized expression, with each row normalized independently.

We applied APP clustering to characterize cellular composition within the tissue context of the human squamous lung carcinoma sample using the 8-plex SignalStar myeloid cell panel. The 8-plex panel (CD11c, SIRPɑ, CD163, CD206, CD68, CD45, HLA-DRA, and Pan-Keratin) generated a dataset of 160 features per cell for approximately 230,000 cells. The 160-feature set comprises 8 antibodies × 5 statistics × 4 cell compartments. Here, the 5 statistics are the measurements of marker mean, median, maximum, minimum, and standard deviation, and the 4 cell compartments are the nucleus, cytoplasm, membrane, and whole cell. We used PCA to reduce the dimensionality of the dataset to 30 dimensions and subsequently performed APP clustering (Fig. 5A and B).

This clustering approach readily identifies various cell types, including tumor epithelium, stromal, and immune cells. The marker composition of the panel reflects the functional heterogeneity within the myeloid compartment: populations of macrophages and monocytes (clusters 9 and 7), typically identified by prominent expression of CD68 and CD206, can further be subdivided based on high vs low expression of CD11c and SIRP-alpha [50]; similarly, CD11c+ dendritic cells (DCs) can belong to CD163-high or CD163-low (clusters 0 and 4, respectively), functionally distinct populations [51–53].

The representation of individual clusters (Fig. 5A) not only demonstrates phenotypic heterogeneity but also provides valuable clues regarding the relative proportion of phenotypes. Macrophages are known to be prominent cell populations within the microenvironment of diverse human tumor types including NSCLC [54, 55], and based on the size of the combined clusters, macrophages are a highly prevalent cell type in the tumor microenvironment of this squamous NSCLC. PanCK expression identifies epithelial cells (in this particular case, cancer cells) unambiguously, and the majority of PanCK+ cells seem to be low or negative for all other markers (cluster 6). However, two smaller PanCK+ clusters, 2 and 3, are identified and show elevated levels of HLA-DR; induction of HLA-DR expression in malignant epithelial cells is a known phenomenon, particularly in an inflammatory milieu [56, 57]. Interestingly, cells of these two clusters seem to preferentially localize to the epithelial–stromal interface with physical proximity between epithelial and immune cells and a potentially high local concentration of inflammatory cytokines.

Cell type label assignments were determined through a combination of marker expression patterns and the spatial location of identified clusters on the tissue slide (Fig. 5C and D). Because this dataset lacks ground truth labels, assessing clustering results requires considering both the expression patterns of panel markers and the spatial distribution and co-location of identified cell phenotypes within the tissue context. Therefore, domain experts evaluated the biological relevance of APP clustering decisions. Pathology and immunology experts (see Acknowledgments) independently assessed and confirmed the adequacy of APP clustering in characterizing and distinguishing meaningful cell populations within the slide tissue. ScRNA-seq or spatially resolved RNA-seq from the same sample or a serial section could serve as an orthogonal approach to assess cluster quality. However, we did not have further access to the sample used in this study for additional analysis.

### Application to TCR-peptide sequence representation data

Recent advancements in LLMs have shown significant promise for applications in the analysis of protein sequence data [58–60]. LLMs have been investigated for functional annotation of protein sequences [61], demonstrating capabilities in predicting protein functions, interactions, as well as antibody and TCR specificity [62, 63]. These models excel at capturing contextual relationships within sequences. In the context of TCR sequences, this proficiency involves understanding the specific arrangement of amino acids and their roles in recognizing and binding to antigens. Analyzing the language-like patterns in TCR and antigen sequences using these models may unveil insights into binding specificity.

**Figure 6:**
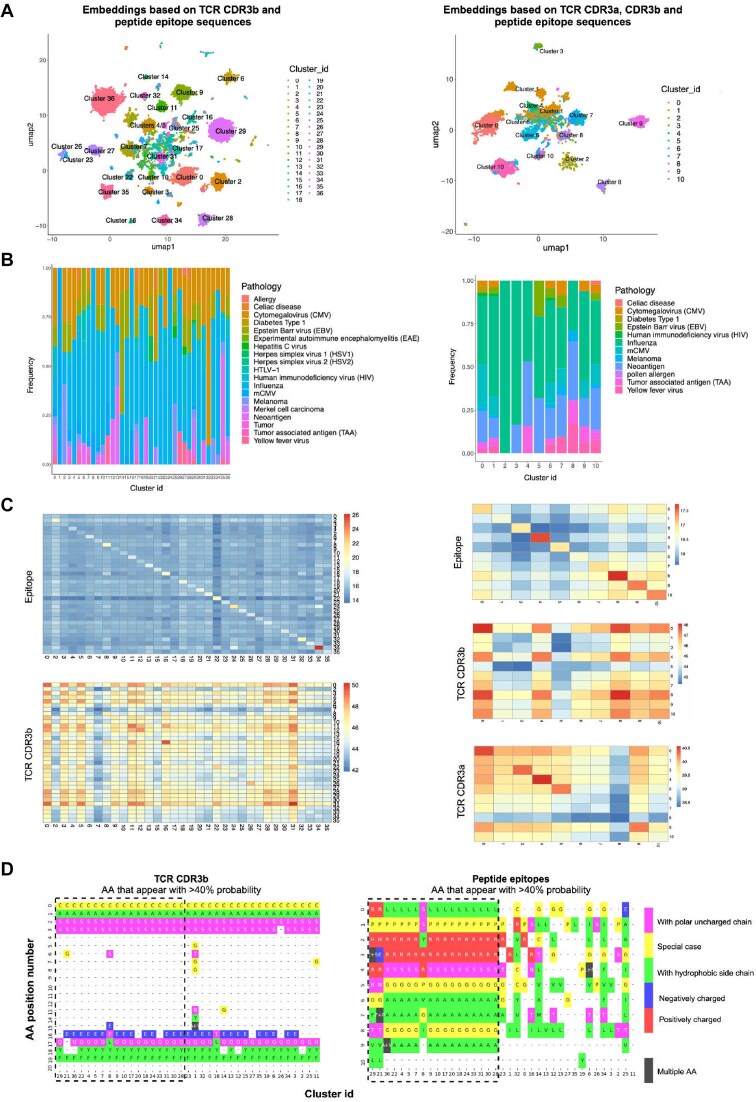
There is no identifiable common binding motif or a universal amino acid R group attribute characterizing the pMHC–TCR interaction. The clusters annotated by APP (A) are, in most cases, heterogeneous in terms of associated pathology, but some clusters are disproportionately enriched for particular pathologies, such as Epstein–Barr virus in cluster 14 or cytomegalovirus in cluster 32 in the clustering based on CDR3b and epitope sequences (B). Half of the pathologies in this dataset are associated with multiple unique epitope sequences, with an average of 8 unique epitope sequences per pathology. When sequence similarity scores are calculated for each pair of unique epitope sequences in the dataset and those scores are averaged across each unique pair within a cluster and and across each unique pair between all pairwise clusters, average similarity scores are systematically higher within clusters—on the diagonal line in these heatmaps—than between clusters. The same pattern is observed for CDR3b and CDR3a sequences to a lesser degree (C). Note that cluster 2 from the CDR3b, CDR3a, and epitope clustering was removed from the epitope and CDR3a heatmaps because it contained <20 unique epitope sequences (all other clusters had between 41 and 125 unique epitope sequences), causing it to have average within-cluster scores high enough to visually overwhelm the heatmaps, so as to allow the patterns between other clusters to be more perceptible, and clusters 1, 14, and 36 were removed from the CDR4b and epitope clustering because their average within-cluster scores were also visually distracting outliers. Per cluster amino acid enrichment analysis (D) revealed that, while there is no singular characteristic universally defining TCR–pMHC interactions, specific clusters may demonstrate shared electrostatic patterns involving charged amino acids at the interface. A noteworthy example includes a set of clusters (highlighted with a dashed line) enriched with the LPRRSGAAGA peptide, characterized by positively charged amino acids.

Although LLMs showcase remarkable capabilities in capturing intricate patterns and relationships within sequences, their black-box nature can pose challenges to interpretability. To address this, clustering techniques can be employed to interpret LLM decisions. In this example, using human and mouse TCR sequences and their cognate antigens (Fig. 6), we illustrate that the combination of LLMs and APP clustering methods offers a synergistic approach for uncovering patterns, relationships, and semantic structures within large and high-dimensional TCR-antigen sequence datasets.

To illustrate this point, we created embeddings for over 13,000 unique data points containing TCR CDR3b and cognate epitope sequence information for over 350 unique peptides (left side of Fig. 6A). Additionally, we independently generated embeddings for a subset of approximately 4,000 unique TCRs with sequence information for both TCR CDR3a and CDR3b (right side of Fig. 6A). These embeddings were generated independently for each sequence class (CDR3a, CDR3b, and epitope) and then concatenated for CDR3b-epitope and CDR3a/b-epitope. PCA was subsequently performed, and the first 30 PCs were utilized for APP clustering, using a minimum cluster size of 100. The composition of identified clusters was characterized based on epitope content and association with disease information retrieved from the McPAS-TCR database (Fig. 6B).

By combining embeddings constructed independently for TCRs and peptides, we generated a fused representation that captures similarities on both the TCR and peptide sides (Supplementary Fig. 10). This approach highlights co-similarities between TCRs and peptides across multiple pairs, enabling exploration of the joint sequence space of TCR–peptide interactions. This comprehensive view could offer valuable immunological insights into the complexities of immune recognition, cross-reactivity, specificity, and diversity.

The inherent black-box nature of LLMs presents a challenge in immediately interpreting the rationale behind the grouping of data points within embeddings. Introducing a clustering approach to these embeddings and subsequently overlaying domain knowledge and interpretable feature information onto the clustering decisions provides an opportunity to partially unravel the decision-making process of LLMs.

One evident hypothesis to investigate was assessing whether sequence similarity played a significant role as one of the driving forces behind the clustering of TCR–peptide data points within the class-separated and the combined embeddings. This investigation aimed to uncover the impact of sequence similarity on the grouping patterns within the combined embeddings, providing valuable insights into the underlying factors influencing the model’s behavior. To evaluate this hypothesis, we calculated sequence similarity independently for peptides, TCR CDR3b, and TCR CDR3a among all pairwise combinations of clusters (see Fig. 6C and Supplementary Fig. 10).

The within-cluster average sequence similarity consistently displayed higher scores compared with intercluster average sequence similarity in all 3 cases, i.e., for peptides, CDR3a, and CDR3b. This is evident from the observed higher Blosum62 score diagonal pattern on the heatmaps (Fig. 6C, Supplementary Figs 10, and 11A,C). As depicted in Supplementary Figs 10 and 11, sequence similarity, particularly in cases of peptide epitopes, plays a significant role in data clustering when class-separate embeddings are constructed. A clear pattern of peptide clustering based on disease categories (pathogens vs cancers, etc.) is observed. However, once TCR and peptide embeddings are concatenated, the data points undergo significant rearrangement in the embedded space. The disease of origin becomes a less significant feature for peptides. Overall, while sequence similarity between peptide epitopes and between TCRs still contributes to clustering, it becomes less prominent, as evidenced by a significant decrease in the median Blosum62 score on the diagonal, particularly for epitope peptides.

Certain peptide groups, such as those within cluster 4 on the right side of Fig. 6B, originate from antigens associated with diseases that are not immediately related. Cluster 4 predominantly consists of peptides derived from cancer neoantigens and influenza. TCRs recognizing these peptides also exhibit a closer sequence similarity in their CDR3a and CDR3b regions within cluster 4 compared with TCRs outside of this cluster (Fig. 6C). This suggests a potential shared recognition pattern among TCRs responding to sequence-similar peptides from disparate diseases within the same cluster.

The clustering based on sequence similarity indicates a commonality in the TCR responses, implying a common recognition pattern that extends across distinct disease contexts. This observation could potentially be explained via TCRs’ promiscuity towards structurally similar epitopes, even if they originate from different antigens. This promiscuity could be facilitated by commonalities in binding motifs among TCRs. Nonetheless, our analysis, illustrated in Supplementary Fig. 11, reveals the absence of an immediately discernible common binding motif. This holds true even among CDR3b sequences that specifically recognize the same peptide epitope (LPRRSGAAGA). It is worth noting that the promiscuity works both ways, namely that a TCR can recognise multiple epitope peptides, but also an epitope peptide can be recognized by multiple TCRs. Our clustering results show that the recognition patterns can be very different because different peptide–TCR pairs corresponding to the same peptide are often sorted into different clusters (Supplementary Fig. 11).

By further exploring the joint sequence space, we observed a novel pattern of “similarity” between the data points. This led to significantly different within-cluster arrangements for both peptides and TCR CDR3b. The label transfer pipeline was used to quantitatively assess the degree of rearrangement that both peptide and TCR CDR3b classes undergo between the single class embeddings and concatenated embeddings (Supplementary Fig. 12). Only 23% of peptide epitope data (and 12% for TCR CDR3b) is arranged in similar patterns between the single class and concatenated embedding space. This suggests that, during the exploration of the joint sequence space of TCR–peptide interactions, a new rationale for data point similarity emerged that is not necessarily related to the origin of the antigen peptide.

In our pursuit of a deeper understanding of the arrangement of data points in the joint sequence space, we explored the possibility of utilizing the characteristic properties of amino acid R groups as a novel similarity criterion. However, we found no immediate patterns of complementarity or cluster-specific motifs defined by the properties of amino acid R groups (refer to Fig. 6D and Supplementary Figs 10 and 11). The diversity among TCRs and epitope peptides suggests that the mechanisms of interaction can vary significantly between individual TCR–peptide pairs. For instance, in Supplementary Fig. 13, we highlight a case where a positively charged residue from the peptide side interacts with the TCR chains through the formation of hydrogen bonds, underscoring the intricate and diverse nature of these molecular interactions.

An intriguing pattern did emerge, though, revealing a prevalence of negatively charged amino acid residues on CDR3b and positively charged residues on CDR3a chains across almost all identified clusters (see Supplementary Fig. 13). To delve into the potential significance of these charged residues, we meticulously examined 5 TCR-pMHC crystal structures (PDB accession numbers 3GSN, 3PQY, 1OGA, 3O4L, 5EUO). Upon closer investigation of these structures, it became evident that these charged amino acid residues play a role in stabilizing the interface between CDR3a and CDR3b, orienting partners to each other in 3D space. Most frequently, this stabilization occurs through hydrogen bonds, although there is also an instance of ionic interaction (Supplementary Fig. 13E). However, follow-up studies will be necessary to further validate the findings and strengthen these claims.

It is also important to note that while LLMs could potentially be biased by learning patterns from the limited training data provided, we have recently shown [64] that, regardless of the grouping approach (e.g., distance-based clustering, LLM), only a minority of TCRs form pure clusters predominantly composed of peptide-specific TCRs.

## Conclusions

Identifying robust patterns in high-dimensional data is a challenging and crucial task in various scientific fields. High-dimensional data often pose challenges such as the curse of dimensionality, where traditional methods may struggle due to increased sparsity.

Projection pursuit has been proposed as a potential approach to address or mitigate some of the challenges posed by the curse of dimensionality. As a concept, this technique has been around for several decades, and its development and application span across different fields such as statistics, machine learning, and data visualization. The idea of seeking interesting projections of high-dimensional data can be traced back to the 1970s and 1980s [3–5, 65].

While the concept of projection pursuit has been around for decades, high-dimensional clustering (such as HDBSCAN, KMeans, and SPADE) has recently gained more popularity and attention, especially as an approach to the computational challenges posed by modern data analysis. High-dimensional clustering methods are explicitly designed to directly address the task of grouping data points into clusters. Projection pursuit approaches were originally designed as powerful tools for exploring interesting projections, not clustering. Additionally, the projection pursuit approach is challenged by the many low-dimensional projections generated from modern high-dimensional data.

Here, we have integrated the principles of projection pursuit and clustering to create an automated projection pursuit clustering approach, designed to reveal noteworthy structures in high-dimensional data and assign cluster labels. The “projection” aspect of our approach involves orthogonal projection of high-dimensional data into a 2D space. The “pursuit” component is guided by the concept of, at each step, identifying a projection with the smallest data density distribution along the decision boundary. The automated clustering aspect is executed through the recursive and exhaustive application of the “projection” and “pursuit” steps. To facilitate the computational challenge associated with exploring a high number of low-dimensional projections, we reduced the number of operations required by the algorithm at each recursive step by adopting the calculation of the optimal number of histogram bins. We also implemented parallelization of the APP algorithm, enhancing its computational performance. Future improvements may focus on additionally optimizing specific algorithm components and/or exploring alternative computational strategies such as leveraging high-performance computing when a relatively large amount of high-dimensional data needs to be processed. Also, in some cases clusters that are well separated in higher-dimensional space may appear mixed when projected onto certain 2D planes. To address this, proper rotation of the coordinate system may help uncover projections that better reveal separation. Although our current approach does not explicitly incorporate rotation optimization, one potential future extension could involve employing rotation search methods such as those used in projection pursuit to identify projections that maximize separation between clusters.

In general, projection pursuit clustering can offer advantages in certain scenarios compared with traditional high-dimensional clustering methods. Projection pursuit can be effective when certain dimensions or variables are more important for clustering than others. It actively seeks projections that highlight important features, focusing on relevant dimensions and potentially improving cluster separation.

In scenarios characterized by sparse or imbalanced data (such as the dataset presented in Fig. 2), where there is a high degree of sparsity amid more abundant populations (which can extend to situations involving outliers and/or noise), projection pursuit proves beneficial in identifying pertinent dimensions and enhancing cluster separation. Traditional methods (such as HDBSCAN, KMeans, Phenograph, and SPADE) may face challenges in handling sparsity (Fig. 2C) due to a dearth of informative features. Projection pursuit seeks to discover projections that are not only conducive to clustering but also interpretable. If the objective is to extract meaningful insights from clustering outcomes and comprehend the contributions of individual dimensions, projection pursuit may be the preferred choice.

The approach of discovering patterns through projection pursuit and clustering proves to be versatile across various data modalities. In this context, our focus was specifically on high-dimensional biological data given their frequent representation of both abundant and sparse populations within the same sample. We demonstrated APP’s capability to replicate experimentally validated cell type definitions, emphasizing the biological relevance of the clusters identified by our approach. We conducted a performance comparison of APP with other widely adopted clustering methods. Although APP’s results align well with other methods, there are instances where one method may outperform the other, and we illustrated these nuances using real-world datasets.

In many biological real-world datasets, the availability of a clear “ground truth” can be challenging. As illustrated in the examples presented, reliance on domain experts’ knowledge-driven clustering or clustering done with widely adopted approaches, serves as a substitute for ground truth. While expert-driven clustering provides a valuable reference point, a more accurate (albeit labor-intensive) method for assessing clustering performance involves conducting functional tests on groups of cells assigned to the same cluster. By observing the functional “purity” and homogeneity of a given cluster compared with other cell clusters in the sample, researchers can achieve a more precise evaluation of the clustering results.

In one such example, we used a dataset with functionally validated ground truth and demonstrated that APP effectively recapitulated experimentally validated cell-type definitions better than other widely used clustering approaches. We also presented APP’s utility in discovering new biologically meaningful patterns. By combining the strengths of LLMs for sequence analysis with the interpretability provided by clustering, we gained deeper insights into the complex relationships within TCR–pMHC sequence datasets. This integrative approach contributes to the elucidation of patterns that may have important implications for better understanding of TCR and pMHC function and design. Our results also emphasize the ongoing challenges with universal pMHC-TCR specificity machine-learning models, showing that the pMHC–TCR recognition patterns are not always self-evident.

## Availability of source code and requirements

Project name: APP

Project home page: https://github.com/tabatsky/projectionpursuit

Operating system(s): MAC, Windows

Programming language: Python

Other requirements: Requirements are listed at the project home page

License: MIT License

RRID:SCR_026560

Project name: phenoimager2mc

Project home page: https://github.com/SchapiroLabor/phenoimager2mc

Operating system(s): MAC, Linux

Programming language: Python

Other requirements: https://github.com/SchapiroLabor/phenoimager2mc/blob/main/environment.yml, optionally Docker or Singularity

License: AGPL-3.0 License

RRID:SCR_026561

## Supplementary Material

giaf052_Supplemental_File

giaf052_Authors_Response_To_Reviewer_Comments_original_submission

giaf052_GIGA-D-24-00440_original_submission

giaf052_GIGA-D-24-00440_Revision_1

giaf052_Reviewer_1_Report_original_submission Alexander Moskalensky -- 12/27/2024

giaf052_Reviewer_1_Report_Revision_1 Alexander Moskalensky -- 3/6/2025

giaf052_Reviewer_2_Report_original_submission Abdulhamit Subasi -- 1/3/2025

giaf052_Reviewer_3_Report_original_submission Yuri Vyatkin -- 1/6/2025

giaf052_Reviewer_4_Report_original_submission Ruslan Rust -- 1/6/2025

giaf052_Reviewer_4_Report_Revision_1 Ruslan Rust -- 3/6/2025

## Data Availability

The dataset of human squamous cell carcinoma stained with SignalStar mIHC is deposited in Mendeley Data [66]. All additional supporting data are available in the *GigaScience* repository, GigaDB [67].

## Additional Files

**Supplementary Figure S1:** Example of an optimal decision boundary search for one of the 2D data projections. The decision boundary begins at the left edge and ends at the right edge of a 2D projection. However, in *xy* vs *yx* orientations, the left and right edges differ, leading to distinct decision boundaries. Therefore, *xy* and *yx* projections should be analyzed independently.

**Supplementary Figure S2:** Workflow for the automated label transfer across samples. This pipeline utilizes labeled or partially labeled training samples with the marker expression data and ground truth cluster labels. It applies UMAP to learn a distance metric that optimally separates classes while preserving relationships in the marker space. The unlabeled test set is then projected into the UMAP embedding space built using the training set. The QFMatch algorithm aligns cluster labels between the test set and the training set for the downstream calculation for the number of misclassified events per cluster ID. Label transfer pipeline makes it possible to quantitatively compare and align cluster labels across training and test samples in cases where clusters may be absent in either the test (A) or training data (B).

**Supplementary Figure S3:** An example of a discrepancy between the data topology and clustering decisions. Data topology suggests cluster 11 is more heterogeneous than was originally defined by the clustering approach (manual gating, in this case).

**Supplementary Figure S4:** Schematic representation of the label transfer pipeline application to a quantitative comparison of decisions made by 2 clustering algorithms on the same dataset. We used a synthetic dataset consisting of a mixture of Gaussian distributions.

**Supplementary Figure S5:** Expert-defined manual gating strategies used for high-dimensional flow cytometry data. (A) Representative gating strategy of a randomly selected health donor sample to interrogate myeloid-derived cells (MdCs) enriched from PBMCs (see Methods). (B) The original gating strategy from (A) was refined following the label transfer pipeline “sanity check,” revealing an intermediate monocyte (iMo) population in COVID-19 patients that was not ubiquitously present in healthy donors, similar to non-classical monocytes (ncMo; see blue box and Fig. 3G). This figure was created using FlowJo v.10.8 (BD Biosciences).

**Supplementary Figure S6:** An illustration of misclassification by APP (A) and Phenograph (B) compared to manually gated cell population annotations. Misclassified events, calculated using the automated label transfer pipeline, are highlighted in black.

**Supplementary Figure S7:** Smaller cell populations are more prone to misclassification by clustering algorithms in both healthy donor (A) and COVID-19 patient samples (B).

**Supplementary Figure S8:** Discrepancies between the underlying data topology and the ground truth labels could negatively affect the performance of the label transfer pipeline. (A) One and three (out of 10) randomly chosen healthy control (hc) PBMC samples, characterized with the ∼30 marker CYTOF panel, were used as training sets and the rest of samples were used as the test set. (B) The primary source of misclassification arises from the more heterogeneous nature of cell populations than initially identified with the established expert-defined manual gating strategy. For instance, the gamma delta T-cell population in (C) is actually distributed between the 2 clusters, as data topology suggests.

**Supplementary Figure S9:** The expression of B and T marker genes in cell subsets identified within the PBMC dataset. MS4A1 is a B-cell-specific marker, the rest are CD4 and CD8 T-cell-specific markers. The group of cells that was misclassified by APP is here labeled “CD4/CD8.”

**Supplementary Figure S10:** Sequence similarity and amino acid R group properties analysis of ESM embeddings generated for peptide epitope sequences (A), TCR CDR3b sequences (C), and the concatenation of both TCR and peptide embeddings (E). Amino acids at a specific position within a designated cluster were categorized according to their R group properties (as indicated in the inserted legend). The most prevalent property within each group is presented, along with its corresponding probability (B,D,F).

**Supplementary Figure S11:** No common binding motif is identified on TCR CDR3b, even when considering similar peptide epitopes. The distribution of the 5 most common peptides (in the database utilized for this study) appears highly cluster-specific in the context of single-class embeddings (A). However, when using concatenated embeddings (CDR3b and peptide), these 5 peptides are dispersed across multiple clusters (B). (C) No discernible binding motifs appear in the TCR CDR3b, whether analyzed individually (left side) or as concatenated embeddings (right side). Even when focusing exclusively on CDR3b sequences specific to the LPRRSGAAGA peptide (D), these sequences exhibit no notable increase in sequence similarity (E), as approximated by the Blosum62 score, when compared with the CDR3b sequences clustered together in the concatenated embeddings data presented in Fig. 6.

**Supplementary Figure S12:** Concatenation of TCR CDR3b and peptide embeddings results in distinct similarity criteria captured by the ESM model. The automated label transfer pipeline was employed to align clusters obtained for the same class of sequences (peptides (A) and TCR CDR3b (B)) generated from the single-class embeddings and concatenated embeddings. The label transfer pipeline was executed on 30 PCs, using the APP cluster labels generated for the single-class embeddings as the training set. On the top row of (A, B), cluster IDs are shown before the cluster alignment, and thus the same color may represent 2 unrelated clusters on the left and right UMAP plots for each class. On the bottom row of (A) and (B), cluster IDs are shown after the match/cluster alignment, and thus the same color represents aligned clusters within the same sequence class. Non-matched clusters are shown in black. (C) Per-cluster-ID misclassification as estimated by the label transfer pipeline.

**Supplementary Figure S13:** The TCR CDR3b sequences exhibit an enrichment of negatively charged amino acids, while TCR CDR3a sequences are characterized by an enrichment of positively charged amino acids. (A) Amino acids that appear with more than 30% probability in a given position and in a given cluster are color-coded based on their R group property. These physicochemical maps are constructed for the 4,000 CDR3a–CDR3b–peptide dataset presented on the right side of Fig. 6. (B) Amino acids at a specific position within a designated cluster were sorted into the corresponding R group property, as specified in the legend. The most dominant property within each group is displayed, along with its associated probability. The results presented focus on R group property classes that exhibit a prevalence of more than 30% at a given position within a designated cluster. Analysis of these properties for the peptides showed important hydrophobic enrichment in all clusters (marked in green in the middle panel right). For 5 independent clusters, we found in the PDB 5 TCR–pMHC crystal structures and analyzed these. (C) All showed that the antigen directly interacted with both, alpha- and beta-TCR units. Unexpected for such hydrophobic peptides was the presence of hydrogen bonds, observed for all of them (from 2 to 8 hydrogen bonds) and involving both TCRa and TCRb. In the center of peptides, we noticed either the presence of one positively charged amino acid (often Arg), or the presence of one Gly. (D) Structure analysis of PDB 3PQY showed that Arg7 has its 3 nitrogens from the lateral chain involved in the formation of hydrogen bonds with TCRa (4 hydrogen bonds) and TCRb (2 hydrogen bonds). We suggest that through the formation of all the hydrogen bonds the peptide orients itself between the TCRs subunits and complements the stable 3D surface of the TCRs. Such hydrogen bonds also participate in the stability of the [peptide–TCRs] complex and the presence of Gly (B, middle panel right, “Peptide epitopes,” yellow) allows partial bending of the peptide, when necessary. (E) We noticed for all CDR3a and CDR3b the respective presence of conserved Lys and Glu (A, B). Analysis of the previous 5 structures showed that these amino acids do not interact directly with the peptide, or between themselves. They form hydrogen bonds or ionic bonds between the 2 TCRs and through this mechanism participate in the pairing of the 2 subunits (see 1 hydrogen bond between the conserved Glu102 (CDR3b) and Thr49 from TCRa; and the ionic bonds below between Arg97 (CDR3a) and Glu60 (TCRb)).

## Abbreviations

APP: Automated Projection Pursuit; ESM: Evolutionary Scale Modeling; ICU: Intensive Care Unit; LLM: Large Language Model; PCA: Principal Component Analysis; SVC: Support Vector Clustering; WT: Wild-Type.
