# Supplementary material for: Lifting the curse from high-dimensional data: automated projection pursuit clustering for a variety of biological data modalities
Source: Gigascience. 2025 May 29;14:giaf052. doi: 10.1093/gigascience/giaf052 (PMC12121483; doi:10.1093/gigascience/giaf052)
Supplement: giaf052_Supplemental_File [file giaf052_supplemental_file.pdf]

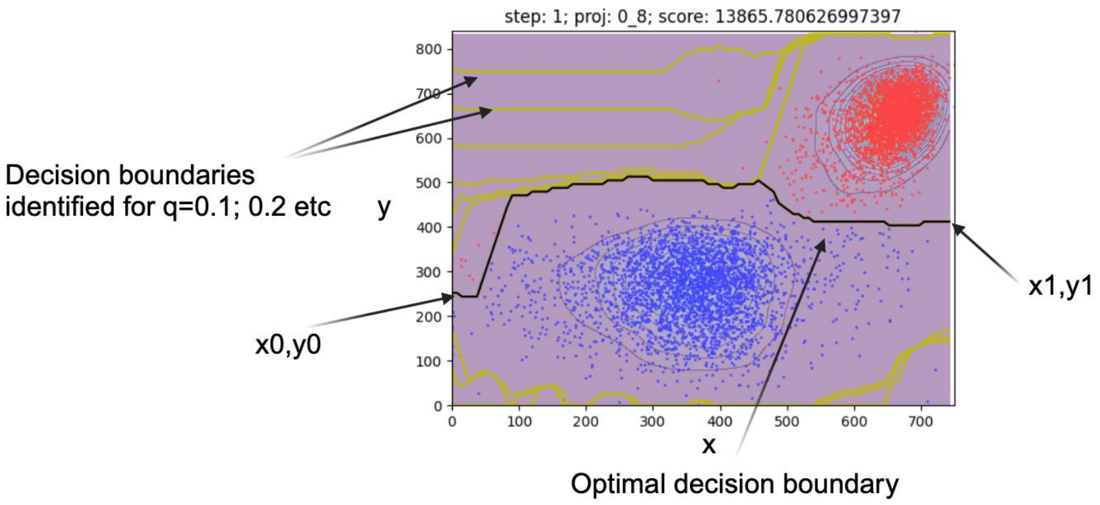

Supplementary Figure 1. Example of an optimal decision boundary search for one of the 2D data projections. The decision boundary begins at the left edge and ends at the right edge of a 2D projection. However, in xy vs yx orientations, the left and right edges differ, leading to distinct decision boundaries. Therefore, xy and yx projections should be analyzed independently.

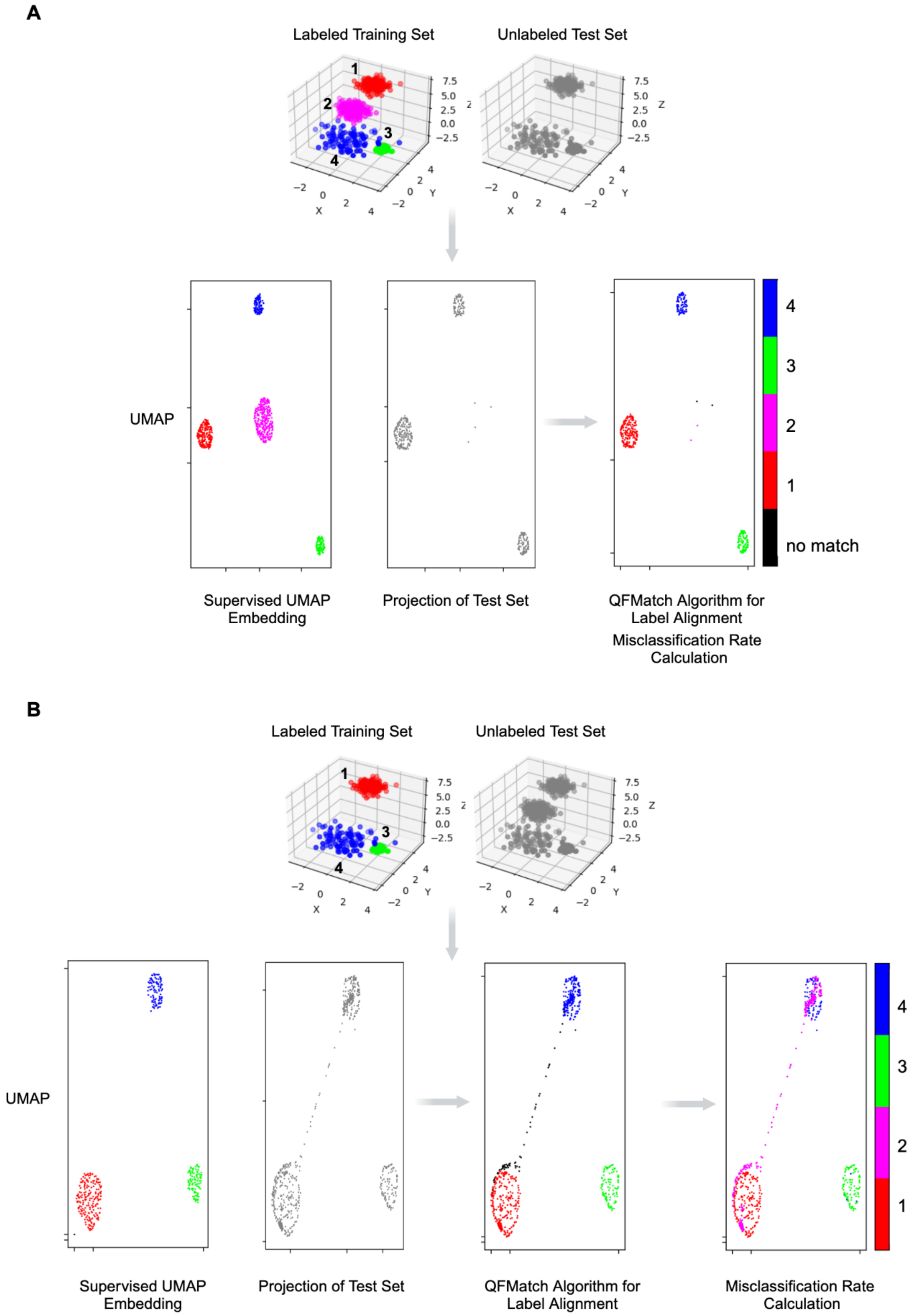
A
Labeled Training Set
Unlabeled Test Set
UMAP
Supervised UMAP Embedding
Projection of Test Set
QFMatch Algorithm for Label Alignment
Misclassification Rate Calculation
4
3
2
1
no match
B
Labeled Training Set
Unlabeled Test Set
UMAP
Supervised UMAP Embedding
Projection of Test Set
QFMatch Algorithm for Label Alignment
Misclassification Rate Calculation
4
3
2
1

Supplementary Figure 2. Workflow for the automated label transfer across samples. This pipeline utilizes labeled or partially labeled training samples with the marker expression data and ground truth cluster labels. It applies UMAP to learn a distance metric that optimally separates classes while preserving relationships in the marker space. The unlabeled test set is then projected into the UMAP embedding space built using the training set. The QFMatch algorithm aligns cluster labels between the test set and the training set for the downstream calculation for the number of misclassified events per cluster ID. Label transfer pipeline allows quantitatively comparing and aligning cluster labels across training and test samples in cases where clusters may be absent in either the test (**A**) or training data (**B**).

UMAP

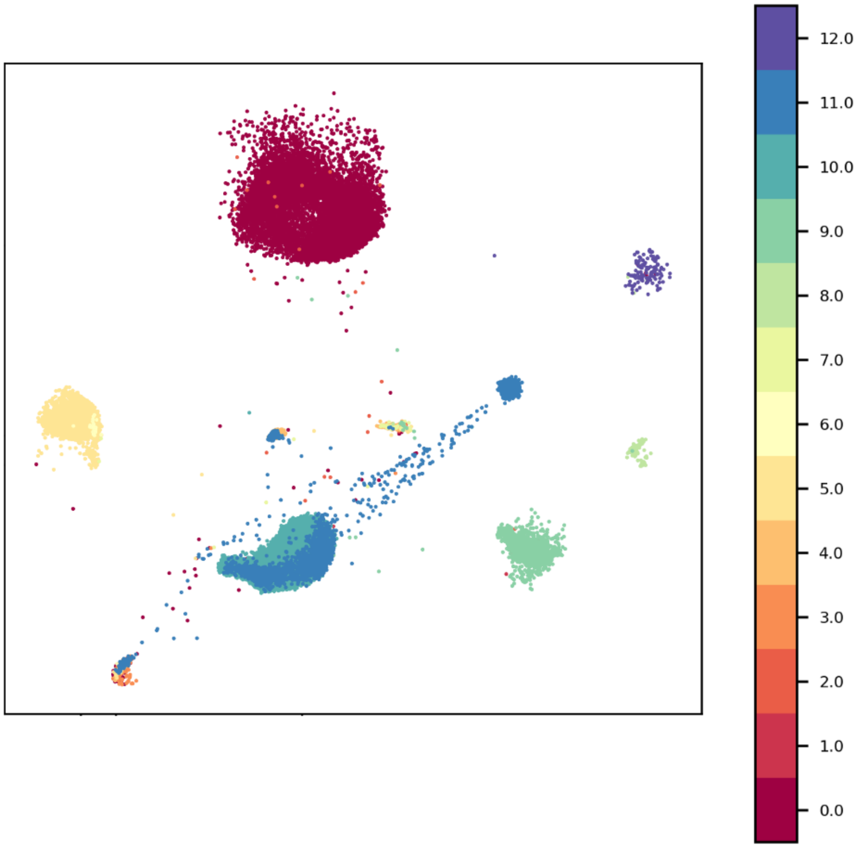

Supplementary Figure 3. An example of a discrepancy between the data topology and clustering decisions. Data topology suggests cluster 11 is more heterogeneous than was originally defined by the clustering approach (manual gating, in this case).

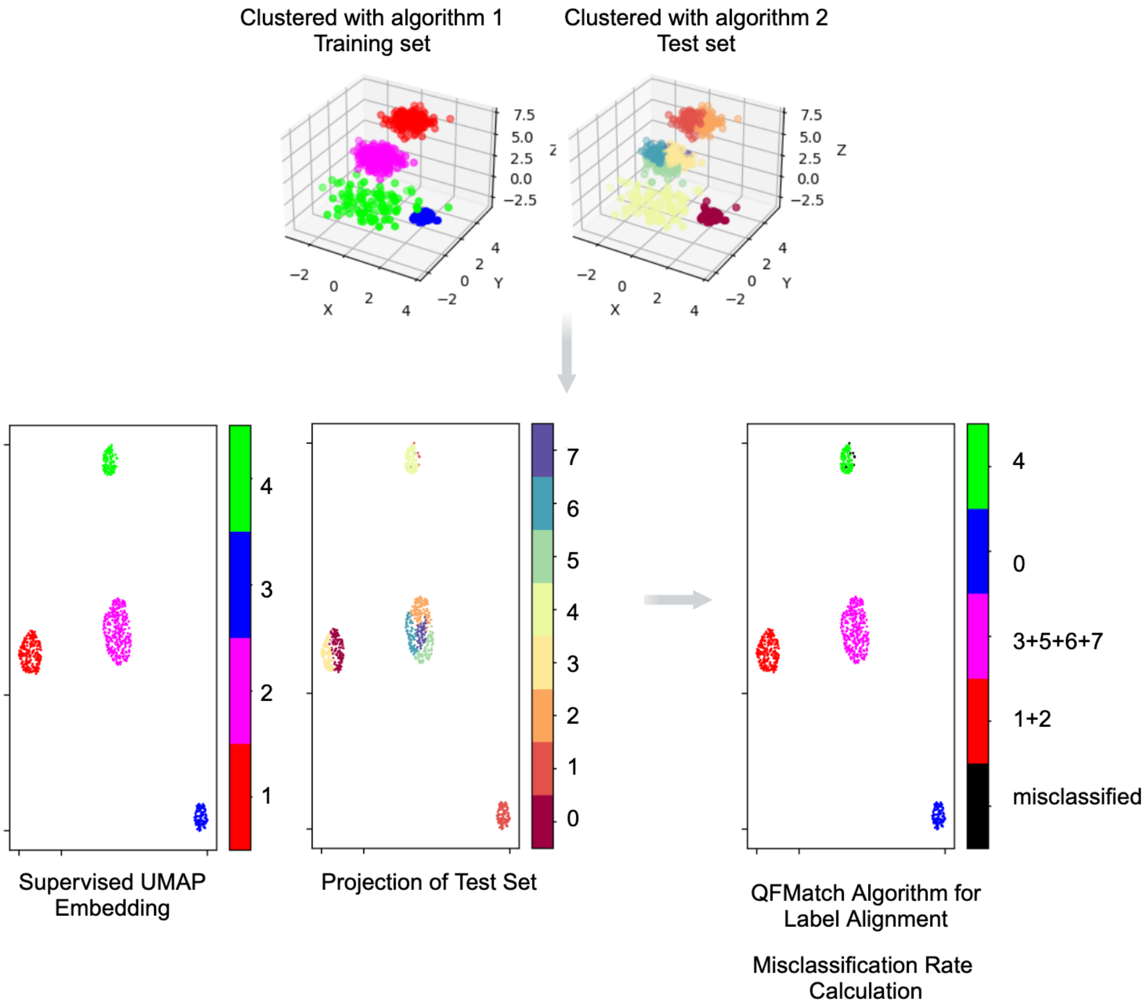

Supplementary Figure 4. Schematic representation of the label transfer pipeline application to quantitative comparison of two clustering algorithms decisions made on the same data set. We used a synthetic dataset consisting of a mixture of Gaussian distributions.

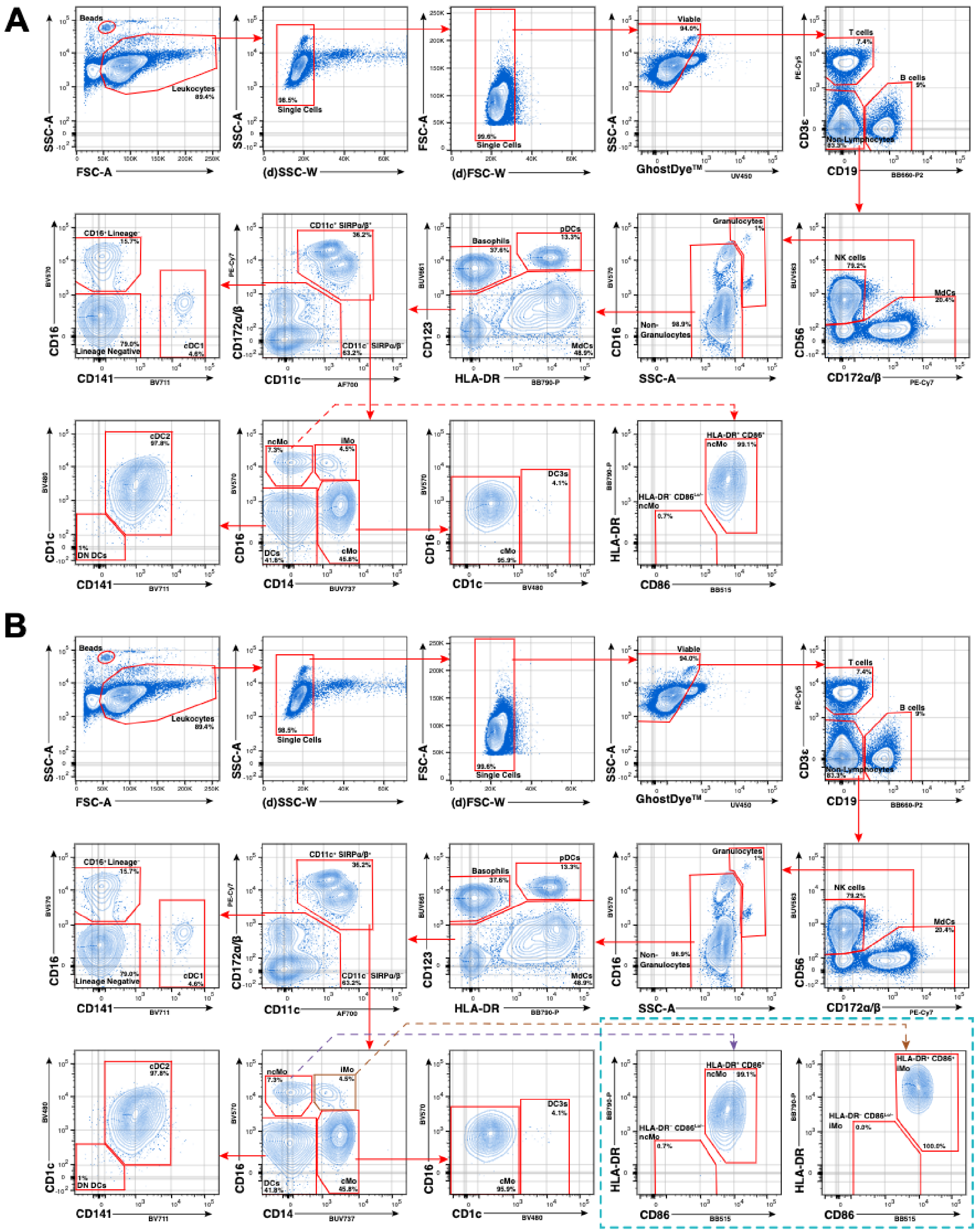
A
B
Beads
Leukocytes
89.4%
SSC-A
FSC-A
98.5%
Single Cells
(d)SSC-W
FSC-A
99.6%
Single Cells
(d)FSC-W
Viable
94.0%
GhostDye™
UV450
T cells
7.4%
B cells
9%
Non-Lymphocytes
83.3%
CD3ε
PE-Cy5
CD19
BB660-P2
CD16⁺ Lineage
15.7%
79.0%
Lineage Negative
cDC1
4.6%
CD16
BV570
CD141
BV711
CD11c⁺ SIRPα/β⁺
36.2%
CD11c⁻ SIRPα/β⁻
63.2%
CD172α/β
PE-Cy7
CD11c
AF700
Basophils
37.6%
pDCs
13.3%
MdCs
48.9%
CD123
BUV661
HLA-DR
BB790-P
Granulocytes
1%
Non-Granulocytes
98.9%
SSC-A
NK cells
79.2%
MdCs
20.4%
CD56
BUV563
CD172α/β
PE-Cy7
cDC2
97.8%
1%
DN DCs
CD1c
BV480
ncMo
7.3%
iMo
4.5%
DCs
41.8%
cMo
45.8%
CD14
BUV737
DC3s
4.1%
cMo
95.9%
CD1c
BV480
HLA-DR⁺ CD86⁺
ncMo
99.1%
HLA-DR⁻ CD86
ncMo
0.7%
HLA-DR
BB790-P
CD86
BB515
HLA-DR⁺ CD86⁺
iMo
HLA-DR⁻ CD86
iMo
0.0%
100.0%

Supplementary Figure 5. Expert-defined manual gating strategies used for high-dimensional flow cytometry data. A. Representative gating strategy of a randomly selected health donor sample to interrogate myeloid-derived cells (MdCs) enriched from PBMCs (see methods). **B.** Original gating strategy from (A) was refined following the label transfer pipeline "sanity check", revealing an intermediate monocyte (iMo) population in COVID-19 patients that was not ubiquitously present in healthy donors, similar to non-classical monocytes (ncMo; see blue box & Figure 3G). This figure was created using FlowJo™ v10.8 (BD Biosciences).

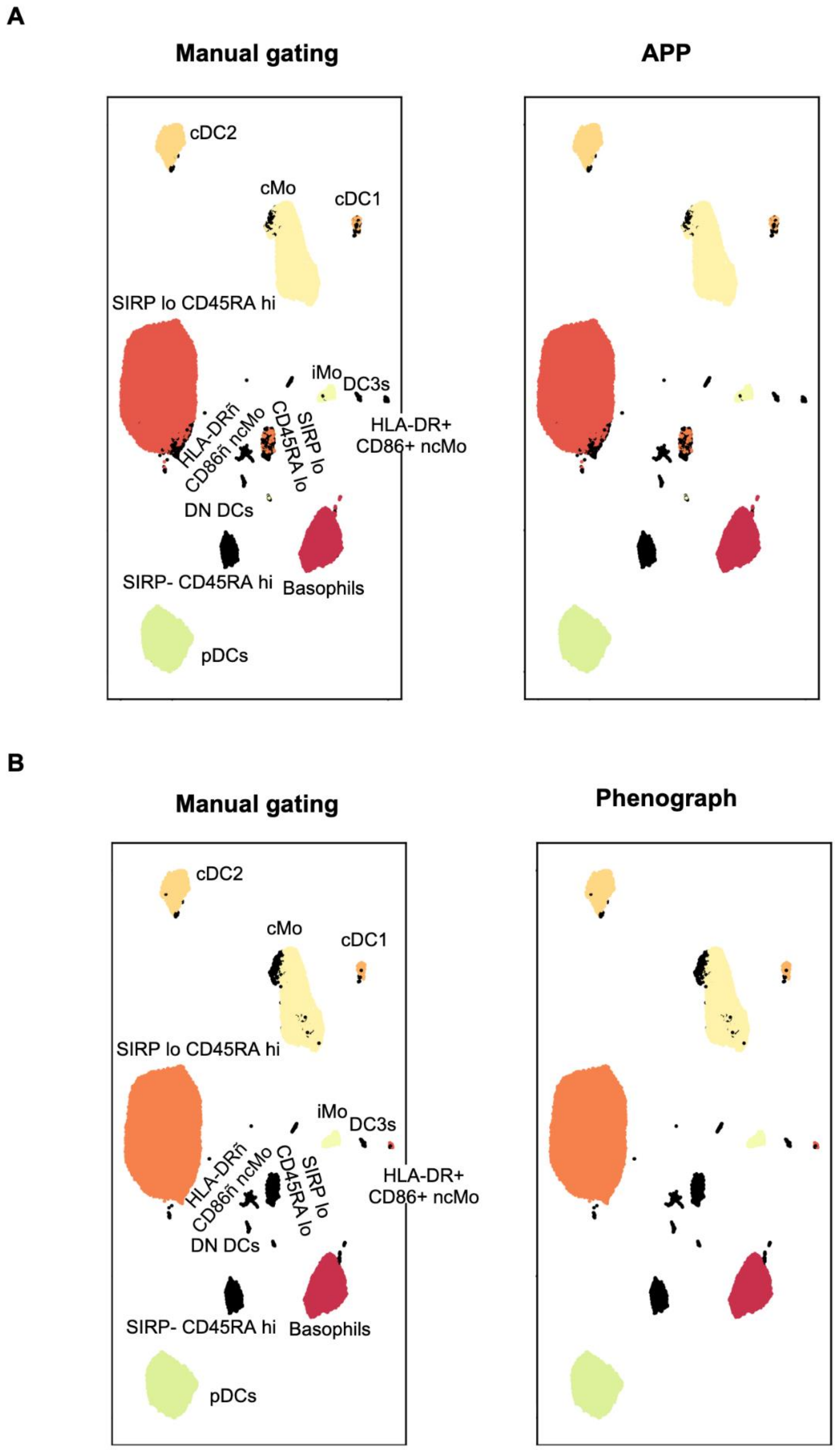

Supplementary Figure 6. An illustration of misclassification by APP (A) and Phenograph (B) compared to manually gated cell population annotations. Misclassified events, calculated using the automated label transfer pipeline, are highlighted in black.

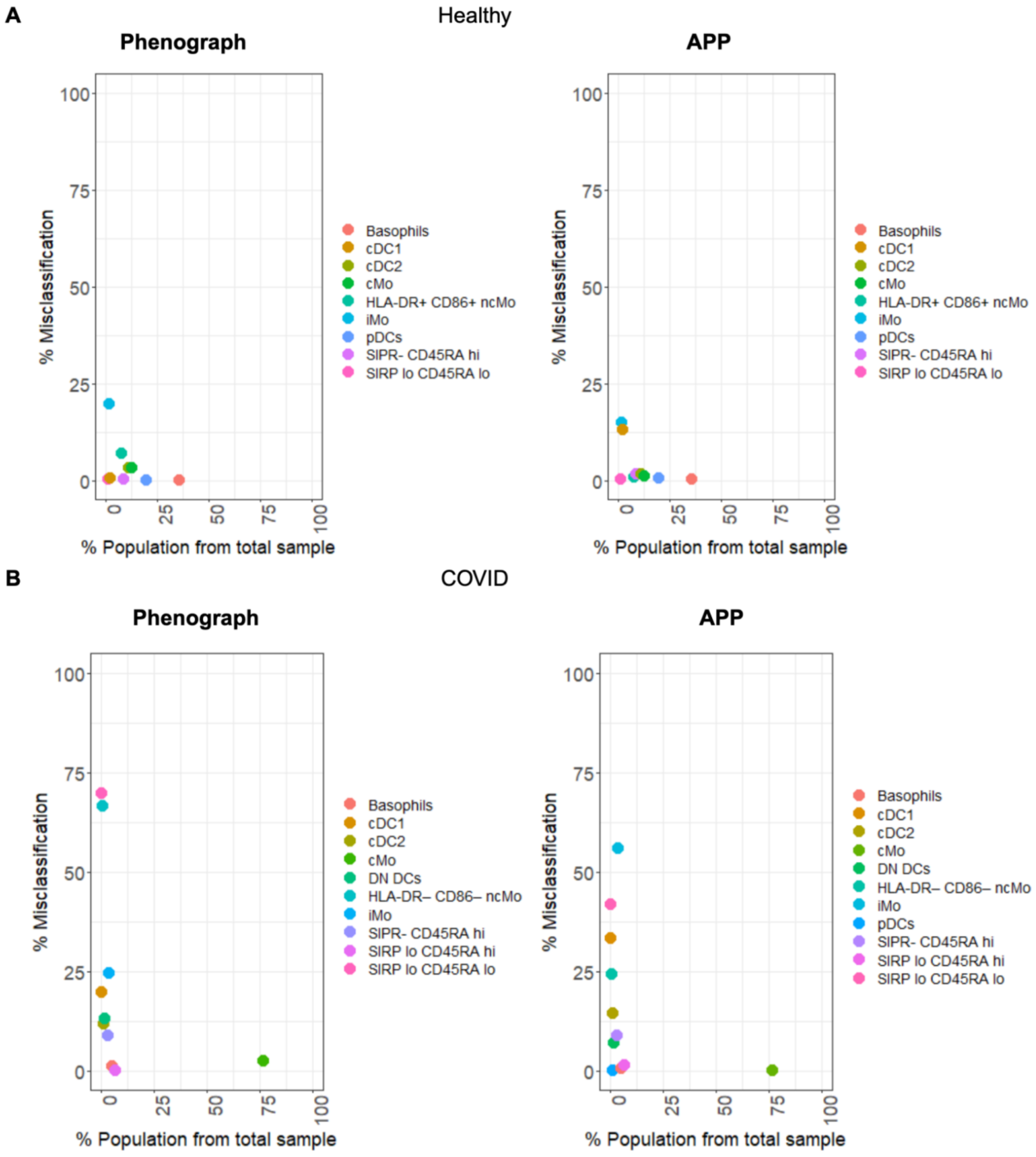

Supplementary Figure 7. Smaller cell populations are more prone to misclassification by clustering algorithms in both healthy donor (A) and COVID-19 patient samples (B).

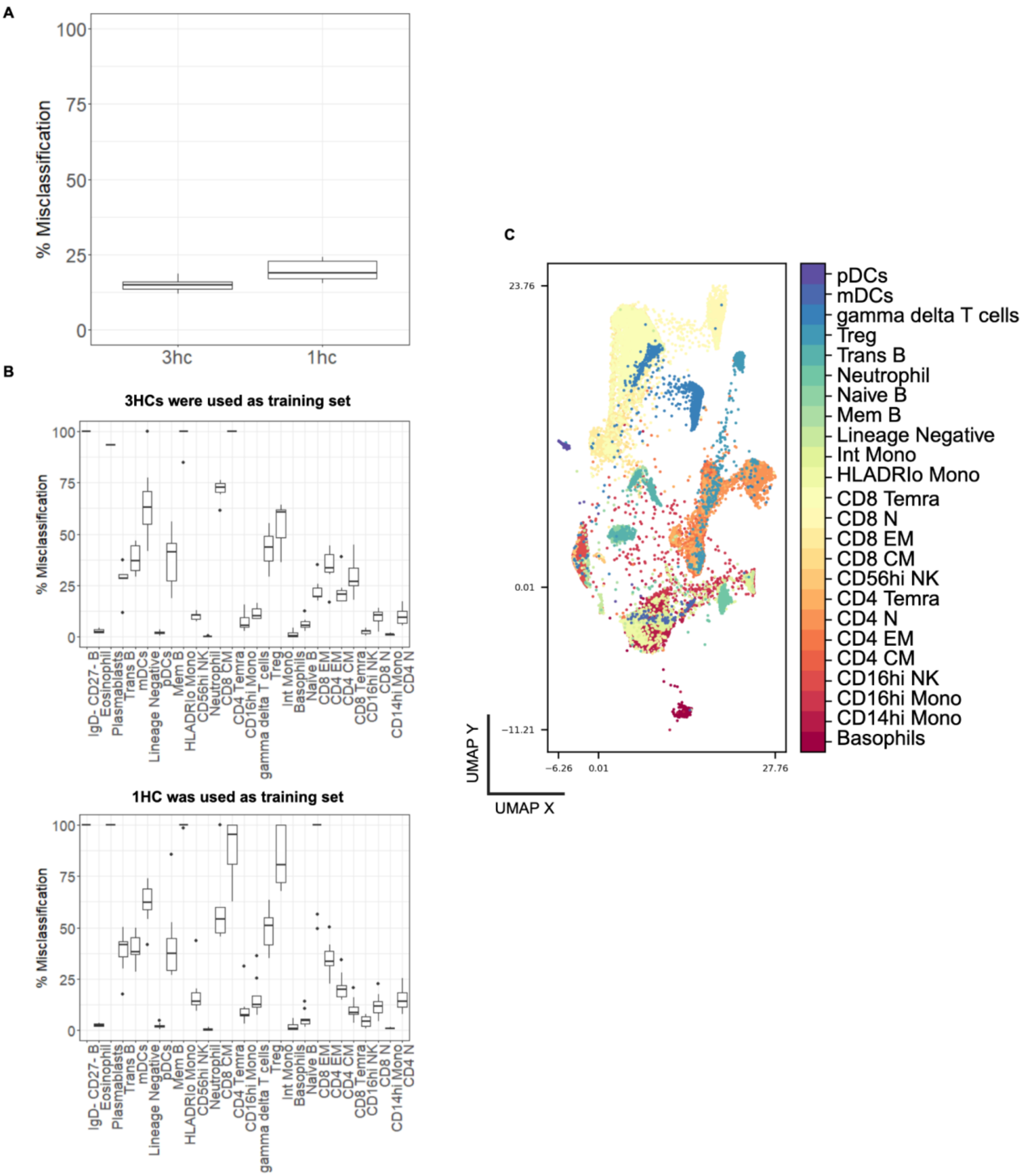

Supplementary Figure 8. Discrepancies between the underlying data topology and the ground truth labels could negatively affect the performance of the label transfer pipeline. A. Randomly chosen one and three (out of ten) healthy control (hc) PBMCs samples, characterized with the ~30 marker CYTOF panel, were used as

training sets and the rest of samples were used as the test set. **B.** The primary source of misclassification arises from the more heterogeneous nature of cell populations than initially identified with the established expert-defined manual gating strategy. For instance, the gamma delta T cells population on panel **C** is actually distributed between the two clusters, as data topology suggests.

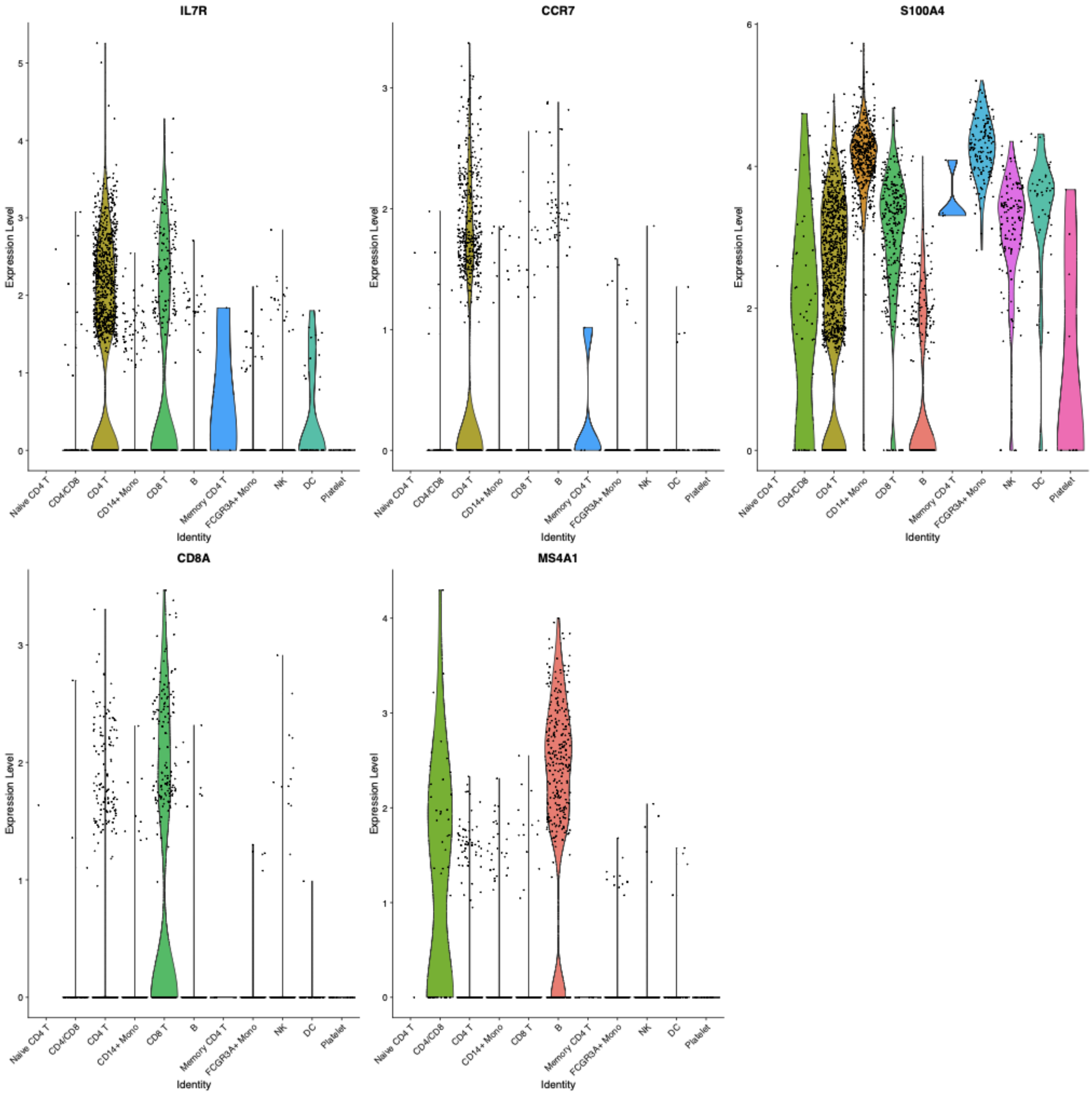

Supplementary Figure 9. The expression of B and T marker genes in cell subsets identified within the PBMCs dataset. MS4A1 is a B cell specific marker, the rest are CD4 and CD8 T cell specific markers. The group of cells that was misclassified by APP is here labeled "CD4/CD8".

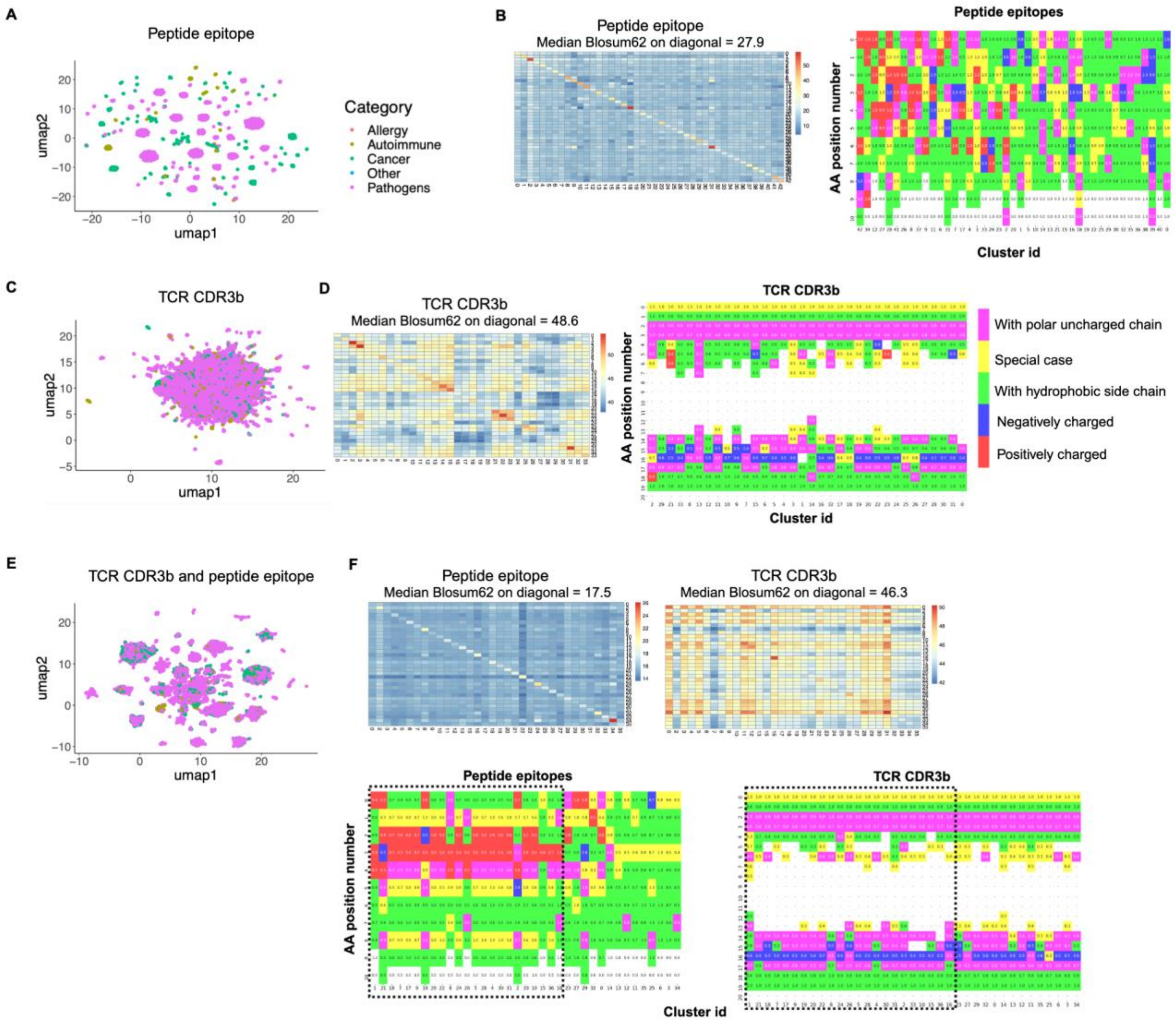

Supplementary Figure 10. Sequence similarity and amino acid R group properties analysis of ESM embeddings generated for peptide epitope sequences (A), TCR CDR3b sequences (C), and the concatenation of both TCR and peptide embeddings (E). Amino acids at a specific position within a designated cluster were categorized according to their R group properties (as indicated in the inserted legend). The most prevalent property within each group is presented, along with its corresponding probability (**B**,**D**,**F**).

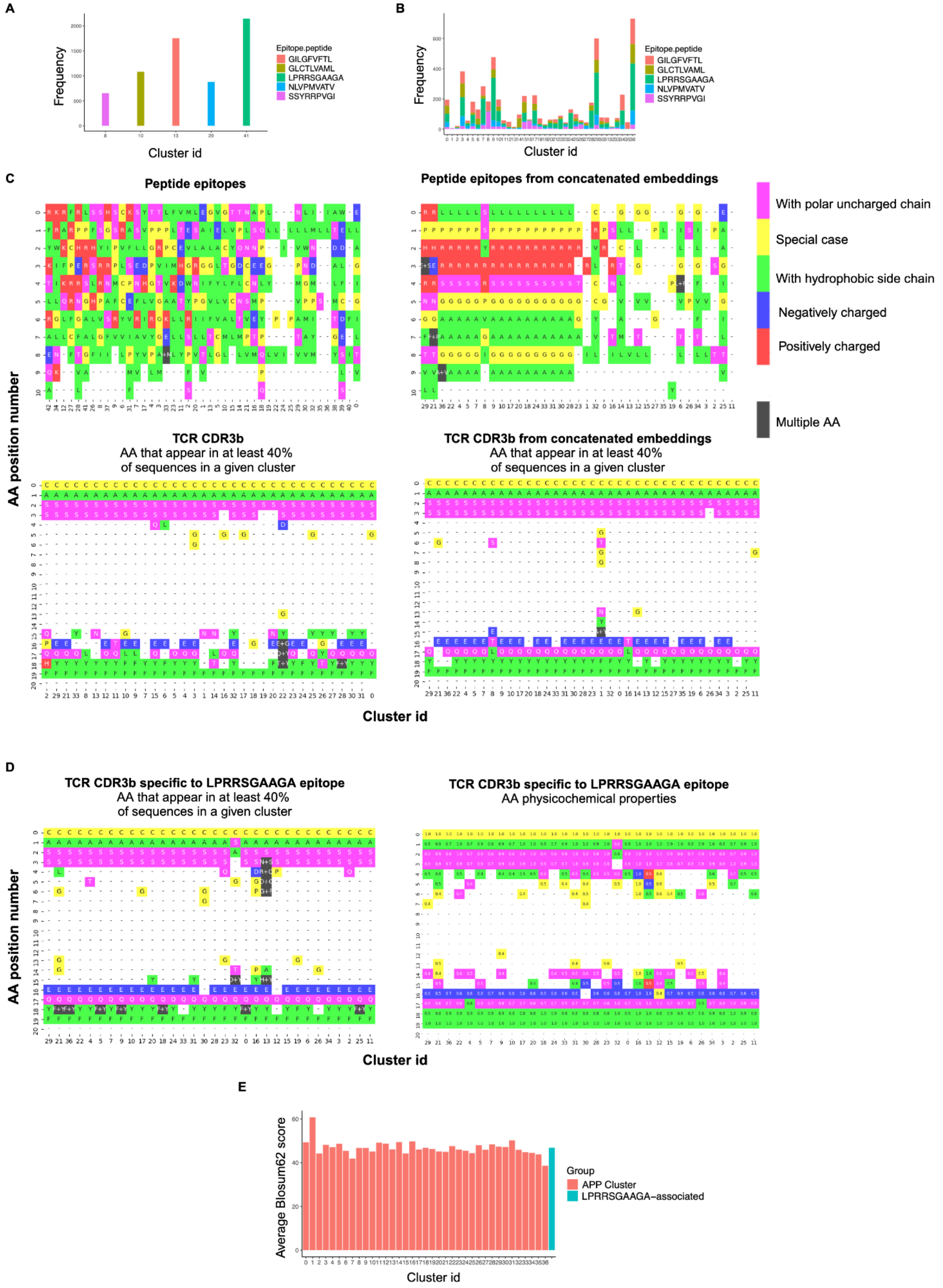
A
Frequency
Cluster id
Epitope.peptide
GILGFVFTL
GLCTLVAML
LPRRSGAAGA
NLVPMVATV
SSYRRPVGI
B
Frequency
Cluster id
Epitope.peptide
GILGFVFTL
GLCTLVAML
LPRRSGAAGA
NLVPMVATV
SSYRRPVGI
C
Peptide epitopes
Peptide epitopes from concatenated embeddings
With polar uncharged chain
Special case
With hydrophobic side chain
Negatively charged
Positively charged
Multiple AA
AA position number
TCR CDR3b
AA that appear in at least 40%
of sequences in a given cluster
TCR CDR3b from concatenated embeddings
AA that appear in at least 40%
of sequences in a given cluster
Cluster id
D
TCR CDR3b specific to LPRRSGAAGA epitope
AA that appear in at least 40%
of sequences in a given cluster
TCR CDR3b specific to LPRRSGAAGA epitope
AA physicochemical properties
AA position number
Cluster id
E
Average Blosum62 score
Cluster id
Group
APP Cluster
LPRRSGAAGA–associated

Supplementary Figure 11. No common binding motif is identified on TCR CDR3b, even when considering similar peptide epitopes. The distribution of the five most common peptides (in the database utilized for this study) appears highly cluster-specific in the context of single-class embeddings (**A**). However, when using concatenated embeddings (CDR3b and peptide), these five peptides are dispersed across multiple clusters (**B**). **C.** No discernible binding motifs appear in the TCR CDR3b, whether analyzed individually (left side) or as concatenated embeddings (right side). Even when focusing exclusively on CDR3b sequences specific to the LPRRSGAAGA peptide (**D**), these sequences exhibit no notable increase in sequence similarity (**E**), as approximated by the Blosum62 score, when compared to the CDR3b sequences clustered together in the concatenated embeddings data presented in Figure 6.

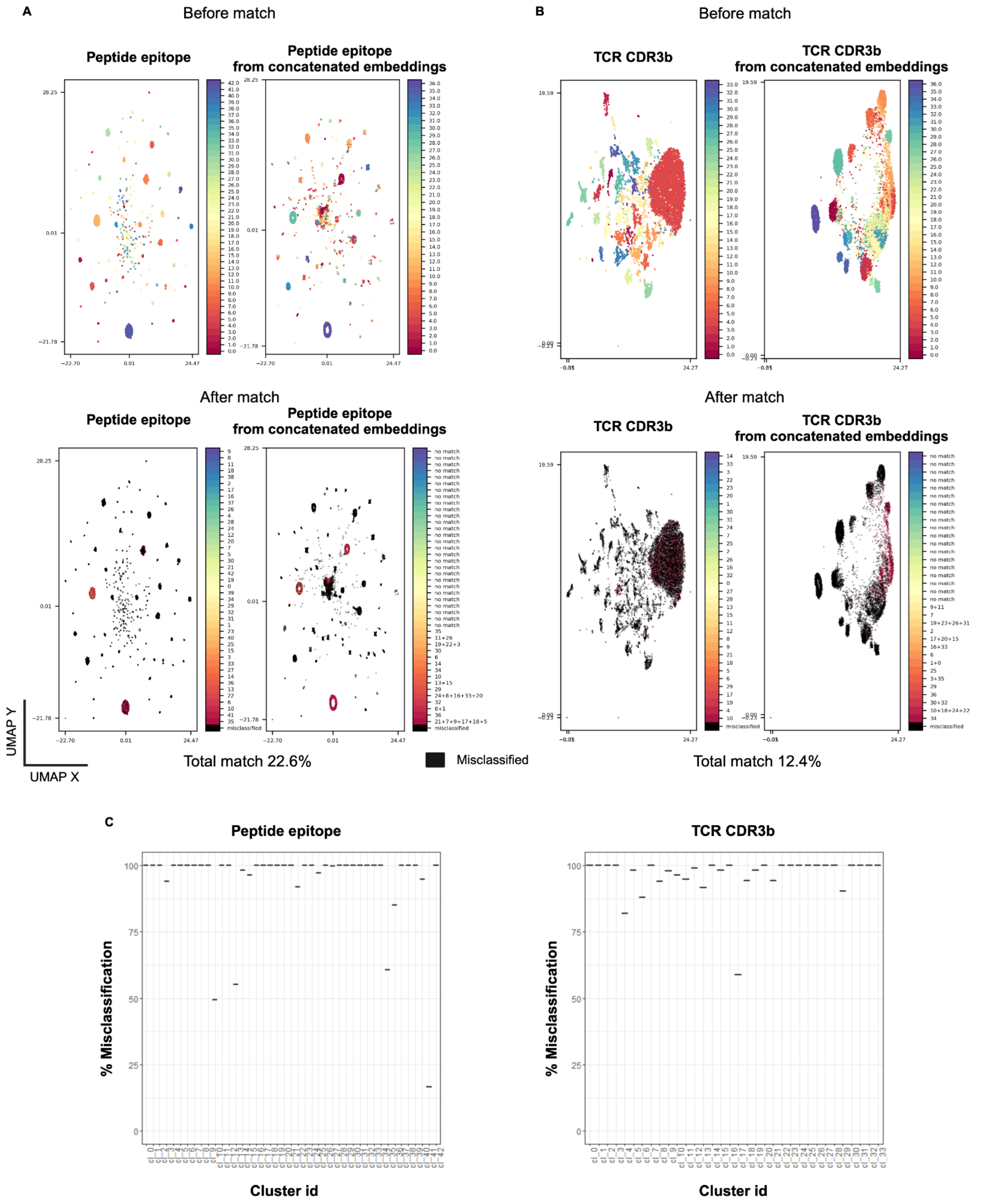

Supplementary Figure 12. Concatenation of TCR CDR3b and peptide embeddings results in distinct similarity criteria captured by the ESM model. The automated

label transfer pipeline was employed to align clusters obtained for the same class of sequences (peptides (**A**) and TCR CDR3b (**B**)) generated from the single class embeddings and concatenated embeddings. The label transfer pipeline was executed on 30 PCs, using the APP cluster labels generated for the single class embeddings as the training set. On the top row of panels A and B, cluster IDs are shown before the cluster alignment, and thus the same color may represent two unrelated clusters on the left and right UMAP plots for each class. On the bottom row of panels A and B, cluster IDs are shown after the match/cluster alignment, and thus the same color represents aligned clusters within the same sequence class. Non-matched clusters are shown in black. **C.** Per-cluster-ID misclassification as estimated by the label transfer pipeline.

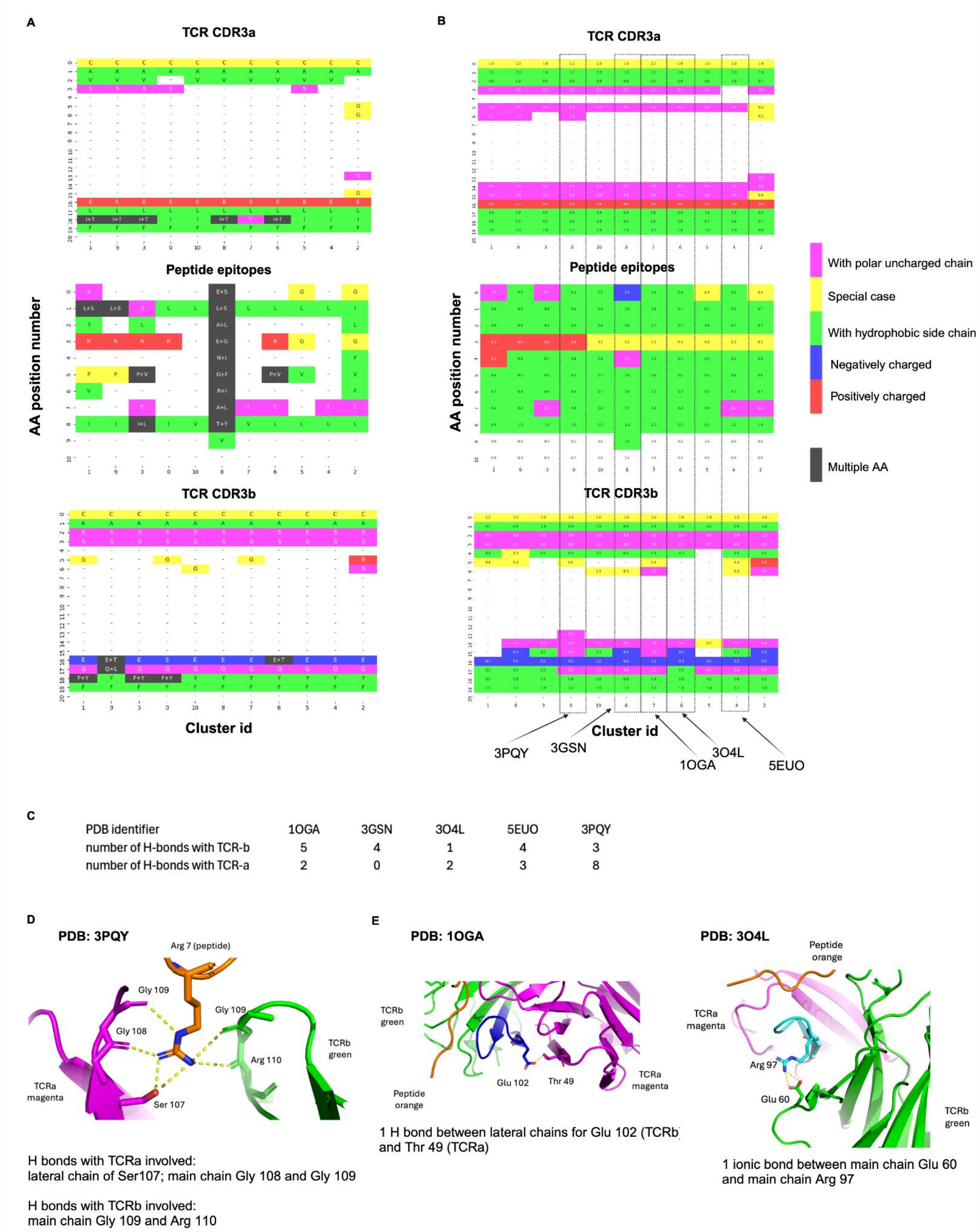

Supplementary Figure 13. The TCR CDR3b sequences exhibit an enrichment of negatively charged amino acids, while TCR CDR3a sequences are characterized

by an enrichment of positively charged amino acids. A. Amino acids that appear with more than 30 percent probability in a given position and in a given cluster are color-coded based on their R group property. These physicochemical maps are constructed for the 4000 CDR3a-CDR3b-peptide data set presented on the right side of Figure 6. **B.** Amino acids at a specific position within a designated cluster were sorted into the corresponding R group property, as specified in the legend. The most dominant property within each group is displayed, along with its associated probability. The results presented focus on R group property classes that exhibit a prevalence of more than 30 percent at a given position within a designated cluster. Analysis of these properties for the peptides showed important hydrophobic enrichment in all clusters (middle panel right, green color). For five independent clusters, we found in the PDB five TCR-pMHC crystal structures and analyzed them. **C.** All showed that the antigen directly interacted with both, alpha- and beta- TCR units. Unexpectedly for such hydrophobic peptides was the presence of H-bonds, noticed for all of them (from 2 to 8 H-bonds) and involving both TCRab. In the center of peptides, we noticed either the presence of one positively charged amino acid (often Arg), or the presence of one Gly. **D.** Structure analysis of PDB_3PQY showed that Arg7 has its 3-nitrogens from the lateral chain involved in the formation of H-bonds with TCRa (4 H-bonds) and TCRb (2 H-bonds). We suggest that through the formation of all the H-bonds the peptide orients itself between the TCRs sub-units and complement the stable 3D surface of the TCRs. Such H-bonds also participate in the stability of the [peptide-TCRs] complex and the presence of Gly (B, middle panel right called "Peptide epitopes", yellow color) allows partial bending of the peptide, when necessary. **E.** We noticed for all CDR3a and CDR3b the respective presence of conserved Lys and Glu (A,B). Analysis of the previous 5 structures showed that these amino acids don’t interact directly with the peptide, neither between themselves. They form H-bonds or ionic bonds between the two TCRs and through this mechanism participate in the pairing of the two subunits (see 1 H-bond between the conserved Glu102 (CDR3b) and Thr49 from TCRa; the ionic bonds bellow between Arg97 (CDR3a) and Glu60 (TCRb).
