# Supplementary material for: Lifting the curse from high-dimensional data: automated projection pursuit clustering for a variety of biological data modalities
Source: Gigascience. 2025 May 29;14:giaf052. doi: 10.1093/gigascience/giaf052 (PMC12121483; doi:10.1093/gigascience/giaf052)
Supplement: giaf052_GIGA-D-24-00440_Revision_1 [file giaf052_giga-d-24-00440_revision_1.pdf]

GigaScience

Lifting the curse from high-dimensional data: Automated projection pursuit clustering for the variety of biological data modalities

--Manuscript Draft--

**Manuscript Number:**	GIGA-D-24-00440R1
**Full Title:**	Lifting the curse from high-dimensional data: Automated projection pursuit clustering for the variety of biological data modalities
**Article Type:**	Research
**Funding Information:**	
**Abstract:**	Unsupervised clustering is a powerful machine-learning technique widely used to analyze high-dimensional biological data. It plays a crucial role in uncovering patterns, structure, and inherent relationships within complex datasets without relying on predefined labels. In the context of biology, high-dimensional data may include transcriptomics, proteomics, and a variety of single-cell omics data. Most existing clustering algorithms operate directly in the high-dimensional space, and their performance may be negatively affected by the phenomenon known as the curse of dimensionality. Here, we show an alternative clustering approach that alleviates the curse by sequentially projecting high-dimensional data into a low-dimensional representation. We validated the effectiveness of our approach, named APP, across various biological data modalities, including flow and mass cytometry data, scRNA-seq, multiplex imaging data, and T-cell receptor repertoire data. APP efficiently recapitulated experimentally validated cell-type definitions and revealed new biologically meaningful patterns.
**Corresponding Author:**	Darya Orlova<br>Cell Signaling Technology Inc<br>Danvers, UNITED STATES
**Corresponding Author Secondary Information:**	
**Corresponding Author's Institution:**	Cell Signaling Technology Inc
**Corresponding Author's Secondary Institution:**	
**First Author:**	Darya Orlova
**First Author Secondary Information:**	
**Order of Authors:**	Darya Orlova
	Claire Simpson
	Evgeniy Tabatsky
	Zainab Rahil
	Devon Eddins
	Sasha Tkachev
	Florian Georgescauld
	Derek Papalegis
	Martin Culka
	Tyler Levy
	Ivan Gregoretti
	Connor Meehan
	Chiara Schiller

Powered by Editorial Manager® and ProduXion Manager® from Aries Systems Corporation

	Kresimir Bestak
	Denis Schapiro
	Andrei Chernyshev
	Guenther Walther
	Eliver Ghosn
**Order of Authors Secondary Information:**	
**Response to Reviewers:**	Dear Editorial Board,<br>Below are our responses to the reviewers' comments. We sincerely appreciate the reviewers' thoughtful and thorough evaluation. We have addressed all concerns to the best of our ability and look forward to your feedback.<br>For your convenience, we have highlighted the changes in the revised manuscript in blue.<br><br><br>Reviewer #1:<br><br>Comment: The authors reference the 'Curse of Dimensionality' in both the title and introduction but do not provide a detailed explanation of the issue. A more thorough discussion of this problem would help readers better understand how the proposed approach addresses and mitigates it.<br><br>Response: We have added the explanation to the manuscript: “The well-known phrase "the curse of dimensionality" was coined by Richard Bellman [Morgenstern, D., & Bellman, R. 1962] and describes challenges that arise when working with high-dimensional data. A key issue is that the amount of data needed to obtain reliable results grows exponentially with the number of dimensions. Data analysis often depends on identifying regions where data points form meaningful clusters, but in high-dimensional spaces, such structures become sparse, making effective analysis increasingly difficult.”<br><br>Comment: The presented pipeline is based on the custom procedure for 2d clustering/decision boundary construction. This procedure is described to some extent, but the underlying logic is could be explained in more details. For now, it seems counter-intuitive that the same projections should be analyzed twice (I mean, different separating boundaries are constructed for x-y and y-x plots).<br><br>Response: We have added the explanation to the manuscript: “In our algorithm, the same projections are analyzed twice to determine a 2D cluster boundary in (x, y)-space: first to identify the x-projection of the boundary and then to determine its y-projection.” We also added a clarification to the Supplementary Figure 1 legend: “Supplementary Figure 1. Example of an optimal decision boundary search for one of the 2D data projections. The decision boundary begins at the left edge and ends at the right edge of a 2D projection. However, in xy vs yx orientations, the left and right edges differ, leading to distinct decision boundaries. Therefore, xy and yx projections should be analyzed independently.”<br><br>Comment: Additionally, the "initial distribution density H" is not defined.<br><br>Response: We have appended the explanation “—that is, the initial distribution density H” to the end of the phrase: “Otherwise, for each 2D (x,y) projection mapped onto a unit square (side length of 1), we build the Gaussian-smoothed histogram H(x,y) of the data points.” The revised sentence in the manuscript now reads: “Otherwise, for each 2D (x,y) projection mapped onto a unit square (side length of 1), we build the Gaussian-smoothed histogram H(x,y) of the data points— that is, the initial distribution density H.”<br><br>Comment: Overall, there seem to be a lot of existing algorithms for construction of optimal separating boundary or even automatic clustering of data in 2D space. It is unclear what advantages and disadvantages they have compared to the procedure developed by the authors (probably higher computational cost?).

Powered by Editorial Manager® and ProduXion Manager® from Aries Systems Corporation

Response: Most existing clustering algorithms operate in high-dimensional space rather than in 2D, making them vulnerable to the curse of dimensionality. Previous implementations of projection pursuit clustering algorithms sequentially operate on 2D projections; however, as discussed in the Introduction of our manuscript, their criteria for selecting the most informative low-dimensional projections was not optimized for identifying decision boundaries with the lowest data density between clusters-a crucial aspect of cell phenotyping. Instead, previous implementations of the projection pursuit algorithm primarily focused on identifying projections with "high" information content, such as those that deviate from normality or uniformity.
We have now emphasized this point in the manuscript with the following text: “The concept of exhaustively exploring low-dimensional projections of high-dimensional data has existed for a few decades. Historically, efforts have been made to systematically explore low-dimensional projections, known as the "grand tour" [Cook et al., 1995], or to optimize specific criteria for identifying informative projections, such as those that reveal structure in the data by deviating from normality or uniformity [Friedman et al., 1974]. However, challenges in determining the optimal criterion and the computational complexities associated with processing numerous low-dimensional projections have hindered the widespread adoption of projection pursuit methods for data clustering tasks.”

Comment: The authors tested their approach against three state-of-the art clustering algorithms (HDBSCAN, KMeans and Phenograph), but they were operating in the original high-dimensional space. The possibility of using these algorithms in 2D projections seems obvious and at least should be discussed.

Response: While HDBSCAN, KMeans, and Phenograph clustering can be applied to individual 2D projections, these algorithms do not include a mechanism to systematically traverse the entire set of 2D projections. They lack recursive steps that would score and evaluate each 2D projection to iteratively refine data splits for downstream processing, which is necessary for effectively identifying final clusters in a multidimensional dataset. Thus, the only way to directly compare the final clusters produced by APP with those from HDBSCAN, KMeans, and Phenograph is to run HDBSCAN, KMeans, and Phenograph on the full high-dimensional dataset rather than on individual 2D projections. Since a multidimensional point space can be uniquely represented by its complete set of 2D projections, it follows that comparing APP with modern multidimensional clustering algorithms (e.g., HDBSCAN, KMeans, and Phenograph) within these 2D projections should be sufficient.
We added the following explanation to the manuscript: “Since a multidimensional point space can be uniquely represented by its complete set of 2D projections, it follows that comparing APP with modern multidimensional clustering algorithms (e.g., HDBSCAN, KMeans, and Phenograph) within these 2D projections should be sufficient.”

Comment: In some cases, clusters may be well-separated in 3D space but appear mixed in 2D projections (such as xy, xz, yz). This can occur, especially when there are many clusters, but the situation might be improved through proper rotation of the coordinate system. Could the authors comment on the potential for this improvement? Additionally, an optimal decision boundary should ideally be independent of in-plane rotations, including the interchange of x- and y- axes. Could the authors suggest possible ways to algorithm modification to satisfy this requirement?

Response: We thank the reviewer for this insightful comment. Indeed, in some cases clusters that are well-separated in higher-dimensional space may appear mixed when projected onto certain 2D planes. To address this, proper rotation of the coordinate system may help uncover projections that better reveal separation. While our current approach does not explicitly incorporate rotation optimization, one potential extension could involve employing rotation search methods such as those used in Projection Pursuit to identify projections that maximize separation between clusters.
Regarding the independence of the decision boundary from in-plane rotations and axis interchange, we agree that this is an important consideration. One potential modification would be to apply symmetry operations (e.g., swapping the x- and y-axes) during the decision boundary search and averaging the resulting decision boundaries to enforce rotational invariance. Additionally, incorporating optimization steps that account for rotational symmetry during boundary determination could help ensure robustness.

Powered by Editorial Manager® and ProduXion Manager® from Aries Systems Corporation

We will consider these suggestions for future algorithmic improvements and exploration in subsequent studies. Additionally, we have included the following statement in the Discussion section: “Also, in some cases clusters that are well-separated in higher-dimensional space may appear mixed when projected onto certain 2D planes. To address this, proper rotation of the coordinate system may help uncover projections that better reveal separation. While our current approach does not explicitly incorporate rotation optimization, one potential future extension could involve employing rotation search methods such as those used in projection pursuit to identify projections that maximize separation between clusters.”

Minor points:

Comment: Figure 1C appears overwhelming due to the large number of 2D projection plots, which seem disorganized. The authors might consider grouping the plots for better clarity. Additionally, the text on the plots is too small. Moreover, there seems to be no need to present six projections in 3D space for the identified clusters.

Response: We simplified panel C and updated the Figure 1C legend accordingly. Additionally, we increased the text font size for better readability.

Comment: Equation (1): The text mentions a square root, but the expression is raised to the power of 0.1. Should it be 0.5?

Response: The key point is that Mann's original formula for the total number of two-dimensional bins includes a power of 0.2. Taking the square root of this power results in a power of 0.1 in our Equation (1), ensuring that the calculation is correct.

Comment: The sentence "If the number of cells at the input to the recursion step is less than 2 * min_cluster_size" might be clearer if "the number of cells" is changed to "the number of points."

Response: We have incorporated this suggestion.

Reviewer #2:

Comment: The authors should present the silhouette of the clusters.

Response: We thank the reviewer for this suggestion and have updated the manuscript accordingly. Please refer to Figures 2 and 3, as well as the legend of Figure 4.

Comment: Paper should be revised for minor typos.

Response: We have revised the manuscript accordingly.

Reviewer #3:

Comment: However, some control experiments are not fully explained. For example, the optimization of APP parameters and the limited comparison with other clustering algorithms could be expanded.

Response: APP has only one required user input parameter: the minimum cluster size, which should be set based on the smallest population the user expects to detect in the dataset. Reducing this value introduces smaller clusters, as expected, while other APP clustering outcomes remain unchanged. All other parameters, such as Gaussian smoothing width, are optional. We have set default values optimized for the Calinski-Harabasz index based on the datasets presented in the manuscript. These parameters are user-adjustable to allow optimization for other data types, guided by the Calinski-Harabasz index. We have now clarified this more explicitly in the Methods section.
We have also updated the GitHub folder with a more detailed parameter description.
We now include comparisons to additional commonly cited clustering methods in flow cytometry, beyond PhenoGraph: flowSOM (1,310 Google Scholar citations since 2021) and SPADE (272 Google Scholar citations since 2021). Please refer to the updated

Powered by Editorial Manager® and ProduXion Manager® from Aries Systems Corporation

Figure 2.

Comment: Additionally, while ground-truth validation is used for some datasets, others rely on expert evaluations, which can introduce bias.

Response: Unfortunately, the current state of the art lacks ground-truth validation for assessing and confirming that identified populations are functionally distinct. In most cases, expert evaluation serves as the “gold standard.” We acknowledge that this introduces bias and have explicitly discussed it in the Conclusions section of the manuscript. In this study, we introduced a method to generate functionally validated ground truth; however, we also recognize that this is a resource-intensive endeavor, making it impractical to expect it to be routinely performed for every new experiment.
“In many biological real-world datasets, the availability of a clear "ground truth" can be challenging. As illustrated in the examples presented, reliance on domain experts' knowledge-driven clustering or clustering done with widely-adopted approaches serves as a substitute for ground truth. While expert-driven clustering provides a valuable reference point, a more accurate (albeit labor-intensive) method for assessing clustering performance involves conducting functional tests on groups of cells assigned to the same cluster. By observing the functional "purity" and homogeneity of a given cluster compared to other cell clusters in the sample, researchers can achieve a more precise evaluation of the clustering results.”

Comment: The total sample size (six healthy controls and six COVID-19 patient) analyzed in the paper appears small for drawing robust conclusions, especially considering the biological variability in human samples. However, it's quite understandable that the number of samples is limited due to the availability of biological samples and the high cost associated of experimental techniques.

Response: We appreciate the reviewers’ feedback and their understanding regarding the relatively low number of samples, which is due to the limited access to this type of biological material and the relatively high cost of the associated experiments. We agree with the reviewer and have considered the biological variability among COVID-19 patients. For example, the six COVID-19 samples analyzed here were collected from patients with severe disease in the ICU, and we intentionally avoided mixing samples from COVID-19 patients with different disease severity. We recently showed that despite the variability among COVID-19 patients, the patients in ICU with severe disease remain distinguishable from healthy controls using flow cytometry to characterize major immune lineages (see Eddins et al., Blood Adv. 2023).
Nonetheless, while six samples per group may be relatively low for deriving strong biological conclusions (due to potential sample-to-sample heterogeneity, and we have adjusted our statement accordingly), from a statistical perspective, a cohort of twelve samples is sufficient to assess the performance of the clustering and cluster-matching pipelines.
We have incorporated the relevant statements in the revised manuscript.

Comment: The study provides strong evidence for APP's ability to identify meaningful clusters and compares it with other clustering methods. However, some conclusions need stronger evidence. For instance, the claim that APP handles sparse data better than other methods is not consistently proven across all datasets.

Response: The overall theoretical conclusion regarding the superiority of projection pursuit over high-dimensional methods in clustering sparse cell populations is derived from the definition of the curse of dimensionality and the well-established fact that, to achieve a given accuracy with typical high-dimensional methods, the sample size must increase exponentially with the number of dimensions. This leads to the logical conclusion that sparse cell populations are particularly susceptible to the effects of the curse of dimensionality. For further illustration, please refer to Supplementary Figure 1 in [Meehan et al., 2019], which we also cited in our manuscript.
To illustrate this in practice, we updated Figure 2 (new panel C) to show the relationship between percent misclassification and the abundance of cell populations in the sample. For "purity," we present this data using samples with functionally validated ground truth labels.

Comment: The statistical methods used in the study, such as the Calinski-Harabasz

Powered by Editorial Manager® and ProduXion Manager® from Aries Systems Corporation

index, are suitable for the types of data analyzed. PCA and Gaussian smoothing are reasonable choices for reducing data complexity and handling noise. However, some choices, like the beta parameter in APP, seem to be based on trial and error and need more justification.

Response: We have included the explanation in the manuscript: "APP has only one required user input parameter: the minimum cluster size, which should be set based on the smallest population the user expects to detect in the dataset. Reducing this value introduces smaller clusters, as expected, while other APP clustering outcomes remain unchanged. All other parameters, such as Gaussian smoothing width, are optional. We have set default values optimized for the Calinski-Harabasz index based on the datasets presented in the manuscript. These parameters are user-adjustable to allow optimization for other data types, guided by the Calinski-Harabasz index."
We have also updated the GitHub folder with a more detailed parameter description.

Comment: Additionally, the study reports accuracy metrics but could include other measures like F1-score to give a more complete picture of performance.

Response: We thank the reviewer for this suggestion and have now included the F1-measure in Figures 2 and 3.

Comment: There are minor grammar issues and repetitive phrases that could be cleaned up. E.g.,
- P.2: While the latter challenge is computational rather than a fundamental scientific limitation, it can be a serious practical hurdle.
- P.3: These insights include evaluating hypotheses regarding the existence of a binding motif between in CDR3b of TCRs that recognize the same peptide ...
- P.3: To gain a deeper understanding ... we initially applied ...
- etc.

Response: We have revised the manuscript accordingly.

Comment: Overall, moderate editing would improve the readability and flow of the manuscript.

Response: We have revised the manuscript accordingly.

Reviewer #4:

Comment: In my opinion, in some parts, the manuscript is almost overwhelming with different applications and datasets that could be explained in more detail. Potentially, Figure 6 could even be split into a separate manuscript to streamline the different applications of APP. However, this can be addressed in the revised version.

Response: There is a clear logic behind how the material is presented: we first demonstrate the method's performance using a dataset with functionally validated ground truth (GFP mice data). Next, we apply the method to a dataset where expert-defined manual gating serves as the "ground truth" (COVID dataset). We then apply the algorithm to single-cell mRNA expression and imaging data, where no predefined ground truth is available. However, clustering outcomes are assessed using domain knowledge from pathology and immunology. For the imaging data, both expression patterns and co-location/spatial distribution are used to evaluate cluster quality. Finally, we apply APP clustering in a fully exploratory mode, where no ground truth is available and no immediate assessment of clustering outcomes is possible due to the lack of pre-existing domain knowledge about how TCR receptor embeddings should cluster with respect to their cognate antigens (TCR data). This approach covers the range of real-world scenarios for using clustering algorithms, whether in the presence or absence of ground truth and domain knowledge.
We have now included this general statement in the Results section of our manuscript.

Comment: I validated that all datasets and code are available. Some descriptions of

Powered by Editorial Manager® and ProduXion Manager® from Aries Systems Corporation

the data could be improved, e.g., more detailed README files, etc. (see below). Perhaps the datasets could also be uploaded to permanent repositories in addition to the GitHub link to ensure permanent accessibility.

Response: Previously unavailable datasets are now shared on Zenodo, accompanied by corresponding readme files. Please refer to the updated version of the Data Availability section in the manuscript.

Introduction

Comment: While the limitations and objectives are clearly defined in the introduction, I believe the authors could expand on alternative, newer methods that people use for clustering. The authors largely describe the historical initial work by Friedman et al., 1982; Huber, 1985, which is 40-50 years old. For instance, newer clustering algorithms might be relevant to include, along with mentioning their limitations. For example, the authors benchmark APP with algorithms such as HDBSCAN and K-means, but these are not introduced. Additionally, mentioning graph-based clustering and dimensionality reduction techniques such as UMAP and t-SNE, and how they address challenges in high-dimensional clustering and have become standard for scRNA-seq data analysis, might be helpful.

Response: We have incorporated these suggestions and revised the Introduction accordingly.

Comment: Since much of the manuscript is about the validation of APP with real-world data, the authors may consider including a bit more information on why they chose these datasets and the different modalities (e.g., flow cytometry, scRNA-seq, multiplex imaging). I appreciate the diversity of the data, but expanding a little on the rationale would be helpful.

Response: This is a very useful suggestion; thank you. We have now included the rationale for selecting the datasets at the beginning of the Results section: “To encompass the range of real-world scenarios for clustering algorithm usage, both with and without ground truth or domain knowledge, we first demonstrate the method’s performance using a dataset with functionally validated ground truth. We then apply the method to a dataset where expert-defined manual gating serves as the ground truth. Next, we test the algorithm on single-cell mRNA expression data and imaging data, which lack predefined ground truth. However, cluster evaluation is enabled using domain knowledge from pathology and immunology, leveraging expression patterns and spatial distribution. Finally, we apply APP clustering in a fully exploratory mode, where no ground truth or pre-existing domain knowledge is available to assess how TCR receptor embeddings cluster with respect to their cognate antigens.”

Comment: Additionally, the authors may clarify the novelty or major advantage of the APP clustering tool. For instance, the authors mention that APP is an alternative clustering approach, but they could perhaps elaborate on its major strengths or novelty in the last paragraph.

Response: We thank the reviewer for this suggestion. In the last paragraph of the Introduction, we have now highlighted that the major advantage and novelty of APP clustering lie in its ability to automatically uncover meaningful structures in high-dimensional data while mitigating the curse of dimensionality, a common challenge in high-dimensional clustering. Unlike traditional projection pursuit, where an analyst manually explores projections to identify patterns, APP automates this process by recursively finding low-dimensional projections with the smallest data density between clusters and continuing the analysis until no further splits are detected.

Comment: The last three paragraphs of the introduction discuss the findings of this manuscript. I would suggest shortening this part into a single last paragraph in the introduction.

Response: We followed the recommendation and condensed the last three paragraphs into a single, more concise paragraph.

Minor comments:

Comment: The authors could use a reference instead of a link in the introduction when referring to supervised UMAP.

Response: To our knowledge, there is no peer-reviewed paper specifically dedicated to supervised UMAP. However, an explanation has been provided by the first authors of the original UMAP paper, which can be found here:
https://github.com/lmcinnes/umap/issues/135.
“We have not written a paper on the subject unfortunately -- there really isn't enough material for one. The idea is pretty straightforward (intersecting fuzzy simplicial sets / taking limits) and largely self-evident. The implementation is publicly available, and hopefully takes care of the technical matters of "making it work in practice". I would certainly welcome suggestions for where code comments might be added to make things clearer.”

Comment: It may not be ideal to use "APP" for the name of the software in the context of models of Alzheimer's disease, as most AD researchers associate APP with Amyloid Precursor Protein.

Response: We appreciate the reviewer bringing this up and are aware of the term APP in the neuroscience field. However, we believe that for a broader readership, particularly computational scientists, the term APP is more commonly associated with "Application" (or "app" for short). Therefore, we would like to retain the name APP for our algorithm.
Methods

Comment: Since the authors generated some of their own data and used publicly available datasets, it might be helpful to add a table with an overview of all datasets they used. It took me some time to understand which datasets were generated in this study and which ones were taken from a public database. Perhaps the authors could explain why they decided to include their own data if the main purpose is to benchmark APP against other clustering software.

Response: We thank the reviewer for this suggestion. We have reformatted the Data Availability section into a table to provide the requested information. Currently, there is a lack of datasets with real ground truth, and most datasets, particularly in flow cytometry, imaging, and scRNA-seq, are accompanied by user-defined ground truth based on field-specific best practices for data preprocessing and annotation. Our decision to include our own data was driven by our commitment to adhering to what we believe are the best practices for preprocessing and annotating cell populations. As illustrated in Supplementary Figure 3, generating ground truth cell population annotations is a challenging task.

Comment: For the flow cytometry data, the authors could add the manufacturer, species, and dilution of the antibody.

Response: We have now incorporated this information into the manuscript.

Comment: More information about the antibodies and their dilution would also be beneficial for the multiplex imaging data.

Response: We have added clone names and working concentrations to the relevant Materials and Methods section of the manuscript.

Comment: The CyTOF methods are not properly described. Although the authors refer to previous studies, they should consider providing a bit more detail.

Response: Although the CyTOF data used in our manuscript was previously published and processed elsewhere, and we are only reusing it, we have now added the following description in the relevant methods section: “Whole blood was collected from consenting healthy human donors (N = 10), and peripheral blood mononuclear cells (PBMCs) were isolated and stained with a metal-conjugated 38-parameter mAb panel

Powered by Editorial Manager® and ProduXion Manager® from Aries Systems Corporation

(see Table S1 in [Toghi Eshghi et al., 2019]), enabling the comparison of 28 immune cell subset frequencies [Toghi Eshghi et al., 2019]. Data were acquired using the Helios™ CyTOF® system (Fluidigm, South San Francisco, CA)."

Comment: Why did the authors use Seurat v3? The current version is v5. Perhaps the authors could double-check whether their code is still executable with v5.

Response: The original data analysis, as described in [Zhou et al., 2020], was conducted using Seurat v3. To avoid potential discrepancies due to version differences, we adopted the same version. However, we have now tested Seurat v5 and confirmed that the code remains executable, including cluster assignments. We have included a relevant statement in the manuscript.

Comment: The description of the scRNA-seq analysis is quite brief; however, the coding data is available on GitHub. In case the authors have not done so, it would be helpful to deposit the data in a permanent database such as Zenodo (since the GitHub data could be altered or deleted).

Response: We thank the reviewer for this suggestion. We have reformatted the Data Availability section to provide the requested information.

Comment: All raw data and code appear to be available on GitHub. The authors could perhaps elaborate on the README files in the main folder to provide more detailed instructions and descriptions of the project.

Response: The README in the main folder has been augmented with a more detailed description of the project and repository contents.

Comment: Fig 2:
In Figure 2, the authors compare APP performance to widely used clustering methods in flow cytometry of immune cells from WT and RAG KO mice. The experimental design is very elegant, mixing the population of cells in different proportions to validate individual cell types, but I think this needs to be better explained in the results. Especially since most scientists reading this manuscript might have a bioinformatics background and may not be familiar with the mouse model and the experimental setup.

Response: We thank the reviewer for the positive comments. We have expanded on this experimental approach in the text to include the following explanation: Mice lacking the RAG1 gene (i.e., RAG-KO) are deficient in immune cells known as B and T lymphocytes, while still developing all other major immune lineages, including myeloid cells and NK cells. Since the wild-type (WT) mice, expressing the RAG1 gene can develop both B and T lymphocytes, we intentionally mixed WT cells from a GFP+ mouse, which expresses green fluorescent protein on their lymphocytes, with cells from the RAG-KO mouse, which does not contain lymphocytes (i.e., WT-GFP mixed with RAG-KO). We used this experimental approach to define a biological and technical ground truth. For example, any B and T cells identified by our new APP pipeline should express the green (GFP) protein since they can only come from the WT-GFP mice and not from the RAG-KO mice. If the pipeline detects any B and T lymphocytes lacking the green/GFP protein, these events would be considered as "misclassification" by the APP pipeline.

Comment: Also, the clustering algorithms that were used as a comparison have several parameters that can be adjusted. How were those chosen? Did any of them lead to better/worse clustering?

Response: We used the default input parameters recommended in available tutorials (e.g., Seurat: [https://satijalab.org/seurat/articles/pbmc3k_tutorial.html]) for the given data type, as parameter adjustments did not significantly improve clustering. This information has now been included in the relevant methods section.

Comment: Perhaps in a comparison like this, it would be helpful to briefly mention the computational performance of the methods. For instance, how long did each algorithm take to run under which computer configuration?

Response: Although we have now included these statistics for the dataset in Fig. 2 (see panel 2B), we emphasize that achieving robust clustering results is more critical than obtaining rapid but inaccurate outcomes.

Comment: It is not clear to me how they generated the UMAP in Fig 2C. Is this based on the APP clustering? And then were the labels transferred from the other clustering algorithms? A bit more explanation is needed.

Response: In response to these questions we now adjusted the Fig. 2D (previously 2C) legend accordingly: "The misclassification for the 50/50 mix is visually represented in black color using the automated label transfer pipeline, as detailed in Supplementary Figure 4. Here, the performance of each of the six clustering algorithms is assessed against the ground truth labels (functionally distinct cell types) used to construct the supervised UMAP embedding."

Comment: Did the authors perform any statistical analysis on the quantified data? The plots look convincing but should be tested using appropriate statistical tests in Fig 2D and E.

Response: In response to this request, we have updated Fig. 2 accordingly. The performance of the APP algorithm in cell population classification is now evaluated using total and per-cell-type population misclassification, F1-measure, and Silhouette coefficient, and is compared to state-of-the-art clustering algorithms.

Comment: Fig 3: The authors should add statistical analysis to Fig 3 A-D and F.

Response: In response to this request, we have updated Fig. 3 accordingly. The performance of the APP algorithm in cell population classification is now evaluated using total misclassification, F1-measure, and Silhouette coefficient and compared to Phenograph. Per-cell-type population misclassification is shown in Supplementary Figure 7.

Comment: The authors convincingly show that the label-transfer pipeline is very accurate when training on one healthy control sample, and that there were more discrepancies with COVID samples. I think the authors could expand a little more on the diverse responses of COVID patients and cellular shifts, which could also include cellular transition states. Also, intragroup variability is likely higher in COVID patients.

Response: We thank the reviewer for raising this important point on biological variables in COVID-19 patients, ranging from asymptomatic to mild, moderate, and severe patients in the ICU. We have revised our manuscript to better describe the patients analyzed here for this method's paper. All six COVID-19 samples analyzed here were collected from patients with severe disease in the ICU, and we intentionally avoided mixing samples from COVID-19 patients with different disease severity, which could add additional biological variables. However, despite analyzing samples from the same disease severity (i.e., ICU patients), we agree with the reviewer that COVID-19 disease induces changes in immune cells, leading to transitional cellular states. These cellular states are dynamic and can vary across patients depending on various factors, including patient demographics, comorbidity, viral load, timing of infection to analysis, etc. Thus, it is expected that samples from COVID-19 patients would show more discrepancy compared to healthy controls. In any event, we recently showed that despite the variability among COVID-19 patients, the patients in ICU with severe disease remain distinguishable from healthy controls using flow cytometry to characterize major immune lineages and cellular states (see Eddins et al., Blood Adv. 2023).
We have incorporated the relevant information into the manuscript.

Comment: The authors suggest that "these findings highlight new mechanisms of SARS-CoV-2-induced immuno-modulation that underlie the COVID-19 immunopathology in hospitalized patients," which might be a slight overstatement. They detect a subpopulation of monocytes, which is interesting. However, there is no data indicating a new mechanism. Toning this statement down would solve the issue.

Response: We agree with the reviewer and have now revised the manuscript to

Powered by Editorial Manager® and ProduXion Manager® from Aries Systems Corporation

highlight that although our new findings might represent a novel mechanism of immune modulation in severe COVID-19, further studies with more patients and different disease states are required to determine whether this is a general mechanism of SARS-CoV-2 infection or a phenotype unique to our small patient cohort.

Comment: Fig 4: The scRNA-seq clustering in Seurat depends on the parameters chosen. Did the authors use default parameters?

Response: Parameters were selected based on the Seurat tutorial (https://satijalab.org/seurat/articles/pbmc3k_tutorial.html), using default settings with a resolution of 0.5. We have added this information to the relevant Methods section.

Comment: The authors claim that naïve and memory CD4 T cells do not show any distinction in clustering in any pairwise dimensions explored by APP but refer to Supplementary Data without specifying where in the Supplementary Data this information can be found. Please add a specific reference to where this information can be found.

Response: Although we believe this supplementary data was uploaded to the journal server during the submission process, we have now uploaded it to Zenodo (https://zenodo.org/records/14874269) and provided a link in the manuscript.

Comment: Did the authors examine CD45RA/PTPRC and CD62L/SELL levels in their PBMC RNA-seq data? These markers are usually used to distinguish naïve and memory CD4 T cells.

Response: We did; however, in this dataset, there was no significant difference in PTPRC and SELL levels among the T cell clusters. We have attached a figure to support this statement.

Comment: For the scRNA-seq dataset, it would be helpful to compare the performance in terms of the time required for clustering. scRNA-seq datasets can have millions of cells, and Seurat may face challenges with conventional analysis. How did APP perform compared to Louvain clustering in terms of speed? Could the authors also test it on a larger dataset?

Response: We thank the reviewer for this suggestion and have incorporated the following information into the manuscript: "To assess the computational complexity of the APP algorithm, which is generally $O(N^2)$, where N is the number of dimensions, we recorded the clustering time for APP versus Seurat v5 using a MacBook Pro with a 2.3 GHz Dual-Core Intel Core i5 and 8 GB memory. For the PBMC dataset (containing 2700 cells), Seurat clustering (resolution = 0.5) took 1–2 seconds in real time. APP, with 10 PCs and a minimum cluster size of 10, took 11m 19.952s real time, 26m 6.587s user time, and 2m 43.988s system time. APP, with 10 PCs and a minimum cluster size of 100, took 2m 16.023s real time, 5m 47.742s user time, and 0m 34.924s system time."

Comment: Perhaps the authors could check whether the clustering algorithm in Seurat was updated between v3 and v5 (the most current version). If not, they could consider adding this information to the methods.

Response: The original data analysis for the 5XFAD dataset, as described in [Zhou et al., 2020], was conducted using Seurat v3. To ensure consistency and avoid potential discrepancies due to version differences, we adopted the same version for scRNAseq data processing within this manuscript. However, we have now tested Seurat v5 and confirmed that the code remains executable, including cluster assignments, with no changes in the clustering results between v3 and v5. We have included a relevant statement in the manuscript.

Comment: Fig 5: What do coordinate X and Y represent? Please add the labels (in Fig 5C-D).

Response: XY Cartesian coordinates represent the position of a tissue sample on a

glass slide. We have added this clarification to the figure legend and updated the labels in Fig. 5D.

Comment: There is almost no figure description for Fig 5. Please expand, e.g., add a scale bar and a description of the heatmap (what does 0-1 in the scale mean? Normalized expression?).

Response: Thank you for pointing this out. We have added the scale bar and updated the figure legend to include details about the heatmap.

Comment: As the authors mentioned, they do not have the information on the ground truth in this dataset, and they also do not compare APP to alternative clustering algorithms for this dataset. What was the rationale for that?

Response: As we stated above, to encompass the range of real-world scenarios for clustering algorithm usage, both with and without ground truth or domain knowledge, we first demonstrate the method's performance using a dataset with functionally validated ground truth. We then apply the method to a dataset where expert-defined manual gating serves as the ground truth. Next, we test the algorithm on single-cell mRNA expression data and imaging data, which lack predefined ground truth. However, cluster evaluation is enabled using domain knowledge from pathology and immunology, leveraging expression patterns and spatial distribution. Finally, we apply APP clustering in a fully exploratory mode, where no ground truth or pre-existing domain knowledge is available to assess how TCR receptor embeddings cluster with respect to their cognate antigens.
We have now included this general statement in the Results section of our manuscript. A comparison to alternative clustering solutions would require the presence of ground truth, which is not available in this case. Moreover, we did not intend to claim the superiority of APP clustering over other solutions for each data modality.

Comment: The authors say: "Pathology and immunology experts independently assessed and confirmed the adequacy of APP clustering in characterizing and distinguishing meaningful cell populations within the slide tissue, particularly in the context of the human squamous lung carcinoma sample." I am not sure how helpful this assessment is, as it is hard to validate and anonymous, and I am also not sure how accurately a pathologist can assess the clustering of a rare cell type that might be misclassified. Would it be helpful if the authors examined the composition of similar cancerous tissues in scRNA-seq/spatial RNA-seq databases and confirmed the expression of the identified marker genes? If this is not possible, the authors could add this as a limitation.

Response: Evaluating the quality and validity of clusters derived from spatially resolved data requires domain expertise in pathology and immunology. This assessment must consider both the expression patterns of panel markers and the spatial distribution and co-location of identified cell phenotypes within the tissue context, in this case, human squamous lung carcinoma.
We do not keep the name of the pathologist who evaluated the clustering outcomes anonymous; in fact, we explicitly acknowledged him in the manuscript's Acknowledgments section. To further clarify his credentials, we have now appended his current affiliation next to his name, and explicitly referenced this in the manuscript text. Additionally, an independent immunological assessment was conducted by Dr. Eliver Ghosn's lab, with Dr. Ghosn and a lab member listed as co-authors of this manuscript.
We appreciate the suggestion to use scRNA-seq or spatially resolved RNA-seq as an orthogonal approach to assess cluster quality. We agree that performing such an analysis on the same sample or a serial section would be ideal. However, we do not have access to the sample used in this study for further analysis. Given the inherent heterogeneity of tissue composition, even within the same patient, extrapolating tissue composition from external patient samples could introduce noise rather than providing a robust validation of clustering accuracy. With this in mind, we have added the following statement to the manuscript: "ScRNA-seq or spatially resolved RNA-seq from the same sample or a serial section could serve as an orthogonal approach to assess cluster quality. However, we did not have further access to the sample used in this

Powered by Editorial Manager® and ProduXion Manager® from Aries Systems Corporation

study for additional analysis."

Comment: Fig 6: Although I find this an interesting approach to partially unravel the decision-making of LLMs, in my opinion, this part of the results is slightly disconnected from the previous findings in Figs 1-5. The data in Fig 6 (and the associated Supplementary Figure) could almost represent a new manuscript. I am not saying it needs to be separated, but it may help to streamline the findings. If the authors want to keep Fig 6, they should benchmark the use of APP with other clustering methods alongside the LLMs to determine whether the performance of APP is comparable to other clustering algorithms.

Response: We thank the reviewer for this suggestion. To help streamline the message we aim to convey in this manuscript, we have added the following statement at the beginning of the Results section: "To encompass the range of real-world scenarios for clustering algorithm usage, both with and without ground truth or domain knowledge, we first demonstrate the method's performance using a dataset with functionally validated ground truth. We then apply the method to a dataset where expert-defined manual gating serves as the ground truth. Next, we test the algorithm on single-cell mRNA expression data and imaging data, which lack predefined ground truth. However, cluster evaluation is enabled using domain knowledge from pathology and immunology, leveraging expression patterns and spatial distribution. Finally, we apply APP clustering in a fully exploratory mode, where no ground truth or pre-existing domain knowledge is available to assess how TCR receptor embeddings cluster with respect to their cognate antigens."

Comment: The authors generate two embeddings for Fig 6, the second embedding being a subset of approximately 4,000 unique TCRs with sequence information for both TCR CDR3α and CDR3β. Could the authors add the rationale for doing this?

Response: We have added the following statement into the relevant method section: " The TCR molecule is a heterodimer that primarily interacts with pMHC through its CDR3a and CDR3b chains. While using paired CDR3a and CDR3b data points is ideal, the high cost of sequencing has led most studies to focus solely on the CDR3b chain. Despite this limitation, the field continues to rely on the CDR3b chain as the primary determinant of TCR specificity, as it is the most highly variable region that contacts pMHC. Accordingly, we have maximized the use of the available dataset by leveraging both CDR3b chain data points and those with paired CDR3a sequences whenever possible."

Comment: Also, similar to previous findings, it is important to know what parameters were used for APP and how robust the results are to changes in these parameters.

Response: APP has only one required user input parameter: the minimum cluster size, which should be set based on the smallest population the user expects to detect in the dataset. Reducing this value introduces smaller clusters, as expected, while other APP clustering outcomes remain unchanged. All other parameters, such as Gaussian smoothing width, are optional. We have set default values optimized for the Calinski-Harabasz index based on the datasets presented in the manuscript. These parameters are user-adjustable to allow optimization for other data types, guided by the Calinski-Harabasz index. We have now clarified this more explicitly in the Methods section. We have also updated the GitHub folder with a more detailed parameter description.

Suppl Figures:

Comment: Suppl Fig 1 shows an example of decision boundary search for 2D projections in APP. Perhaps the figure legend could be expanded, but otherwise, no issues here.

Response: We have expanded the figure legend to include the following information: "The decision boundary begins at the left edge and ends at the right edge of a 2D projection. However, in xy vs yx orientations, the left and right edges differ, leading to distinct decision boundaries. Therefore, xy and yx projections should be analyzed independently."

Powered by Editorial Manager® and ProduXion Manager® from Aries Systems Corporation

	Comment: Suppl Fig 3 shows an example of discrepancies between data topology and clustering decisions in manual gating. Since this is a UMAP, the numbers on the x and y axes are not particularly meaningful and can be removed. Otherwise, no issues here.<br><br>Response: We have removed the numerical labels from the x and y axes.<br><br>Comment: Suppl Fig 4 shows a representation of the label transfer pipeline comparing clustering algorithms. I am not sure why the authors used the word "cartoon." Perhaps they can clarify—this is real data from their clustering, right?<br><br>Response: To clarify this, we have now adjusted the figure legend accordingly: “Schematic representation of the label transfer pipeline application to quantitative comparison of two clustering algorithms decisions made on the same data set. Here, we used a synthetic dataset consisting of a mixture of Gaussian distributions.”<br><br>Comment: Suppl Fig 6 shows a UMAP with misclassification by APP and Phenograph compared to manual gating annotations. The labeling is a bit difficult to read and not always clear to which cluster it refers. Also, the numbers on the x and y axes can be removed.<br><br>Response: We increased the font size of the labels and removed the numbers on the x and y axes.<br><br>Comment: Suppl Fig 10 shows sequence similarity and amino acid group properties in peptide and TCR embeddings. The figure feels a bit busy and overwhelming with data (true for subsequent supplementary figures as well). Perhaps the authors could decide whether all of the data is required to show the sequence similarity, and they should also subdivide the figure into more panels than a-c.<br><br>Response: We have now subdivided the figure into panels (a–f).<br><br>Comment: Suppl Fig 11 shows the absence of common binding motifs in TCR CDR3b sequences for similar epitopes. See comments on Suppl Fig 10.<br><br>Response: We have now subdivided the figure into panels (a–e).<br><br>Comment: Suppl Fig 12 shows cluster alignment differences between single-class and concatenated embeddings for peptides and TCRs. The labels are very small and can be enlarged. Otherwise, no issues here.<br><br>Response: We have increased the font size of the labels.<br><br>Discussion<br><br>Comment: While APP's ability to assign cluster labels is highlighted, the authors could further discuss how the approach to projection is unique compared to modern methods like, for example, Phenograph.<br><br>Response: Please see the third and fourth paragraphs of the Introduction, where we have added some details to bring clarity to this. The main difference is that APP repeatedly restricts focus to a two-dimensional projection of the dataset, other modern clustering methods generally operate in high-dimensional space while using v...
**Additional Information:**	
**Question**	**Response**
Are you submitting this manuscript to a special series or article collection?	No
**Experimental design and statistics**<br><br>Full details of the experimental design and	Yes

Powered by Editorial Manager® and ProduXion Manager® from Aries Systems Corporation

statistical methods used should be given in the Methods section, as detailed in our Minimum Standards Reporting Checklist. Information essential to interpreting the data presented should be made available in the figure legends.<br><br>Have you included all the information requested in your manuscript?	
**Resources**<br><br>A description of all resources used, including antibodies, cell lines, animals and software tools, with enough information to allow them to be uniquely identified, should be included in the Methods section. Authors are strongly encouraged to cite Research Resource Identifiers (RRIDs) for antibodies, model organisms and tools, where possible.<br><br>Have you included the information requested as detailed in our Minimum Standards Reporting Checklist?	Yes
**Availability of data and materials**<br><br>All datasets and code on which the conclusions of the paper rely must be either included in your submission or deposited in publicly available repositories (where available and ethically appropriate), referencing such data using a unique identifier in the references and in the "Availability of Data and Materials" section of your manuscript.<br><br>Have you have met the above requirement as detailed in our Minimum Standards Reporting Checklist?	Yes
GigaScience has policies and guidelines in place for the use of generative AI-writing tools such as ChatGPT. If you have used such writing tools to assist with writing the manuscript this must be	Yes

declared and cited in the text. Authors should not list AI-writing tools and other AI-assisted technologies as an author or co-author and should acknowledge that they are fully responsible for text generated or refined by AI-writing tools.<p><br><br>A summary of use (particularly in the introduction or among methods) needs to be included at the end of the paper, and the outputs should also be included as a supplementary file hosted in GigaDB or other open repositories. Please read our guidelines for more information. <p><br><br>By submitting to GigaScience, you are aware of the journal's AI-writing tools policy, and if you have declared use of such tools below, you have acknowledged this where appropriate in your manuscript and have made a summary of use and outputs available. <p><br><b>AI-assisted writing tools have been used in the preparation of this manuscript?	

Manuscript
Click here to access/download;Manuscript;APP_paper_revised_Apr 3-

Lifting the curse from high-dimensional data: Automated projection pursuit clustering for the variety of biological data modalities

Claire Simpson[1]*, Evgeniy Tabatsky[2]*, Zainab Rahil[3], Devon J. Eddins[4], Sasha Tkachev[1], Florian Georgescauld[1], Derek Papalegis[1], Martin Culka[5], Tyler Levy[1], Ivan Gregoretti[1], Connor Meehan[6], Chiara Schiller[7,8], Kresimir Bestak[7], Denis Schapiro[7,8,9], Andrei Chernyshev[10], Guenther Walther[11], Eliver E. B. Ghosn[4], Darya Orlova[1#]

[1] Cell Signaling Technology, Danvers, Massachusetts, 01915, USA

[2] Independent researcher, Komsomolsk-on-Amur, 681021, Russia

[3] Genentech, South San Francisco, California, 94080, USA

[4] Division of Immunology and Rheumatology, Department of Medicine, Lowance Center for Human Immunology, Emory University School of Medicine, Atlanta, Georgia, 30322, USA

[5] Department of Systems Biology, Columbia University, New York, New York, 10032, USA

[6] Independent researcher, Surrey, British Columbia, V3T 3V4, Canada

[7] Institute for Computational Biomedicine, Heidelberg University, Faculty of Medicine, Heidelberg University Hospital, Heidelberg, 69120, Germany

[8] Translational Spatial Profiling Center (TSPC), Heidelberg, 69120, Germany

[9] Institute of Pathology, Heidelberg University Hospital, Heidelberg, 69120, Germany

[10] Voevodsky Institute of Chemical Kinetics and Combustion SB RAS, Novosibirsk, 630090, Russia

[11] Department of Statistics, Stanford University, Stanford, California, 94305, USA

# To whom correspondence should be addressed: dyorlova@gmail.com

* CS and ET are joint first authors

ORCID iDs: Darya Orlova [0009-0000-7608-6865]; Devon Eddins [0000-0002-7905-8306]; Florian Georgescauld [0000-0003-0530-1884]; Ivan Gregoretti [0000-0001-6965-2494]; Connor

Meehan [0000-0002-7596-2437]; Chiara Schiller [0009-0000-3771-2202]; Kresimir Bestak [0009-0009-8245-9846]; Denis Schapiro [0000-0002-9391-5722]; Andrei Chernyshev [0000-0002-7224-1992]; Guenther Walther [0000-0002-0374-123X]; Eliver Ghosn [0000-0001-7258-906X]

Abstract

Unsupervised clustering is a powerful machine-learning technique widely used to analyze high-dimensional biological data. It plays a crucial role in uncovering patterns, structure, and inherent relationships within complex datasets without relying on predefined labels. In the context of biology, high-dimensional data may include transcriptomics, proteomics, and a variety of single-cell omics data. Most existing clustering algorithms operate directly in the high-dimensional space, and their performance may be negatively affected by the phenomenon known as the curse of dimensionality. Here, we show an alternative clustering approach that alleviates the curse by sequentially projecting high-dimensional data into a low-dimensional representation. We validated the effectiveness of our approach, named APP, across various biological data modalities, including flow and mass cytometry data, scRNA-seq, multiplex imaging data, and T-cell receptor repertoire data. APP efficiently recapitulated experimentally validated cell-type definitions and revealed new biologically meaningful patterns.

Keywords

Curse of dimensionality, clustering, high-dimensional data, projection pursuit, unsupervised machine learning

Introduction

The well-known phrase "the curse of dimensionality" was coined by Richard Bellman [1], who used it to describe the fact that the computational complexity of many numerical methods increases exponentially with the dimension. But there is also a statistical version of the curse of dimensionality, which is also called the

"empty space phenomenon" and which refers to the fact that data become sparse in high dimensions. For example, if data are sampled from a ten-dimensional cube $\{x : |x_i| \leq 1 \text{ for all } i\}$, then only about 1% of the data will fall into the cube $\{x : |x_i| \leq 0.63 \text{ for all } i\}$. As a consequence, density estimators that use local averaging will not work well as most local neighborhoods will be empty. In order to obtain the same number of observations in the neighborhood given by a sub-cube as in the univariate case, the number of observations needs to increase exponentially with the dimension, see for example [2]. This is the reason why for example multivariate density estimation is a notoriously difficult problem.

Modern biological data can be quite complex and high-dimensional, making it challenging to uncover meaningful insights from the data. Clustering is often employed to discover interesting patterns in the data by partitioning it into clusters, where data points within the same cluster are more similar to each other than to those in other clusters. High-dimensional clustering and projection pursuit [3-5] aim to address the problem of discovering patterns in the data. However, they approach the problem from different angles.

High-dimensional clustering, such as HDBSCAN [6], KMeans [7], Phenograph [8], FlowSOM [9], and SPADE [10], aim to group similar data points together based on a similarity measure or fit to a posited generative model directly in the high-dimensional space. For example, PhenoGraph represents the dataset as a mathematical graph built using the k-nearest neighbors of each point, then runs a Louvain community detection algorithm to cluster the graph. While these approaches retain the full information of the original data, they are susceptible to the curse of dimensionality [2,11,12], which can lead to data sparsity and uninformative distance metrics. As a result, traditional clustering methods may struggle to accurately uncover biological patterns [13] (see also Supplementary Tables 1 and 2 in [14]). To address these limitations, dimensionality

reduction techniques (e.g., PCA [15], t-SNE [16], UMAP [17] are often employed before clustering to enhance interpretability and mitigate high-dimensional challenges. However, these methods come with trade-offs, as they may distort global structures or obscure biologically relevant variations.

An alternative approach is projection pursuit, which seeks lower-dimensional projections that reveal meaningful structures while preserving key data characteristics. This is motivated by the fact that in many situations the relevant information (such as cluster relationships) is contained in a lower dimensional subspace [2], with the remaining dimensions being uninformative. Projection pursuit involves finding projections that maximize some criterion or interesting property. Once an interesting set of projections has been found, existing structures (clusters) can be extracted and analyzed separately. Projection pursuit can reveal hidden structures and relationships in the data that might be difficult to detect in the original high-dimensional space due to the curse of dimensionality. However, the choice of criterion will determine the types of patterns that the optimization will search for, and many choices exist. Additionally, identifying the right projection can be computationally intensive, since one needs to explore approximately d^N/N! projections, where d is the data set dimensionality, and N is the dimensionality of the low-dimensional projection. While this challenge is primarily computational rather than a fundamental scientific limitation, it can pose a significant practical obstacle.

The concept of exhaustively exploring low-dimensional projections of high-dimensional data has existed for a few decades. Historically, efforts have been made to systematically explore low-dimensional projections, known as the "grand tour" [18], or to optimize specific criteria for identifying informative projections, such as those that reveal structure in the data by deviating from normality or uniformity [3]. However, challenges in determining the optimal criterion and the computational complexities associated with processing numerous low-dimensional projections have hindered the widespread adoption of projection pursuit methods for data clustering tasks.

We developed Automated Projection Pursuit (APP) clustering, combining projection pursuit principles [3-5] with clustering to uncover structures in high-dimensional data.

Unlike traditional projection pursuit, APP automates the search for low-dimensional projections with minimal density between clusters, recursively refining clusters until no further splits are detected. This enhances reproducibility and mitigates the curse of dimensionality. APP was applied to diverse data modalities, including flow/mass cytometry, scRNA-seq, multiplex imaging, and TCR repertoire data, accurately recapitulating known cell types and providing new biological insights. Notably, it enabled the assessment of a potential binding motif in the CDR3b region of TCRs and a charged amino acid pattern stabilizing CDR3a–CDR3b interactions. To evaluate APP's performance against other high-dimensional clustering methods, we applied it to biological data with known ground truth and implemented a label transfer pipeline using supervised UMAP [19]. This approach facilitated quantitative comparisons while preserving data topology. As an example, our analysis uncovered a novel myeloid cell population enriched in hospitalized COVID-19 patients.

Materials and methods

Data Overview

Flow cytometry data

Dataset with a functionally validated ground truth

Mice lacking the RAG1 gene (i.e., RAG-KO) are deficient in immune cells known as B and T lymphocytes, while still developing all other major immune lineages, including myeloid cells and NK cells. Since the wild-type (WT) mice, expressing the RAG1 gene, can develop both B and T lymphocytes, we intentionally mixed WT cells from a GFP+ mouse, which expresses green fluorescent protein on lymphocytes, with cells from the RAG-KO mouse, which does not contain lymphocytes (i.e., WT-GFP mixed with RAG-KO). We used this experimental approach to define a biological and technical ground truth. For example, any B and T cells identified by our new APP pipeline should express the green (GFP) protein since they can only come from the WT-GFP mice and not from the RAG-KO mice. If the pipeline detects any B and T lymphocytes lacking the

green/GFP protein, these events would be considered as “misclassification” by the APP pipeline.

COVID dataset

Whole blood from consenting COVID-19 patients and healthy donors were collected as part of our previous study (see [20] for Emory Institutional Review Board (IRB) protocol numbers) by standard venipuncture, then samples were processed as previously described [20]. Peripheral blood mononuclear cells (PBMCs) were isolated from whole blood after serum collection using the EasySep™ Direct Human PBMC Isolation Kit (StemCell Technologies) following the manufacturer’s instructions. We then performed either a custom monocyte-enrichment procedure (via negative selection) utilizing Mojosort™ anti-PE Nanobeads (BioLegend) and PBMCs stained with CD3ε::PE (clone: UCHT1), CD19::PE (SJ25C1), CD56::PE (5.1H11), and CD57::PE (HNK-1; all from BioLegend) for COVID-19 samples or the EasySep™ Human Monocyte Enrichment Kit without CD16 Depletion kit (StemCell Technologies) for health donor samples.

An aliquot of monocyte-enriched PBMCs ($<10^7$ total) was resuspended in fluorescence-activated cell sorter (FACS) buffer in 5 mL FACS tubes and pre-incubated with Human TruStain FcX™ (BioLegend). The 28-color extracellular staining master mix included: CD86::BB515 (clone: FUN-1; titration 1:10), CD45-RA::BB630-P2 (HI100; 1:160), CD19::BB660-P2 (HIB19; 1:50), CD45::BB700 (HI30; 1:640), CD4::BB755-P (RPA-T4; 1:100), HLA-DR::BB790-P (G46-6; 1:50), CD1c::BV480 (746677; 1:20), HLA-ABC::BV650 (G46-2.6; 1:320), CD11b::BV750 (ICRF44; 1:20), CD56::BUV563 (NCAM16.2; 1:160), CD123::BUV661 (9F5; 1:40), CD14::BUV737 (M5E2; 1:50), CD8α::BUV805 (SK1; 1:80) from BD Biosciences; CD163::BV421 (GHI/61; 1:50), CD16::BV570 (3GB; 1:100), CD169::BV605 (7-239; 1:100), CD141::BV711 (1A4; 1:100), CD197::BV785 (G043H7; 1:50), XCR1::PE (S15046E; 1:50), CD206::PE-Dazzle594 (15-2; 1:200), CD10::PE-Cy5.5 (HI10a; 1:20), CD3ε::PE-Cy5 (UCHT1; 1:100), CD172a/b::PE-Cy7 (SE5A5; 1:200), CD66b::APC (QA17A51; 1:200), CD11c::AF700 (Bu15; 1:100), and CD32::APC-Fire750 (FUN-2; 1:50) from BioLegend; and the amine-reactive viability stain GhostDye™ UV450 (1:100) from Tonbo Biosciences. Fluorophores marked with -P(2) denote prototype reagents that are

custom conjugations from BD Biosciences and purified CD10 was conjugated in-house using the Lightning-Link® PE-Cy5.5 Antibody Labeling Kit (cat no. 761-0010) from Novus Biologicals (Abbreviations: AF: AlexaFluor, APC: Allophycocyanin, BB: Brilliant Blue, BUV: Brilliant Ultraviolet, BV: Brilliant Violet, FITC: Fluorescein isothiocyanate, PE: Phycoerythrin). Titrations of all reagents were determined empirically for each lot independently. The staining master mix was prepared 2X in BD Horizon™ Brilliant Stain Buffer (BD Biosciences) and added 1:1 to cells. After staining, cells were fixed with 4% paraformaldehyde, then washed with FACS buffer, and resuspended in 200-1000 µL FACS buffer for acquisition using BD FACSDiva™ Software on the Emory Pediatric/Winship Flow Cytometry Core BD FACSymphony™ A5.

All six COVID-19 samples analyzed in this study were collected from patients with severe disease in the intensive care unit (ICU). To minimize biological variability, we intentionally avoided mixing samples from COVID-19 patients with different disease severities.

Mass cytometry (CyTOF) data

Whole blood was collected from consenting healthy human donors (N = 10), and peripheral blood mononuclear cells (PBMCs) were isolated and stained with a metal-conjugated 38-parameter mAb panel (see Table S1 in [21]), enabling the comparison of 28 immune cell subset frequencies [21]. Data were acquired using the Helios™ CyTOF® system (Fluidigm, South San Francisco, CA).

RNAseq data

PBMC dataset

Publicly available scRNA-seq counts data from 2,700 single peripheral blood mononuclear cells (PBMC) were accessed from 10X Genomics (see Data availability).

Wildtype and 5XFAD mouse model dataset

Single-nucleus RNA-seq counts data for 3 wildtype and 3 5XFAD 7-month-old mouse brains was downloaded from the Gene Expression Omnibus (GEO) database (see Data availability) [22]. Droplet-based 5' end massively parallel single-cell RNA sequencing

had been performed on the samples, and data processing was done using the Cell Ranger Single-Cell Software Suite from 10x Genomics by the originators of the data.

Multiplex imaging data

CD11c (D3V1E), SIRPα (D6I3M), CD163 (D6U1J), CD206/MRC1 (E2L9N), CD68 (D4B9C), CD45 (D9M8I), HLA-DRA (E9R2Q) and Pan-Keratin (C11) antibodies were conjugated to oligonucleotides (oligos) and then validated in the SignalStar Multiplex IHC assay to assess the myeloid compartment of the tumor microenvironment. Paraffin-embedded human squamous cell carcinoma tissue was tested using 8-plex panel Pan-Keratin (C11) & CO-0003-488 SignalStarTM Oligo-Antibody Pair #63566 (0.25 ug/ml), CD68 (D4B9C) & CO-0007-594 SignalStarTM Oligo-Antibody Pair #77318 (0.5 ug/ml), CD206/MRC1 (E2L9N) & CO- 0035-488 SignalStarTM Oligo-Antibody Pair #99626 (0.25 ug/ml), CD163 (D6U1J) & CO-0022-750 SignalStarTM Oligo-Antibody Pair #71043 (0.7 ug/ml), SIRPα/SHPS1 (D6I3M) & CO-0034-647 SignalStarTM Oligo-Antibody Pair #80150 (0.625 ug/ml), CD45 (Intracellular Domain) (D9M8I) & CO-0013-647 SignalStarTM Oligo-Antibody Pair #32740 (0.1 ug/ml), CD11c (D3V1E) & CO-0017-594 SignalStarTM Oligo- Antibody Pair #85384 (2.0 ug/ml), HLA-DRA (E9R2Q) & CO-0023- 750 SignalStarTM Oligo-Antibody Pair #58446 (0.05 ug/ml) using SignalStarTM mIHC technology.

All 8 primary antibodies are applied at once in one primary incubation step. A network of complementary oligonucleotides with fluorescent channels 488, 594, 647, 750 nm amplify the signal of up to 4 oligo-conjugated antibodies in the first round of imaging, followed by removal and amplification of 4 additional antibodies in the second round of imaging. Images were acquired on the PhenoImager HT (Akoya Biosciences). The antibodies were quantitatively validated in the SignalStar assay to ensure maximum fluorescent signal with minimal background, and compared against the chromogenic gold standard.

TCR repertoire data

The TCR repertoire data utilized in this study were obtained from the McPAS-TCR database [23]. McPAS-TCR is a manually curated resource containing human and mouse TCR sequences associated with various pathologies and their cognate antigens. We downloaded the September 10, 2022 version of McPAS-TCR (latest available), providing over 13,000 TCR CDR3-beta chain and epitope pairs.

The TCR molecule is a heterodimer that primarily interacts with pMHC through its CDR3a and CDR3b chains. While using paired CDR3a and CDR3b data points is ideal, the high cost of sequencing has led most studies to focus solely on the CDR3b chain. Despite this limitation, the field continues to rely on the CDR3b chain as the primary determinant of TCR specificity, as it is the most highly variable region that contacts pMHC. Accordingly, we have maximized the use of the available dataset by leveraging both CDR3b chain data points and those with paired CDR3a sequences whenever possible.

Data analysis

Flow cytometry data

Manual, user-guided analyses were performed using AutoGate [14, 24] and FlowJo™ v10.8 (BD Biosciences).
For assessing the performance of the label transfer pipeline, only healthy control samples were used for training (one or three samples randomly selected from a total of six), as the manual gating strategy was established based on healthy controls. The remaining manually gated healthy control samples, along with all COVID samples, were used as the test set (one sample at a time). This procedure was repeated three times, and representative results are shown.

Mass cytometry (CyTOF) data

Comprehensive conventional manual gating strategy for 38-parameter human immunophenotyping is described at [21].

RNAseq data

Both datasets were processed using Seurat v3 [25]. Counts were log-normalized and scaled, and UMAP reduction and PCA (principal component analysis) were performed. Seurat's default graph-based clustering algorithm was used to identify cell-type clusters [26]. Cell types were annotated by comparing known biomarkers with the markers calculated for each cluster. The PBMC dataset was re-processed using Seurat v5 in order to assess any updates to Seurat's clustering algorithm, and the clustering results were unchanged between versions.

Principal components (10 for the PBMC dataset and 20 for the brain dataset [22]) were extracted from the Seurat objects in order to run the APP clustering procedure (using a minimum cluster size of 150 for both datasets and a minimum cluster size of 10 to further cluster the T cells in the PBMC dataset at a more granular level), which produced new cluster identifications for each cell. New cell annotations were identified by comparing known biomarkers with cluster markers calculated using the new identifications. Differently-matched and non-matched cells were identified and tabulated. Dimensionality reduction plots and heat maps were produced using Seurat, and other visualizations were produced using ggplot.

Multiplex imaging data

To interpret SignalStar data collected in two imaging rounds (Round 1 and Round 2) with the PhenoImager HT (Vectra Polaris) several data pre-processing and processing steps were performed. More specifically, whole slide imaging data collected with PhenoImager HT underwent image stamping and whole section selection in the Phenochart (Akoya Biosciences), with the further spectral unmixing and autofluorescence removal done in the Inform (Akoya Biosciences) to distinguish true signals from background noise and ensure accurate quantification of each fluorophore signal. We then offer two analysis options for downstream data processing: using QuPath or MCMICRO.

QuPath analysis

TIFF components were then exported from the Inform (Akoya Biosciences) into the QuPath [27] software where the TIFF components stitching, image alignment co-registration, and image fusion were sequentially performed. These steps allow simultaneous visualization of multiple markers signals from which were recorded across different cycles or time points.

Nuclear and membrane segmentation were then done using Cellpose QuPath extension [28] on the pre-processed images. Specifically, the 'nuclei' base model of the Cellpose algorithm was employed, with the DAPI nuclear signal from Round 1 serving as its input. Expected diameter of detected nuclei was set to zero to allow for automatic computation by Cellpose. To approximate cell boundaries, a nucleus expansion algorithm implemented in Cellpose was employed, with the cellExpansion parameter set to 5 micrometers. Cell expansion was constrained to 1.5 times the size of the nucleus, controlled by the cellConstrainScale parameter. Additionally, tile size was set to 2048 pixels and the setOverlap parameter that accounts for overlaps between the tiles was set to 100 pixels.

Following segmentation, twenty features per marker were extracted for each single cell and used for the subsequent cellular analysis including cell phenotyping. Specifically, measurements of marker mean, median, maximum, minimum, and standard deviation were calculated for the nucleus, cytoplasm, membrane, and the entire cell.

MCMICRO analysis

The image-processing pipeline MCMICRO [29,30] allows scalable and modular analysis of highly-multiplexed images. The component data .tif files exported with the Inform software after unmixing were pre-processed to be compatible with the pipeline. In detail, the original per channel and tile .tif files were stacked into one ome-tiff file per cycle (Round 1 and Round 2). The signal intensities were normalized across each channel within a cycle by the respective maximum value and converted from float32 to uint16. Further, the metadata was restructured to meet the ome-xml metadata standard. We

combined the described pre-processing steps into a phenoimager2mc staging module (see Availability of source code and requirements) that users can apply in order to analyze their multicyclic PhenoImager data with MCMICRO.

Within MCMICRO, additionally, registration and stitching were performed with ASHLAR (1.18.0) [31] based on the DAPI channels from both cycles (channel 5 and 11 in the stacked image). Cell segmentation was performed with DeepCell Mesmer based on the max projection of DAPI channels (compartment “nuclear”, 0.4.0) [32]. Mean intensities and morphological properties were returned per cell using the MCQuant module (1.6.0) [30]. The pipeline was run using Nextflow [33].

While in some instances the MCMICRO analysis pipeline may demonstrate comparable performance to the QuPath pipeline (see Supporting_Figure_MCMICRO_vs_QuPath in phenoimager2mc staging module (see Availability of source code and requirements)), MCMICRO offers the advantage of enabling batch processing of multiplex imaging data such as SignalStar.

TCR repertoire data

The TCR CDR3b, CDR3a sequences and associated peptide epitope sequence data underwent conversion into embeddings using recent techniques in Large Language Model (LLM) technology. Evolutionary Scale Modeling (ESM) [34] has recently harnessed LLMs to create a collection of protein language models. Specifically, we utilized the esm2_t33_650M_UR50D model from ESM to initially generate embeddings for TCR CDR3b, CDR3a and peptide epitope sequences. These embeddings, characterized by a high dimensionality (1280 dimensions), were independently created for TCR CDR3b, CDR3a, and peptide epitope sequences. Subsequently, we concatenated the embeddings (1280D for TCR CDR3b and 1280D for peptide; 1280D TCR CDR3a, 1280D CDR3b, and 1280D peptide) to capture the combined information pertaining to TCR-antigen interactions.

The concatenation of these embeddings results in a feature vector (2560D for CDR3b and peptide; 3840D for CDR3a, CDR3b and peptide) that encapsulates the unique characteristics of both the TCR and antigenic sequences. This combined representation aims to capture the intricacies of TCR-antigen interactions. PCA was then applied to these combined embeddings, and the first 30 principal components were used in APP clustering (minimum cluster size = 100). This approach enabled thorough exploration and analysis of the dataset, unveiling intricate patterns and relationships within the combined TCR-antigen sequence space.

The sequence similarity within each class (CDR3a, CDR3b and peptide epitope) was calculated by aligning each pair of sequences and computing the Blosum62 score for the alignment (utilizing the Bio.pairwise2 module in the Biopython package [35]). The Blosum62 score offers a quantitative measure of the similarity or dissimilarity between amino acids at specific positions in protein sequences, relying on observed frequencies of substitutions in related proteins. It is commonly utilized in sequence alignment algorithms to assess the evolutionary relationships between proteins and identify regions of conservation or divergence. Within each cluster, an average sequence similarity was calculated by averaging the scores for each unique pair of sequences found in that cluster. Between each pair of clusters, an average sequence similarity score was calculated by averaging the scores for each unique pair of sequences between the two clusters. Unique pairs were used to avoid biasing within-cluster average scores for clusters containing many repeated sequences.

Cluster sequence logos for epitope peptides were generated by selecting all sequences of uniform length. This length was defined as the rounded average sequence length among all sequences in a cluster within a given class of sequences. This approach ensured the ~70-80 percent (varies among the clusters) coverage for epitope sequences. Cluster sequence logos for CDR3 sequences were calculated by aligning CDR3a and CDR3b sequences using ANARCI, using temporary pseudo sequences to fill in the gaps and simulate full TCR sequences [36]. The distribution of amino acid residues at each position was then calculated for these sequences. The Python

package LogoMaker was employed to create probability matrices for the sequence logos, which were subsequently utilized for the analysis of amino acid R group properties.

TCR-pMHC crystal structure analysis

Crystal structures under PDB accession codes 3GSN, 3PQY, 1OGA, 3O4L and 5EUO were used to analyze interfaces between antigen and TCRa, antigen and TCRb, TCRa and TCRb. For each structure the analysis was performed using PISA service [37,38]. A manual verification was performed for each structure using PyMOL Molecular Graphics System, Version 1.2r3pre (Schrödinger, LLC). Figures were generated with PyMOL.

Automated projection pursuit clustering based on the best separation score

The overall data clustering workflow (Figure 1) is constructed to unambiguously assign a cluster identification number (ID) to each data point in the data set by recursively performing the following three steps: 1) presenting the multidimensional data in all its two-dimensional (2D) orthogonal projections; 2) for each 2D projection finding the decision boundary (i.e. the boundary separating cluster assignments) according to the local minimum density of the data points; 3) choosing the 2D projection that has decision boundary with the highest Calinski-Harabasz [39] score, splitting the data along this decision boundary. The Calinski-Harabasz index, that is often used to evaluate the goodness of split, is calculated as a ratio of the sum of inter-cluster dispersion and the sum of intra-cluster dispersion for all clusters (where the dispersion is the sum of squared distances). These steps 1 and 2 are repeated recursively and exhaustively until there are no further splits, as defined by the user-input minimum cluster size parameter. This parameter should be set based on the smallest population the user expects to detect in the dataset.

The generalized structure for such an algorithm is as follows.

In each recursive step:

If the number of points at the input to the recursion step is less than 2* min_cluster_size (user defined parameter), then this piece of data is considered as the final cluster, cluster ID is assigned, and no further splits are performed. The algorithm exits the recursion step.

Otherwise, for each 2D (x,y) projection mapped onto a unit square (side length of 1), we build the Gaussian-smoothed histogram H(x,y) of the data points - that is, the initial distribution density H. For the optimal number of bins of 2D histogram, we use Mann's formula [40], taking into account the number of data points *n* in the current 2D projection. The challenge of determining the optimal number of histogram bins, contingent on the number of data points, remains an ongoing issue without unanimous consensus in the literature. The optimal choice must strike a balance between having too few bins (resulting in poor resolution) and too many bins (leading to increased noise). The Gaussian smoothing width σ is taken as a free parameter of the algorithm. Then, the number of bins *N* for each of the two coordinates is the square root of the total number of two-dimensional bins (as determined by Mann's formula) multiplied by the width of the Gaussian smoothing:

$$N = 4\sigma[3(n-1)^2/4]^{0.1} \quad (1)$$

Thus, the use of Gaussian smoothing not only reduces the statistical noise of the data, but also increases the number of histogram bins. Calculating the optimal number of histogram bins depending on the number of data points considered at each recursion step made it possible to significantly speed up the calculations, since it reduced the number of algorithm operations due to the reduction in the size of clustered projections during program operation.

To initiate the search for a decision boundary function $y(x)$ (Supplementary Figure 1) we build H_1, a function that is a sum of the initial distribution density H and a parabolic function H_g - the "y_0-gravity potential". The function $H_g(y)$ is added for partial straightening the decision boundary function $y(x)$ along the *x*-axis. Addition of the $H_g(y)$

function to the data histogram results in a constraint for the following decision boundary condition: $y(x_0, q) = y_0(q)$ and in a constraint that $y(x, q)$ will be as close as possible to the $y_0(q)$ for every $x \in [x_{min}, x_{max}]$. Where q is the variable being optimized, writing $y(x)$ instead of $y(x, q)$ implies that q has taken its optimal value.

$$H_g(y, q) = (y - y_o)^2;\ y_o = q y_{max} + (1 - q) y_{min} \quad (2)$$

Where $q \in [0, 1]$ is a numeric parameter.

$$H_1(x, y, q) = H(x, y) + k H_g(y, q) \quad (3)$$

Here, k is a positive constant, the optimal value of which is calculated by the following expression:

$$k = \beta \cdot (max(H) - min(H))/max(H_g) \quad (4)$$

The coefficient k plays a crucial role in achieving a balance between the parabola and the data, particularly in determining the trajectory of the decision boundary. The greater the coefficient k, the greater the straightening effect. Here, the multiplier β is a free parameter that varies the degree of influence of the "y_0-gravity potential" on the clustering process. Our empirical assessment shows that a value of β=0.1 (i.e. 10% "gravity") gives fairly good clustering results in many cases.

To "draw" a decision curve on a 2D plane one needs to know its initial [$y(x_{min})$] as y_o and final [$y(x_{max})$] as y_1 boundary conditions. The explicit, but more computationally intense, solution would be to search for a decision boundary for each possible y_o and y_1 in a given 2D projection. To optimize this process, we instead made the parabolic function H_g depend on a parameter q that is used to introduce the next incremental step along the axes (i.e., δq = 0.1).

To find the decision boundary with the smallest data density between the resulting clusters, for every parameter value $q \in [0,1]$ (Supplementary Figure 1), we search for extremals $f_q(x)$ (an analogue of trajectory from analytical mechanics) and the curvilinear integral S(q) (an analogue of action from analytical mechanics) of the probability density along the decision boundary:

$$f_q(x) = argmin_{y(x,q)} \int_{x_{min}}^{x_{max}} H_1(x, y(x, q), q) dx \quad (5)$$

$$S(q) = \int_{x_{min}}^{x_{max}} H(x, f_q(x))dx \quad (6)$$

Further, we need to find the values q_0, that would satisfy the following condition: $S(q_0 - \delta q) > S(q_0) < S(q_0 + \delta q)$. For such values, $f_{q_0}(x)$ will be a true extremal or decision boundary. Once a decision boundary is found, data is then split along that decision boundary. If both parts of the data obtained after splitting contain more than min_cluster_size cells, then this decision boundary is added to the list of decision boundaries for a given projection. If more than one decision boundary is found on a given projection, these decision boundaries are then ranked according to their Calinski-Harabasz score, and the decision boundary with the maximum Calinski-Harabasz score is chosen to represent the given projection. In our algorithm, the same projections are analyzed twice to determine a 2D cluster boundary in (x, y)-space: first to identify the x-projection of the boundary and then to determine its y-projection.

Then, among all possible 2D projections at a given recursion step, the algorithm chooses the one that contains the decision boundary with the maximum Calinski-Harabasz score. The data is then split along this decision boundary, and the new recursive step is initiated on each of two data pieces independently. If, however, there are no candidate decision boundaries (because every boundary leads to a split for which one part of the data contains less than min_cluster_size cells), the algorithm assigns the final cluster ID to this piece of data and recursion stops.

APP has only one required user input parameter: the minimum cluster size, which should be set based on the smallest population the user expects to detect in the dataset. Reducing this value introduces smaller clusters, as expected, while other APP clustering outcomes remain unchanged. All other parameters, such as Gaussian smoothing width, are optional. We have set default values optimized for the Calinski-Harabasz index based on the datasets presented in the manuscript. These parameters are user-adjustable to allow optimization for other data types, guided by the Calinski-Harabasz index.

Automated label transfer across samples

At a high level, the label transfer pipeline enables the use of a labeled (or partially labeled) set of points to learn a metric on the data. This learned metric is then employed as a measure of distance between new, unlabeled points. The immediate practical applications of such a pipeline, demonstrated here, include the automation of an expert-defined manual gating strategy, and the assessment of clustering algorithm performance against the ground truth cluster labels. Our concise four-step pipeline facilitates the visualization and quantification of the misclassification rate between the clustering algorithm and the ground truth labels (see Supplementary Figure 2).

The first step of the pipeline involves creating a supervised UMAP embedding [19] using labeled or partially labeled training sample(s) with both marker expression data and ground truth cluster labels. UMAP is applied to this training data with the goal to learn a distance metric that best separates the classes while preserving their relationships in the marker space. Using equal weights (target_weight=0.5 as described in [41]) for marker expression and ground truth cluster labels ensures that both data-driven and prior knowledge are considered in the embedding.

This step itself provides an opportunity to assess the quality of the ground truth cluster labels by observing an agreement (or disagreement) between the data topology and clustering decisions. By observing the agreement or disagreement between the data's topological structure in the UMAP space and the provided ground truth cluster labels, one can gain insight into whether the ground truth labels accurately reflect the underlying structure of the data (Supplementary Figure 3). High agreement between the UMAP topology and the ground truth labels suggests that the ground truth labels are representative of the data's natural clustering patterns. Conversely, disagreements may indicate issues with the ground truth labels.

In the second step (Supplementary Figure 2), the set of labeled points is employed to learn a metric on the data. This learned metric subsequently serves as a distance

measure between new unlabeled points, facilitating the projection of an unlabeled test set into the UMAP embedding space constructed using the training set. This ensures that the test set occupies the same reduced-dimensional space as the training set.

In the third step, the test set is subjected to clustering using the Support Vector Clustering (SVC) [42] algorithm directly applied in the supervised UMAP embedding space. Given that clustering in this context is confined to two dimensions (UMAP_x and UMAP_y), we opted for an algorithm that refrains from assigning any of the events to noise and, at the same time, offers computational superiority over APP. Subsequently, the QFMatch algorithm [43] is employed to align the cluster labels between the test set (with cluster IDs defined by SVC or assigned by the clustering algorithm under assessment—see Supplementary Figure 4) and the training set (with ground truth cluster IDs). The alignment of labels is crucial as it accommodates the following scenarios: 1) transferring cluster labels from the test set to the training set; 2) directly assessing the agreement of clustering decisions made by multiple clustering algorithms (see Supplementary Figure 4).

In the final step, we compute the number of misclassified events per cluster ID. This quantifies how effectively the clustering algorithm has assigned data points to clusters in comparison to the ground truth.

Beyond clustering algorithm evaluation, this pipeline holds broader applications in supervised learning tasks. As demonstrated here, it can be employed to transfer labels from one sample to another or from a partially labeled dataset to the remaining data in a given set. This feature proves particularly valuable in scenarios where labeled data is limited.

Results

To encompass the range of real-world scenarios for clustering algorithm usage, both with and without ground truth or domain knowledge, we first demonstrate the method's

performance using a dataset with a functionally validated ground truth. We then apply the method to a dataset where expert-defined manual gating serves as the "ground truth." Next, we test the algorithm on single-cell mRNA expression data and imaging data, which lack predefined ground truth. However, cluster evaluation is enabled using domain knowledge from pathology and immunology, leveraging expression patterns and spatial distribution. Finally, we apply APP clustering in a fully exploratory mode, where no ground truth or pre-existing domain knowledge is available, to assess how TCR receptor embeddings cluster with respect to their cognate antigens.

Performance on data with functionally validated ground truth labels

To objectively assess APP's performance against widely used clustering algorithms in application to realistic, biologically relevant data, we used ground truth data where each cell population was quantified and functionally validated. To generate such biologically relevant data with a known ground truth, we combined spleen cells from the GFP+Wild-type and RAG-KO mice at five different proportions (Figure 2A). RAG-KO mice are deficient in generating lymphoid lineages, while GFP+ mice represent a healthy immune system with constitutive GFP expression in all immune cell lineages. This allows for easy and accurate detection of immune cell populations by flow cytometry, simplifying the identification and quantitation of cell lineages. The latter ensures that the clusters obtained by clustering algorithms can be compared against a known biological truth [44].

To assess the performance of the APP algorithm in comparison to state-of-the-art clustering algorithms, we selected two generally widely used high-dimensional clustering algorithms, irrespective of data origin: HDBSCAN [45] and KMeans [46]. Additionally, we included Phenograph [47], FlowSOM [9], and SPADE [10], as methods that are widely used in the flow cytometry field. Our selection of algorithms reflects a diverse range, encompassing density-based (HDBSCAN), centroid-based (KMeans), graph-based (Phenograph), self-organizing map-based (FlowSOM), and tree-based density-normalized (SPADE) clustering methods. This ensures a comprehensive

comparison, considering different clustering paradigms and their suitability for various data structures.

As demonstrated here, HDBSCAN, KMeans (K=8), Phenograph, FlowSOM, and SPADE encounter challenges in robustly detecting rare cell populations that coexist with more abundant cell populations in the same sample (Figure 2B-D). When clustering algorithms operate across multiple dimensions simultaneously, they may face difficulties in effectively detecting and distinguishing sparse populations from more prevalent ones. The increased sparsity within the vast high-dimensional space poses a challenge in identifying clusters that exist in lower-dimensional subspaces. Our findings illustrate that even with seven dimensions (excluding live/dead PI from clustering), clustering algorithms may encounter challenges when dealing with multiple dimensions simultaneously.

Our findings suggest that the APP clustering method excels in scenarios where there are clear distinctions between the cluster under consideration and the other cells in at least one dimension. The APP method's proficiency in identifying clusters with evident separations in one or more dimensions makes it well-suited for situations where distinct cell populations exist. In such cases, it can leverage the dimension(s) where the separation is apparent to successfully identify and differentiate clusters, even in the presence of much larger populations and noise in the data. This aligns with scenarios resembling cell phenotyping using flow/mass cytometry, imaging antibody panels, and scRNAseq data, where there are often identifiable patterns or markers distinguishing cell types. Another application, as demonstrated here, is clustering molecules, such as TCRs and their cognate peptides, based on their sequence similarity and other features.

On the other hand, high-dimensional clustering approaches may potentially outperform the APP method when there is no clear split between clusters in any of the dimensions, and the information about a given cluster is "distributed" across multiple dimensions. High-dimensional clustering algorithms excel in aggregating information from multiple dimensions simultaneously, which can be advantageous in situations where cluster

boundaries are less well-defined. An example of this is the classification of cells' activation states.

To mitigate some of the limitations of APP in scenarios with less clear cluster separations, we explored incorporating dimensionality reduction techniques, such as PCA, as a pre-processing step. As detailed in subsequent Results sections, this approach proved effective in revealing underlying structures in high-dimensional data.

Application to flow and mass cytometry data

The ability to simultaneously measure multiple cell parameters through high-dimensional flow cytometry has enabled transformative discoveries in medicine, including identifying new cell types and cellular mechanisms critical to disease pathology. As a result, high-dimensional flow cytometry became indispensable for biomedical and clinical research, medical diagnosis, and therapy assessment. The gold-standard approach in analyzing flow cytometry data relies on user-defined manual gating on sequential two-dimensional data projections. However, as the technology evolves and the number of cell parameters that can be measured simultaneously drastically increases, it becomes impractical and often impossible to rely on user-defined manual gates.

To address this limitation, the flow cytometry community has developed new automated methods that simultaneously analyze multiple cell parameters in high-dimensional space [8, 48]. Much effort has been directed to standardize these automated methods and develop guidelines for the user, including through the flowCAP initiative [49]. However, while these methods proved helpful in some studies, reproducibility has become a major concern, as cell types and phenotypes were often not preserved across different methods. As the community recognizes the limitations of the automated high-dimensional analysis, including the curse of dimensionality, the original sequential manual gating in 2D data projections has remained the de facto gold standard. Therefore, there is an emerging need to develop new automated and scalable data analysis approaches that leverage the ground truth of 2D manual gating.

To address this need, we developed APP for unsupervised discovery of cell populations and an automated label transfer pipeline for expert-defined gating automation. We expect APP to facilitate the reproducible analysis of large-scale datasets that rely on automated gating strategies on sequential 2D projections of the data, mitigating user-defined subjective gating and avoiding the curse of dimensionality that affects other methods.

Using an expert-defined manual gating strategy as the "gold standard" (Supplementary Figure 5), we evaluated the performance of APP in characterizing PBMCs from healthy donors and COVID-19 patients. APP demonstrated an overall performance accuracy exceeding 95 percent, significantly outperforming one of the widely used clustering algorithms in the flow cytometry field, Phenograph (Figure 3A,B). The primary source of misclassification for both algorithms stems from sparse cell populations (Supplementary Figures 6, 7). A detailed comparison (Supplementary Figures 6,7) reveals that APP and Phenograph may misclassify different portions of the data, and this discrepancy can be attributed to the distinct clustering logic employed by the two methods.

Furthermore, we assessed the label transfer pipeline's capability to automate the expert-defined gating strategy. The pipeline achieved remarkably high performance for the healthy control cohort, exceeding 99 percent. This level of accuracy was attained even when using just one randomly chosen manually gated healthy control sample as a training set (Figure 3C&D). In the analysis of COVID-19 samples, there were more discrepancies between the manual gating strategy established on healthy control samples and the label transfer pipeline's outcomes. However, as shown here, this apparent discrepancy may indicate that the original expert-defined gating strategy, established on healthy control samples, might require adjustment when applied to disease samples. For example, as demonstrated here, COVID-19 samples exhibit more complex and diverse cell populations than the healthy control samples used to establish the initial manual gating strategy. COVID-19 induces changes in immune cells, leading to transitional cellular states that are dynamic and vary across patients based on factors

such as demographics, treatment regimen, comorbidities, viral load, and the time from infection to analysis. As a result, samples from COVID-19 patients are expected to show greater variability compared to healthy controls. However, we recently demonstrated that despite this variability, ICU patients with severe COVID-19 remain distinguishable from healthy controls using flow cytometry to characterize major immune lineages and cellular states [20].

Our label transfer pipeline includes an immediate sanity check to assess the quality of the "ground truth" labels. This check examines whether the ground truth labels align with the underlying data topology. If there is a disagreement, such as a cell population defined as homogeneous in manual gating but being spread across multiple clusters on the supervised UMAP plot, it suggests a potential issue with the original gating strategy (Figure 3E). Indeed, revisiting the original expert-defined gating strategy, as illustrated in Figure 3F&G and Supplementary Figure 5B, led to the discovery of a COVID-19-specific cell population labeled as HLA-DR–, CD86Lo/– intermediate monocytes (iMo).

Thus, APP identified a new population of myeloid cells specifically enriched in hospitalized COVID-19 patients. Notably, this myeloid cell subset lacks cell-surface expression of key proteins relevant for antigen presentation (HLA-DR) and co-stimulation of T-cells (CD86), likely affecting viral antigen presentation and T-cell activation. Although our new findings might represent a novel mechanism of immune modulation in severe COVID-19, further studies with more patients and different disease states are required to determine whether this is a general mechanism of SARS-CoV-2 infection or a phenotype unique to our small patient cohort.

The label transfer pipeline was also tested in its application to mass cytometry data, achieving 87 percent accuracy (Supplementary Figure 8) compared to the manual gating labels described in [21]. Discrepancies between the underlying data topology and manually assigned cell populations (Supplementary Figure 8) were a source of reduced accuracy for the label transfer pipeline.

Application to scRNAseq data

In contrast to flow and mass cytometry, which measure dozens of dimensions for each individual cell, the dimensionality of gene expression data is often on the order of thousands of genes per cell. Given the high-dimensional nature of gene expression data, dimensionality reduction techniques, such as PCA (Principal Component Analysis), are often employed as a pre-processing step before clustering to extract meaningful patterns and reduce the computational complexity associated with analyzing a large number of genes. We decided to test APP clustering performance on PCA preprocessed scRNAseq data, since dealing directly with the combinatorial combinations of all possible pairwise projections from thousands of genes can be computationally prohibitive.

For this purpose we choose a publicly available scRNAseq dataset generated from human PBMCs and processed by 10X Genomics. We compared APP clustering performance to the performance of a clustering process using KNN and Louvain algorithms as implemented in the R package Seurat (referred to in this manuscript as “Louvain clustering”, and for our purposes, treated as the ground truth since it is a workflow widely used by the field today). The overall misclassification rate between Louvain clustering and APP clustering decisions in the PBMCs dataset is about thirty percent, but less than fifteen percent overall when memory CD4 T cells are excluded (Figure 4 A&B). APP clustering faces challenges in resolving the distinction between Naive and Memory CD4 T cells in the dataset (Figure 4B), likely due to the fact that these cells essentially represent distinct functional states and responses from the same population of cells. There is no clear split between these two populations in any pairwise dimensions explored by APP (see Supplementary Data on Zenodo), since the differentiation between Naive and Memory CD4 T cells may rely on simultaneous changes in several genes, and that gene set can vary based on factors such as the context of the immune response or the microenvironment.

In our analysis, we further examined the group of cells that Louvain clustering identified as B cells and APP as T cells, labeled as "CD4/CD8" in Figure 4A. We projected this

cell population in B cell, T cell and other marker space to gain insights into the APP algorithm decision logic (see Supplementary Figure 9). It becomes apparent that while the "CD4/CD8" cell population exhibits high expression of MS4A1, a B cell-specific marker, it also demonstrates relatively high expression of S100A4, which is a memory CD4 T cell marker, not a B cell-specific marker. This dual expression pattern likely contributed to the source of confusion for APP clustering.

Louvain clustering and APP clustering were next applied to a snRNA-seq mouse brain dataset with wildtype and Alzheimer disease model samples (Figure 4C). The overall mismatch rate between the two methods was about thirteen percent, indicating a good level of agreement (Figure 4D). In this dataset, we did not see any “ground truth” clusters that could not be discovered through APP clustering, as we did in the PBMC dataset. All Louvain clusters had equivalent APP clusters with matching marker genes (Figure 4C). The significant areas of discrepancy tended to be in cells which were assigned to their original Louvain clusters with some level of ambiguity.

To assess the computational complexity of the APP algorithm, which is generally O(N^2), where N is the number of dimensions, we recorded the clustering time for APP versus Seurat v5 using a MacBook Pro with a 2.3 GHz Dual-Core Intel Core i5 and 8 GB memory. For the PBMC dataset (containing 2700 cells), Seurat clustering (resolution = 0.5) took 1–2 seconds in real time. APP, with 10 PCs and a minimum cluster size of 10, took 11m 19.952s real time, 26m 6.587s user time, and 2m 43.988s system time. APP, with 10 PCs and a minimum cluster size of 100, took 2m 16.023s real time, 5m 47.742s user time, and 0m 34.924s system time.

Application to multiplex imaging data

Recent advancements in multiplex imaging technologies, exemplified by SignalStar, have greatly enhanced our capacity to profile individual cells within the tissue context. These technologies enable the simultaneous visualization of multiple biomolecules at the single-cell or even the subcellular level. This capability provides valuable insights into cellular heterogeneity, spatial organization, and tissue composition.

We applied APP clustering to characterize cellular composition within the tissue context of the human squamous lung carcinoma sample using the 8-plex SignalStar myeloid cell panel. The 8-plex panel (CD11c, SIRPα, CD163, CD206, CD68, CD45, HLA-DRA and Pan-Keratin) generated a dataset of 160 features per cell for approximately 230,000 cells. The 160-feature set comprises 8 antibodies x 5 statistics x 4 cell compartments. Here, the 5 statistics are the measurements of marker mean, median, maximum, minimum, and standard deviation, and the 4 cell compartments are the nucleus, cytoplasm, membrane, and whole cell. We used PCA to reduce the dimensionality of the dataset to 30 dimensions and subsequently performed APP clustering (see Figure 5A&B).

This clustering approach readily identifies various cell types, including tumor epithelium, stromal, and immune cells. The marker composition of the panel reflects the functional heterogeneity within the myeloid compartment: populations of macrophages and monocytes (Clusters 9 and 7), typically identified by prominent expression of CD68 and CD206, can further be subdivided based on high vs low expression of CD11c and SIRP-alpha [50]; similarly, CD11c+ dendritic cells (DCs) can belong to CD163-high or CD163-low (Clusters 0 and 4, respectively), functionally distinct populations [51-53].

The representation of individual clusters (Figure 5A) not only demonstrates phenotypic heterogeneity but also provides valuable clues regarding the relative proportion of phenotypes. Macrophages are known to be prominent cell populations within the microenvironment of diverse human tumor types including NSCLC [54,55], and based on the size of the combined clusters, macrophages are a highly prevalent cell type in the tumor microenvironment of this squamous NSCLC. PanCK expression identifies epithelial cells (in this particular case, cancer cells) unambiguously, and the majority of PanCK+ cells seem to be low or negative for all other markers (cluster 6). However, two smaller PanCK+ clusters, 2 and 3, are identified showing elevated levels of HLA-DR; induction of HLA-DR expression in malignant epithelial cells is a known phenomenon, particularly in an inflammatory milieu [56,57]. Interestingly, cells of these two clusters

seem to preferentially localize to the epithelial-stromal interface with physical proximity between epithelial and immune cells and a potentially high local concentration of inflammatory cytokines.

Cell type label assignments were determined through a combination of marker expression patterns and the spatial location of identified clusters on the tissue slide (Figure 5 C&D). Since this dataset lacks ground truth labels, assessing clustering results requires considering both the expression patterns of panel markers and the spatial distribution and co-location of identified cell phenotypes within the tissue context. Therefore, domain experts evaluated the biological relevance of APP clustering decisions. Pathology and immunology experts (see Acknowledgement) independently assessed and confirmed the adequacy of APP clustering in characterizing and distinguishing meaningful cell populations within the slide tissue. ScRNA-seq or spatially resolved RNA-seq from the same sample or a serial section could serve as an orthogonal approach to assess cluster quality. However, we did not have further access to the sample used in this study for additional analysis.

Application to TCR-peptide sequence representation data

Recent advancements in Large Language Models (LLMs) have shown significant promise for applications in the analysis of protein sequence data [58-60]. LLMs have been investigated for functional annotation of protein sequences [61], demonstrating capabilities in predicting protein functions, interactions, as well as antibody and TCR specificity [62,63]. These models excel at capturing contextual relationships within sequences. In the context of TCR sequences, this proficiency involves understanding the specific arrangement of amino acids and their roles in recognizing and binding to antigens. Analyzing the language-like patterns in TCR and antigen sequences using these models may unveil insights into binding specificity.

Although LLMs showcase remarkable capabilities in capturing intricate patterns and relationships within sequences, their black-box nature can pose challenges to interpretability. To address this, clustering techniques can be employed to interpret LLM

decisions. In this example, using human and mouse TCR sequences and their cognate antigens (Figure 6), we illustrate that the combination of LLMs and APP clustering methods offers a synergistic approach for uncovering patterns, relationships, and semantic structures within large and high-dimensional TCR-antigen sequence datasets.

To illustrate this point, we created embeddings for over 13,000 unique data points containing TCR CDR3b and cognate epitope sequence information for over 350 unique peptides (left side of Figure 6A). Additionally, we independently generated embeddings for a subset of approximately 4000 unique TCRs with sequence information for both TCR CDR3a and CDR3b (right side of Figure 6A). These embeddings were generated independently for each sequence class (CDR3a, CDR3b, and epitope) and then concatenated for CDR3b-epitope and CDR3a/b-epitope. PCA was subsequently performed, and the first 30 PCs were utilized for APP clustering, employing a minimum cluster size of 100. The composition of identified clusters was characterized based on epitope content and association with disease information retrieved from the McPAS-TCR database (Figure 6B).

By combining embeddings constructed independently for TCRs and peptides, we generated a fused representation that captures similarities on both the TCR and peptide sides (Supplementary Figure 10). This approach highlights co-similarities between TCRs and peptides across multiple pairs, enabling exploration of the joint sequence space of TCR-peptide interactions. This comprehensive view could offer valuable immunological insights into the complexities of immune recognition, cross-reactivity, specificity, and diversity.

The inherent black-box nature of LLMs presents a challenge in immediately interpreting the rationale behind the grouping of data points within embeddings. Introducing a clustering approach to these embeddings and subsequently overlaying domain knowledge and interpretable feature information onto the clustering decisions provides an opportunity to partially unravel the decision-making process of LLMs.

One evident hypothesis to investigate was assessing whether sequence similarity played a significant role as one of the driving forces behind the clustering of TCR-peptide data points within the class-separated and the combined embeddings. This investigation aimed to uncover the impact of sequence similarity on the grouping patterns within the combined embeddings, providing valuable insights into the underlying factors influencing the model's behavior. To evaluate this hypothesis, we calculated sequence similarity independently for peptides, TCR CDR3b, and TCR CDR3a among all pairwise combinations of clusters (refer to Figure 6C and Supplementary Figure 10).

The within-cluster average sequence similarity consistently displayed higher scores compared to inter-cluster average sequence similarity in all three cases: for peptides, CDR3a, and CDR3b. This is evident from the observed higher Blosum62 score diagonal pattern on the heatmaps (Figure 6C, Supplementary Figure 10, Supplementary Figure 11 A,C). As depicted in Supplementary Figures 10 and 11 sequence similarity, particularly in cases of peptide epitopes, plays a significant role in data clustering when class-separate embeddings are constructed. A clear pattern of peptide clustering based on disease categories (pathogens vs cancers, etc.) is observed. However, once TCR and peptide embeddings are concatenated, the data points undergo significant rearrangement in the embedded space. The disease of origin becomes a less significant feature for peptides. Overall, while sequence similarity between peptide epitopes and between TCRs still contributes to clustering, it becomes less prominent, as evidenced by a significant decrease in the median Blosum62 score on the diagonal, particularly for epitope peptides.

Certain peptide groups, such as those within cluster 4 on the right side of Figure 6B, originate from antigens associated with diseases that are not immediately related. Cluster 4 predominantly consists of peptides derived from cancer neoantigens and influenza. TCRs recognizing these peptides also exhibit a closer sequence similarity in their CDR3a and CDR3b regions within cluster 4 compared to TCRs outside of this cluster (Figure 6C) . This suggests a potential shared recognition pattern among TCRs

responding to sequence-similar peptides from disparate diseases within the same cluster.

The clustering based on sequence similarity indicates a commonality in the TCR responses, implying a common recognition pattern that extends across distinct disease contexts. This observation could potentially be explained via TCRs' promiscuity towards structurally similar epitopes, even if they originate from different antigens. This promiscuity could be facilitated by commonalities in binding motifs among TCRs. Nonetheless, our analysis, illustrated in Supplementary Figure 11, reveals the absence of an immediately discernible common binding motif. This holds true even among CDR3b sequences that specifically recognize the same peptide epitope (LPRRSGAAGA). It is worth noting that the promiscuity works both ways, namely that a TCR can recognise multiple epitope peptides, but also an epitope peptide can be recognized by multiple TCRs. Our clustering results show that the recognition patterns can be very different, since different peptide-TCR pairs corresponding to the same peptide are often sorted into different clusters (Supplementary Figure 11).

By further exploring the joint sequence space, we observed a novel pattern of "similarity" between the data points. This led to significantly different within-cluster arrangements for both peptides and TCR CDR3b. The label transfer pipeline was employed to quantitatively assess the degree of rearrangement that both peptide and TCR CDR3b classes undergo between the single class embeddings and concatenated embeddings (Supplementary Figure 12). Only 23 percent of peptide epitope data (and 12 percent for TCR CDR3b) is arranged in similar patterns between the single class and concatenated embeddings space. This suggests that, during the exploration of the joint sequence space of TCR-peptide interactions, a new rationale for data point similarity emerged that is not necessarily related to the origin of the antigen peptide.

In our pursuit of a deeper understanding of the arrangement of data points in the joint sequence space, we explored the possibility of utilizing the characteristic properties of amino acid R groups as a novel similarity criterion. However, we found no immediate

patterns of complementarity or cluster-specific motifs defined by the properties of amino acid R groups (refer to Figure 6D, Supplementary Figures 10&11). The diversity among TCRs and epitope peptides suggests that the mechanisms of interaction can vary significantly between individual TCR-peptide pairs. For instance, in Supplementary Figure 13, we highlight a case where a positively charged residue from the peptide side interacts with the TCR chains through the formation of hydrogen bonds, underscoring the intricate and diverse nature of these molecular interactions.

An intriguing pattern did emerge, though, revealing a prevalence of negatively charged amino acid residues on CDR3b and positively charged residues on CDR3a chains across almost all identified clusters (see Supplementary Figure 13). To delve into the potential significance of these charged residues, we meticulously examined five TCR-pMHC crystal structures (PDB accession numbers 3GSN, 3PQY, 1OGA, 3O4L, 5EUO). Upon closer investigation of these structures, it became evident that these charged amino acid residues play a role in stabilizing the interface between CDR3a and CDR3b, orienting partners to each other in three-dimensional space. Most frequently, this stabilization occurs through hydrogen bonds, although there is also an instance of ionic interaction (Supplementary Figure 13E). However, follow-up studies will be necessary to further validate the findings and strengthen these claims.

It is also important to note that while LLMs could potentially be biased by learning patterns from the limited training data provided, we have recently shown [64] that, regardless of the grouping approach (e.g., distance-based clustering, LLM, etc.), only a minority of TCRs form pure clusters predominantly composed of peptide-specific TCRs.

Conclusions

Identifying robust patterns in high-dimensional data is a challenging and crucial task in various scientific fields. High-dimensional data often poses challenges such as the curse of dimensionality, where traditional methods may struggle due to increased sparsity.

Projection pursuit has been proposed as a potential approach to address or mitigate some of the challenges posed by the curse of dimensionality. As a concept, this technique has been around for several decades, and its development and application span across different fields such as statistics, machine learning, and data visualization. The idea of seeking interesting projections of high-dimensional data can be traced back to the 1970s and 1980s [3-5, 65].

While the concept of projection pursuit has been around for decades, high-dimensional clustering (such as HDBSCAN, KMeans, and SPADE) has gained more popularity and attention, especially as an approach to the computational challenges posed by modern data analysis. High-dimensional clustering methods are explicitly designed to directly address the task of grouping data points into clusters. Projection pursuit approaches were originally designed as powerful tools for exploring interesting projections, not clustering. Additionally, the projection pursuit approach is challenged by the many low-dimensional projections generated from modern high-dimensional data.

Here, we have integrated the principles of projection pursuit and clustering to create an automated projection pursuit clustering approach, designed to reveal noteworthy structures in high-dimensional data and assign cluster labels. The "projection" aspect of our approach involves orthogonal projection of high-dimensional data into a two-dimensional space. The "pursuit" component is guided by the concept of, at each step, identifying a projection with the smallest data density distribution along the decision boundary. The automated clustering aspect is executed through the recursive and exhaustive application of the "projection" and "pursuit" steps. To facilitate the computational challenge associated with exploring a high number of low-dimensional projections, we reduced the number of operations required by the algorithm at each recursive step by adopting the calculation of the optimal number of histogram bins. We also implemented parallelization of the APP algorithm enhancing its computational performance. Future improvements may focus on additionally optimizing specific algorithm components and/or exploring alternative computational strategies such as leveraging high-performance computing when a relatively large amount of high-dimensional data is needed to be processed. Also, in some cases clusters that are well-

separated in higher-dimensional space may appear mixed when projected onto certain 2D planes. To address this, proper rotation of the coordinate system may help uncover projections that better reveal separation. While our current approach does not explicitly incorporate rotation optimization, one potential future extension could involve employing rotation search methods such as those used in projection pursuit to identify projections that maximize separation between clusters.

In general, projection pursuit clustering can offer advantages in certain scenarios compared to traditional high-dimensional clustering methods. Projection pursuit can be effective when certain dimensions or variables are more important for clustering than others. It actively seeks projections that highlight important features, focusing on relevant dimensions and potentially improving cluster separation.

In scenarios characterized by sparse or imbalanced data (such as the dataset presented in Figure 2), where there is a high degree of sparsity amid more abundant populations (which can extend to situations involving outliers and/or noise), projection pursuit proves beneficial in identifying pertinent dimensions and enhancing cluster separation. Traditional methods (such as HDBSCAN, KMeans, Phenograph and SPADE) may face challenges in handling sparsity (see Figure 2C) due to a dearth of informative features. Projection pursuit seeks to discover projections that are not only conducive to clustering but also interpretable. If the objective is to extract meaningful insights from clustering outcomes and comprehend the contributions of individual dimensions, projection pursuit may be the preferred choice.

The approach of discovering patterns through projection pursuit and clustering proves to be versatile across various data modalities. In this context, our focus was specifically on high-dimensional biological data given its frequent representation of both abundant and sparse populations within the same sample. We demonstrated APP’s capability to replicate experimentally validated cell type definitions, emphasizing the biological relevance of the clusters identified by our approach. We conducted a performance comparison of APP with other widely adopted clustering methods. While APP's results

align well with other methods, there are instances where one method may outperform the other, and we illustrated these nuances using real-world datasets.

In many biological real-world datasets, the availability of a clear "ground truth" can be challenging. As illustrated in the examples presented, reliance on domain experts' knowledge-driven clustering or clustering done with widely-adopted approaches serves as a substitute for ground truth. While expert-driven clustering provides a valuable reference point, a more accurate (albeit labor-intensive) method for assessing clustering performance involves conducting functional tests on groups of cells assigned to the same cluster. By observing the functional "purity" and homogeneity of a given cluster compared to other cell clusters in the sample, researchers can achieve a more precise evaluation of the clustering results.

In one of such examples, we used a data set with functionally validated ground truth and demonstrated that APP effectively recapitulated experimentally validated cell-type definitions better than other widely used clustering approaches. We also presented APP's utility in discovering new biologically meaningful patterns. By combining the strengths of LLMs for sequence analysis with the interpretability provided by clustering, we gained deeper insights into the complex relationships within TCR-pMHC sequence datasets. This integrative approach contributes to the elucidation of patterns that may have important implications for better understanding of TCR and pMHC function and design. Our results also emphasize the ongoing challenges with universal pMHC-TCR specificity machine-learning models, showing that the pMHC-TCR recognition patterns are not always self-evident.

Availability of source code and requirements

Project name: APP
Project home page: https://github.com/tabatsky/projectionpursuit
Operating system(s): MAC, Windows
Programming language: Python
Other requirements: Requirements are listed at the project home page
License: MIT License
RRID: SCR_026560

Project name: phenoimager2mc
Project home page: https://github.com/SchapiroLabor/phenoimager2mc
Operating system(s): MAC, Linux
Programming language: Python
Other requirements: https://github.com/SchapiroLabor/phenoimager2mc/blob/main/environment.yml, optionally Docker or Singularity
License: AGPL-3.0 License
RRID: SCR_026561

Data availability

The dataset of human squamous cell carcinoma stained with SignalStar mIHC is deposited in Mendeley Data [66]. All additional supporting data are available in the *GigaScience* repository, GigaDB [67].

Acknowledgement

We would like to thank Hartmut Koeppen, Senior Principal Fellow at the Research Pathology Department, Genentech, for his critical review of the cell type annotations in the human squamous lung carcinoma sample stained with the 8-plex SignalStar myeloid cell panel.

D.S., K.B. and C.S. are supported by the German Federal Ministry of Education and Research (BMBF 01ZZ2004); the Bruno and Helene Joester Stiftung, the Ministry for Science, Research and Science Baden-Württemberg „AI Health Innovation Cluster“ and "MULTI-SPACE"; and research funding from Cellzome, a GSK company.

The authors gratefully acknowledge the data storage service SDS@hd supported by the Ministry of Science, Research and the Arts Baden-Württemberg (MWK) and support by the state of Baden-Württemberg through bwHPC and the German Research Foundation (DFG) through grant INST 35/1314-1 FUGG and INST 35/1503-1 FUGG. This work is

supported by the Health + Life Science Alliance Heidelberg Mannheim and received state funds approved by the State Parliament of Baden-Württemberg.

This study was supported in part by the National Institutes of Health (NIH) National Institute of Allergy and Infectious Diseases (NIAID) R01AI123126 (EEBG), R01AI123126-05S1 (EEBG), R21AI167032 (to EEBG), and the Lowance Center for Human Immunology (EEBG). DJE was partially supported by Emory’s Laney Graduate School Fellowship.

We acknowledge the use of OpenAI's ChatGPT for assistance in refining the language and style of this manuscript. The model was utilized to improve clarity and coherence while ensuring that the original scientific content remained intact. All intellectual contributions and interpretations are the sole responsibility of the authors.

Conflict of interest

C.S., S.T., F.G., D.P., T.L., I.G., D.O. are employees of Cell Signaling Technology. D.S. reports funding from Cellzome, a GSK company and received honorariums and fees from Immunai, Noetik, Alpenglow and Lunaphore. K.B. reports fees from Lunaphore.

References

1. Morgenstern, D. & Bellman, R. (1962). Adaptive Control Processes: A Guided Tour. In Econometrica (Vol. 30, Issue 3, pp. 599).

2. Hastie, T., Tibshirani, R., & Friedman, J. (2009). The Elements of Statistical Learning: Data Mining, Inference, and Prediction (2nd ed.). Stanford University Press.

3. Friedman, J. H., & Tukey, J. W. (1974). A Projection Pursuit Algorithm for Exploratory Data Analysis. In IEEE Transactions on Computers: Vol. C–23 (Issue 9, pp. 881–890). https://doi.org/10.1109/t-c.1974.224051

4. Friedman, J. H. & Stuetzle, W. (1982). Projection pursuit methods for data analysis, in Modern Data Analysis, R.L., Launer & A.F., Siegel, eds, Academic Press (pp. 123–147).

5. Huber, P. J. (1985). Projection Pursuit. In The Annals of Statistics (Vol. 13, Issue 2). Institute of Mathematical Statistics. https://doi.org/10.1214/aos/1176349519

6. McInnes, L., Healy, J., & Astels, S. (2017). hdbscan: Hierarchical density based clustering. In The Journal of Open Source Software (Vol. 2, Issue 11, p. 205). The Open Journal. https://doi.org/10.21105/joss.00205

7. MacQueen, J. (1967). Some methods for classification and analysis of multivariate observations. In Berkeley Symposium on Math, Statistics, and Probability (pp. 281-297).

8. Levine, J. H., Simonds, E. F., Bendall, S. C., Davis, K. L., Amir, E. D., Tadmor, M. D., Litvin, O., Fienberg, H. G., Jager, A., Zunder, E. R., Finck, R., Gedman, A. L., Radtke, I., Downing, J. R., Pe'er, D., & Nolan, G. P. (2015). Data-Driven Phenotypic Dissection of AML Reveals Progenitor-like Cells that Correlate with Prognosis. In Cell (Vol. 162, Issue 1, pp. 184–197). Elsevier BV. https://doi.org/10.1016/j.cell.2015.05.047

9. Van Gassen, S., Callebaut, B., Van Helden, M. J., Lambrecht, B. N., Demeester, P., Dhaene, T., & Saeys, Y. (2015). FlowSOM: Using self- organizing maps for visualization and interpretation of cytometry data. In Cytometry Part A (Vol. 87, Issue 7, pp. 636–645). Wiley. https://doi.org/10.1002/cyto.a.22625

10. Qiu, P., Simonds, E. F., Bendall, S. C., Gibbs, K. D., Jr, Bruggner, R. V., Linderman, M. D., Sachs, K., Nolan, G. P., & Plevritis, S. K. (2011). Extracting a cellular hierarchy from high-dimensional cytometry data with SPADE. In Nature Biotechnology

(Vol. 29, Issue 10, pp. 886–891). Springer Science and Business Media LLC. https://doi.org/10.1038/nbt.1991

11. Bellman, R. E. & Rand Corporation (1957). Dynamic programming. Princeton University Press (pp. ix.) ISBN 978-0-691-07951-6.

12. Bellman, R. E. (1961). Adaptive control processes: a guided tour. Princeton University Press. ISBN 9780691079011.

13. Orlova, D. Y., Herzenberg, L. A., & Walther, G. (2017). Science not art: statistically sound methods for identifying subsets in multi-dimensional flow and mass cytometry data sets. In Nature Reviews Immunology (Vol. 18, Issue 1, pp. 77–77). https://doi.org/10.1038/nri.2017.150

14. Meehan, S., Kolyagin, G. A., Parks, D., Youngyunpipatkul, J., Herzenberg, L. A., Walther, G., Ghosn, E. E. B., & Orlova, D. Y. (2019). Automated subset identification and characterization pipeline for multidimensional flow and mass cytometry data clustering and visualization. In Communications Biology (Vol. 2, Issue 1). https://doi.org/10.1038/s42003-019-0467-6

15. Jolliffe, I. T., & Cadima, J. (2016). Principal component analysis: a review and recent developments. In Philosophical Transactions of the Royal Society A: Mathematical, Physical and Engineering Sciences (Vol. 374, Issue 2065, p. 20150202). The Royal Society. https://doi.org/10.1098/rsta.2015.0202

16. Van der Maaten, L., & Hinton, J. (2008). Visualizing Data using t-SNE. In Journal of Machine Learning Research (Vol. 9, Issue 86, pp. 2579-2605). http://jmlr.org/papers/v9/vandermaaten08a.html.

17. McInnes, L., Healy, J., Saul, N., & Großberger, L. (2018). UMAP: Uniform Manifold Approximation and Projection. In Journal of Open Source Software (Vol. 3, Issue 29, p. 861). The Open Journal. https://doi.org/10.21105/joss.00861

18. Cook, D., Buja, A., Cabrera, J., & Hurley, C. (1995). Grand Tour and Projection Pursuit. In Journal of Computational and Graphical Statistics (Vol. 4, Issue 3, p. 155). JSTOR. https://doi.org/10.2307/1390844

19. McInnes, L. (2018). UMAP for Supervised Dimension Reduction and Metric Learning. https://umap-learn.readthedocs.io/en/latest/supervised.html.

20. Eddins, D. J., Yang, J., Kosters, A., Giacalone, V. D., Pechuan-Jorge, X., Chandler, J. D., Eum, J., Babcock, B. R., Dobosh, B. S., Hernández, M. R., Abdulkhader, F., Collins, G. L., Orlova, D. Y., Ramonell, R. P., Sanz, I., Moussion, C., Eun-Hyung Lee, F., Tirouvanziam, R. M., & Ghosn, E. E. B. (2023). Transcriptional

reprogramming of infiltrating neutrophils drives lung pathology in severe COVID-19 despite low viral load. In Blood Advances (Vol. 7, Issue 5, pp. 778–799). American Society of Hematology. https://doi.org/10.1182/bloodadvances.2022008834

21. Toghi Eshghi, S., Au-Yeung, A., Takahashi, C., Bolen, C. R., Nyachienga, M. N., Lear, S. P., Green, C., Mathews, W. R., & O'Gorman, W. E. (2019). Quantitative Comparison of Conventional and t-SNE-guided Gating Analyses. In Frontiers in Immunology (Vol. 10). https://doi.org/10.3389/fimmu.2019.01194

22. Zhou, Y., Song, W. M., Andhey, P. S., Swain, A., Levy, T., Miller, K. R., Poliani, P. L., Cominelli, M., Grover, S., Gilfillan, S., Cella, M., Ulland, T. K., Zaitsev, K., Miyashita, A., Ikeuchi, T., Sainouchi, M., Kakita, A., Bennett, D. A., Schneider, J. A., … Colonna, M. (2020). Human and mouse single-nucleus transcriptomics reveal TREM2-dependent and TREM2-independent cellular responses in Alzheimer's disease. In Nature Medicine (Vol. 26, Issue 1, pp. 131–142). https://doi.org/10.1038/s41591-019-0695-9

23. Tickotsky, N., Sagiv, T., Prilusky, J., Shifrut, E., & Friedman, N. (2017). McPAS-TCR: a manually curated catalogue of pathology-associated T cell receptor sequences. In J. Wren (Ed.), Bioinformatics (Vol. 33, Issue 18, pp. 2924–2929). Oxford University Press (OUP). https://doi.org/10.1093/bioinformatics/btx286

24. AutoGate's fully automated methods. https://cytogenie.org. Accessed Sep 15, 2024.

25. Satija, R., Satija Lab & Collaborators. Seurat- R toolkit for single cell genomics. https://satijalab.org/seurat/. Accessed Sep 15, 2024.

26. Satija, R., Satija Lab & Collaborators. Seurat - guided clustering tutorial. https://satijalab.org/seurat/articles/pbmc3k_tutorial.html. Accessed Sep 15, 2024.

27. Bankhead, P., Loughrey, M. B., Fernández, J. A., Dombrowski, Y., McArt, D. G., Dunne, P. D., McQuaid, S., Gray, R. T., Murray, L. J., Coleman, H. G., James, J. A., Salto-Tellez, M., & Hamilton, P. W. (2017). QuPath: Open source software for digital pathology image analysis. In Scientific Reports (Vol. 7, Issue 1). https://doi.org/10.1038/s41598-017-17204-5

28. Stringer, C., Wang, T., Michaelos, M., & Pachitariu, M. (2020). Cellpose: a generalist algorithm for cellular segmentation. In Nature Methods (Vol. 18, Issue 1, pp. 100–106). https://doi.org/10.1038/s41592-020-01018-x

29. Laboratory of Systems Pharmacology, Harvard Medical School. Multiple-choice microscopy pipeline. https://mcmicro.org. Accessed Sep 15, 2024.

30. Schapiro, D., Sokolov, A., Yapp, C., Chen, Y.-A., Muhlich, J. L., Hess, J., Creason, A. L., Nirmal, A. J., Baker, G. J., Nariya, M. K., Lin, J.-R., Maliga, Z., Jacobson, C. A., Hodgman, M. W., Ruokonen, J., Farhi, S. L., Abbondanza, D., McKinley, E. T., Persson, D., … Sorger, P. K. (2022). MCMICRO: a scalable, modular image-processing pipeline for multiplexed tissue imaging. In Nature Methods (Vol. 19, Issue 3, pp. 311–315). https://doi.org/10.1038/s41592-021-01308-y

31. Muhlich, J. L., Chen, Y.-A., Yapp, C., Russell, D., Santagata, S., & Sorger, P. K. (2022). Stitching and registering highly multiplexed whole-slide images of tissues and tumors using ASHLAR. In A. Valencia (Ed.), Bioinformatics (Vol. 38, Issue 19, pp. 4613–4621). Oxford University Press (OUP). https://doi.org/10.1093/bioinformatics/btac544

32. Greenwald, N. F., Miller, G., Moen, E., Kong, A., Kagel, A., Dougherty, T., Fullaway, C. C., McIntosh, B. J., Leow, K. X., Schwartz, M. S., Pavelchek, C., Cui, S., Camplisson, I., Bar-Tal, O., Singh, J., Fong, M., Chaudhry, G., Abraham, Z., Moseley, J., … Van Valen, D. (2021). Whole-cell segmentation of tissue images with human-level performance using large-scale data annotation and deep learning. In Nature Biotechnology (Vol. 40, Issue 4, pp. 555–565). https://doi.org/10.1038/s41587-021-01094-0

33. Di Tommaso, P., Chatzou, M., Floden, E. W., Barja, P. P., Palumbo, E., & Notredame, C. (2017). Nextflow enables reproducible computational workflows. In Nature Biotechnology (Vol. 35, Issue 4, pp. 316–319). https://doi.org/10.1038/nbt.3820

34. Lin, Z., Akin, H., Rao, R., Hie, B., Zhu, Z., Lu, W., Smetanin, N., Verkuil, R., Kabeli, O., Shmueli, Y., dos Santos Costa, A., Fazel-Zarandi, M., Sercu, T., Candido, S., & Rives, A. (2022). Evolutionary-scale prediction of atomic level protein structure with a language model. Cold Spring Harbor Laboratory. https://doi.org/10.1101/2022.07.20.500902

35. The Biopython Contributors. Bio.pairwise2 module. https://biopython.org/docs/1.75/api/Bio.pairwise2.html. Accessed Sep 15, 2024.

36. Dunbar, J., & Deane, C. M. (2015). ANARCI: antigen receptor numbering and receptor classification. In Bioinformatics (Vol. 32, Issue 2, pp. 298–300). Oxford University Press (OUP). https://doi.org/10.1093/bioinformatics/btv552

37. PDBePISA (Proteins, Interfaces, Structures and Assemblies). http://www.ebi.ac.uk/pdbe/prot_int/pistart.html . Accessed Sep 15, 2024.

38. Krissinel, E., & Henrick, K. (2007). Inference of Macromolecular Assemblies from Crystalline State. In Journal of Molecular Biology (Vol. 372, Issue 3, pp. 774–797). https://doi.org/10.1016/j.jmb.2007.05.022

39. Calinski, T., & Harabasz, J. (1974). A dendrite method for cluster analysis. In Communications in Statistics - Theory and Methods (Vol. 3, Issue 1, pp. 1–27). https://doi.org/10.1080/03610927408827101

40. Mann, H. B. and Wald, A. (1942) On the Choice of the Number of Class Intervals in the Application of Chi-Square Test. In Annals of Mathematical Statistics (Vol. 13, Issue 3, pp. 306-317).

41. McInnes, L. UMAP. https://github.com/lmcinnes/umap/issues/415. Accessed Sep 15, 2024.

42. Ben-Hur, A. et al. (2001). Support Vector Clustering. In Journal of Machine Learning Research (Vol. 2, pp. 125-137).

43. Orlova, D. Y., Meehan, S., Parks, D., Moore, W. A., Meehan, C., Zhao, Q., Ghosn, E. E. B., Herzenberg, L. A., & Walther, G. (2018). QFMatch: multidimensional flow and mass cytometry samples alignment. In Scientific Reports (Vol. 8, Issue 1). https://doi.org/10.1038/s41598-018-21444-4

44. Zimmerman, N. (2011). A computational approach to identification and comparison of cell subsets in flow cytometry data. Ph.D. Thesis, Stanford University. Available: https://stacks.stanford.edu/file/druid:hg137hq6178/Zimmerman-Dissertation-v2-augmented.pdf.

45. McInnes, L. HDBSCAN. Version: 0.8.33. https://pypi.org/project/hdbscan/. Accessed Sep 15, 2024.

46. Scikit-learn developers. KMeans. Version: 1.0.2. https://scikit-learn.org/stable/modules/generated/sklearn.cluster.KMeans.html. Accessed Sep 15, 2024.

47. Levine, J. PhenoGraph for Python3. PhenoGraph 1.5.3. https://pypi.org/project/PhenoGraph. Accessed Sep 15, 2024.

48. Quintelier, K., Couckuyt, A., Emmaneel, A., Aerts, J., Saeys, Y., Van Gassen, S. (2011). Analyzing high-dimensional cytometry data using FlowSOM. In Nature Protocols. (Vol. 16, Issue 8, pp. 3775–3801).https://doi.org/10.1038/s41596-021-00550-0

49. Aghaeepour, N., Chattopadhyay, P., Chikina, M., Dhaene, T., Van Gassen, S., Kursa, M., Lambrecht, B. N., Malek, M., McLachlan, G. J., Qian, Y., Qiu, P., Saeys, Y., Stanton, R., Tong, D., Vens, C., Walkowiak, S., Wang, K., Finak, G., Gottardo, R., … Brinkman, R. R. (2015). A benchmark for evaluation of algorithms for identification of cellular correlates of clinical outcomes. In Cytometry Part A (Vol. 89, Issue 1, pp. 16–21). Wiley. https://doi.org/10.1002/cyto.a.22732

50. Xu, M. M., Pu, Y., Han, D., Shi, Y., Cao, X., Liang, H., Chen, X., Li, X.-D., Deng, L., Chen, Z. J., Weichselbaum, R. R., & Fu, Y.-X. (2017). Dendritic Cells but Not Macrophages Sense Tumor Mitochondrial DNA for Cross-priming through Signal Regulatory Protein α Signaling. In Immunity (Vol. 47, Issue 2, pp. 363-373.e5). https://doi.org/10.1016/j.immuni.2017.07.016

51. Dutertre, C.-A., Becht, E., Irac, S. E., Khalilnezhad, A., Narang, V., Khalilnezhad, S., Ng, P. Y., van den Hoogen, L. L., Leong, J. Y., Lee, B., Chevrier, M., Zhang, X. M., Yong, P. J. A., Koh, G., Lum, J., Howland, S. W., Mok, E., Chen, J., Larbi, A., … Ginhoux, F. (2019). Single-Cell Analysis of Human Mononuclear Phagocytes Reveals Subset-Defining Markers and Identifies Circulating Inflammatory Dendritic Cells. In Immunity (Vol. 51, Issue 3, pp. 573-589.e8). https://doi.org/10.1016/j.immuni.2019.08.008

52. Bourdely, P., Anselmi, G., Vaivode, K., Ramos, R. N., Missolo-Koussou, Y., Hidalgo, S., Tosselo, J., Nuñez, N., Richer, W., Vincent-Salomon, A., Saxena, A., Wood, K., Lladser, A., Piaggio, E., Helft, J., & Guermonprez, P. (2020). Transcriptional and Functional Analysis of CD1c+ Human Dendritic Cells Identifies a CD163+ Subset Priming CD8+CD103+ T Cells. In Immunity (Vol. 53, Issue 2, pp. 335-352.e8). https://doi.org/10.1016/j.immuni.2020.06.002

53. Comi, M., Avancini, D., Santoni de Sio, F., Villa, M., Uyeda, M. J., Floris, M., Tomasoni, D., Bulfone, A., Roncarolo, M. G., & Gregori, S. (2019). Coexpression of CD163 and CD141 identifies human circulating IL-10-producing dendritic cells (DC-10). In Cellular & Molecular Immunology (Vol. 17, Issue 1, pp. 95–107). https://doi.org/10.1038/s41423-019-0218-0

54. Sedighzadeh, S. S., Khoshbin, A. P., Razi, S., Keshavarz-Fathi, M., & Rezaei, N. (2021). A narrative review of tumor-associated macrophages in lung cancer: regulation of macrophage polarization and therapeutic implications. In Translational Lung Cancer Research (Vol. 10, Issue 4, pp. 1889–1916). https://doi.org/10.21037/tlcr-20-1241

55. Laviron, M., Petit, M., Weber-Delacroix, E., Combes, A. J., Arkal, A. R., Barthélémy, S., Courau, T., Hume, D. A., Combadière, C., Krummel, M. F., & Boissonnas, A. (2022). Tumor-associated macrophage heterogeneity is driven by tissue

territories in breast cancer. In Cell Reports (Vol. 39, Issue 8, p. 110865). https://doi.org/10.1016/j.celrep.2022.110865

56. Miura, Y., Anami, T., Yatsuda, J., Motoshima, T., Oka, S., Suyama, K., Inoshita, N., Kinowaki, K., Urakami, S., Kamba, T., & Komohara, Y. (2021). HLA-DR and CD74 Expression and the Immune Microenvironment in Renal Cell Carcinoma. In Anticancer Research (Vol. 41, Issue 6, pp. 2841–2848). https://doi.org/10.21873/anticanres.15065

57. Senosain, M.-F., Zou, Y., Novitskaya, T., Vasiukov, G., Balar, A. B., Rowe, D. J., Doxie, D. B., Lehman, J. M., Eisenberg, R., Maldonado, F., Zijlstra, A., Novitskiy, S. V., Irish, J. M., & Massion, P. P. (2021). HLA-DR cancer cells expression correlates with T cell infiltration and is enriched in lung adenocarcinoma with indolent behavior. In Scientific Reports (Vol. 11, Issue 1). https://doi.org/10.1038/s41598-021-93807-3

58. Madani, A., Krause, B., Greene, E. R., Subramanian, S., Mohr, B. P., Holton, J. M., Olmos, J. L., Jr., Xiong, C., Sun, Z. Z., Socher, R., Fraser, J. S., & Naik, N. (2023). Large language models generate functional protein sequences across diverse families. In Nature Biotechnology (Vol. 41, Issue 8, pp. 1099–1106). https://doi.org/10.1038/s41587-022-01618-2

59. Chandra, A., Tünnermann, L., Löfstedt, T., & Gratz, R. (2023). Transformer-based deep learning for predicting protein properties in the life sciences. In eLife (Vol. 12). https://doi.org/10.7554/elife.82819

60. Ruffolo, J. A., & Madani, A. (2024). Designing proteins with language models. In Nature Biotechnology (Vol. 42, Issue 2, pp. 200–202). https://doi.org/10.1038/s41587-024-02123-4

61. Quintana, F., Treangen, T., & Kavraki, L. (2023). Leveraging Large Language Models for Predicting Microbial Virulence from Protein Structure and Sequence. In Proceedings of the 14th ACM International Conference on Bioinformatics, Computational Biology, and Health Informatics. BCB ’23: 14th ACM International Conference on Bioinformatics, Computational Biology, and Health Informatics. ACM. https://doi.org/10.1145/3584371.3612953

62. Leem, J., Mitchell, L. S., Farmery, J. H. R., Barton, J., & Galson, J. D. (2022). Deciphering the language of antibodies using self-supervised learning. In Patterns (Vol. 3, Issue 7, p. 100513). https://doi.org/10.1016/j.patter.2022.100513

63. Wu, K., Yost, K. E., Daniel, B., Belk, J. A., Xia, Y., Egawa, T., Satpathy, A., Chang, H. Y., & Zou, J. (2021). TCR-BERT: learning the grammar of T-cell receptors for flexible antigen-xbinding analyses. Cold Spring Harbor Laboratory. https://doi.org/10.1101/2021.11.18.469186

64. Culka, M., & Orlova, D. (2024). Tricked by Edge Cases: Can Current Approaches Lead to Accurate Prediction of T-Cell Specificity with Machine Learning? Cold Spring Harbor Laboratory. https://doi.org/10.1101/2024.10.23.619492

65. Kruskal, J. B. (1969). Toward a practical method which helps uncover the structure of a set of observations by finding the line transformation which optimizes a new “index of condensation.” Milton, RC, & Nelder, JA (eds), Statistical computation; New York, Academic Press (pp. 427–440).

66. Papalegis, D. (2024). Human squamous cell carcinoma stained with SignalStar mIHC technology. Mendeley Data, V1, doi: 10.17632/5vfz9vhm2s.1

67. Simpson C, Tabatsky E, Rahil Z, et al. Supporting data for "Lifting the curse from high-dimensional data: Automated projection pursuit clustering for the variety of biological data modalities" GigaScience Database. 2025. https://doi.org/10.5524/102687

Main Text Figures

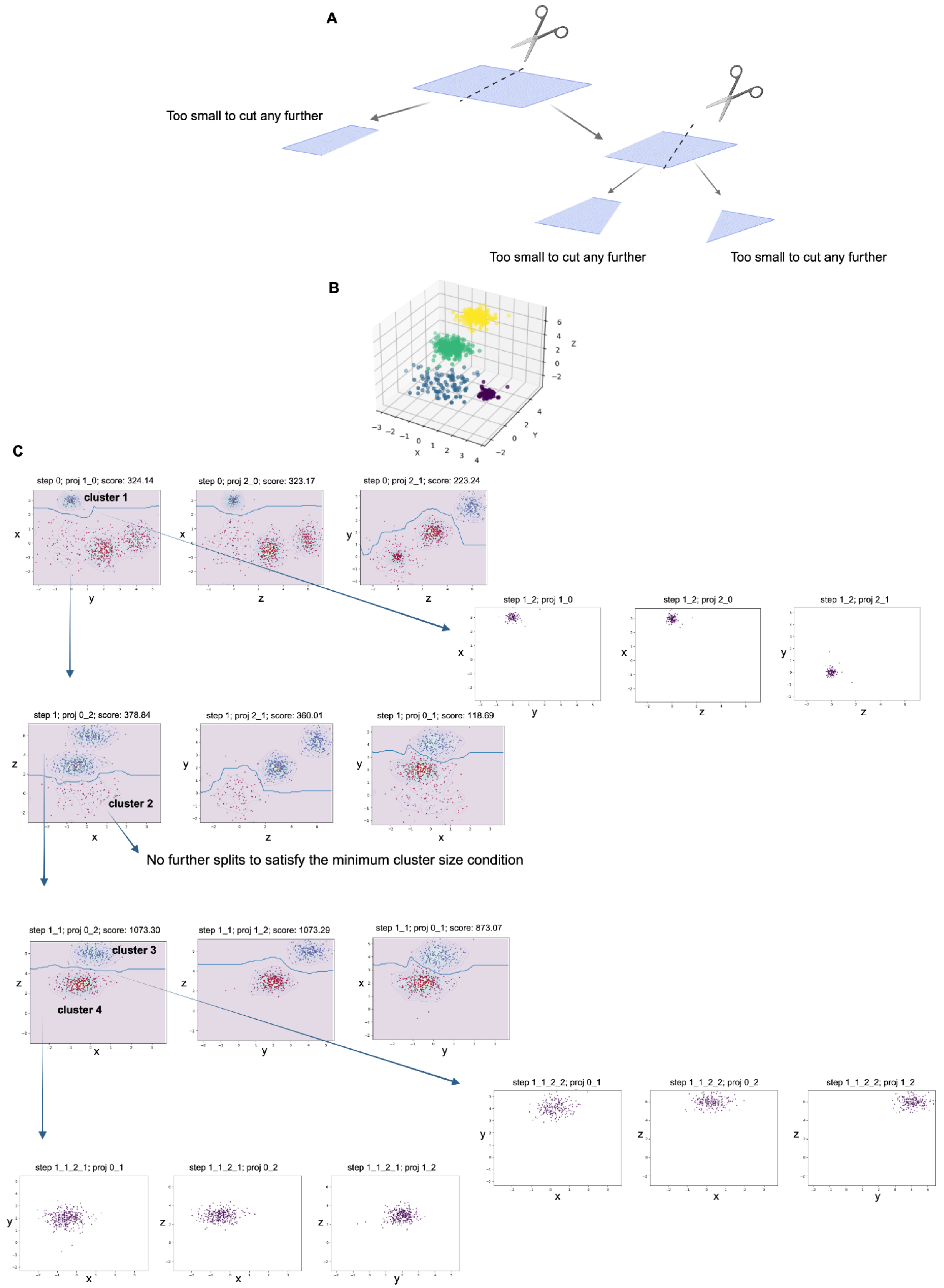
A
Too small to cut any further
Too small to cut any further
Too small to cut any further
B
C
step 0; proj 1_0; score: 324.14
cluster 1
step 0; proj 2_0; score: 323.17
step 0; proj 2_1; score: 223.24
step 1_2; proj 1_0
step 1_2; proj 2_0
step 1_2; proj 2_1
step 1; proj 0_2; score: 378.84
cluster 2
step 1; proj 2_1; score: 360.01
step 1; proj 0_1; score: 118.69
No further splits to satisfy the minimum cluster size condition
step 1_1; proj 0_2; score: 1073.30
cluster 3
cluster 4
step 1_1; proj 1_2; score: 1073.29
step 1_1; proj 0_1; score: 873.07
step 1_1_2_2; proj 0_1
step 1_1_2_2; proj 0_2
step 1_1_2_2; proj 1_2
step 1_1_2_1; proj 0_1
step 1_1_2_1; proj 0_2
step 1_1_2_1; proj 1_2

Figure 1. Automated projection pursuit clustering workflow. A. Metaphorical representation of projection pursuit clustering, where the paper sheet represents multidimensional data projected into a pair of dimensions with the best separation (all other projections are not shown). Scissors represent the process of splitting the data into segments along a decision boundary (dotted line). This step reflects the idea of sequentially finding the most informative projections to separate the data. **B.** To illustrate the concept behind the APP algorithm in a simplified manner, we used a three-dimensional synthetic dataset. **C.** The APP algorithm systematically explores orthogonal two-dimensional projections, selects the one with the smallest density distribution along the decision boundary (represented by a blue line on the two-dimensional projections), and recursively splits the data until the defined stop criteria are met. This approach helps uncover meaningful patterns and structures in the data. For simplicity, projections with interchanged axes are not displayed (e.g., only the xy projection is shown instead of both xy and yx).

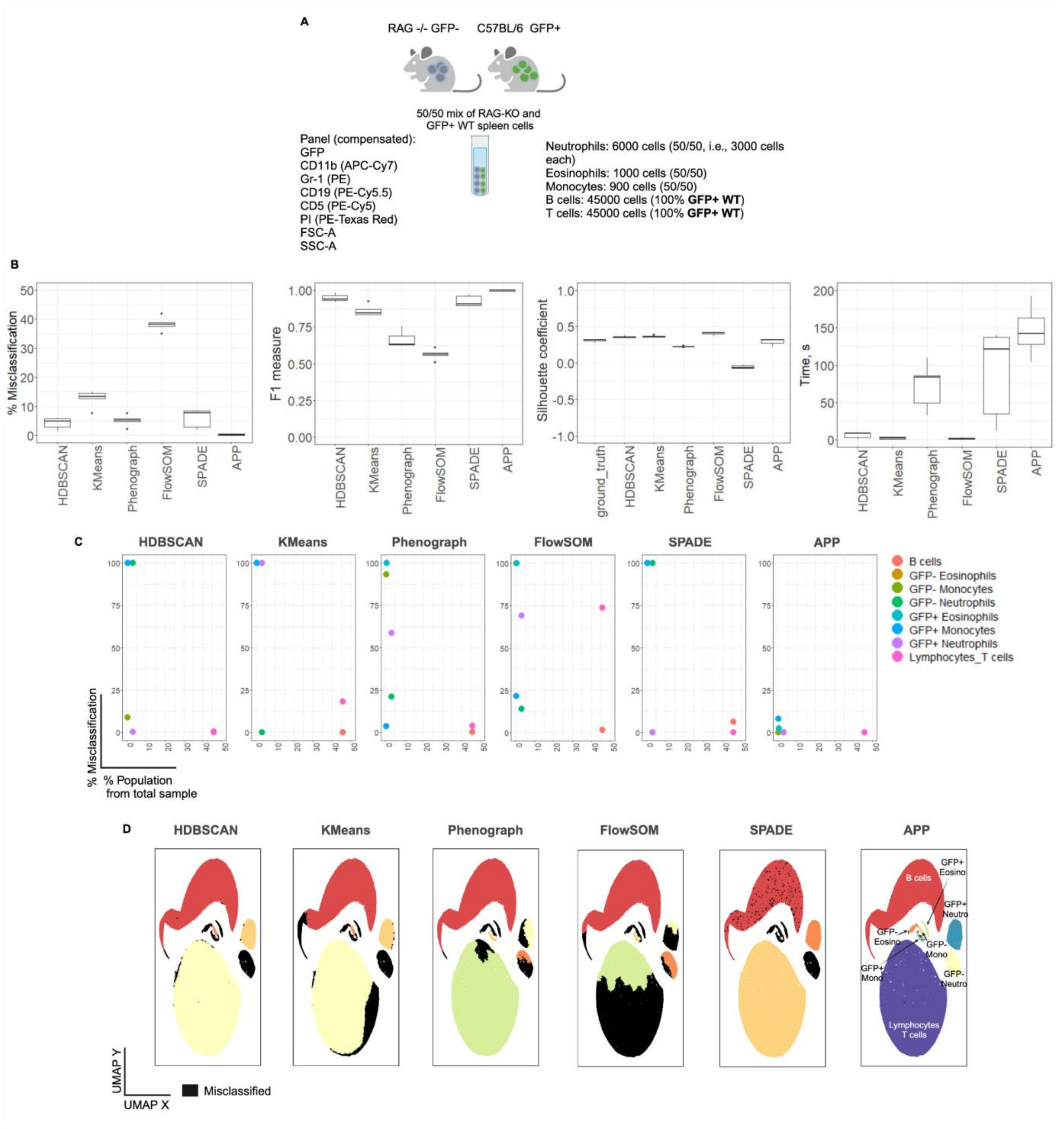

Figure 2. APP outperforms widely used clustering methods in application to the representative biological dataset that has functionally validated ground truth labels. A. A representative ground truth sample, selected from several similar types of samples, was generated by mixing cells in equal proportions from the GFP+ Wild-type spleen and RAG-KO spleen. It's important to note that RAG-KO mice lack B and T cells. **B.** The performance of the APP algorithm in cell population classification, evaluated

through total misclassification, F1-measure, and Silhouette coefficient, is being assessed and compared to state-of-the-art clustering algorithms. The evaluation encompasses the blending of wild-type spleen cells and RAG-KO spleen cells at five different proportions (see the RAG-KO immune dataset for more details). Clustering time was evaluated on a laptop equipped with an 11th Gen Intel® Core™ i7-1165G7 @ 2.80GHz processor and 32.0 GB of RAM, using datasets ranging from ~27k to 98k cells with a dimensionality of 7. We used the default input parameters recommended for each clustering algorithm based on the available tutorials. **C.** Per-cell-type population misclassification assessment, with cell populations ordered from least to most abundant (left to right). **D.** The misclassification for the 50/50 mix is visually represented in black color using the automated label transfer pipeline, as detailed in Supplementary Figure 4. Here, the performance of each of the six clustering algorithms is assessed against the ground truth labels (functionally distinct cell types) used to construct the supervised UMAP embedding.

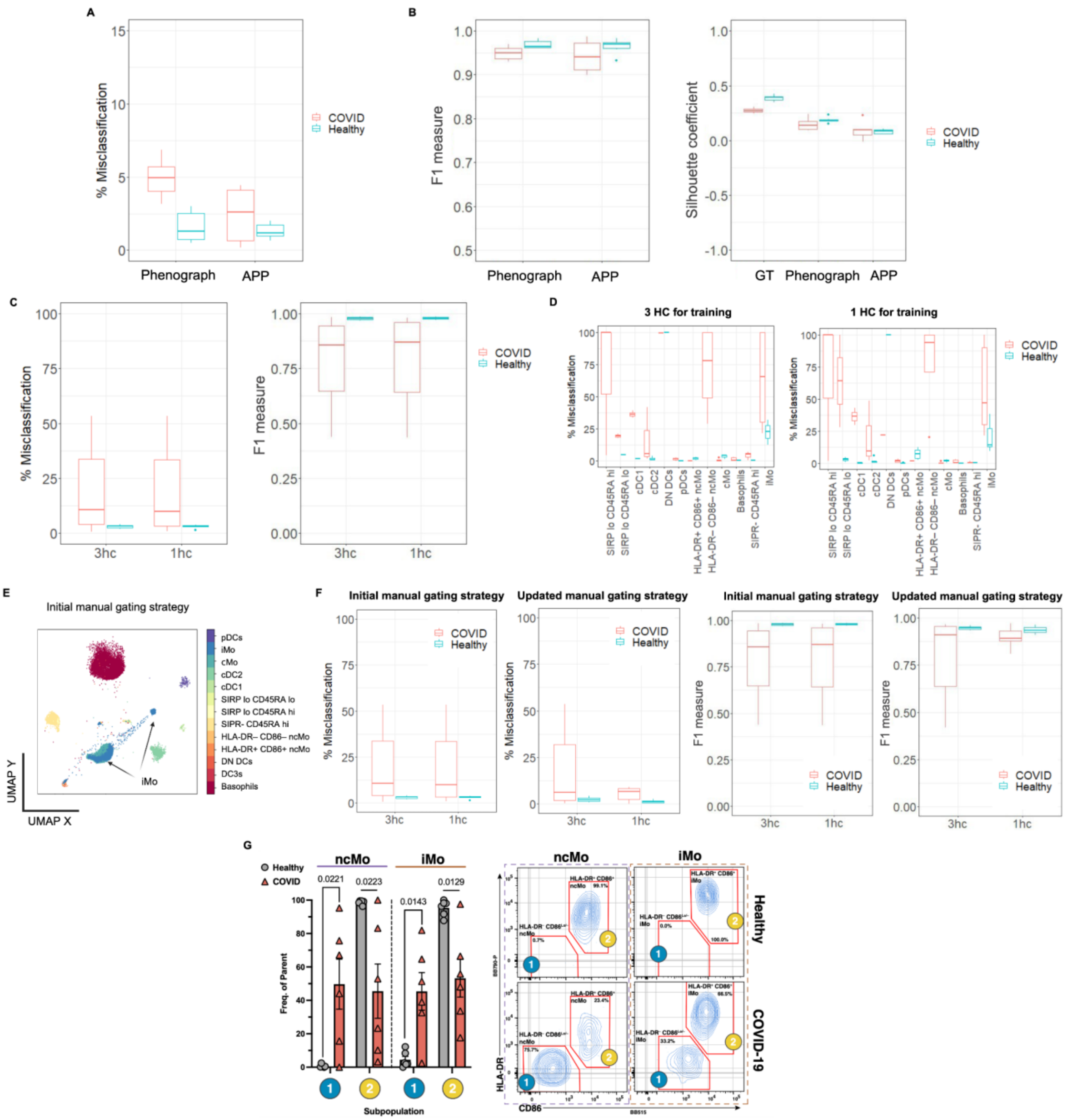

Figure 3. The label transfer pipeline reveals distinct cellular responses in COVID-19 patients and achieves over 99 percent accuracy in automating the manual gating strategy for healthy control samples, using minimal training data. A. On average, less than three percent of the data were misclassified when comparing APP clustering decisions to the manual ground truth labels. **B.** Comparison of F1-measure and Silhouette coefficient between Phenograph and APP. "GT" refers to ground truth.

C. The automation of the gating strategy application, implemented via the automated label transfer pipeline, achieves very high accuracy for healthy control (HC or hc) samples. **D.** Misclassification evaluation on a per-cell-population basis allows the identification of populations that are most problematic for the automated gating strategy transfer, providing targeted insights for improvement. Cell populations are ordered from most abundant to least abundant (left to right). **E.** The label transfer pipeline facilitates the observation of agreement or disagreement between data topology and ground truth cluster labels. It provides insights into the accuracy of ground truth labels and serves as a quality control step. Detected discrepancies (e.g., iMo cell population is more heterogeneous than defined by the original manual gating strategy) may prompt reevaluation or refinement of annotations (**F**), resulting in the discovery of biologically meaningful cell populations (**G**, HLA-DR- CD86lo ncMo and iMo populations that are unique to COVID patients). Minimum cluster size and bin size input parameters of 100 and 50, respectively, were used for both APP and the label transfer pipeline to generate the results presented in panels A-G.

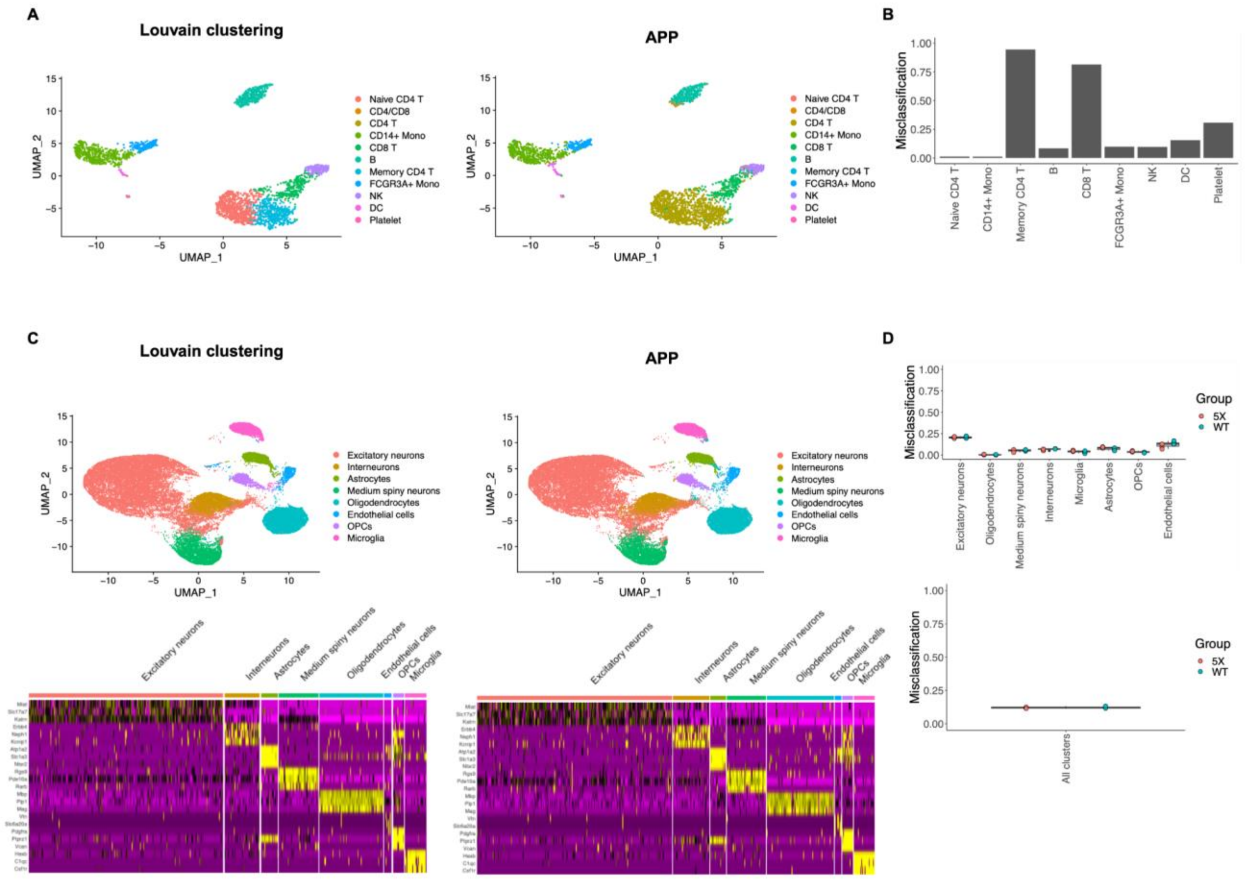

Figure 4. Both Louvain clustering and APP clustering, applied to PCA-reduced scRNAseq data, exhibit good alignment with each other. The overall misclassification rate between Louvain and APP clustering decisions in the PBMCs dataset is about 29 percent . The misclassification rates are determined with reference to the "ground truth" clusters, indicating how many cells in each ground truth cluster produced by Louvain were marked as misclassified. APP clustering encounters challenges in distinguishing between CD4 Naive and Memory cells in the dataset (**A,B**). The Silhouette Coefficient is 0.28 for Louvain and 0.23 for APP clustering outcomes. **C.** There is a high degree of concordance (approximately 87 percent) between Louvain and APP clustering when applied to pooled combinations of wildtype (WT) and Alzheimer Disease model (5X) mice samples. The Silhouette Coefficient is 0.3 for Louvain and 0.2 for APP clustering outcomes. Heatmaps illustrate the concordance of gene expression patterns in each cluster type as identified by the two clustering

algorithms. Panel **D** presents per cell type misclassification, providing insights into specific cell types where misclassification occurs.

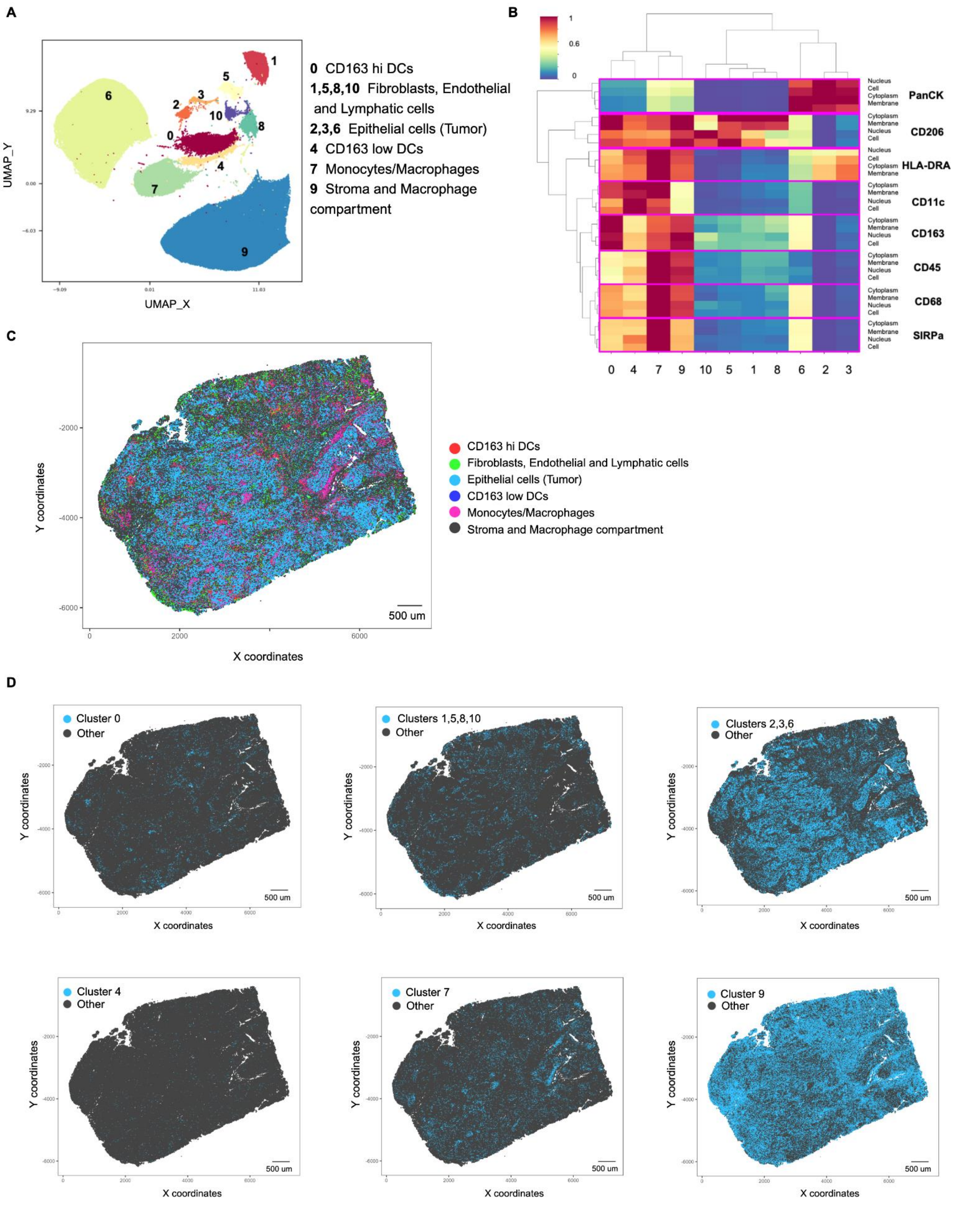

Figure 5. APP clustering successfully leverages multiplexed imaging data to gain insights into biologically meaningful cell populations within the complex tissue

context of a human squamous lung carcinoma sample. Eleven cell clusters detected by APP (**A**) were annotated based on the pattern of median marker expression (**B**) and their spatial location (**C,D**). XY Cartesian coordinates represent the position of a tissue sample on a glass slide. The 0–1 scale in panel B represents normalized expression, with each row normalized independently.

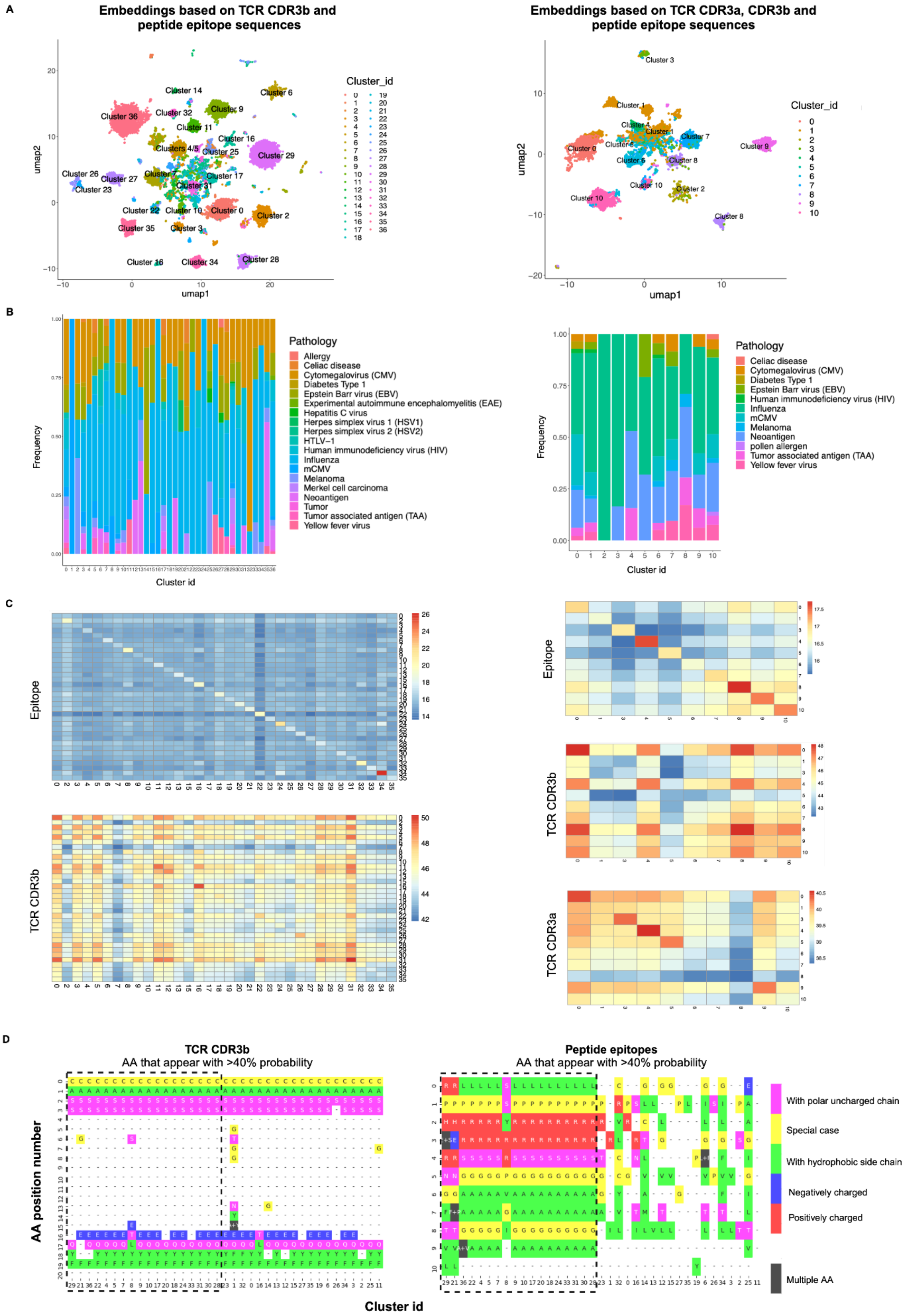
A
Embeddings based on TCR CDR3b and peptide epitope sequences
Embeddings based on TCR CDR3a, CDR3b and peptide epitope sequences
Cluster_id
umap1
umap2
B
Pathology
Allergy
Celiac disease
Cytomegalovirus (CMV)
Diabetes Type 1
Epstein Barr virus (EBV)
Experimental autoimmune encephalomyelitis (EAE)
Hepatitis C virus
Herpes simplex virus 1 (HSV1)
Herpes simplex virus 2 (HSV2)
HTLV−1
Human immunodeficiency virus (HIV)
Influenza
mCMV
Melanoma
Merkel cell carcinoma
Neoantigen
Tumor
Tumor associated antigen (TAA)
Yellow fever virus
pollen allergen
Frequency
Cluster id
C
Epitope
TCR CDR3b
TCR CDR3a
D
TCR CDR3b
AA that appear with >40% probability
Peptide epitopes
AA that appear with >40% probability
AA position number
Cluster id
With polar uncharged chain
Special case
With hydrophobic side chain
Negatively charged
Positively charged
Multiple AA

Figure 6. There is no identifiable common binding motif or a universal amino acid R group attribute characterizing the pMHC-TCR interaction. The clusters annotated by APP (**A**) are, in most cases, heterogeneous in terms of associated pathology, but some clusters are disproportionately enriched for particular pathologies, such as Epstein-Barr virus in cluster 14 or cytomegalovirus in cluster 32 in the clustering based on CDR3b and epitope sequences (**B**). Half of the pathologies in this dataset are associated with multiple unique epitope sequences, with an average of 8 unique epitope sequences per pathology. When sequence similarity scores are calculated for each pair of unique epitope sequences in the dataset and those scores are averaged across each unique pair within a cluster and and across each unique pair between all pairwise clusters, average similarity scores are systematically higher within clusters—on the diagonal line in these heatmaps—than between clusters. The same pattern is observed for CDR3b and CDR3a sequences to a lesser degree (**C**). Note that Cluster 2 from the CDR3b, CDR3a, and epitope clustering was removed from the epitope and CDR3a heatmaps because it contained < 20 unique epitope sequences (all other clusters had between 41-125 unique epitope sequences), causing it to have average within-cluster scores high enough to visually overwhelm the heatmaps, so as to allow the patterns between other clusters to be more perceptible, and clusters 1, 14, and 36 were removed from the CDR4b and epitope clustering because their average within-cluster scores were also visually distracting outliers. Per cluster amino acid enrichment analysis (**D**) revealed that, while there is no singular characteristic universally defining TCR-pMHC interactions, specific clusters may demonstrate shared electrostatic patterns involving charged amino acids at the interface. A noteworthy example includes a set of clusters (highlighted with a dashed line) enriched with the LPRRSGAAGA peptide, characterized by positively charged amino acids.

Supplementary materials to

Lifting the curse from high dimensional data: Automated projection pursuit clustering for the variety of biological data modalities

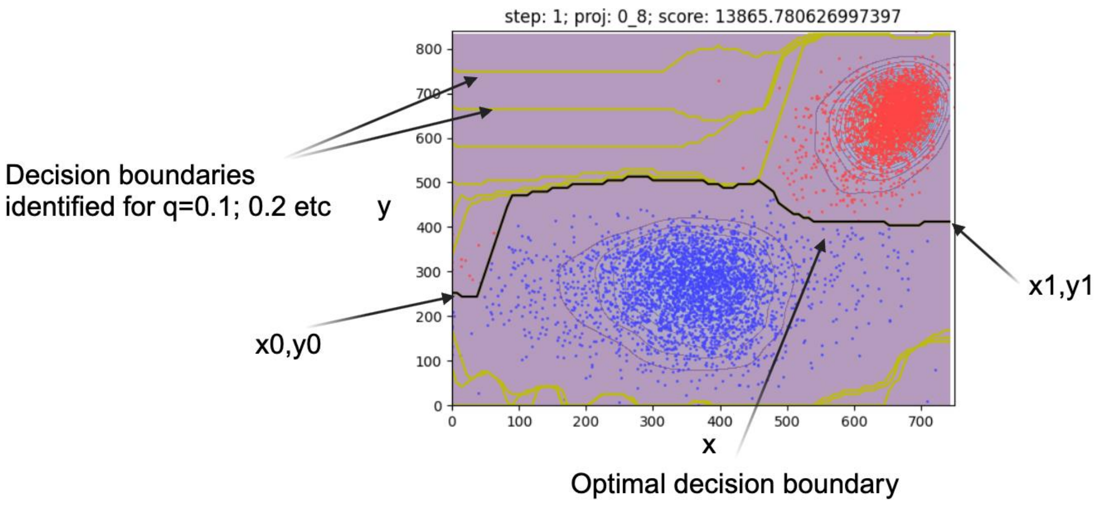

Supplementary Figure 1. Example of an optimal decision boundary search for one of the 2D data projections. The decision boundary begins at the left edge and ends at the right edge of a 2D projection. However, in xy vs yx orientations, the left and right edges differ, leading to distinct decision boundaries. Therefore, xy and yx projections should be analyzed independently.

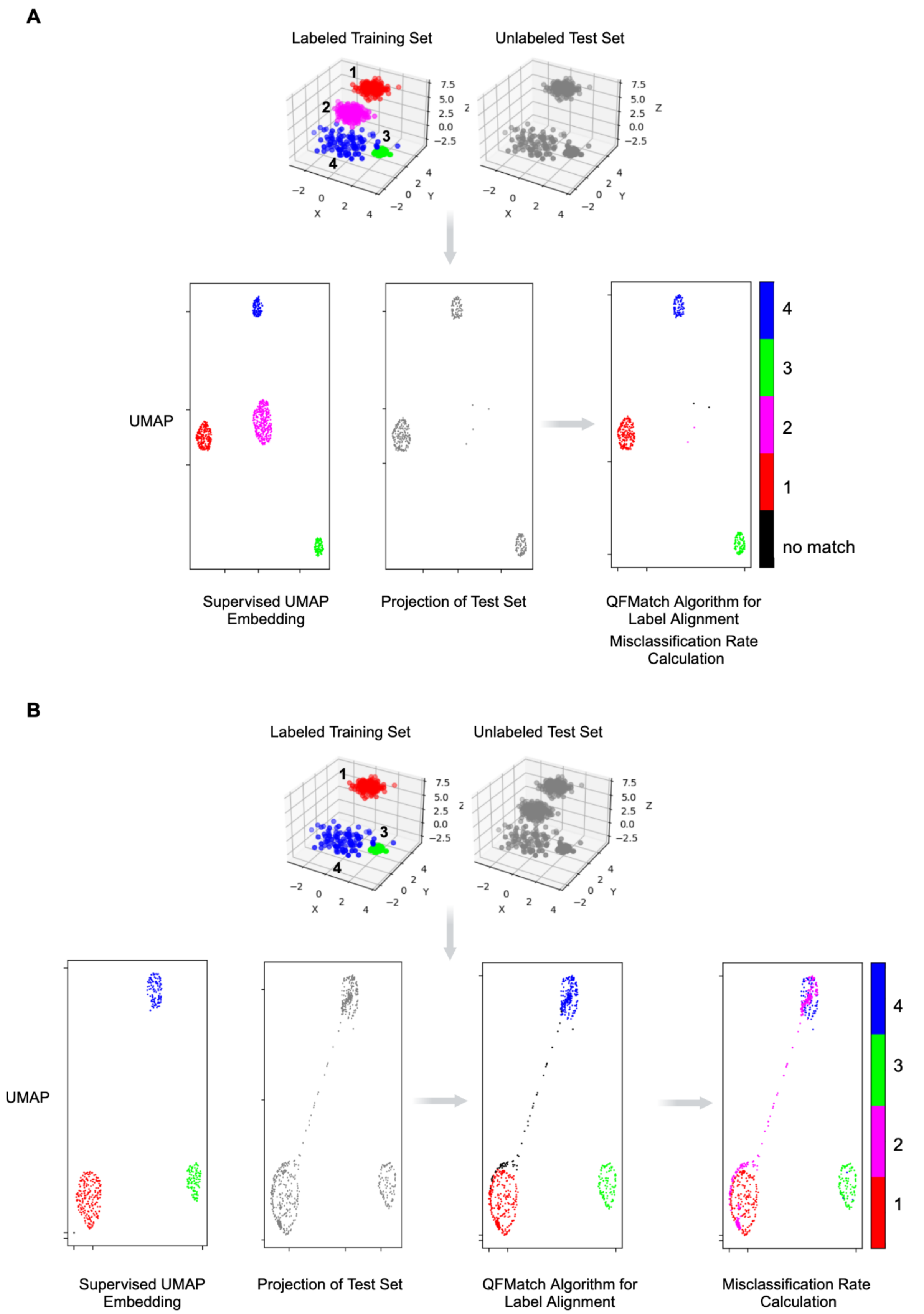
A
Labeled Training Set
Unlabeled Test Set
UMAP
Supervised UMAP Embedding
Projection of Test Set
QFMatch Algorithm for Label Alignment
Misclassification Rate Calculation
4
3
2
1
no match
B
Labeled Training Set
Unlabeled Test Set
UMAP
Supervised UMAP Embedding
Projection of Test Set
QFMatch Algorithm for Label Alignment
Misclassification Rate Calculation
4
3
2
1

Supplementary Figure 2. Workflow for the automated label transfer across samples. This pipeline utilizes labeled or partially labeled training samples with the marker expression data and ground truth cluster labels. It applies UMAP to learn a distance metric that optimally separates classes while preserving relationships in the marker space. The unlabeled test set is then projected into the UMAP embedding space built using the training set. The QFMatch algorithm aligns cluster labels between the test set and the training set for the downstream calculation for the number of misclassified events per cluster ID. Label transfer pipeline allows quantitatively comparing and aligning cluster labels across training and test samples in cases where clusters may be absent in either the test (**A**) or training data (**B**).

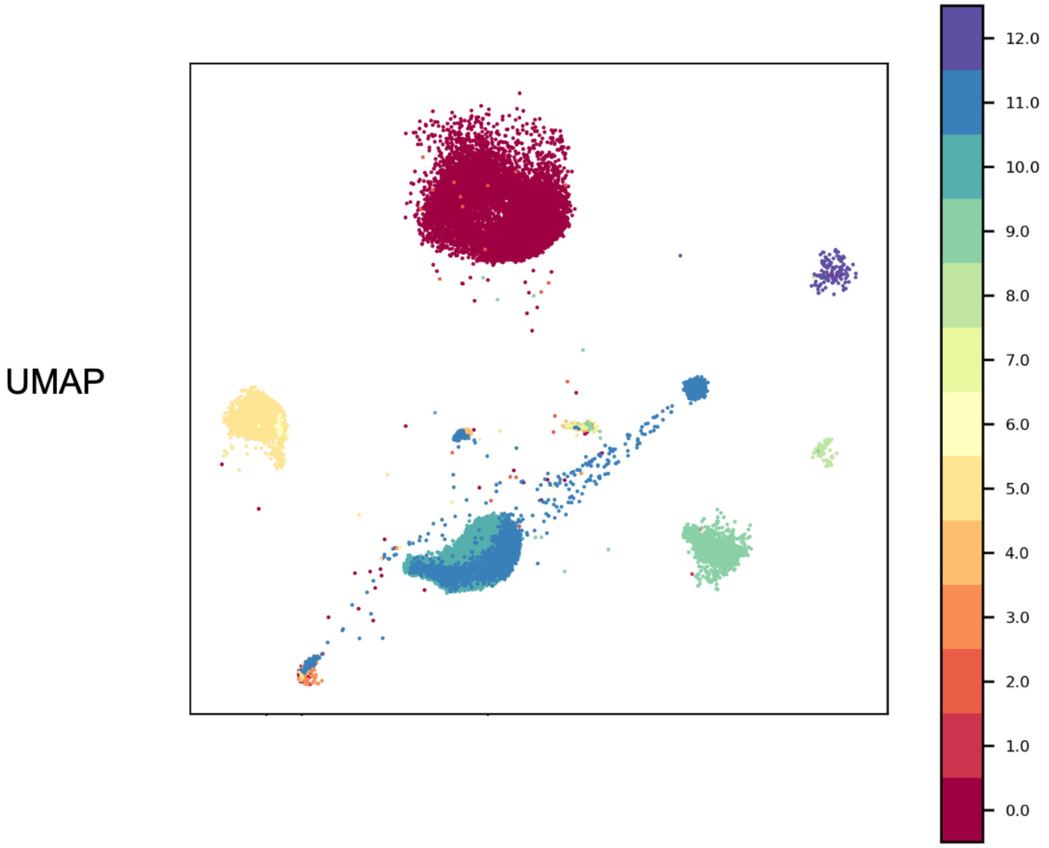

Supplementary Figure 3. An example of a discrepancy between the data topology and clustering decisions. Data topology suggests cluster 11 is more heterogeneous than was originally defined by the clustering approach (manual gating, in this case).

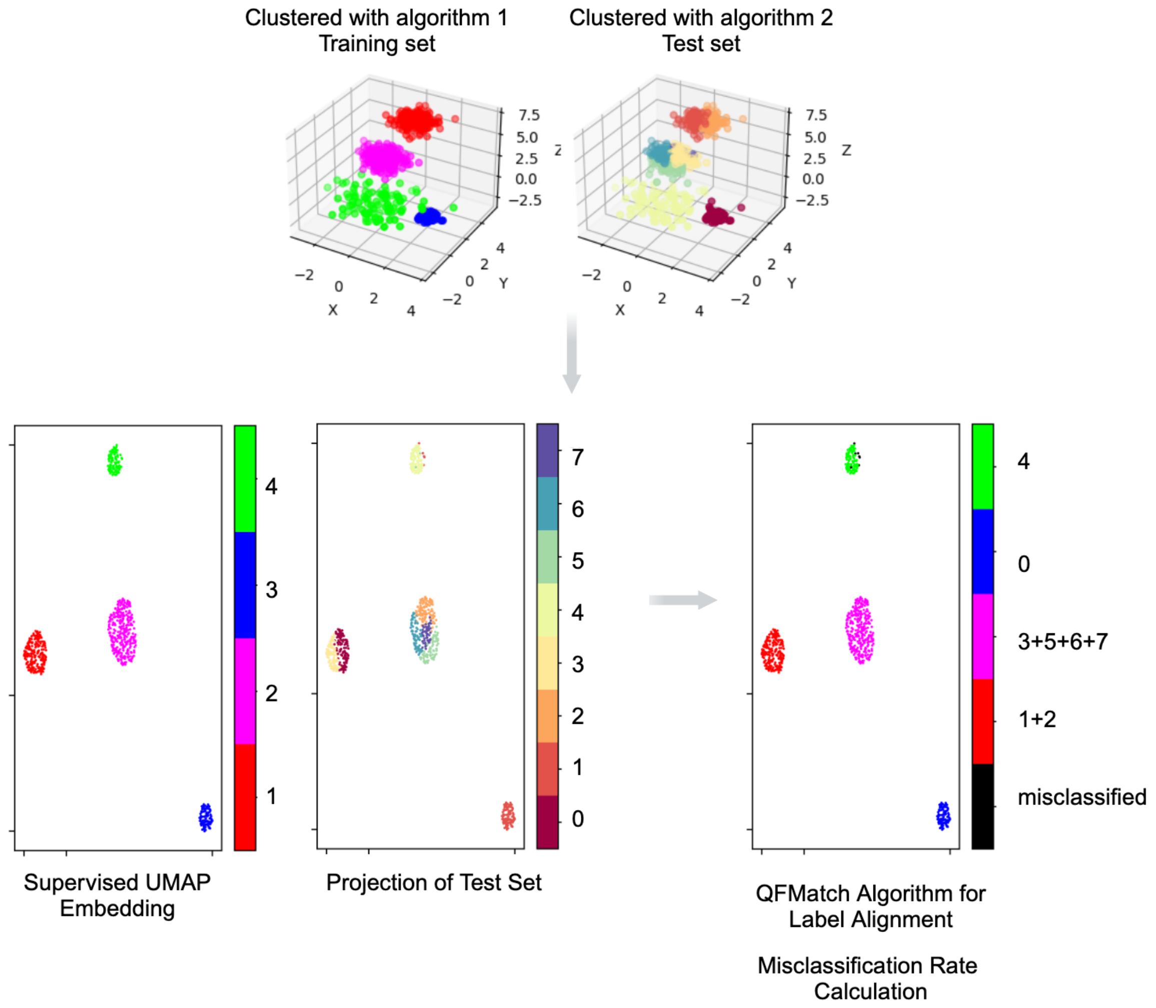

Supplementary Figure 4. Schematic representation of the label transfer pipeline application to quantitative comparison of two clustering algorithms decisions made on the same data set. We used a synthetic dataset consisting of a mixture of Gaussian distributions.

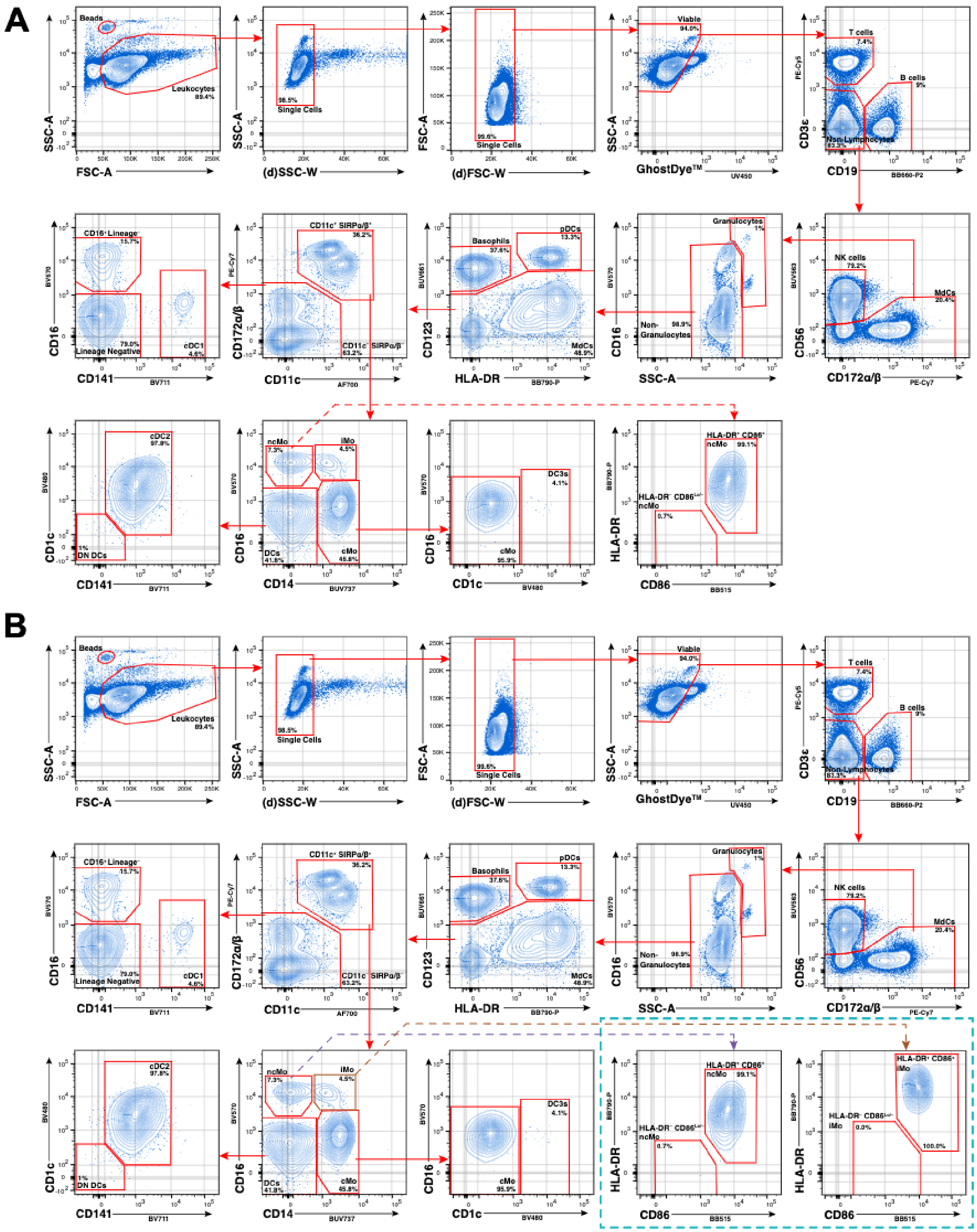
A
B
Beads
Leukocytes
89.4%
Single Cells
98.5%
99.6%
Viable
94.0%
T cells
7.4%
B cells
9%
Non-Lymphocytes
83.3%
SSC-A
FSC-A
(d)SSC-W
(d)FSC-W
GhostDye™
UV450
CD3ε
PE-Cy5
CD19
BB660-P2
CD16+ Lineage
15.7%
Lineage Negative
79.0%
cDC1
4.6%
CD11c+ SIRPα/β+
36.2%
CD11c− SIRPα/β−
63.2%
Basophils
37.6%
pDCs
13.3%
MdCs
48.9%
Granulocytes
1%
Non-Granulocytes
98.9%
NK cells
79.2%
MdCs
20.4%
CD16
BV570
CD141
BV711
CD172α/β
PE-Cy7
CD11c
AF700
CD123
BUV661
HLA-DR
BB790-P
SSC-A
CD56
BUV563
cDC2
97.8%
DN DCs
1%
ncMo
7.3%
iMo
4.5%
DCs
41.8%
cMo
45.8%
DC3s
4.1%
cMo
95.9%
HLA-DR+ CD86+
ncMo
99.1%
HLA-DR− CD86Lo/−
ncMo
0.7%
HLA-DR+ CD86+
iMo
100.0%
HLA-DR− CD86Lo/−
iMo
0.0%
CD1c
BV480
CD14
BUV737
CD86
BB515

Supplementary Figure 5. Expert-defined manual gating strategies used for high-dimensional flow cytometry data. A. Representative gating strategy of a randomly selected health donor sample to interrogate myeloid-derived cells (MdCs) enriched from PBMCs (see methods). **B.** Original gating strategy from (A) was refined following the label transfer pipeline “sanity check”, revealing an intermediate monocyte (iMo) population in COVID-19 patients that was not ubiquitously present in healthy donors, similar to non-classical monocytes (ncMo; see blue box & Figure 3G). This figure was created using FlowJo™ v10.8 (BD Biosciences).

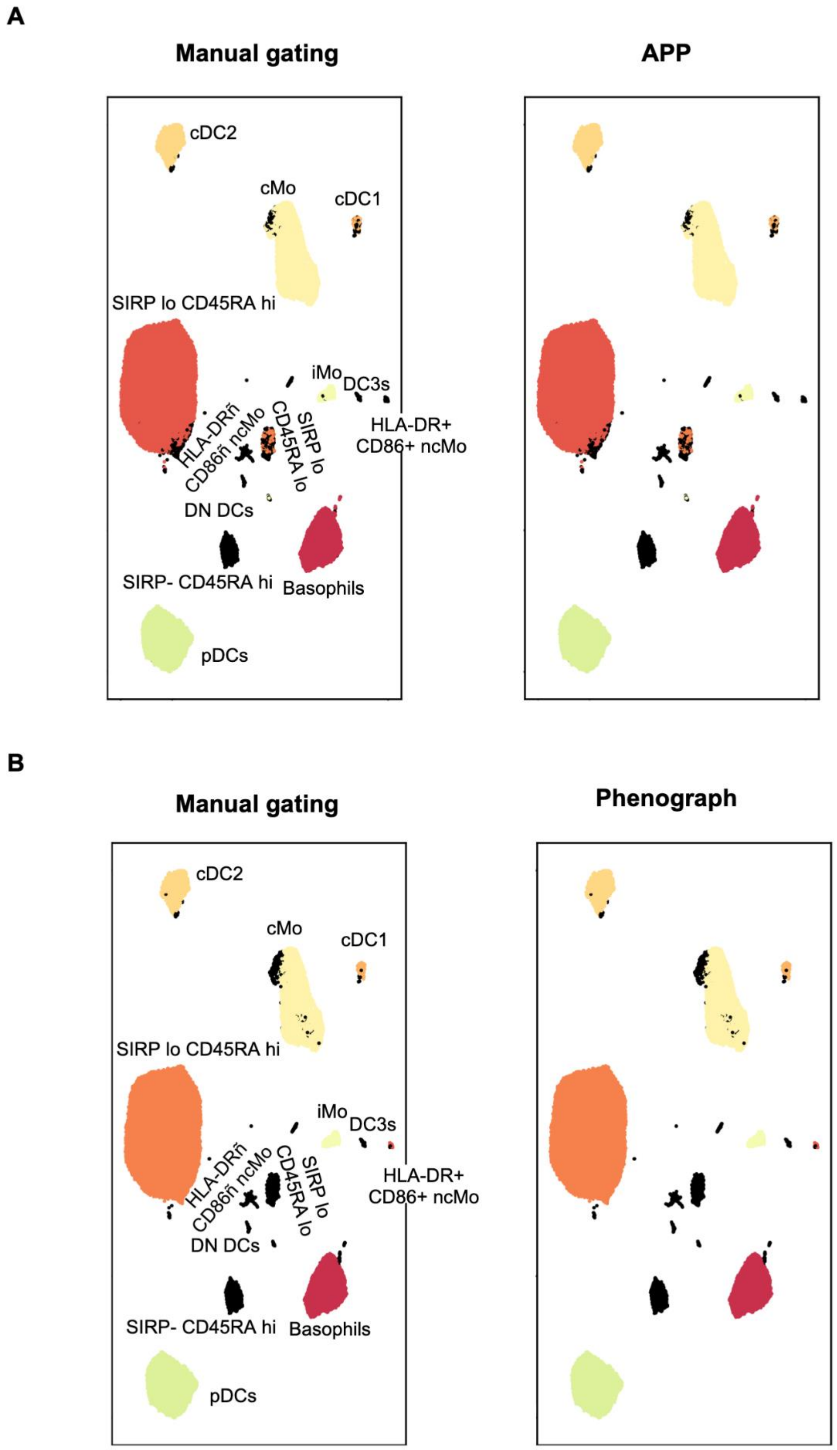

Supplementary Figure 6. An illustration of misclassification by APP (A) and Phenograph (B) compared to manually gated cell population annotations. Misclassified events, calculated using the automated label transfer pipeline, are highlighted in black.

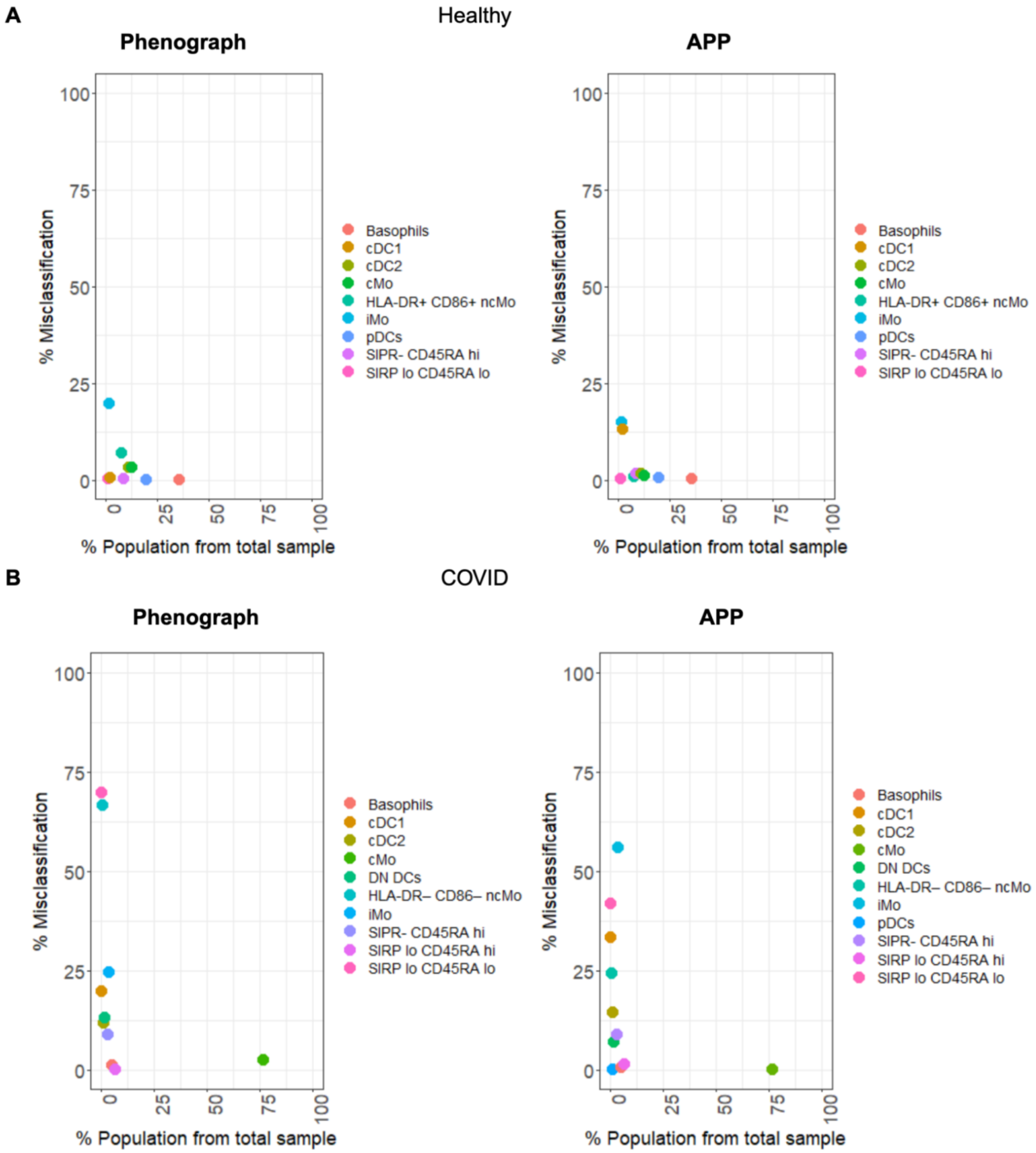

Supplementary Figure 7. Smaller cell populations are more prone to misclassification by clustering algorithms in both healthy donor (A) and COVID-19 patient samples (B).

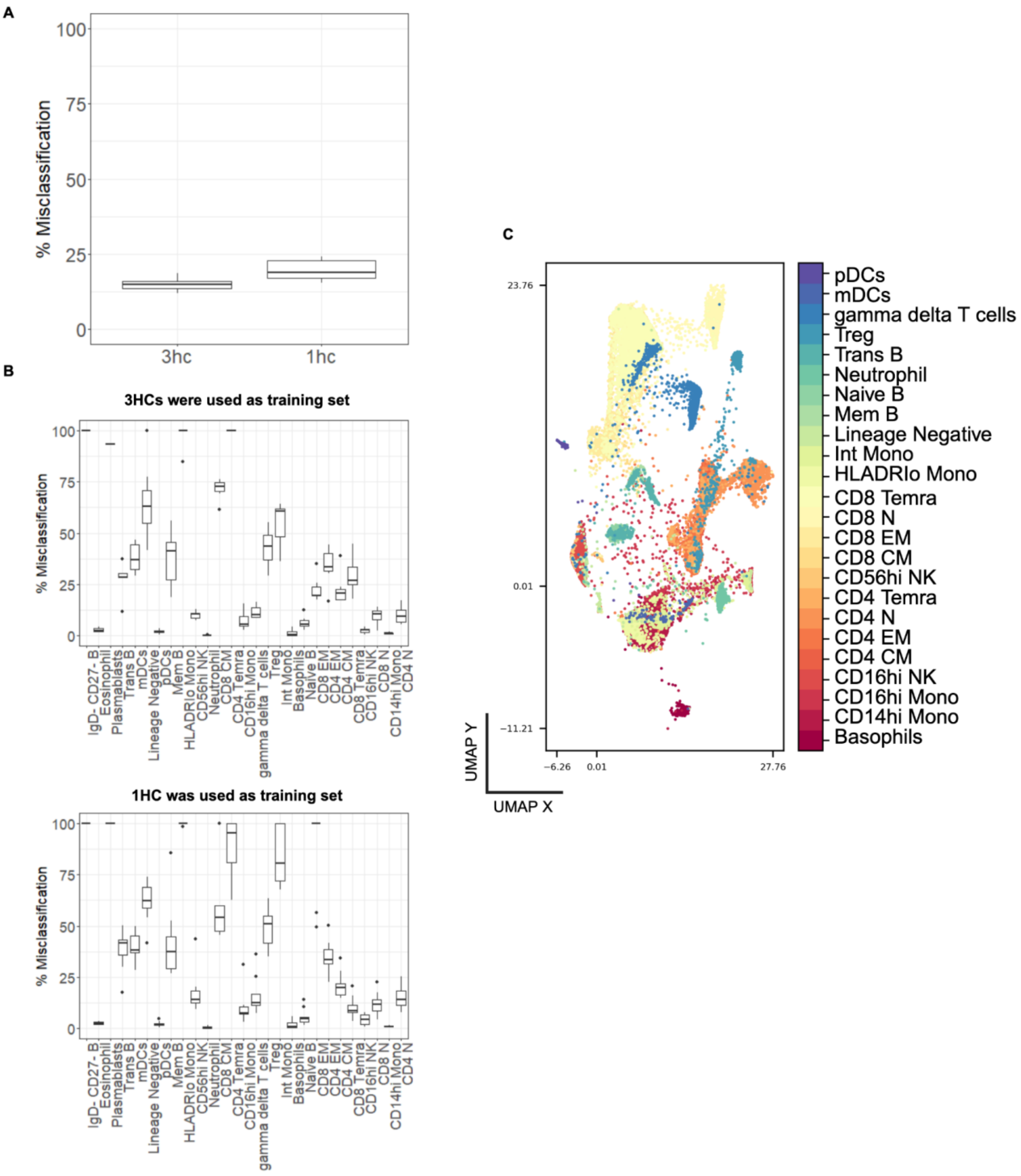

Supplementary Figure 8. Discrepancies between the underlying data topology and the ground truth labels could negatively affect the performance of the label transfer pipeline. A. Randomly chosen one and three (out of ten) healthy control (hc) PBMCs samples, characterized with the ~30 marker CYTOF panel, were used as

training sets and the rest of samples were used as the test set. **B.** The primary source of misclassification arises from the more heterogeneous nature of cell populations than initially identified with the established expert-defined manual gating strategy. For instance, the gamma delta T cells population on panel **C** is actually distributed between the two clusters, as data topology suggests.

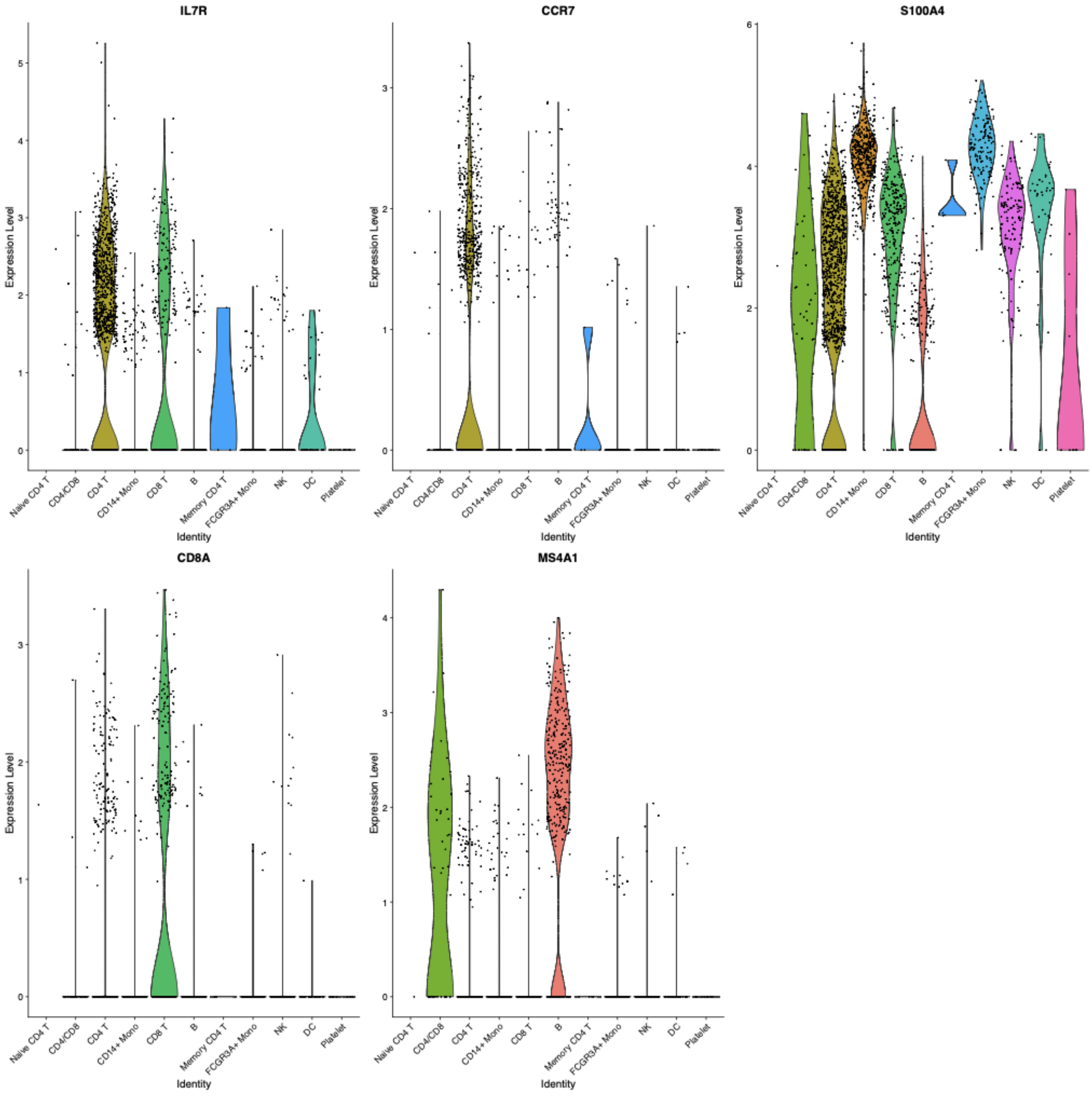

Supplementary Figure 9. The expression of B and T marker genes in cell subsets identified within the PBMCs dataset. MS4A1 is a B cell specific marker, the rest are CD4 and CD8 T cell specific markers. The group of cells that was misclassified by APP is here labeled "CD4/CD8".

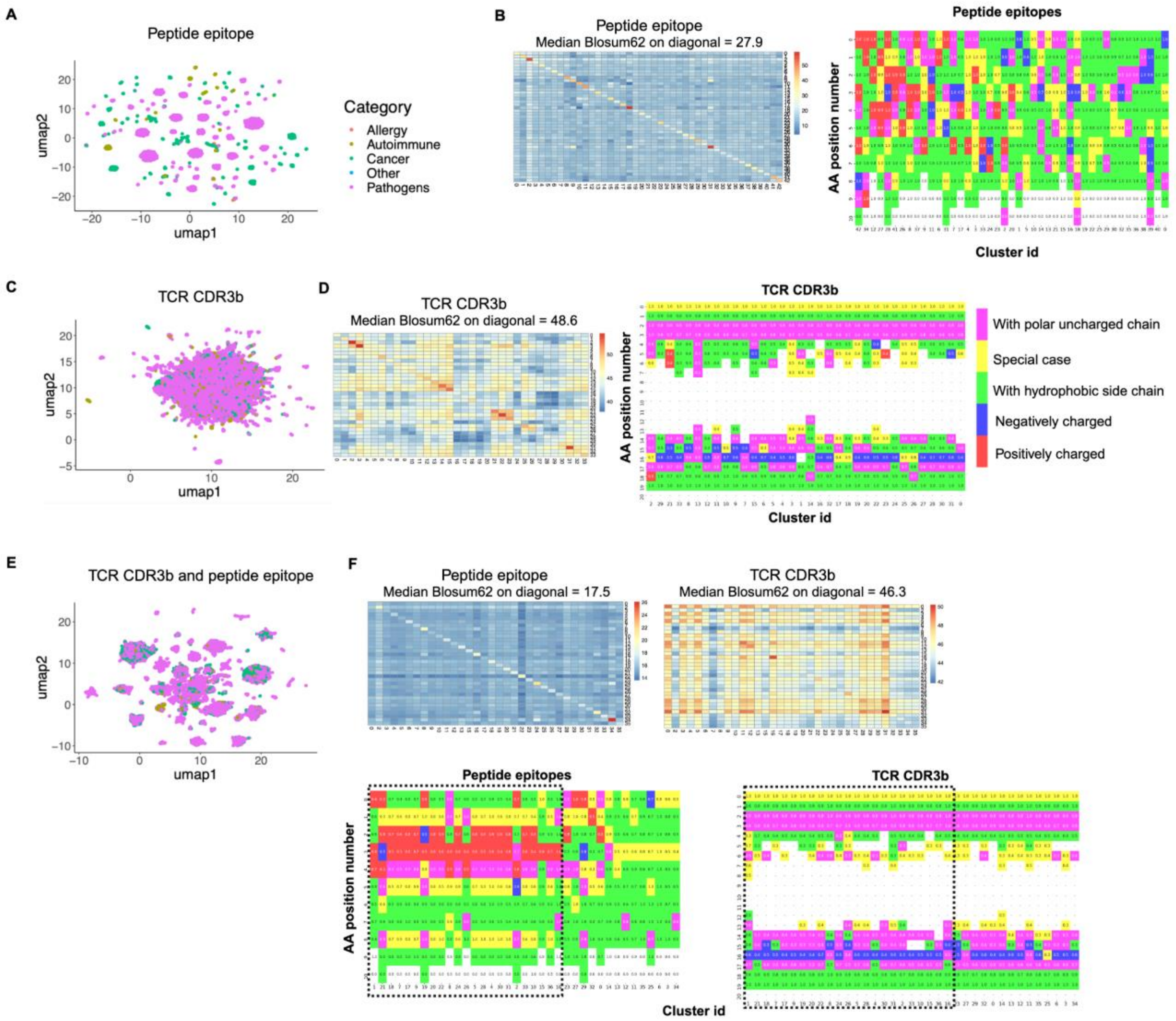

Supplementary Figure 10. Sequence similarity and amino acid R group properties analysis of ESM embeddings generated for peptide epitope sequences (A), TCR CDR3b sequences (C), and the concatenation of both TCR and peptide embeddings (E). Amino acids at a specific position within a designated cluster were categorized according to their R group properties (as indicated in the inserted legend). The most prevalent property within each group is presented, along with its corresponding probability (**B**,**D**,**F**).

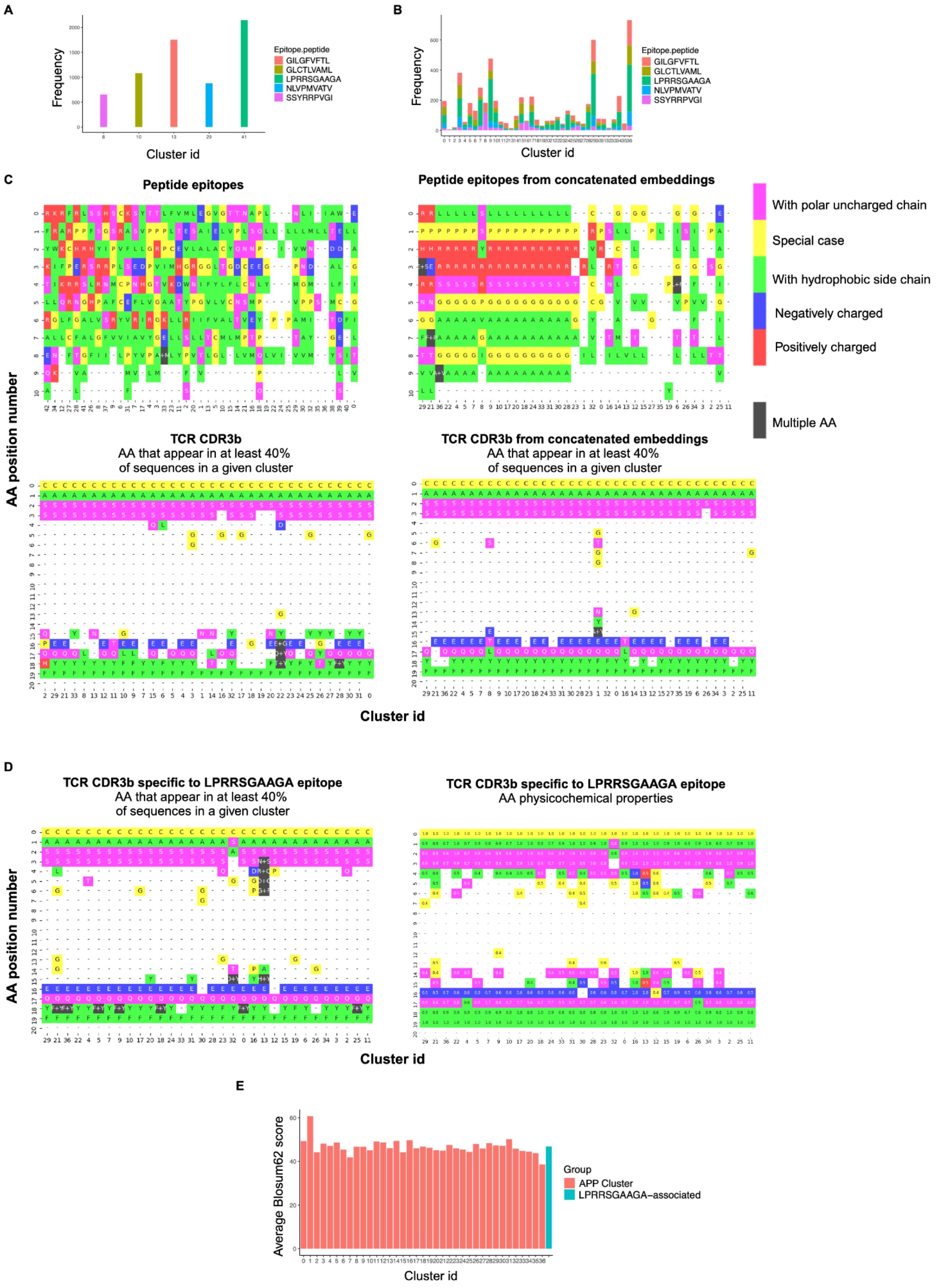

A
Frequency
Cluster id
Epitope.peptide
GILGFVFTL
GLCTLVAML
LPRRSGAAGA
NLVPMVATV
SSYRRPVGI
B
Frequency
Cluster id
Epitope.peptide
GILGFVFTL
GLCTLVAML
LPRRSGAAGA
NLVPMVATV
SSYRRPVGI
C
Peptide epitopes
Peptide epitopes from concatenated embeddings
With polar uncharged chain
Special case
With hydrophobic side chain
Negatively charged
Positively charged
Multiple AA
AA position number
TCR CDR3b
AA that appear in at least 40%
of sequences in a given cluster
TCR CDR3b from concatenated embeddings
AA that appear in at least 40%
of sequences in a given cluster
Cluster id
D
TCR CDR3b specific to LPRRSGAAGA epitope
AA that appear in at least 40%
of sequences in a given cluster
TCR CDR3b specific to LPRRSGAAGA epitope
AA physicochemical properties
AA position number
Cluster id
E
Average Blosum62 score
Cluster id
Group
APP Cluster
LPRRSGAAGA–associated

Supplementary Figure 11. No common binding motif is identified on TCR CDR3b, even when considering similar peptide epitopes. The distribution of the five most common peptides (in the database utilized for this study) appears highly cluster-specific in the context of single-class embeddings (**A**). However, when using concatenated embeddings (CDR3b and peptide), these five peptides are dispersed across multiple clusters (**B**). **C.** No discernible binding motifs appear in the TCR CDR3b, whether analyzed individually (left side) or as concatenated embeddings (right side). Even when focusing exclusively on CDR3b sequences specific to the LPRRSGAAGA peptide (**D**), these sequences exhibit no notable increase in sequence similarity (**E**), as approximated by the Blosum62 score, when compared to the CDR3b sequences clustered together in the concatenated embeddings data presented in Figure 6.

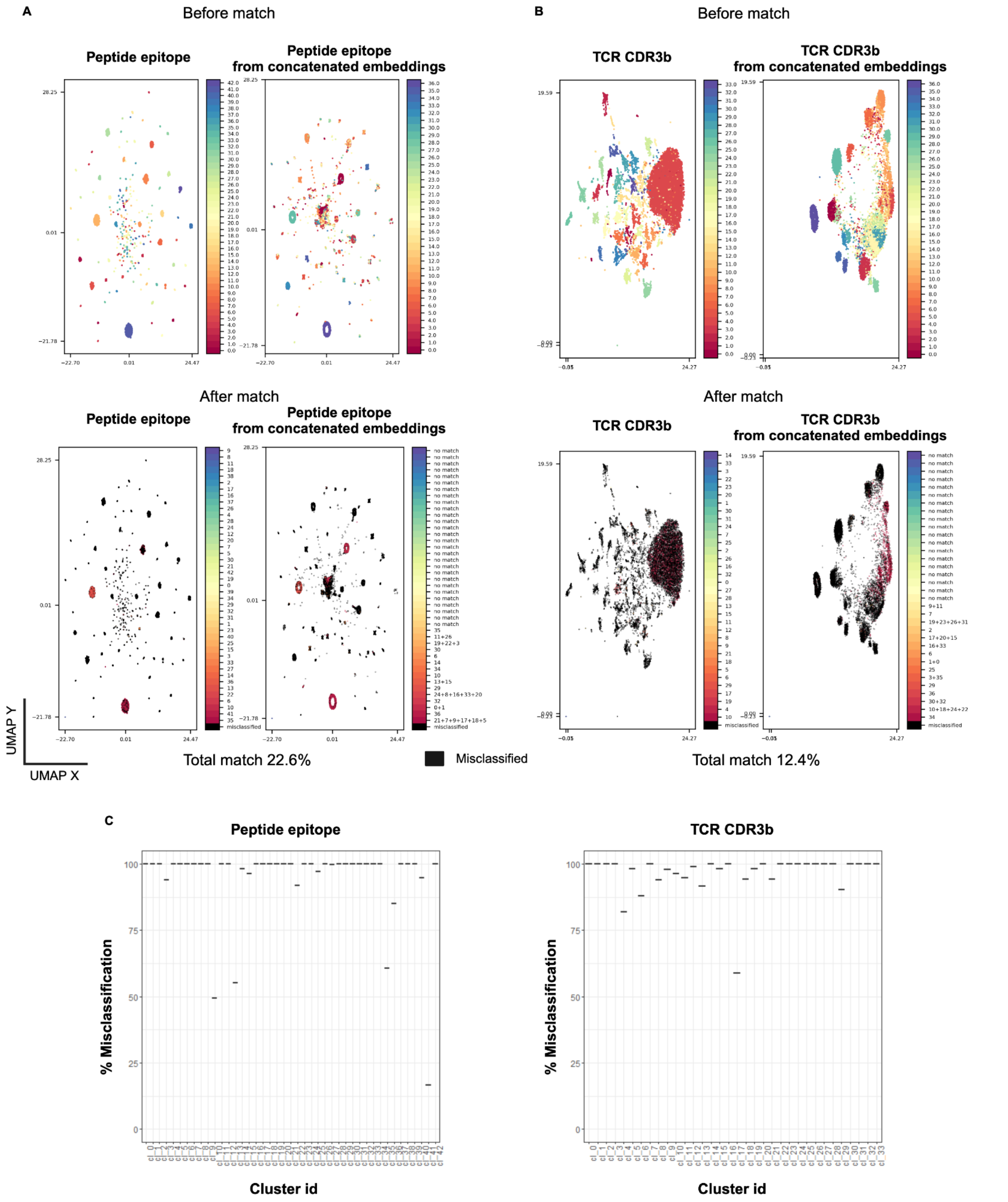

Supplementary Figure 12. Concatenation of TCR CDR3b and peptide embeddings results in distinct similarity criteria captured by the ESM model. The automated

label transfer pipeline was employed to align clusters obtained for the same class of sequences (peptides (**A**) and TCR CDR3b (**B**)) generated from the single class embeddings and concatenated embeddings. The label transfer pipeline was executed on 30 PCs, using the APP cluster labels generated for the single class embeddings as the training set. On the top row of panels A and B, cluster IDs are shown before the cluster alignment, and thus the same color may represent two unrelated clusters on the left and right UMAP plots for each class. On the bottom row of panels A and B, cluster IDs are shown after the match/cluster alignment, and thus the same color represents aligned clusters within the same sequence class. Non-matched clusters are shown in black. **C.** Per-cluster-ID misclassification as estimated by the label transfer pipeline.

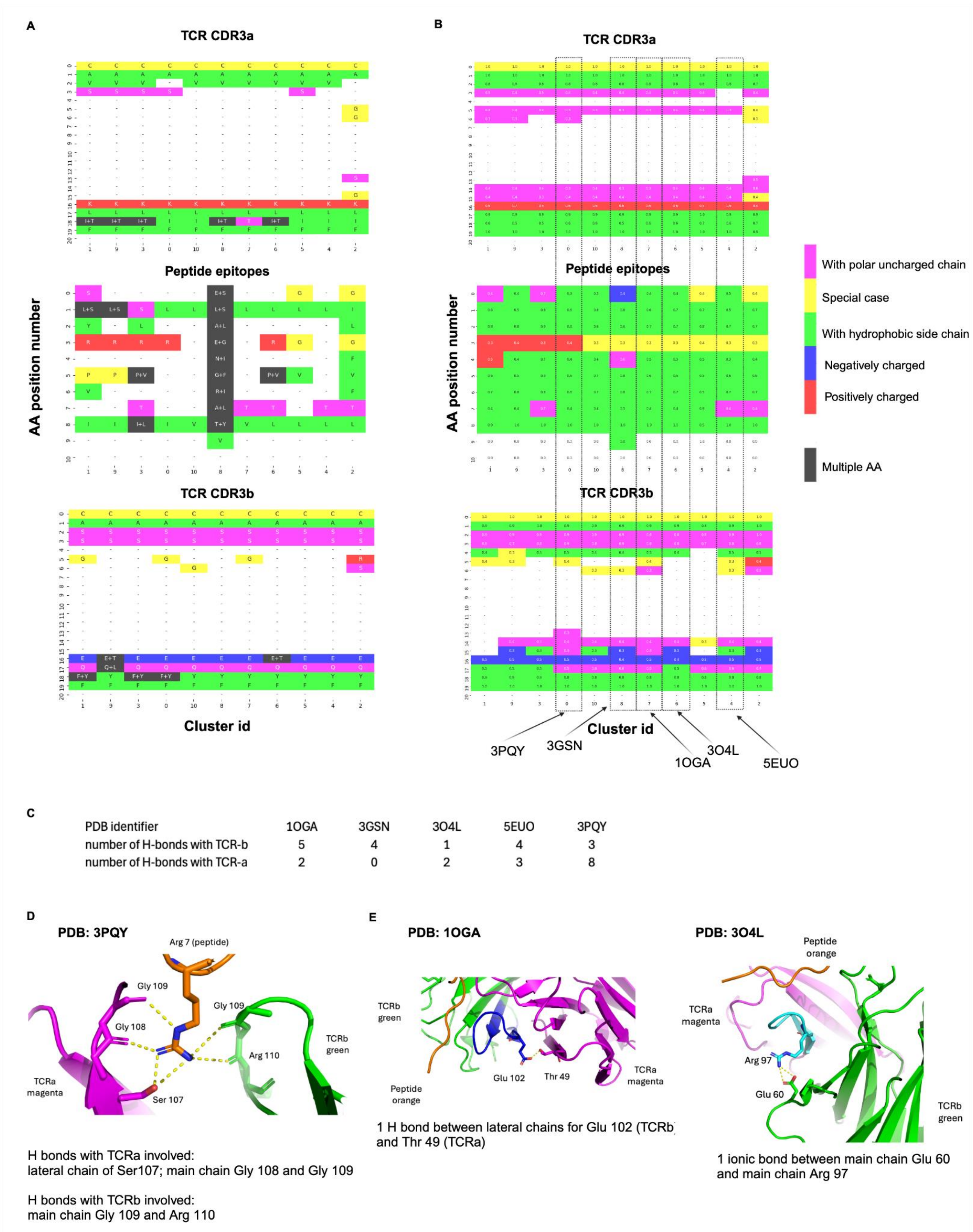

Supplementary Figure 13. The TCR CDR3b sequences exhibit an enrichment of negatively charged amino acids, while TCR CDR3a sequences are characterized

by an enrichment of positively charged amino acids. A. Amino acids that appear with more than 30 percent probability in a given position and in a given cluster are color-coded based on their R group property. These physicochemical maps are constructed for the 4000 CDR3a-CDR3b-peptide data set presented on the right side of Figure 6. **B.** Amino acids at a specific position within a designated cluster were sorted into the corresponding R group property, as specified in the legend. The most dominant property within each group is displayed, along with its associated probability. The results presented focus on R group property classes that exhibit a prevalence of more than 30 percent at a given position within a designated cluster. Analysis of these properties for the peptides showed important hydrophobic enrichment in all clusters (middle panel right, green color). For five independent clusters, we found in the PDB five TCR-pMHC crystal structures and analyzed them. **C.** All showed that the antigen directly interacted with both, alpha- and beta- TCR units. Unexpectedly for such hydrophobic peptides was the presence of H-bonds, noticed for all of them (from 2 to 8 H-bonds) and involving both TCRab. In the center of peptides, we noticed either the presence of one positively charged amino acid (often Arg), or the presence of one Gly. **D.** Structure analysis of PDB_3PQY showed that Arg7 has its 3-nitrogens from the lateral chain involved in the formation of H-bonds with TCRa (4 H-bonds) and TCRb (2 H-bonds). We suggest that through the formation of all the H-bonds the peptide orients itself between the TCRs sub-units and complement the stable 3D surface of the TCRs. Such H-bonds also participate in the stability of the [peptide-TCRs] complex and the presence of Gly (B, middle panel right called "Peptide epitopes", yellow color) allows partial bending of the peptide, when necessary. **E.** We noticed for all CDR3a and CDR3b the respective presence of conserved Lys and Glu (A,B). Analysis of the previous 5 structures showed that these amino acids don’t interact directly with the peptide, neither between themselves. They form H-bonds or ionic bonds between the two TCRs and through this mechanism participate in the pairing of the two subunits (see 1 H-bond between the conserved Glu102 (CDR3b) and Thr49 from TCRa; the ionic bonds bellow between Arg97 (CDR3a) and Glu60 (TCRb).

Click here to access/download;Personal Cover;Response_to_reviewers_Mar04_2025.pdf

Dear Editorial Board,

Below are our responses to the reviewers' comments. We sincerely appreciate the reviewers' thoughtful and thorough evaluation. We have addressed all concerns to the best of our ability and look forward to your feedback.

For your convenience, we have highlighted the changes in the revised manuscript in blue.

Reviewer #1:

***Comment*:** The authors reference the 'Curse of Dimensionality' in both the title and introduction but do not provide a detailed explanation of the issue. A more thorough discussion of this problem would help readers better understand how the proposed approach addresses and mitigates it.

***Response*:** We have added the explanation to the manuscript: “The well-known phrase "the curse of dimensionality" was coined by Richard Bellman [Morgenstern, D., & Bellman, R. 1962] and describes challenges that arise when working with high-dimensional data. A key issue is that the amount of data needed to obtain reliable results grows exponentially with the number of dimensions. Data analysis often depends on identifying regions where data points form meaningful clusters, but in high-dimensional spaces, such structures become sparse, making effective analysis increasingly difficult.”

***Comment*:** The presented pipeline is based on the custom procedure for 2d clustering/decision boundary construction. This procedure is described to some extent, but the underlying logic is could be explained in more details. For now, it seems counter-intuitive that the same projections should be analyzed twice (I mean, different separating boundaries are constructed for x-y and y-x plots).

***Response*:** We have added the explanation to the manuscript: “In our algorithm, the same projections are analyzed twice to determine a 2D cluster boundary in (x, y)-space: first to identify the x-projection of the boundary and then to determine its y-projection.” We also added a clarification to the Supplementary Figure 1 legend: “Supplementary Figure 1. Example of an optimal decision boundary search for one of the 2D data projections. The decision boundary begins at the left edge and ends at the right edge of a 2D projection. However, in xy vs yx orientations, the left and right edges differ, leading to distinct decision boundaries. Therefore, xy and yx projections should be analyzed independently.”

***Comment*:** Additionally, the "initial distribution density H" is not defined.

***Response*:** We have appended the explanation "—that is, the initial distribution density H" to the end of the phrase: "Otherwise, for each 2D (x,y) projection mapped onto a unit square (side length of 1), we build the Gaussian-smoothed histogram H(x,y) of the data points." The revised sentence in the manuscript now reads: "Otherwise, for each 2D (x,y) projection mapped onto a unit square (side length of 1), we build the Gaussian-smoothed histogram H(x,y) of the data points— that is, the initial distribution density H."

***Comment*:** Overall, there seem to be a lot of existing algorithms for construction of optimal separating boundary or even automatic clustering of data in 2D space. It is unclear what advantages and disadvantages they have compared to the procedure developed by the authors (probably higher computational cost?).

***Response*:** Most existing clustering algorithms operate in high-dimensional space rather than in 2D, making them vulnerable to the curse of dimensionality. Previous implementations of projection pursuit clustering algorithms sequentially operate on 2D projections; however, as discussed in the Introduction of our manuscript, their criteria for selecting the most informative low-dimensional projections was not optimized for identifying decision boundaries with the lowest data density between clusters-a crucial aspect of cell phenotyping. Instead, previous implementations of the projection pursuit algorithm primarily focused on identifying projections with "high" information content, such as those that deviate from normality or uniformity.

We have now emphasized this point in the manuscript with the following text: "The concept of exhaustively exploring low-dimensional projections of high-dimensional data has existed for a few decades. Historically, efforts have been made to systematically explore low-dimensional projections, known as the "grand tour" [Cook et al., 1995], or to optimize specific criteria for identifying informative projections, such as those that reveal structure in the data by deviating from normality or uniformity [Friedman et al., 1974]. However, challenges in determining the optimal criterion and the computational complexities associated with processing numerous low-dimensional projections have hindered the widespread adoption of projection pursuit methods for data clustering tasks."

***Comment*:** The authors tested their approach against three state-of-the art clustering algorithms (HDBSCAN, KMeans and Phenograph), but they were operating in the original high-dimensional space. The possibility of using these algorithms in 2D projections seems obvious and at least should be discussed.

Response**:** While HDBSCAN, KMeans, and Phenograph clustering can be applied to individual 2D projections, these algorithms do not include a mechanism to systematically traverse the entire set of 2D projections. They lack recursive steps that would score and evaluate each 2D projection to iteratively refine data splits for downstream processing, which is necessary for effectively identifying final clusters in a multidimensional dataset. Thus, the only way to directly compare the final clusters produced by APP with those from HDBSCAN, KMeans, and Phenograph is to run HDBSCAN, KMeans, and Phenograph on the full high-dimensional dataset rather than on individual 2D projections. Since a multidimensional point space can be uniquely represented by its complete set of 2D projections, it follows that comparing APP with modern multidimensional clustering algorithms (e.g., HDBSCAN, KMeans, and Phenograph) within these 2D projections should be sufficient.

We added the following explanation to the manuscript: "Since a multidimensional point space can be uniquely represented by its complete set of 2D projections, it follows that comparing APP with modern multidimensional clustering algorithms (e.g., HDBSCAN, KMeans, and Phenograph) within these 2D projections should be sufficient."

Comment**:** In some cases, clusters may be well-separated in 3D space but appear mixed in 2D projections (such as xy, xz, yz). This can occur, especially when there are many clusters, but the situation might be improved through proper rotation of the coordinate system. Could the authors comment on the potential for this improvement? Additionally, an optimal decision boundary should ideally be independent of in-plane rotations, including the interchange of x- and y- axes. Could the authors suggest possible ways to algorithm modification to satisfy this requirement?

Response**:** We thank the reviewer for this insightful comment. Indeed, in some cases clusters that are well-separated in higher-dimensional space may appear mixed when projected onto certain 2D planes. To address this, proper rotation of the coordinate system may help uncover projections that better reveal separation. While our current approach does not explicitly incorporate rotation optimization, one potential extension could involve employing rotation search methods such as those used in Projection Pursuit to identify projections that maximize separation between clusters.

Regarding the independence of the decision boundary from in-plane rotations and axis interchange, we agree that this is an important consideration. One potential modification would be to apply symmetry operations (e.g., swapping the x- and y-axes) during the decision boundary search and averaging the resulting decision boundaries to enforce rotational

invariance. Additionally, incorporating optimization steps that account for rotational symmetry during boundary determination could help ensure robustness.

We will consider these suggestions for future algorithmic improvements and exploration in subsequent studies. Additionally, we have included the following statement in the Discussion section: “Also, in some cases clusters that are well-separated in higher-dimensional space may appear mixed when projected onto certain 2D planes. To address this, proper rotation of the coordinate system may help uncover projections that better reveal separation. While our current approach does not explicitly incorporate rotation optimization, one potential future extension could involve employing rotation search methods such as those used in projection pursuit to identify projections that maximize separation between clusters.”

Minor points:

***Comment*:** Figure 1C appears overwhelming due to the large number of 2D projection plots, which seem disorganized. The authors might consider grouping the plots for better clarity. Additionally, the text on the plots is too small. Moreover, there seems to be no need to present six projections in 3D space for the identified clusters.

***Response*:** We simplified panel C and updated the Figure 1C legend accordingly. Additionally, we increased the text font size for better readability.

***Comment*:** Equation (1): The text mentions a square root, but the expression is raised to the power of 0.1. Should it be 0.5?

***Response*:** The key point is that Mann's original formula for the total number of two-dimensional bins includes a power of 0.2. Taking the square root of this power results in a power of 0.1 in our Equation (1), ensuring that the calculation is correct.

***Comment*:** The sentence "If the number of cells at the input to the recursion step is less than 2 * min_cluster_size" might be clearer if "the number of cells" is changed to "the number of points."

***Response*:** We have incorporated this suggestion.

Reviewer #2:

***Comment*:** The authors should present the silhouette of the clusters.

Response: We thank the reviewer for this suggestion and have updated the manuscript accordingly. Please refer to Figures 2 and 3, as well as the legend of Figure 4.

Comment: Paper should be revised for minor typos.

Response: We have revised the manuscript accordingly.

Reviewer #3:

Comment: However, some control experiments are not fully explained. For example, the optimization of APP parameters and the limited comparison with other clustering algorithms could be expanded.

Response: APP has only one required user input parameter: the minimum cluster size, which should be set based on the smallest population the user expects to detect in the dataset. Reducing this value introduces smaller clusters, as expected, while other APP clustering outcomes remain unchanged. All other parameters, such as Gaussian smoothing width, are optional. We have set default values optimized for the Calinski-Harabasz index based on the datasets presented in the manuscript. These parameters are user-adjustable to allow optimization for other data types, guided by the Calinski-Harabasz index. We have now clarified this more explicitly in the Methods section.

We have also updated the GitHub folder with a more detailed parameter description.

We now include comparisons to additional commonly cited clustering methods in flow cytometry, beyond PhenoGraph: flowSOM (1,310 Google Scholar citations since 2021) and SPADE (272 Google Scholar citations since 2021). Please refer to the updated Figure 2.

Comment: Additionally, while ground-truth validation is used for some datasets, others rely on expert evaluations, which can introduce bias.

Response: Unfortunately, the current state of the art lacks ground-truth validation for assessing and confirming that identified populations are functionally distinct. In most cases, expert evaluation serves as the “gold standard.” We acknowledge that this introduces bias and have explicitly discussed it in the Conclusions section of the manuscript. In this study, we introduced a method to generate functionally validated ground truth; however, we also recognize that this is a resource-intensive endeavor, making it impractical to expect it to be routinely performed for every new experiment.

“In many biological real-world datasets, the availability of a clear "ground truth" can be challenging. As illustrated in the examples presented, reliance on domain experts' knowledge-driven clustering or clustering done with widely-adopted approaches serves as a substitute for ground truth. While expert-driven clustering provides a valuable reference point, a more accurate (albeit labor-intensive) method for assessing clustering performance involves conducting functional tests on groups of cells assigned to the same cluster. By observing the functional "purity" and homogeneity of a given cluster compared to other cell clusters in the sample, researchers can achieve a more precise evaluation of the clustering results.”

Comment: The total sample size (six healthy controls and six COVID-19 patient) analyzed in the paper appears small for drawing robust conclusions, especially considering the biological variability in human samples. However, it's quite understandable that the number of samples is limited due to the availability of biological samples and the high cost associated of experimental techniques.

Response: We appreciate the reviewers’ feedback and their understanding regarding the relatively low number of samples, which is due to the limited access to this type of biological material and the relatively high cost of the associated experiments. We agree with the reviewer and have considered the biological variability among COVID-19 patients. For example, the six COVID-19 samples analyzed here were collected from patients with severe disease in the ICU, and we intentionally avoided mixing samples from COVID-19 patients with different disease severity. We recently showed that despite the variability among COVID-19 patients, the patients in ICU with severe disease remain distinguishable from healthy controls using flow cytometry to characterize major immune lineages (see Eddins et al., Blood Adv. 2023). Nonetheless, while six samples per group may be relatively low for deriving strong biological conclusions (due to potential sample-to-sample heterogeneity, and we have adjusted our statement accordingly), from a statistical perspective, a cohort of twelve samples is sufficient to assess the performance of the clustering and cluster-matching pipelines.

We have incorporated the relevant statements in the revised manuscript.

Comment: The study provides strong evidence for APP's ability to identify meaningful clusters and compares it with other clustering methods. However, some conclusions need stronger evidence. For instance, the claim that APP handles sparse data better than other methods is not consistently proven across all datasets.

Response: The overall theoretical conclusion regarding the superiority of projection pursuit over high-dimensional methods in clustering sparse cell populations is derived from the definition of

the curse of dimensionality and the well-established fact that, to achieve a given accuracy with typical high-dimensional methods, the sample size must increase exponentially with the number of dimensions. This leads to the logical conclusion that sparse cell populations are particularly susceptible to the effects of the curse of dimensionality. For further illustration, please refer to Supplementary Figure 1 in [Meehan et al., 2019], which we also cited in our manuscript.

To illustrate this in practice, we updated Figure 2 (new panel C) to show the relationship between percent misclassification and the abundance of cell populations in the sample. For "purity," we present this data using samples with functionally validated ground truth labels.

***Comment*:** The statistical methods used in the study, such as the Calinski-Harabasz index, are suitable for the types of data analyzed. PCA and Gaussian smoothing are reasonable choices for reducing data complexity and handling noise. However, some choices, like the beta parameter in APP, seem to be based on trial and error and need more justification.

***Response*:** We have included the explanation in the manuscript: "APP has only one required user input parameter: the minimum cluster size, which should be set based on the smallest population the user expects to detect in the dataset. Reducing this value introduces smaller clusters, as expected, while other APP clustering outcomes remain unchanged. All other parameters, such as Gaussian smoothing width, are optional. We have set default values optimized for the Calinski-Harabasz index based on the datasets presented in the manuscript. These parameters are user-adjustable to allow optimization for other data types, guided by the Calinski-Harabasz index."

We have also updated the GitHub folder with a more detailed parameter description.

***Comment*:** Additionally, the study reports accuracy metrics but could include other measures like F1-score to give a more complete picture of performance.

***Response*:** We thank the reviewer for this suggestion and have now included the F1-measure in Figures 2 and 3.

***Comment*:** There are minor grammar issues and repetitive phrases that could be cleaned up. E.g.,
- P.2: While the latter challenge is computational rather than a fundamental scientific limitation, it can be a serious practical hurdle.
- P.3: These insights include evaluating hypotheses regarding the existence of a binding motif between in CDR3b of TCRs that recognize the same peptide ...
- P.3: To gain a deeper understanding ... we initially applied ...

- etc.

***Response*:** We have revised the manuscript accordingly.

***Comment*:** Overall, moderate editing would improve the readability and flow of the manuscript.

***Response*:** We have revised the manuscript accordingly.

Reviewer #4:

***Comment*:** In my opinion, in some parts, the manuscript is almost overwhelming with different applications and datasets that could be explained in more detail. Potentially, Figure 6 could even be split into a separate manuscript to streamline the different applications of APP. However, this can be addressed in the revised version.

***Response*:** There is a clear logic behind how the material is presented: we first demonstrate the method's performance using a dataset with functionally validated ground truth (GFP mice data). Next, we apply the method to a dataset where expert-defined manual gating serves as the "ground truth" (COVID dataset). We then apply the algorithm to single-cell mRNA expression and imaging data, where no predefined ground truth is available. However, clustering outcomes are assessed using domain knowledge from pathology and immunology. For the imaging data, both expression patterns and co-location/spatial distribution are used to evaluate cluster quality. Finally, we apply APP clustering in a fully exploratory mode, where no ground truth is available and no immediate assessment of clustering outcomes is possible due to the lack of pre-existing domain knowledge about how TCR receptor embeddings should cluster with respect to their cognate antigens (TCR data). This approach covers the range of real-world scenarios for using clustering algorithms, whether in the presence or absence of ground truth and domain knowledge.

We have now included this general statement in the Results section of our manuscript.

***Comment*:** I validated that all datasets and code are available. Some descriptions of the data could be improved, e.g., more detailed README files, etc. (see below). Perhaps the datasets could also be uploaded to permanent repositories in addition to the GitHub link to ensure permanent accessibility.

Response**:** Previously unavailable datasets are now shared on Zenodo, accompanied by corresponding readme files. Please refer to the updated version of the Data Availability section in the manuscript.

Introduction

Comment**:** While the limitations and objectives are clearly defined in the introduction, I believe the authors could expand on alternative, newer methods that people use for clustering. The authors largely describe the historical initial work by Friedman et al., 1982; Huber, 1985, which is 40-50 years old. For instance, newer clustering algorithms might be relevant to include, along with mentioning their limitations. For example, the authors benchmark APP with algorithms such as HDBSCAN and K-means, but these are not introduced. Additionally, mentioning graph-based clustering and dimensionality reduction techniques such as UMAP and t-SNE, and how they address challenges in high-dimensional clustering and have become standard for scRNA-seq data analysis, might be helpful.

Response**:** We have incorporated these suggestions and revised the Introduction accordingly.

Comment**:** Since much of the manuscript is about the validation of APP with real-world data, the authors may consider including a bit more information on why they chose these datasets and the different modalities (e.g., flow cytometry, scRNA-seq, multiplex imaging). I appreciate the diversity of the data, but expanding a little on the rationale would be helpful.

Response**:** This is a very useful suggestion; thank you. We have now included the rationale for selecting the datasets at the beginning of the Results section: “To encompass the range of real-world scenarios for clustering algorithm usage, both with and without ground truth or domain knowledge, we first demonstrate the method’s performance using a dataset with functionally validated ground truth. We then apply the method to a dataset where expert-defined manual gating serves as the ground truth. Next, we test the algorithm on single-cell mRNA expression data and imaging data, which lack predefined ground truth. However, cluster evaluation is enabled using domain knowledge from pathology and immunology, leveraging expression patterns and spatial distribution. Finally, we apply APP clustering in a fully exploratory mode, where no ground truth or pre-existing domain knowledge is available to assess how TCR receptor embeddings cluster with respect to their cognate antigens.”

Comment**:** Additionally, the authors may clarify the novelty or major advantage of the APP clustering tool. For instance, the authors mention that APP is an alternative clustering approach, but they could perhaps elaborate on its major strengths or novelty in the last paragraph.

Response**:** We thank the reviewer for this suggestion. In the last paragraph of the Introduction, we have now highlighted that the major advantage and novelty of APP clustering lie in its ability to automatically uncover meaningful structures in high-dimensional data while mitigating the curse of dimensionality, a common challenge in high-dimensional clustering. Unlike traditional projection pursuit, where an analyst manually explores projections to identify patterns, APP automates this process by recursively finding low-dimensional projections with the smallest data density between clusters and continuing the analysis until no further splits are detected.

Comment**:** The last three paragraphs of the introduction discuss the findings of this manuscript. I would suggest shortening this part into a single last paragraph in the introduction.

Response**:** We followed the recommendation and condensed the last three paragraphs into a single, more concise paragraph.

Minor comments:

Comment**:** The authors could use a reference instead of a link in the introduction when referring to supervised UMAP.

Response**:** To our knowledge, there is no peer-reviewed paper specifically dedicated to supervised UMAP. However, an explanation has been provided by the first authors of the original UMAP paper, which can be found here: https://github.com/lmcinnes/umap/issues/135.

“We have not written a paper on the subject unfortunately -- there really isn't enough material for one. The idea is pretty straightforward (intersecting fuzzy simplicial sets / taking limits) and largely self-evident. The implementation is publicly available, and hopefully takes care of the technical matters of "making it work in practice". I would certainly welcome suggestions for where code comments might be added to make things clearer.”

Comment**:** It may not be ideal to use "APP" for the name of the software in the context of models of Alzheimer's disease, as most AD researchers associate APP with Amyloid Precursor Protein.

Response**:** We appreciate the reviewer bringing this up and are aware of the term APP in the neuroscience field. However, we believe that for a broader readership, particularly computational scientists, the term APP is more commonly associated with "Application" (or "app" for short). Therefore, we would like to retain the name APP for our algorithm.

Methods

Comment: Since the authors generated some of their own data and used publicly available datasets, it might be helpful to add a table with an overview of all datasets they used. It took me some time to understand which datasets were generated in this study and which ones were taken from a public database. Perhaps the authors could explain why they decided to include their own data if the main purpose is to benchmark APP against other clustering software.

Response: We thank the reviewer for this suggestion. We have reformatted the Data Availability section into a table to provide the requested information. Currently, there is a lack of datasets with real ground truth, and most datasets, particularly in flow cytometry, imaging, and scRNA-seq, are accompanied by user-defined ground truth based on field-specific best practices for data preprocessing and annotation. Our decision to include our own data was driven by our commitment to adhering to what we believe are the best practices for preprocessing and annotating cell populations. As illustrated in Supplementary Figure 3, generating ground truth cell population annotations is a challenging task.

Comment: For the flow cytometry data, the authors could add the manufacturer, species, and dilution of the antibody.

Response: We have now incorporated this information into the manuscript.

Comment: More information about the antibodies and their dilution would also be beneficial for the multiplex imaging data.

Response: We have added clone names and working concentrations to the relevant Materials and Methods section of the manuscript.

Comment: The CyTOF methods are not properly described. Although the authors refer to previous studies, they should consider providing a bit more detail.

Response: Although the CyTOF data used in our manuscript was previously published and processed elsewhere, and we are only reusing it, we have now added the following description in the relevant methods section: “Whole blood was collected from consenting healthy human donors (N = 10), and peripheral blood mononuclear cells (PBMCs) were isolated and stained with a metal-conjugated 38-parameter mAb panel (see Table S1 in [Toghi Eshghi et al., 2019]), enabling the comparison of 28 immune cell subset frequencies [Toghi Eshghi et al., 2019]. Data were acquired using the Helios™ CyTOF® system (Fluidigm, South San Francisco, CA).”

***Comment*:** Why did the authors use Seurat v3? The current version is v5. Perhaps the authors could double-check whether their code is still executable with v5.

***Response*:** The original data analysis, as described in [Zhou et al., 2020], was conducted using Seurat v3. To avoid potential discrepancies due to version differences, we adopted the same version. However, we have now tested Seurat v5 and confirmed that the code remains executable, including cluster assignments. We have included a relevant statement in the manuscript.

***Comment*:** The description of the scRNA-seq analysis is quite brief; however, the coding data is available on GitHub. In case the authors have not done so, it would be helpful to deposit the data in a permanent database such as Zenodo (since the GitHub data could be altered or deleted).

***Response*:** We thank the reviewer for this suggestion. We have reformatted the Data Availability section to provide the requested information.

***Comment*:** All raw data and code appear to be available on GitHub. The authors could perhaps elaborate on the README files in the main folder to provide more detailed instructions and descriptions of the project.

***Response*:** The README in the main folder has been augmented with a more detailed description of the project and repository contents.

***Comment*:** Fig 2:
In Figure 2, the authors compare APP performance to widely used clustering methods in flow cytometry of immune cells from WT and RAG KO mice. The experimental design is very elegant, mixing the population of cells in different proportions to validate individual cell types, but I think this needs to be better explained in the results. Especially since most scientists reading this manuscript might have a bioinformatics background and may not be familiar with the mouse model and the experimental setup.

***Response*:** We thank the reviewer for the positive comments. We have expanded on this experimental approach in the text to include the following explanation: Mice lacking the RAG1 gene (i.e., RAG-KO) are deficient in immune cells known as B and T lymphocytes, while still developing all other major immune lineages, including myeloid cells and NK cells. Since the wild-type (WT) mice, expressing the RAG1 gene can develop both B and T lymphocytes, we intentionally mixed WT cells from a GFP+ mouse, which expresses green fluorescent protein on their lymphocytes, with cells from the RAG-KO mouse, which does not contain lymphocytes (i.e., WT-GFP mixed with RAG-KO). We used this experimental approach to define a biological

and technical ground truth. For example, any B and T cells identified by our new APP pipeline should express the green (GFP) protein since they can only come from the WT-GFP mice and not from the RAG-KO mice. If the pipeline detects any B and T lymphocytes lacking the green/GFP protein, these events would be considered as “misclassification” by the APP pipeline.

***Comment*:** Also, the clustering algorithms that were used as a comparison have several parameters that can be adjusted. How were those chosen? Did any of them lead to better/worse clustering?

***Response*:** We used the default input parameters recommended in available tutorials (e.g., Seurat: [https://satijalab.org/seurat/articles/pbmc3k_tutorial.html]) for the given data type, as parameter adjustments did not significantly improve clustering. This information has now been included in the relevant methods section.

***Comment*:** Perhaps in a comparison like this, it would be helpful to briefly mention the computational performance of the methods. For instance, how long did each algorithm take to run under which computer configuration?

***Response*:** Although we have now included these statistics for the dataset in Fig. 2 (see panel 2B), we emphasize that achieving robust clustering results is more critical than obtaining rapid but inaccurate outcomes.

***Comment*:** It is not clear to me how they generated the UMAP in Fig 2C. Is this based on the APP clustering? And then were the labels transferred from the other clustering algorithms? A bit more explanation is needed.

***Response*:** In response to these questions we now adjusted the Fig. 2D (previously 2C) legend accordingly: “The misclassification for the 50/50 mix is visually represented in black color using the automated label transfer pipeline, as detailed in Supplementary Figure 4. Here, the performance of each of the six clustering algorithms is assessed against the ground truth labels (functionally distinct cell types) used to construct the supervised UMAP embedding.”

***Comment*:** Did the authors perform any statistical analysis on the quantified data? The plots look convincing but should be tested using appropriate statistical tests in Fig 2D and E.

***Response*:** In response to this request, we have updated Fig. 2 accordingly. The performance of the APP algorithm in cell population classification is now evaluated using total and per-cell-type population misclassification, F1-measure, and Silhouette coefficient, and is compared to state-of-the-art clustering algorithms.

***Comment*:** Fig 3: The authors should add statistical analysis to Fig 3 A-D and F.

***Response*:** In response to this request, we have updated Fig. 3 accordingly. The performance of the APP algorithm in cell population classification is now evaluated using total misclassification, F1-measure, and Silhouette coefficient and compared to Phenograph. Per-cell-type population misclassification is shown in Supplementary Figure 7.

***Comment*:** The authors convincingly show that the label-transfer pipeline is very accurate when training on one healthy control sample, and that there were more discrepancies with COVID samples. I think the authors could expand a little more on the diverse responses of COVID patients and cellular shifts, which could also include cellular transition states. Also, intragroup variability is likely higher in COVID patients.

***Response*:** We thank the reviewer for raising this important point on biological variables in COVID-19 patients, ranging from asymptomatic to mild, moderate, and severe patients in the ICU. We have revised our manuscript to better describe the patients analyzed here for this method's paper. All six COVID-19 samples analyzed here were collected from patients with severe disease in the ICU, and we intentionally avoided mixing samples from COVID-19 patients with different disease severity, which could add additional biological variables. However, despite analyzing samples from the same disease severity (i.e., ICU patients), we agree with the reviewer that COVID-19 disease induces changes in immune cells, leading to transitional cellular states. These cellular states are dynamic and can vary across patients depending on various factors, including patient demographics, comorbidity, viral load, timing of infection to analysis, etc. Thus, it is expected that samples from COVID-19 patients would show more discrepancy compared to healthy controls. In any event, we recently showed that despite the variability among COVID-19 patients, the patients in ICU with severe disease remain distinguishable from healthy controls using flow cytometry to characterize major immune lineages and cellular states (see Eddins et al., Blood Adv. 2023).

We have incorporated the relevant information into the manuscript.

***Comment*:** The authors suggest that "these findings highlight new mechanisms of SARS-CoV-2-induced immuno-modulation that underlie the COVID-19 immunopathology in hospitalized patients," which might be a slight overstatement. They detect a subpopulation of monocytes, which is interesting. However, there is no data indicating a new mechanism. Toning this statement down would solve the issue.

Response: We agree with the reviewer and have now revised the manuscript to highlight that although our new findings might represent a novel mechanism of immune modulation in severe COVID-19, further studies with more patients and different disease states are required to determine whether this is a general mechanism of SARS-CoV-2 infection or a phenotype unique to our small patient cohort.

Comment: Fig 4: The scRNA-seq clustering in Seurat depends on the parameters chosen. Did the authors use default parameters?

Response: Parameters were selected based on the Seurat tutorial (https://satijalab.org/seurat/articles/pbmc3k_tutorial.html), using default settings with a resolution of 0.5. We have added this information to the relevant Methods section.

Comment: The authors claim that naïve and memory CD4 T cells do not show any distinction in clustering in any pairwise dimensions explored by APP but refer to Supplementary Data without specifying where in the Supplementary Data this information can be found. Please add a specific reference to where this information can be found.

Response: Although we believe this supplementary data was uploaded to the journal server during the submission process, we have now uploaded it to Zenodo (https://zenodo.org/records/14874269) and provided a link in the manuscript.

Comment: Did the authors examine CD45RA/PTPRC and CD62L/SELL levels in their PBMC RNA-seq data? These markers are usually used to distinguish naïve and memory CD4 T cells.

Response: We did; however, in this dataset, there was no significant difference in PTPRC and SELL levels among the T cell clusters. We have attached a figure to support this statement.

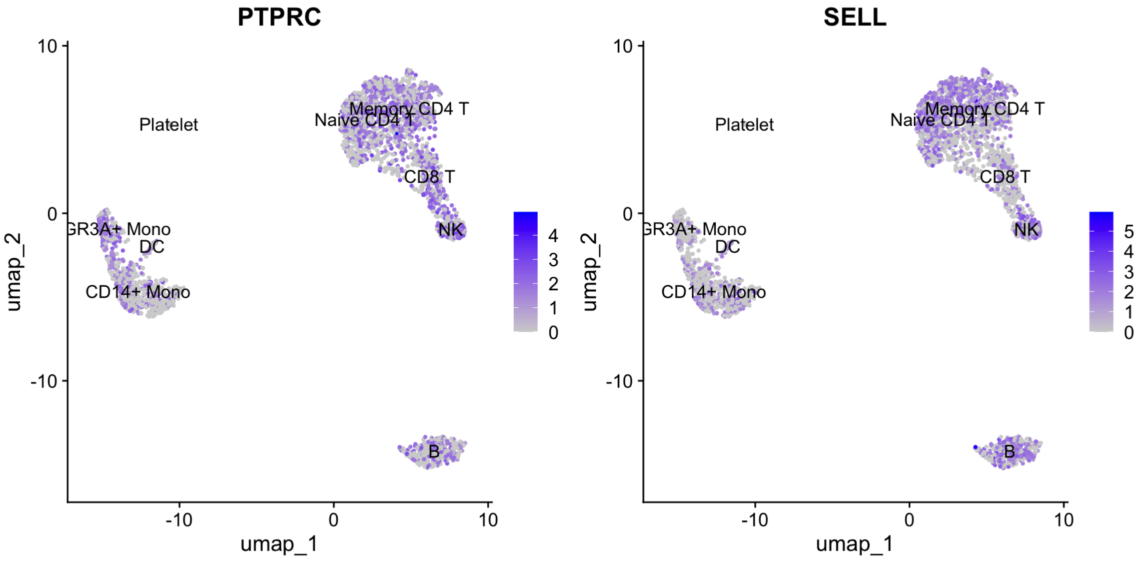

Comment: For the scRNA-seq dataset, it would be helpful to compare the performance in terms of the time required for clustering. scRNA-seq datasets can have millions of cells, and Seurat may face challenges with conventional analysis. How did APP perform compared to Louvain clustering in terms of speed? Could the authors also test it on a larger dataset?

Response: We thank the reviewer for this suggestion and have incorporated the following information into the manuscript: "To assess the computational complexity of the APP algorithm, which is generally O(N^2), where N is the number of dimensions, we recorded the clustering time for APP versus Seurat v5 using a MacBook Pro with a 2.3 GHz Dual-Core Intel Core i5 and 8 GB memory. For the PBMC dataset (containing 2700 cells), Seurat clustering (resolution = 0.5) took 1–2 seconds in real time. APP, with 10 PCs and a minimum cluster size of 10, took 11m 19.952s real time, 26m 6.587s user time, and 2m 43.988s system time. APP, with 10 PCs and a minimum cluster size of 100, took 2m 16.023s real time, 5m 47.742s user time, and 0m 34.924s system time."

Comment: Perhaps the authors could check whether the clustering algorithm in Seurat was updated between v3 and v5 (the most current version). If not, they could consider adding this information to the methods.

Response: The original data analysis for the 5XFAD dataset, as described in [Zhou et al., 2020], was conducted using Seurat v3. To ensure consistency and avoid potential discrepancies due to version differences, we adopted the same version for scRNAseq data processing within this manuscript. However, we have now tested Seurat v5 and confirmed that the code remains

executable, including cluster assignments, with no changes in the clustering results between v3 and v5. We have included a relevant statement in the manuscript.

Comment: Fig 5: What do coordinate X and Y represent? Please add the labels (in Fig 5C-D).

Response: XY Cartesian coordinates represent the position of a tissue sample on a glass slide. We have added this clarification to the figure legend and updated the labels in Fig. 5D.

Comment: There is almost no figure description for Fig 5. Please expand, e.g., add a scale bar and a description of the heatmap (what does 0-1 in the scale mean? Normalized expression?).

Response: Thank you for pointing this out. We have added the scale bar and updated the figure legend to include details about the heatmap.

Comment: As the authors mentioned, they do not have the information on the ground truth in this dataset, and they also do not compare APP to alternative clustering algorithms for this dataset. What was the rationale for that?

Response: As we stated above, to encompass the range of real-world scenarios for clustering algorithm usage, both with and without ground truth or domain knowledge, we first demonstrate the method's performance using a dataset with functionally validated ground truth. We then apply the method to a dataset where expert-defined manual gating serves as the ground truth. Next, we test the algorithm on single-cell mRNA expression data and imaging data, which lack predefined ground truth. However, cluster evaluation is enabled using domain knowledge from pathology and immunology, leveraging expression patterns and spatial distribution. Finally, we apply APP clustering in a fully exploratory mode, where no ground truth or pre-existing domain knowledge is available to assess how TCR receptor embeddings cluster with respect to their cognate antigens.

We have now included this general statement in the Results section of our manuscript. A comparison to alternative clustering solutions would require the presence of ground truth, which is not available in this case. Moreover, we did not intend to claim the superiority of APP clustering over other solutions for each data modality.

Comment: The authors say: "Pathology and immunology experts independently assessed and confirmed the adequacy of APP clustering in characterizing and distinguishing meaningful cell populations within the slide tissue, particularly in the context of the human squamous lung carcinoma sample." I am not sure how helpful this assessment is, as it is hard to validate and

anonymous, and I am also not sure how accurately a pathologist can assess the clustering of a rare cell type that might be misclassified. Would it be helpful if the authors examined the composition of similar cancerous tissues in scRNA-seq/spatial RNA-seq databases and confirmed the expression of the identified marker genes? If this is not possible, the authors could add this as a limitation.

***Response*:** Evaluating the quality and validity of clusters derived from spatially resolved data requires domain expertise in pathology and immunology. This assessment must consider both the expression patterns of panel markers and the spatial distribution and co-location of identified cell phenotypes within the tissue context, in this case, human squamous lung carcinoma.

We do not keep the name of the pathologist who evaluated the clustering outcomes anonymous; in fact, we explicitly acknowledged him in the manuscript's Acknowledgments section. To further clarify his credentials, we have now appended his current affiliation next to his name, and explicitly referenced this in the manuscript text. Additionally, an independent immunological assessment was conducted by Dr. Eliver Ghosn's lab, with Dr. Ghosn and a lab member listed as co-authors of this manuscript.

We appreciate the suggestion to use scRNA-seq or spatially resolved RNA-seq as an orthogonal approach to assess cluster quality. We agree that performing such an analysis on the same sample or a serial section would be ideal. However, we do not have access to the sample used in this study for further analysis. Given the inherent heterogeneity of tissue composition, even within the same patient, extrapolating tissue composition from external patient samples could introduce noise rather than providing a robust validation of clustering accuracy. With this in mind, we have added the following statement to the manuscript: "ScRNA-seq or spatially resolved RNA-seq from the same sample or a serial section could serve as an orthogonal approach to assess cluster quality. However, we did not have further access to the sample used in this study for additional analysis."

***Comment*:** Fig 6: Although I find this an interesting approach to partially unravel the decision-making of LLMs, in my opinion, this part of the results is slightly disconnected from the previous findings in Figs 1-5. The data in Fig 6 (and the associated Supplementary Figure) could almost represent a new manuscript. I am not saying it needs to be separated, but it may help to streamline the findings. If the authors want to keep Fig 6, they should benchmark the use of APP with other clustering methods alongside the LLMs to determine whether the performance of APP is comparable to other clustering algorithms.

***Response*:** We thank the reviewer for this suggestion. To help streamline the message we aim to convey in this manuscript, we have added the following statement at the beginning of the Results section: "To encompass the range of real-world scenarios for clustering algorithm usage, both with and without ground truth or domain knowledge, we first demonstrate the method's performance using a dataset with functionally validated ground truth. We then apply the method to a dataset where expert-defined manual gating serves as the ground truth. Next, we test the algorithm on single-cell mRNA expression data and imaging data, which lack predefined ground truth. However, cluster evaluation is enabled using domain knowledge from pathology and immunology, leveraging expression patterns and spatial distribution. Finally, we apply APP clustering in a fully exploratory mode, where no ground truth or pre-existing domain knowledge is available to assess how TCR receptor embeddings cluster with respect to their cognate antigens."

***Comment*:** The authors generate two embeddings for Fig 6, the second embedding being a subset of approximately 4,000 unique TCRs with sequence information for both TCR CDR3α and CDR3β. Could the authors add the rationale for doing this?

***Response*:** We have added the following statement into the relevant method section: " The TCR molecule is a heterodimer that primarily interacts with pMHC through its CDR3a and CDR3b chains. While using paired CDR3a and CDR3b data points is ideal, the high cost of sequencing has led most studies to focus solely on the CDR3b chain. Despite this limitation, the field continues to rely on the CDR3b chain as the primary determinant of TCR specificity, as it is the most highly variable region that contacts pMHC. Accordingly, we have maximized the use of the available dataset by leveraging both CDR3b chain data points and those with paired CDR3a sequences whenever possible."

***Comment*:** Also, similar to previous findings, it is important to know what parameters were used for APP and how robust the results are to changes in these parameters.

***Response*:** APP has only one required user input parameter: the minimum cluster size, which should be set based on the smallest population the user expects to detect in the dataset. Reducing this value introduces smaller clusters, as expected, while other APP clustering outcomes remain unchanged. All other parameters, such as Gaussian smoothing width, are optional. We have set default values optimized for the Calinski-Harabasz index based on the datasets presented in the manuscript. These parameters are user-adjustable to allow

optimization for other data types, guided by the Calinski-Harabasz index. We have now clarified this more explicitly in the Methods section.

We have also updated the GitHub folder with a more detailed parameter description.

Suppl Figures:

Comment**:** Suppl Fig 1 shows an example of decision boundary search for 2D projections in APP. Perhaps the figure legend could be expanded, but otherwise, no issues here.

Response**:** We have expanded the figure legend to include the following information: “The decision boundary begins at the left edge and ends at the right edge of a 2D projection. However, in xy vs yx orientations, the left and right edges differ, leading to distinct decision boundaries. Therefore, xy and yx projections should be analyzed independently.”

Comment**:** Suppl Fig 3 shows an example of discrepancies between data topology and clustering decisions in manual gating. Since this is a UMAP, the numbers on the x and y axes are not particularly meaningful and can be removed. Otherwise, no issues here.

Response**:** We have removed the numerical labels from the x and y axes.

Comment**:** Suppl Fig 4 shows a representation of the label transfer pipeline comparing clustering algorithms. I am not sure why the authors used the word "cartoon." Perhaps they can clarify—this is real data from their clustering, right?

Response**:** To clarify this, we have now adjusted the figure legend accordingly: “Schematic representation of the label transfer pipeline application to quantitative comparison of two clustering algorithms decisions made on the same data set. Here, we used a synthetic dataset consisting of a mixture of Gaussian distributions.”

Comment**:** Suppl Fig 6 shows a UMAP with misclassification by APP and Phenograph compared to manual gating annotations. The labeling is a bit difficult to read and not always clear to which cluster it refers. Also, the numbers on the x and y axes can be removed.

Response**:** We increased the font size of the labels and removed the numbers on the x and y axes.

Comment**:** Suppl Fig 10 shows sequence similarity and amino acid group properties in peptide and TCR embeddings. The figure feels a bit busy and overwhelming with data (true for subsequent supplementary figures as well). Perhaps the authors could decide whether all of the

data is required to show the sequence similarity, and they should also subdivide the figure into more panels than a-c.

***Response*:** We have now subdivided the figure into panels (a–f).

***Comment*:** Suppl Fig 11 shows the absence of common binding motifs in TCR CDR3b sequences for similar epitopes. See comments on Suppl Fig 10.

***Response*:** We have now subdivided the figure into panels (a–e).

***Comment*:** Suppl Fig 12 shows cluster alignment differences between single-class and concatenated embeddings for peptides and TCRs. The labels are very small and can be enlarged. Otherwise, no issues here.

***Response*:** We have increased the font size of the labels.

Discussion

***Comment*:** While APP's ability to assign cluster labels is highlighted, the authors could further discuss how the approach to projection is unique compared to modern methods like, for example, Phenograph.

***Response*:** Please see the third and fourth paragraphs of the Introduction, where we have added some details to bring clarity to this. The main difference is that APP repeatedly restricts focus to a two-dimensional projection of the dataset, other modern clustering methods generally operate in high-dimensional space while using various other techniques to address the curse of dimensionality.

Notably, methods such as PhenoGraph, FlowSOM, and SPADE do not explicitly use any projections whatsoever. PhenoGraph represents the dataset as a mathematical graph built using the k-nearest neighbors of each point, then runs a Louvain community detection algorithm to cluster the graph. FlowSOM builds a two-dimensional grid of nodes (a self-organizing map) which captures topological information of the underlying dataset, then runs a consensus agglomerative hierarchical clustering method on this grid. SPADE downsamples the high-dimensional dataset in order to equalize local density, then performs agglomerative hierarchical clustering on the resulting downsampled "cloud".

***Comment*:** Similar to my comment in the introduction, I think the authors should discuss APP in the context of modern clustering methods (which they used as benchmarks in the results, e.g., in scRNA-seq) and focus less on historical references.

Response**:** While we believe that we have already discussed projection pursuit clustering approaches, such as APP, in the context of modern clustering methods, we have now made this connection more explicit. Please refer to the statements we added to the Conclusions section of the manuscript.

“While the concept of projection pursuit has been around for decades, high-dimensional clustering (such as HDBSCAN, KMeans, and SPADE) has gained more popularity and attention, especially in the context of the computational challenges posed by modern data analysis.”

“In scenarios characterized by sparse or imbalanced data (such as the dataset presented in Figure 2), where there is a high degree of sparsity amid more abundant populations (which can extend to situations involving outliers and/or noise), projection pursuit proves beneficial in identifying pertinent dimensions and enhancing cluster separation. Traditional methods (such as HDBSCAN, KMeans, Phenograph and SPADE) may face challenges in handling sparsity (see Figure 2C) due to a dearth of informative features.”

Comment**:** For findings that did not have "ground truth" data (e.g., the multiplex imaging data), the authors should point out these limitations in the discussion.

Response**:** We have now added the following statements to the Results section:

“To encompass the range of real-world scenarios for clustering algorithm usage, both with and without ground truth or domain knowledge, we first demonstrate the method’s performance using a dataset with functionally validated ground truth. We then apply the method to a dataset where expert-defined manual gating serves as the ground truth. Next, we test the algorithm on single-cell mRNA expression data and imaging data, which lack predefined ground truth. However, cluster evaluation is enabled using domain knowledge from pathology and immunology, leveraging expression patterns and spatial distribution. Finally, we apply APP clustering in a fully exploratory mode, where no ground truth or pre-existing domain knowledge is available to assess how TCR receptor embeddings cluster with respect to their cognate antigens.”

“ScRNA-seq or spatially resolved RNA-seq from the same sample or a serial section could serve as an orthogonal approach to assess cluster quality. However, we did not have further access to the sample used in this study for additional analysis.”

Comment**:** For some of their interpretations, such as stating that they identified novel mechanisms, I would suggest that the authors clarify that follow-up studies with gain- or

loss-of-function experiments need to be done to validate the in situ findings and strengthen these claims.

***Response*:** We have incorporated the following statements into the COVID results section of the manuscript: “Although our new findings might represent a novel mechanism of immune modulation in severe COVID-19, further studies with more patients and different disease states are required to determine whether this is a general mechanism of SARS-CoV-2 infection or a phenotype unique to our small patient cohort.”

And TCR results section: “However, follow-up studies will be necessary to further validate the findings and strengthen these claims.”

***Comment*:** This is a more general comment, but regarding the use of APP and LLMs, the authors could perhaps discuss the limitation that LLM representations may or may not accurately capture the underlying biological process. For example, how do they account for possible biases in LLM embeddings?

***Response*:** We have now added the following statement to the relevant results section of the manuscript: “It is also important to note that while LLMs could potentially be biased by learning patterns from the limited training data provided, we have recently shown [Culka et al., 2024] that, regardless of the grouping approach (e.g., distance-based clustering, LLM, etc.), only a minority of TCRs form pure clusters predominantly composed of peptide-specific TCRs.”

***Comment*:** Minor (and more a personal interest): Could APP also be applied to non-biological high-dimensional datasets?

***Response*:** Yes, there are no inherent limitations to applying APP to clustering other data types; however, as discussed in our manuscript, it is most suitable for tasks similar to cell phenotyping. Testing APP’s performance on non-biological data modalities is beyond the scope of this manuscript.
