# Supplementary material for: Lifting the curse from high-dimensional data: automated projection pursuit clustering for a variety of biological data modalities
Source: Gigascience. 2025 May 29;14:giaf052. doi: 10.1093/gigascience/giaf052 (PMC12121483; doi:10.1093/gigascience/giaf052)
Supplement: giaf052_Authors_Response_To_Reviewer_Comments_original_submission [file giaf052_authors_response_to_reviewer_comments_original_submission.pdf]

Author's Response To Reviewer Comments

Dear Editorial Board,
Below are our responses to the reviewers' comments. We sincerely appreciate the reviewers' thoughtful and thorough evaluation. We have addressed all concerns to the best of our ability and look forward to your feedback.
For your convenience, we have highlighted the changes in the revised manuscript in blue.

Reviewer #1:

Comment: The authors reference the 'Curse of Dimensionality' in both the title and introduction but do not provide a detailed explanation of the issue. A more thorough discussion of this problem would help readers better understand how the proposed approach addresses and mitigates it.

Response: We have added the explanation to the manuscript: "The well-known phrase "the curse of dimensionality" was coined by Richard Bellman [Morgenstern, D., & Bellman, R. 1962] and describes challenges that arise when working with high-dimensional data. A key issue is that the amount of data needed to obtain reliable results grows exponentially with the number of dimensions. Data analysis often depends on identifying regions where data points form meaningful clusters, but in high-dimensional spaces, such structures become sparse, making effective analysis increasingly difficult."

Comment: The presented pipeline is based on the custom procedure for 2d clustering/decision boundary construction. This procedure is described to some extent, but the underlying logic is could be explained in more details. For now, it seems counter-intuitive that the same projections should be analyzed twice (I mean, different separating boundaries are constructed for x-y and y-x plots).

Response: We have added the explanation to the manuscript: "In our algorithm, the same projections are analyzed twice to determine a 2D cluster boundary in (x, y)-space: first to identify the x-projection of the boundary and then to determine its y-projection." We also added a clarification to the Supplementary Figure 1 legend: "Supplementary Figure 1. Example of an optimal decision boundary search for one of the 2D data projections. The decision boundary begins at the left edge and ends at the right edge of a 2D projection. However, in xy vs yx orientations, the left and right edges differ, leading to distinct decision boundaries. Therefore, xy and yx projections should be analyzed independently."

Comment: Additionally, the "initial distribution density H" is not defined.

Response: We have appended the explanation "—that is, the initial distribution density H" to the end of the phrase: "Otherwise, for each 2D (x,y) projection mapped onto a unit square (side length of 1), we build the Gaussian-smoothed histogram H(x,y) of the data points." The revised sentence in the manuscript now reads: "Otherwise, for each 2D (x,y) projection mapped onto a unit square (side length of 1), we build the Gaussian-smoothed histogram H(x,y) of the data points— that is, the initial distribution density H."

Comment: Overall, there seem to be a lot of existing algorithms for construction of optimal separating boundary or even automatic clustering of data in 2D space. It is unclear what advantages and disadvantages they have compared to the procedure developed by the authors (probably higher computational cost?).

Response: Most existing clustering algorithms operate in high-dimensional space rather than in 2D, making them vulnerable to the curse of dimensionality. Previous implementations of projection pursuit clustering algorithms sequentially operate on 2D projections; however, as discussed in the Introduction of our manuscript, their criteria for selecting the most informative low-dimensional projections was not optimized for identifying decision boundaries with the lowest data density between clusters-a crucial aspect of cell phenotyping. Instead, previous implementations of the projection pursuit algorithm primarily focused on identifying projections with "high" information content, such as those that deviate from normality or

uniformity.
We have now emphasized this point in the manuscript with the following text: “The concept of exhaustively exploring low-dimensional projections of high-dimensional data has existed for a few decades. Historically, efforts have been made to systematically explore low-dimensional projections, known as the "grand tour" [Cook et al., 1995], or to optimize specific criteria for identifying informative projections, such as those that reveal structure in the data by deviating from normality or uniformity [Friedman et al., 1974]. However, challenges in determining the optimal criterion and the computational complexities associated with processing numerous low-dimensional projections have hindered the widespread adoption of projection pursuit methods for data clustering tasks.”

Comment: The authors tested their approach against three state-of-the art clustering algorithms (HDBSCAN, KMeans and Phenograph), but they were operating in the original high-dimensional space. The possibility of using these algorithms in 2D projections seems obvious and at least should be discussed.

Response: While HDBSCAN, KMeans, and Phenograph clustering can be applied to individual 2D projections, these algorithms do not include a mechanism to systematically traverse the entire set of 2D projections. They lack recursive steps that would score and evaluate each 2D projection to iteratively refine data splits for downstream processing, which is necessary for effectively identifying final clusters in a multidimensional dataset. Thus, the only way to directly compare the final clusters produced by APP with those from HDBSCAN, KMeans, and Phenograph is to run HDBSCAN, KMeans, and Phenograph on the full high-dimensional dataset rather than on individual 2D projections. Since a multidimensional point space can be uniquely represented by its complete set of 2D projections, it follows that comparing APP with modern multidimensional clustering algorithms (e.g., HDBSCAN, KMeans, and Phenograph) within these 2D projections should be sufficient.
We added the following explanation to the manuscript: “Since a multidimensional point space can be uniquely represented by its complete set of 2D projections, it follows that comparing APP with modern multidimensional clustering algorithms (e.g., HDBSCAN, KMeans, and Phenograph) within these 2D projections should be sufficient.”

Comment: In some cases, clusters may be well-separated in 3D space but appear mixed in 2D projections (such as xy, xz, yz). This can occur, especially when there are many clusters, but the situation might be improved through proper rotation of the coordinate system. Could the authors comment on the potential for this improvement? Additionally, an optimal decision boundary should ideally be independent of in-plane rotations, including the interchange of x- and y- axes. Could the authors suggest possible ways to algorithm modification to satisfy this requirement?

Response: We thank the reviewer for this insightful comment. Indeed, in some cases clusters that are well-separated in higher-dimensional space may appear mixed when projected onto certain 2D planes. To address this, proper rotation of the coordinate system may help uncover projections that better reveal separation. While our current approach does not explicitly incorporate rotation optimization, one potential extension could involve employing rotation search methods such as those used in Projection Pursuit to identify projections that maximize separation between clusters.
Regarding the independence of the decision boundary from in-plane rotations and axis interchange, we agree that this is an important consideration. One potential modification would be to apply symmetry operations (e.g., swapping the x- and y-axes) during the decision boundary search and averaging the resulting decision boundaries to enforce rotational invariance. Additionally, incorporating optimization steps that account for rotational symmetry during boundary determination could help ensure robustness.
We will consider these suggestions for future algorithmic improvements and exploration in subsequent studies. Additionally, we have included the following statement in the Discussion section: “Also, in some cases clusters that are well-separated in higher-dimensional space may appear mixed when projected onto certain 2D planes. To address this, proper rotation of the coordinate system may help uncover projections that better reveal separation. While our current approach does not explicitly incorporate rotation optimization, one potential future extension could involve employing rotation search methods such as those used in projection pursuit to identify projections that maximize separation between clusters.”

Minor points:

Comment: Figure 1C appears overwhelming due to the large number of 2D projection plots, which seem disorganized. The authors might consider grouping the plots for better clarity. Additionally, the text on the plots is too small. Moreover, there seems to be no need to present six projections in 3D space for the identified clusters.

Response: We simplified panel C and updated the Figure 1C legend accordingly. Additionally, we increased the text font size for better readability.

Comment: Equation (1): The text mentions a square root, but the expression is raised to the power of 0.1. Should it be 0.5?

Response: The key point is that Mann's original formula for the total number of two-dimensional bins includes a power of 0.2. Taking the square root of this power results in a power of 0.1 in our Equation (1), ensuring that the calculation is correct.

Comment: The sentence "If the number of cells at the input to the recursion step is less than 2 * min_cluster_size" might be clearer if "the number of cells" is changed to "the number of points."

Response: We have incorporated this suggestion.

Reviewer #2:

Comment: The authors should present the silhouette of the clusters.

Response: We thank the reviewer for this suggestion and have updated the manuscript accordingly. Please refer to Figures 2 and 3, as well as the legend of Figure 4.

Comment: Paper should be revised for minor typos.

Response: We have revised the manuscript accordingly.

Reviewer #3:

Comment: However, some control experiments are not fully explained. For example, the optimization of APP parameters and the limited comparison with other clustering algorithms could be expanded.

Response: APP has only one required user input parameter: the minimum cluster size, which should be set based on the smallest population the user expects to detect in the dataset. Reducing this value introduces smaller clusters, as expected, while other APP clustering outcomes remain unchanged. All other parameters, such as Gaussian smoothing width, are optional. We have set default values optimized for the Calinski-Harabasz index based on the datasets presented in the manuscript. These parameters are user-adjustable to allow optimization for other data types, guided by the Calinski-Harabasz index. We have now clarified this more explicitly in the Methods section.
We have also updated the GitHub folder with a more detailed parameter description.
We now include comparisons to additional commonly cited clustering methods in flow cytometry, beyond PhenoGraph: flowSOM (1,310 Google Scholar citations since 2021) and SPADE (272 Google Scholar citations since 2021). Please refer to the updated Figure 2.

Comment: Additionally, while ground-truth validation is used for some datasets, others rely on expert evaluations, which can introduce bias.

Response: Unfortunately, the current state of the art lacks ground-truth validation for assessing and confirming that identified populations are functionally distinct. In most cases, expert evaluation serves as the “gold standard.” We acknowledge that this introduces bias and have explicitly discussed it in the Conclusions section of the manuscript. In this study, we introduced a method to generate functionally

validated ground truth; however, we also recognize that this is a resource-intensive endeavor, making it impractical to expect it to be routinely performed for every new experiment.
“In many biological real-world datasets, the availability of a clear "ground truth" can be challenging. As illustrated in the examples presented, reliance on domain experts' knowledge-driven clustering or clustering done with widely-adopted approaches serves as a substitute for ground truth. While expert-driven clustering provides a valuable reference point, a more accurate (albeit labor-intensive) method for assessing clustering performance involves conducting functional tests on groups of cells assigned to the same cluster. By observing the functional "purity" and homogeneity of a given cluster compared to other cell clusters in the sample, researchers can achieve a more precise evaluation of the clustering results.”

Comment: The total sample size (six healthy controls and six COVID-19 patient) analyzed in the paper appears small for drawing robust conclusions, especially considering the biological variability in human samples. However, it's quite understandable that the number of samples is limited due to the availability of biological samples and the high cost associated of experimental techniques.

Response: We appreciate the reviewers’ feedback and their understanding regarding the relatively low number of samples, which is due to the limited access to this type of biological material and the relatively high cost of the associated experiments. We agree with the reviewer and have considered the biological variability among COVID-19 patients. For example, the six COVID-19 samples analyzed here were collected from patients with severe disease in the ICU, and we intentionally avoided mixing samples from COVID-19 patients with different disease severity. We recently showed that despite the variability among COVID-19 patients, the patients in ICU with severe disease remain distinguishable from healthy controls using flow cytometry to characterize major immune lineages (see Eddins et al., Blood Adv. 2023). Nonetheless, while six samples per group may be relatively low for deriving strong biological conclusions (due to potential sample-to-sample heterogeneity, and we have adjusted our statement accordingly), from a statistical perspective, a cohort of twelve samples is sufficient to assess the performance of the clustering and cluster-matching pipelines.
We have incorporated the relevant statements in the revised manuscript.

Comment: The study provides strong evidence for APP's ability to identify meaningful clusters and compares it with other clustering methods. However, some conclusions need stronger evidence. For instance, the claim that APP handles sparse data better than other methods is not consistently proven across all datasets.

Response: The overall theoretical conclusion regarding the superiority of projection pursuit over high-dimensional methods in clustering sparse cell populations is derived from the definition of the curse of dimensionality and the well-established fact that, to achieve a given accuracy with typical high-dimensional methods, the sample size must increase exponentially with the number of dimensions. This leads to the logical conclusion that sparse cell populations are particularly susceptible to the effects of the curse of dimensionality. For further illustration, please refer to Supplementary Figure 1 in [Meehan et al., 2019], which we also cited in our manuscript.
To illustrate this in practice, we updated Figure 2 (new panel C) to show the relationship between percent misclassification and the abundance of cell populations in the sample. For "purity," we present this data using samples with functionally validated ground truth labels.

Comment: The statistical methods used in the study, such as the Calinski-Harabasz index, are suitable for the types of data analyzed. PCA and Gaussian smoothing are reasonable choices for reducing data complexity and handling noise. However, some choices, like the beta parameter in APP, seem to be based on trial and error and need more justification.

Response: We have included the explanation in the manuscript: "APP has only one required user input parameter: the minimum cluster size, which should be set based on the smallest population the user expects to detect in the dataset. Reducing this value introduces smaller clusters, as expected, while other APP clustering outcomes remain unchanged. All other parameters, such as Gaussian smoothing width, are optional. We have set default values optimized for the Calinski-Harabasz index based on the datasets presented in the manuscript. These parameters are user-adjustable to allow optimization for other data types, guided by the Calinski-Harabasz index.”

We have also updated the GitHub folder with a more detailed parameter description.

Comment: Additionally, the study reports accuracy metrics but could include other measures like F1-score to give a more complete picture of performance.

Response: We thank the reviewer for this suggestion and have now included the F1-measure in Figures 2 and 3.

Comment: There are minor grammar issues and repetitive phrases that could be cleaned up. E.g.,
- P.2: While the latter challenge is computational rather than a fundamental scientific limitation, it can be a serious practical hurdle.
- P.3: These insights include evaluating hypotheses regarding the existence of a binding motif between in CDR3b of TCRs that recognize the same peptide ...
- P.3: To gain a deeper understanding ... we initially applied ...
- etc.

Response: We have revised the manuscript accordingly.

Comment: Overall, moderate editing would improve the readability and flow of the manuscript.

Response: We have revised the manuscript accordingly.

Reviewer #4:

Comment: In my opinion, in some parts, the manuscript is almost overwhelming with different applications and datasets that could be explained in more detail. Potentially, Figure 6 could even be split into a separate manuscript to streamline the different applications of APP. However, this can be addressed in the revised version.

Response: There is a clear logic behind how the material is presented: we first demonstrate the method's performance using a dataset with functionally validated ground truth (GFP mice data). Next, we apply the method to a dataset where expert-defined manual gating serves as the "ground truth" (COVID dataset). We then apply the algorithm to single-cell mRNA expression and imaging data, where no predefined ground truth is available. However, clustering outcomes are assessed using domain knowledge from pathology and immunology. For the imaging data, both expression patterns and co-location/spatial distribution are used to evaluate cluster quality. Finally, we apply APP clustering in a fully exploratory mode, where no ground truth is available and no immediate assessment of clustering outcomes is possible due to the lack of pre-existing domain knowledge about how TCR receptor embeddings should cluster with respect to their cognate antigens (TCR data). This approach covers the range of real-world scenarios for using clustering algorithms, whether in the presence or absence of ground truth and domain knowledge.
We have now included this general statement in the Results section of our manuscript.

Comment: I validated that all datasets and code are available. Some descriptions of the data could be improved, e.g., more detailed README files, etc. (see below). Perhaps the datasets could also be uploaded to permanent repositories in addition to the GitHub link to ensure permanent accessibility.

Response: Previously unavailable datasets are now shared on Zenodo, accompanied by corresponding readme files. Please refer to the updated version of the Data Availability section in the manuscript.

Introduction

Comment: While the limitations and objectives are clearly defined in the introduction, I believe the authors could expand on alternative, newer methods that people use for clustering. The authors largely describe

the historical initial work by Friedman et al., 1982; Huber, 1985, which is 40-50 years old. For instance, newer clustering algorithms might be relevant to include, along with mentioning their limitations. For example, the authors benchmark APP with algorithms such as HDBSCAN and K-means, but these are not introduced. Additionally, mentioning graph-based clustering and dimensionality reduction techniques such as UMAP and t-SNE, and how they address challenges in high-dimensional clustering and have become standard for scRNA-seq data analysis, might be helpful.

Response: We have incorporated these suggestions and revised the Introduction accordingly.

Comment: Since much of the manuscript is about the validation of APP with real-world data, the authors may consider including a bit more information on why they chose these datasets and the different modalities (e.g., flow cytometry, scRNA-seq, multiplex imaging). I appreciate the diversity of the data, but expanding a little on the rationale would be helpful.

Response: This is a very useful suggestion; thank you. We have now included the rationale for selecting the datasets at the beginning of the Results section: "To encompass the range of real-world scenarios for clustering algorithm usage, both with and without ground truth or domain knowledge, we first demonstrate the method's performance using a dataset with functionally validated ground truth. We then apply the method to a dataset where expert-defined manual gating serves as the ground truth. Next, we test the algorithm on single-cell mRNA expression data and imaging data, which lack predefined ground truth. However, cluster evaluation is enabled using domain knowledge from pathology and immunology, leveraging expression patterns and spatial distribution. Finally, we apply APP clustering in a fully exploratory mode, where no ground truth or pre-existing domain knowledge is available to assess how TCR receptor embeddings cluster with respect to their cognate antigens."

Comment: Additionally, the authors may clarify the novelty or major advantage of the APP clustering tool. For instance, the authors mention that APP is an alternative clustering approach, but they could perhaps elaborate on its major strengths or novelty in the last paragraph.

Response: We thank the reviewer for this suggestion. In the last paragraph of the Introduction, we have now highlighted that the major advantage and novelty of APP clustering lie in its ability to automatically uncover meaningful structures in high-dimensional data while mitigating the curse of dimensionality, a common challenge in high-dimensional clustering. Unlike traditional projection pursuit, where an analyst manually explores projections to identify patterns, APP automates this process by recursively finding low-dimensional projections with the smallest data density between clusters and continuing the analysis until no further splits are detected.

Comment: The last three paragraphs of the introduction discuss the findings of this manuscript. I would suggest shortening this part into a single last paragraph in the introduction.

Response: We followed the recommendation and condensed the last three paragraphs into a single, more concise paragraph.

Minor comments:

Comment: The authors could use a reference instead of a link in the introduction when referring to supervised UMAP.

Response: To our knowledge, there is no peer-reviewed paper specifically dedicated to supervised UMAP. However, an explanation has been provided by the first authors of the original UMAP paper, which can be found here: https://github.com/lmcinnes/umap/issues/135.
"We have not written a paper on the subject unfortunately -- there really isn't enough material for one. The idea is pretty straightforward (intersecting fuzzy simplicial sets / taking limits) and largely self-evident. The implementation is publicly available, and hopefully takes care of the technical matters of "making it work in practice". I would certainly welcome suggestions for where code comments might be added to make things clearer."

Comment: It may not be ideal to use "APP" for the name of the software in the context of models of Alzheimer's disease, as most AD researchers associate APP with Amyloid Precursor Protein.

Response: We appreciate the reviewer bringing this up and are aware of the term APP in the neuroscience field. However, we believe that for a broader readership, particularly computational scientists, the term APP is more commonly associated with "Application" (or "app" for short). Therefore, we would like to retain the name APP for our algorithm.
Methods

Comment: Since the authors generated some of their own data and used publicly available datasets, it might be helpful to add a table with an overview of all datasets they used. It took me some time to understand which datasets were generated in this study and which ones were taken from a public database. Perhaps the authors could explain why they decided to include their own data if the main purpose is to benchmark APP against other clustering software.

Response: We thank the reviewer for this suggestion. We have reformatted the Data Availability section into a table to provide the requested information. Currently, there is a lack of datasets with real ground truth, and most datasets, particularly in flow cytometry, imaging, and scRNA-seq, are accompanied by user-defined ground truth based on field-specific best practices for data preprocessing and annotation. Our decision to include our own data was driven by our commitment to adhering to what we believe are the best practices for preprocessing and annotating cell populations. As illustrated in Supplementary Figure 3, generating ground truth cell population annotations is a challenging task.

Comment: For the flow cytometry data, the authors could add the manufacturer, species, and dilution of the antibody.

Response: We have now incorporated this information into the manuscript.

Comment: More information about the antibodies and their dilution would also be beneficial for the multiplex imaging data.

Response: We have added clone names and working concentrations to the relevant Materials and Methods section of the manuscript.

Comment: The CyTOF methods are not properly described. Although the authors refer to previous studies, they should consider providing a bit more detail.

Response: Although the CyTOF data used in our manuscript was previously published and processed elsewhere, and we are only reusing it, we have now added the following description in the relevant methods section: “Whole blood was collected from consenting healthy human donors (N = 10), and peripheral blood mononuclear cells (PBMCs) were isolated and stained with a metal-conjugated 38-parameter mAb panel (see Table S1 in [Toghi Eshghi et al., 2019]), enabling the comparison of 28 immune cell subset frequencies [Toghi Eshghi et al., 2019]. Data were acquired using the Helios™ CyTOF® system (Fluidigm, South San Francisco, CA).”

Comment: Why did the authors use Seurat v3? The current version is v5. Perhaps the authors could double-check whether their code is still executable with v5.

Response: The original data analysis, as described in [Zhou et al., 2020], was conducted using Seurat v3. To avoid potential discrepancies due to version differences, we adopted the same version. However, we have now tested Seurat v5 and confirmed that the code remains executable, including cluster assignments. We have included a relevant statement in the manuscript.

Comment: The description of the scRNA-seq analysis is quite brief; however, the coding data is available on GitHub. In case the authors have not done so, it would be helpful to deposit the data in a permanent database such as Zenodo (since the GitHub data could be altered or deleted).

Response: We thank the reviewer for this suggestion. We have reformatted the Data Availability section to provide the requested information.

Comment: All raw data and code appear to be available on GitHub. The authors could perhaps elaborate on the README files in the main folder to provide more detailed instructions and descriptions of the project.

Response: The README in the main folder has been augmented with a more detailed description of the project and repository contents.

Comment: Fig 2:
In Figure 2, the authors compare APP performance to widely used clustering methods in flow cytometry of immune cells from WT and RAG KO mice. The experimental design is very elegant, mixing the population of cells in different proportions to validate individual cell types, but I think this needs to be better explained in the results. Especially since most scientists reading this manuscript might have a bioinformatics background and may not be familiar with the mouse model and the experimental setup.

Response: We thank the reviewer for the positive comments. We have expanded on this experimental approach in the text to include the following explanation: Mice lacking the RAG1 gene (i.e., RAG-KO) are deficient in immune cells known as B and T lymphocytes, while still developing all other major immune lineages, including myeloid cells and NK cells. Since the wild-type (WT) mice, expressing the RAG1 gene can develop both B and T lymphocytes, we intentionally mixed WT cells from a GFP+ mouse, which expresses green fluorescent protein on their lymphocytes, with cells from the RAG-KO mouse, which does not contain lymphocytes (i.e., WT-GFP mixed with RAG-KO). We used this experimental approach to define a biological and technical ground truth. For example, any B and T cells identified by our new APP pipeline should express the green (GFP) protein since they can only come from the WT-GFP mice and not from the RAG-KO mice. If the pipeline detects any B and T lymphocytes lacking the green/GFP protein, these events would be considered as "misclassification" by the APP pipeline.

Comment: Also, the clustering algorithms that were used as a comparison have several parameters that can be adjusted. How were those chosen? Did any of them lead to better/worse clustering?

Response: We used the default input parameters recommended in available tutorials (e.g., Seurat: [https://satijalab.org/seurat/articles/pbmc3k_tutorial.html]) for the given data type, as parameter adjustments did not significantly improve clustering. This information has now been included in the relevant methods section.

Comment: Perhaps in a comparison like this, it would be helpful to briefly mention the computational performance of the methods. For instance, how long did each algorithm take to run under which computer configuration?

Response: Although we have now included these statistics for the dataset in Fig. 2 (see panel 2B), we emphasize that achieving robust clustering results is more critical than obtaining rapid but inaccurate outcomes.

Comment: It is not clear to me how they generated the UMAP in Fig 2C. Is this based on the APP clustering? And then were the labels transferred from the other clustering algorithms? A bit more explanation is needed.

Response: In response to these questions we now adjusted the Fig. 2D (previously 2C) legend accordingly: "The misclassification for the 50/50 mix is visually represented in black color using the automated label transfer pipeline, as detailed in Supplementary Figure 4. Here, the performance of each of the six clustering algorithms is assessed against the ground truth labels (functionally distinct cell types) used to construct the supervised UMAP embedding."

Comment: Did the authors perform any statistical analysis on the quantified data? The plots look convincing but should be tested using appropriate statistical tests in Fig 2D and E.

Response: In response to this request, we have updated Fig. 2 accordingly. The performance of the APP algorithm in cell population classification is now evaluated using total and per-cell-type population misclassification, F1-measure, and Silhouette coefficient, and is compared to state-of-the-art clustering algorithms.

Comment: Fig 3: The authors should add statistical analysis to Fig 3 A-D and F.

Response: In response to this request, we have updated Fig. 3 accordingly. The performance of the APP algorithm in cell population classification is now evaluated using total misclassification, F1-measure, and Silhouette coefficient and compared to Phenograph. Per-cell-type population misclassification is shown in Supplementary Figure 7.

Comment: The authors convincingly show that the label-transfer pipeline is very accurate when training on one healthy control sample, and that there were more discrepancies with COVID samples. I think the authors could expand a little more on the diverse responses of COVID patients and cellular shifts, which could also include cellular transition states. Also, intragroup variability is likely higher in COVID patients.

Response: We thank the reviewer for raising this important point on biological variables in COVID-19 patients, ranging from asymptomatic to mild, moderate, and severe patients in the ICU. We have revised our manuscript to better describe the patients analyzed here for this method's paper. All six COVID-19 samples analyzed here were collected from patients with severe disease in the ICU, and we intentionally avoided mixing samples from COVID-19 patients with different disease severity, which could add additional biological variables. However, despite analyzing samples from the same disease severity (i.e., ICU patients), we agree with the reviewer that COVID-19 disease induces changes in immune cells, leading to transitional cellular states. These cellular states are dynamic and can vary across patients depending on various factors, including patient demographics, comorbidity, viral load, timing of infection to analysis, etc. Thus, it is expected that samples from COVID-19 patients would show more discrepancy compared to healthy controls. In any event, we recently showed that despite the variability among COVID-19 patients, the patients in ICU with severe disease remain distinguishable from healthy controls using flow cytometry to characterize major immune lineages and cellular states (see Eddins et al., Blood Adv. 2023).
We have incorporated the relevant information into the manuscript.

Comment: The authors suggest that "these findings highlight new mechanisms of SARS-CoV-2-induced immuno-modulation that underlie the COVID-19 immunopathology in hospitalized patients," which might be a slight overstatement. They detect a subpopulation of monocytes, which is interesting. However, there is no data indicating a new mechanism. Toning this statement down would solve the issue.

Response: We agree with the reviewer and have now revised the manuscript to highlight that although our new findings might represent a novel mechanism of immune modulation in severe COVID-19, further studies with more patients and different disease states are required to determine whether this is a general mechanism of SARS-CoV-2 infection or a phenotype unique to our small patient cohort.

Comment: Fig 4: The scRNA-seq clustering in Seurat depends on the parameters chosen. Did the authors use default parameters?

Response: Parameters were selected based on the Seurat tutorial (https://satijalab.org/seurat/articles/pbmc3k_tutorial.html), using default settings with a resolution of 0.5. We have added this information to the relevant Methods section.

Comment: The authors claim that naïve and memory CD4 T cells do not show any distinction in clustering in any pairwise dimensions explored by APP but refer to Supplementary Data without specifying where in the Supplementary Data this information can be found. Please add a specific reference to where this information can be found.

Response: Although we believe this supplementary data was uploaded to the journal server during the submission process, we have now uploaded it to Zenodo (https://zenodo.org/records/14874269) and provided a link in the manuscript.

Comment: Did the authors examine CD45RA/PTPRC and CD62L/SELL levels in their PBMC RNA-seq data? These markers are usually used to distinguish naïve and memory CD4 T cells.

Response: We did; however, in this dataset, there was no significant difference in PTPRC and SELL levels among the T cell clusters. We have attached a figure to support this statement.

Comment: For the scRNA-seq dataset, it would be helpful to compare the performance in terms of the time required for clustering. scRNA-seq datasets can have millions of cells, and Seurat may face challenges with conventional analysis. How did APP perform compared to Louvain clustering in terms of speed? Could the authors also test it on a larger dataset?

Response: We thank the reviewer for this suggestion and have incorporated the following information into the manuscript: "To assess the computational complexity of the APP algorithm, which is generally O(N^2), where N is the number of dimensions, we recorded the clustering time for APP versus Seurat v5 using a MacBook Pro with a 2.3 GHz Dual-Core Intel Core i5 and 8 GB memory. For the PBMC dataset (containing 2700 cells), Seurat clustering (resolution = 0.5) took 1–2 seconds in real time. APP, with 10 PCs and a minimum cluster size of 10, took 11m 19.952s real time, 26m 6.587s user time, and 2m 43.988s system time. APP, with 10 PCs and a minimum cluster size of 100, took 2m 16.023s real time, 5m 47.742s user time, and 0m 34.924s system time."

Comment: Perhaps the authors could check whether the clustering algorithm in Seurat was updated between v3 and v5 (the most current version). If not, they could consider adding this information to the methods.

Response: The original data analysis for the 5XFAD dataset, as described in [Zhou et al., 2020], was conducted using Seurat v3. To ensure consistency and avoid potential discrepancies due to version differences, we adopted the same version for scRNAseq data processing within this manuscript. However, we have now tested Seurat v5 and confirmed that the code remains executable, including cluster assignments, with no changes in the clustering results between v3 and v5. We have included a relevant statement in the manuscript.

Comment: Fig 5: What do coordinate X and Y represent? Please add the labels (in Fig 5C-D).

Response: XY Cartesian coordinates represent the position of a tissue sample on a glass slide. We have added this clarification to the figure legend and updated the labels in Fig. 5D.

Comment: There is almost no figure description for Fig 5. Please expand, e.g., add a scale bar and a description of the heatmap (what does 0-1 in the scale mean? Normalized expression?).

Response: Thank you for pointing this out. We have added the scale bar and updated the figure legend to include details about the heatmap.

Comment: As the authors mentioned, they do not have the information on the ground truth in this dataset, and they also do not compare APP to alternative clustering algorithms for this dataset. What was the rationale for that?

Response: As we stated above, to encompass the range of real-world scenarios for clustering algorithm usage, both with and without ground truth or domain knowledge, we first demonstrate the method's performance using a dataset with functionally validated ground truth. We then apply the method to a dataset where expert-defined manual gating serves as the ground truth. Next, we test the algorithm on single-cell mRNA expression data and imaging data, which lack predefined ground truth. However, cluster evaluation is enabled using domain knowledge from pathology and immunology, leveraging expression patterns and spatial distribution. Finally, we apply APP clustering in a fully exploratory mode, where no ground truth or pre-existing domain knowledge is available to assess how TCR receptor embeddings cluster

with respect to their cognate antigens.
We have now included this general statement in the Results section of our manuscript. A comparison to alternative clustering solutions would require the presence of ground truth, which is not available in this case. Moreover, we did not intend to claim the superiority of APP clustering over other solutions for each data modality.

Comment: The authors say: "Pathology and immunology experts independently assessed and confirmed the adequacy of APP clustering in characterizing and distinguishing meaningful cell populations within the slide tissue, particularly in the context of the human squamous lung carcinoma sample." I am not sure how helpful this assessment is, as it is hard to validate and anonymous, and I am also not sure how accurately a pathologist can assess the clustering of a rare cell type that might be misclassified. Would it be helpful if the authors examined the composition of similar cancerous tissues in scRNA-seq/spatial RNA-seq databases and confirmed the expression of the identified marker genes? If this is not possible, the authors could add this as a limitation.

Response: Evaluating the quality and validity of clusters derived from spatially resolved data requires domain expertise in pathology and immunology. This assessment must consider both the expression patterns of panel markers and the spatial distribution and co-location of identified cell phenotypes within the tissue context, in this case, human squamous lung carcinoma.
We do not keep the name of the pathologist who evaluated the clustering outcomes anonymous; in fact, we explicitly acknowledged him in the manuscript's Acknowledgments section. To further clarify his credentials, we have now appended his current affiliation next to his name, and explicitly referenced this in the manuscript text. Additionally, an independent immunological assessment was conducted by Dr. Eliver Ghosn's lab, with Dr. Ghosn and a lab member listed as co-authors of this manuscript.
We appreciate the suggestion to use scRNA-seq or spatially resolved RNA-seq as an orthogonal approach to assess cluster quality. We agree that performing such an analysis on the same sample or a serial section would be ideal. However, we do not have access to the sample used in this study for further analysis. Given the inherent heterogeneity of tissue composition, even within the same patient, extrapolating tissue composition from external patient samples could introduce noise rather than providing a robust validation of clustering accuracy. With this in mind, we have added the following statement to the manuscript: "ScRNA-seq or spatially resolved RNA-seq from the same sample or a serial section could serve as an orthogonal approach to assess cluster quality. However, we did not have further access to the sample used in this study for additional analysis."

Comment: Fig 6: Although I find this an interesting approach to partially unravel the decision-making of LLMs, in my opinion, this part of the results is slightly disconnected from the previous findings in Figs 1-5. The data in Fig 6 (and the associated Supplementary Figure) could almost represent a new manuscript. I am not saying it needs to be separated, but it may help to streamline the findings. If the authors want to keep Fig 6, they should benchmark the use of APP with other clustering methods alongside the LLMs to determine whether the performance of APP is comparable to other clustering algorithms.

Response: We thank the reviewer for this suggestion. To help streamline the message we aim to convey in this manuscript, we have added the following statement at the beginning of the Results section: "To encompass the range of real-world scenarios for clustering algorithm usage, both with and without ground truth or domain knowledge, we first demonstrate the method's performance using a dataset with functionally validated ground truth. We then apply the method to a dataset where expert-defined manual gating serves as the ground truth. Next, we test the algorithm on single-cell mRNA expression data and imaging data, which lack predefined ground truth. However, cluster evaluation is enabled using domain knowledge from pathology and immunology, leveraging expression patterns and spatial distribution. Finally, we apply APP clustering in a fully exploratory mode, where no ground truth or pre-existing domain knowledge is available to assess how TCR receptor embeddings cluster with respect to their cognate antigens."

Comment: The authors generate two embeddings for Fig 6, the second embedding being a subset of approximately 4,000 unique TCRs with sequence information for both TCR CDR3α and CDR3β. Could the authors add the rationale for doing this?

Response: We have added the following statement into the relevant method section: " The TCR molecule is a heterodimer that primarily interacts with pMHC through its CDR3a and CDR3b chains. While using paired CDR3a and CDR3b data points is ideal, the high cost of sequencing has led most studies to focus solely on the CDR3b chain. Despite this limitation, the field continues to rely on the CDR3b chain as the primary determinant of TCR specificity, as it is the most highly variable region that contacts pMHC. Accordingly, we have maximized the use of the available dataset by leveraging both CDR3b chain data points and those with paired CDR3a sequences whenever possible."

Comment: Also, similar to previous findings, it is important to know what parameters were used for APP and how robust the results are to changes in these parameters.

Response: APP has only one required user input parameter: the minimum cluster size, which should be set based on the smallest population the user expects to detect in the dataset. Reducing this value introduces smaller clusters, as expected, while other APP clustering outcomes remain unchanged. All other parameters, such as Gaussian smoothing width, are optional. We have set default values optimized for the Calinski-Harabasz index based on the datasets presented in the manuscript. These parameters are user-adjustable to allow optimization for other data types, guided by the Calinski-Harabasz index. We have now clarified this more explicitly in the Methods section.
We have also updated the GitHub folder with a more detailed parameter description.

Suppl Figures:

Comment: Suppl Fig 1 shows an example of decision boundary search for 2D projections in APP. Perhaps the figure legend could be expanded, but otherwise, no issues here.

Response: We have expanded the figure legend to include the following information: "The decision boundary begins at the left edge and ends at the right edge of a 2D projection. However, in xy vs yx orientations, the left and right edges differ, leading to distinct decision boundaries. Therefore, xy and yx projections should be analyzed independently."

Comment: Suppl Fig 3 shows an example of discrepancies between data topology and clustering decisions in manual gating. Since this is a UMAP, the numbers on the x and y axes are not particularly meaningful and can be removed. Otherwise, no issues here.

Response: We have removed the numerical labels from the x and y axes.

Comment: Suppl Fig 4 shows a representation of the label transfer pipeline comparing clustering algorithms. I am not sure why the authors used the word "cartoon." Perhaps they can clarify—this is real data from their clustering, right?

Response: To clarify this, we have now adjusted the figure legend accordingly: "Schematic representation of the label transfer pipeline application to quantitative comparison of two clustering algorithms decisions made on the same data set. Here, we used a synthetic dataset consisting of a mixture of Gaussian distributions."

Comment: Suppl Fig 6 shows a UMAP with misclassification by APP and Phenograph compared to manual gating annotations. The labeling is a bit difficult to read and not always clear to which cluster it refers. Also, the numbers on the x and y axes can be removed.

Response: We increased the font size of the labels and removed the numbers on the x and y axes.

Comment: Suppl Fig 10 shows sequence similarity and amino acid group properties in peptide and TCR embeddings. The figure feels a bit busy and overwhelming with data (true for subsequent supplementary figures as well). Perhaps the authors could decide whether all of the data is required to show the sequence similarity, and they should also subdivide the figure into more panels than a-c.

Response: We have now subdivided the figure into panels (a–f).

Comment: Suppl Fig 11 shows the absence of common binding motifs in TCR CDR3b sequences for similar epitopes. See comments on Suppl Fig 10.

Response: We have now subdivided the figure into panels (a–e).

Comment: Suppl Fig 12 shows cluster alignment differences between single-class and concatenated embeddings for peptides and TCRs. The labels are very small and can be enlarged. Otherwise, no issues here.

Response: We have increased the font size of the labels.

Discussion

Comment: While APP's ability to assign cluster labels is highlighted, the authors could further discuss how the approach to projection is unique compared to modern methods like, for example, Phenograph.

Response: Please see the third and fourth paragraphs of the Introduction, where we have added some details to bring clarity to this. The main difference is that APP repeatedly restricts focus to a two-dimensional projection of the dataset, other modern clustering methods generally operate in high-dimensional space while using various other techniques to address the curse of dimensionality.
Notably, methods such as PhenoGraph, FlowSOM, and SPADE do not explicitly use any projections whatsoever. PhenoGraph represents the dataset as a mathematical graph built using the k-nearest neighbors of each point, then runs a Louvain community detection algorithm to cluster the graph. FlowSOM builds a two-dimensional grid of nodes (a self-organizing map) which captures topological information of the underlying dataset, then runs a consensus agglomerative hierarchical clustering method on this grid. SPADE downsamples the high-dimensional dataset in order to equalize local density, then performs agglomerative hierarchical clustering on the resulting downsampled "cloud".

Comment: Similar to my comment in the introduction, I think the authors should discuss APP in the context of modern clustering methods (which they used as benchmarks in the results, e.g., in scRNA-seq) and focus less on historical references.

Response: While we believe that we have already discussed projection pursuit clustering approaches, such as APP, in the context of modern clustering methods, we have now made this connection more explicit. Please refer to the statements we added to the Conclusions section of the manuscript.
“While the concept of projection pursuit has been around for decades, high-dimensional clustering (such as HDBSCAN, KMeans, and SPADE) has gained more popularity and attention, especially in the context of the computational challenges posed by modern data analysis.”
“In scenarios characterized by sparse or imbalanced data (such as the dataset presented in Figure 2), where there is a high degree of sparsity amid more abundant populations (which can extend to situations involving outliers and/or noise), projection pursuit proves beneficial in identifying pertinent dimensions and enhancing cluster separation. Traditional methods (such as HDBSCAN, KMeans, Phenograph and SPADE) may face challenges in handling sparsity (see Figure 2C) due to a dearth of informative features.”

Comment: For findings that did not have "ground truth" data (e.g., the multiplex imaging data), the authors should point out these limitations in the discussion.

Response: We have now added the following statements to the Results section:
“To encompass the range of real-world scenarios for clustering algorithm usage, both with and without ground truth or domain knowledge, we first demonstrate the method’s performance using a dataset with functionally validated ground truth. We then apply the method to a dataset where expert-defined manual gating serves as the ground truth. Next, we test the algorithm on single-cell mRNA expression data and imaging data, which lack predefined ground truth. However, cluster evaluation is enabled using domain knowledge from pathology and immunology, leveraging expression patterns and spatial distribution.
Finally, we apply APP clustering in a fully exploratory mode, where no ground truth or pre-existing domain knowledge is available to assess how TCR receptor embeddings cluster with respect to their cognate

antigens."
"ScRNA-seq or spatially resolved RNA-seq from the same sample or a serial section could serve as an orthogonal approach to assess cluster quality. However, we did not have further access to the sample used in this study for additional analysis."

Comment: For some of their interpretations, such as stating that they identified novel mechanisms, I would suggest that the authors clarify that follow-up studies with gain- or loss-of-function experiments need to be done to validate the in situ findings and strengthen these claims.

Response: We have incorporated the following statements into the COVID results section of the manuscript: "Although our new findings might represent a novel mechanism of immune modulation in severe COVID-19, further studies with more patients and different disease states are required to determine whether this is a general mechanism of SARS-CoV-2 infection or a phenotype unique to our small patient cohort."
And TCR results section: "However, follow-up studies will be necessary to further validate the findings and strengthen these claims."

Comment: This is a more general comment, but regarding the use of APP and LLMs, the authors could perhaps discuss the limitation that LLM representations may or may not accurately capture the underlying biological process. For example, how do they account for possible biases in LLM embeddings?

Response: We have now added the following statement to the relevant results section of the manuscript: "It is also important to note that while LLMs could potentially be biased by learning patterns from the limited training data provided, we have recently shown [Culka et al., 2024] that, regardless of the grouping approach (e.g., distance-based clustering, LLM, etc.), only a minority of TCRs form pure clusters predominantly composed of peptide-specific TCRs."

Comment: Minor (and more a personal interest): Could APP also be applied to non-biological high-dimensional datasets?

Response: Yes, there are no inherent limitations to applying APP to clustering other data types; however, as discussed in our manuscript, it is most suitable for tasks similar to cell phenotyping. Testing APP's performance on non-biological data modalities is beyond the scope of this manuscript.

em Editorial Manager®

Cookie Preference Center

We use cookies which are necessary to make our site work. We may also use additional cookies to analyze, improve and personalize our content and your digital experience. For more information, see our Cookie Policy.

You may choose not to allow some types of cookies. However, blocking some types may

impact your experience of our site and the services we are able to offer. See the different category headings below to find out more or change your settings.

Allow all

Manage Consent Preferences

Strictly Necessary Cookies

Always active

These cookies are necessary for the website to function and cannot be switched off in our systems. They are usually only set in response to actions made by you which amount to a request for services, such as setting your privacy preferences, logging in or filling in forms. You can set your browser to block or alert you about these cookies, but some parts of the site will not then work. These cookies do not store any personally identifiable information.

Functional Cookies

These cookies enable the website to provide enhanced functionality and personalisation. They may be set by us or by third party providers whose services we have added to our pages. If you do not allow these cookies then some or all of these services may not function properly.

Performance Cookies

These cookies allow us to count visits and traffic sources so we can measure and improve the performance of our site. They help us to know which pages are the most and least popular and see how visitors move around the site.

Targeting Cookies

These cookies may be set through our site by our advertising partners. They may be used by those companies to build a profile of your interests and show you relevant adverts on other sites. If you do not allow these cookies, you will experience less targeted advertising.

Cookie List

Clear

Apply Cancel

Consent Leg.Interest

Confirm my choices

Powered by onetrust
