# Supplementary material for: Lifting the curse from high-dimensional data: automated projection pursuit clustering for a variety of biological data modalities
Source: Gigascience. 2025 May 29;14:giaf052. doi: 10.1093/gigascience/giaf052 (PMC12121483; doi:10.1093/gigascience/giaf052)
Supplement: giaf052_Reviewer_1_Report_original_submission — Alexander Moskalensky -- 12/27/2024 [file giaf052_reviewer_1_report_original_submission.pdf]

Reviewer Report

Title: Lifting the curse from high-dimensional data: Automated projection pursuit clustering for the variety of biological data modalities

Version: Original Submission **Date:** 12/27/2024

Reviewer name: Alexander Moskalensky

Reviewer Comments to Author:

The manuscript by Claire Simpson, Evgeniy Tabatsky, and colleagues introduces a pipeline for the unsupervised clustering of high-dimensional data, leveraging sequential analysis of two-dimensional projections.
This problem is particularly significant in light of modern experimental techniques that generate data with tens or even hundreds of parameters from numerous cells.
The authors demonstrate applicability of the approach to a variety of datasets, including those with known ground-truth labeling. The algorithm is compared to several other methods for multi-dimensional unsupervised clustering, showing strong agreement and, in some cases, superior performance. Overall, I believe the paper merits publication, though several concerns should be addressed prior to its acceptance.
1. The authors reference the 'Curse of Dimensionality' in both the title and introduction but do not provide a detailed explanation of the issue. A more thorough discussion of this problem would help readers better understand how the proposed approach addresses and mitigates it.
The presented pipeline is based on the custom procedure for 2d clustering/decision boundary construction. This procedure is described to some extent, but the underlying logic is could be explained in more details. For now, it seems counter-intuitive that the same projections should be analyzed twice (I mean, different separating boundaries are constructed for x-y and y-x plots). Additionally, the "initial distribution density H" is not defined.
Overall, there seem to be a lot of existing algorithms for construction of optimal separating boundary or even automatic clustering of data in 2D space. It is unclear what advantages and disadvantages they have compared to the procedure developed by the authors (probably higher computational cost?). The authors tested their approach against three state-of-the art clustering algorithms (HDBSCAN, KMeans and Phenograph), but they were operating in the original high-dimensional space. The possibility of using these algorithms in 2D projections seems obvious and at least should be discussed.
2. The presented pipeline is based on the custom procedure for 2d clustering/decision boundary construction. While this procedure is described to some extent, the underlying logic could benefit from further clarification. As it stands, it seems counterintuitive that the same projections are analyzed twice (i.e., different separating boundaries are constructed for the x-y and y-x plots). Additionally, the 'initial distribution density H' is not defined, which requires further explanation.
Moreover, there are numerous existing algorithms for constructing optimal separating boundaries or performing automatic clustering in 2D space. It remains unclear what advantages or disadvantages the authors' procedure has compared to these methods (probably lower computational cost?). Although the

authors test their approach against three state-of-the-art clustering algorithms (HDBSCAN, KMeans, and Phenograph) operating in the original high-dimensional space, the possibility of applying these algorithms to 2D projections seems obvious and should at least be discussed.
3. In some cases, clusters may be well-separated in 3D space but appear mixed in 2D projections (such as xy, xz, yz). This can occur, especially when there are many clusters, but the situation might be improved through proper rotation of the coordinate system. Could the authors comment on the potential for this improvement? Additionally, an optimal decision boundary should ideally be independent of in-plane rotations, including the interchange of x- and y- axes. Could the authors suggest possible ways to algorithm modification to satisfy this requirement?
Minor points:
- Figure 1C appears overwhelming due to the large number of 2D projection plots, which seem disorganized. The authors might consider grouping the plots for better clarity. Additionally, the text on the plots is too small. Moreover, there seems to be no need to present six projections in 3D space for the identified clusters.
- Equation (1): The text mentions a square root, but the expression is raised to the power of 0.1. Should it be 0.5?
- The sentence "If the number of cells at the input to the recursion step is less than 2 * min_cluster_size" might be clearer if "the number of cells" is changed to "the number of points."

Methods

Are the methods appropriate to the aims of the study, are they well described, and are necessary controls included? Choose an item.

Conclusions

Are the conclusions adequately supported by the data shown? Choose an item.

Reporting Standards

Does the manuscript adhere to the journal's guidelines on minimum standards of reporting? Choose an item.

Choose an item.

Statistics

Are you able to assess all statistics in the manuscript, including the appropriateness of statistical tests used? Choose an item.

Quality of Written English

Please indicate the quality of language in the manuscript: Choose an item.

Declaration of Competing Interests

Please complete a declaration of competing interests, considering the following questions:

- Have you in the past five years received reimbursements, fees, funding, or salary from an organisation that may in any way gain or lose financially from the publication of this manuscript, either now or in the future?
- Do you hold any stocks or shares in an organisation that may in any way gain or lose financially from the publication of this manuscript, either now or in the future?
- Do you hold or are you currently applying for any patents relating to the content of the manuscript?
- Have you received reimbursements, fees, funding, or salary from an organization that holds or has applied for patents relating to the content of the manuscript?
- Do you have any other financial competing interests?
- Do you have any non-financial competing interests in relation to this paper?

If you can answer no to all of the above, write 'I declare that I have no competing interests' below. If your reply is yes to any, please give details below.

I declare that I have no competing interests

I agree to the open peer review policy of the journal. I understand that my name will be included on my report to the authors and, if the manuscript is accepted for publication, my named report including any attachments I upload will be posted on the website along with the authors' responses. I agree for my report to be made available under an Open Access Creative Commons CC-BY license (http://creativecommons.org/licenses/by/4.0/). I understand that any comments which I do not wish to be included in my named report can be included as confidential comments to the editors, which will not be published.

Choose an item.

To further support our reviewers, we have joined with Publons, where you can gain additional credit to further highlight your hard work (see: https://publons.com/journal/530/gigascience). On publication of this paper, your review will be automatically added to Publons, you can then choose whether or not to claim your Publons credit. I understand this statement.

Yes Choose an item.
