# Supplementary material for: Lifting the curse from high-dimensional data: automated projection pursuit clustering for a variety of biological data modalities
Source: Gigascience. 2025 May 29;14:giaf052. doi: 10.1093/gigascience/giaf052 (PMC12121483; doi:10.1093/gigascience/giaf052)
Supplement: giaf052_Reviewer_3_Report_original_submission — Yuri Vyatkin -- 1/6/2025 [file giaf052_reviewer_3_report_original_submission.pdf]

Reviewer Report

Title: Lifting the curse from high-dimensional data: Automated projection pursuit clustering for the variety of biological data modalities

Version: Original Submission **Date:** 1/6/2025

Reviewer name: Yuri Vyatkin

Reviewer Comments to Author:

The methods used in this study are well-suited to its aim of handling high-dimensional biological data. Techniques like APP, PCA, and supervised UMAP are described in detail and align with the study's goals. The inclusion of diverse datasets, such as flow cytometry and scRNA-seq, strengthens the study. However, some control experiments are not fully explained. For example, the optimization of APP parameters and the limited comparison with other clustering algorithms could be expanded. Additionally, while ground-truth validation is used for some datasets, others rely on expert evaluations, which can introduce bias.

The total sample size (six healthy controls and six COVID-19 patient) analyzed in the paper appears small for drawing robust conclusions, especially considering the biological variability in human samples. However, it's quite understandable that the number of samples is limited due to the availability of biological samples and the high cost associated of experimental techniques.

The study provides strong evidence for APP's ability to identify meaningful clusters and compares it with other clustering methods. However, some conclusions need stronger evidence. For instance, the claim that APP handles sparse data better than other methods is not consistently proven across all datasets.

The statistical methods used in the study, such as the Calinski-Harabasz index, are suitable for the types of data analyzed. PCA and Gaussian smoothing are reasonable choices for reducing data complexity and handling noise. However, some choices, like the beta parameter in APP, seem to be based on trial and error and need more justification. Additionally, the study reports accuracy metrics but could include other measures like F1-score to give a more complete picture of performance.

There are minor grammar issues and repetitive phrases that could be cleaned up. E.g.,

- P.2: While the latter challenge is computational rather than a fundamental scientificl imitation, it can be a serious practical hurdle.
- P.3: These insights include evaluating hypotheses regarding the existence of a binding motif between in CDR3b of TCRs that recognize the same peptide ...
- P.3: To gain a deeper understanding ... we initially applied ...
- etc.

Overall, moderate editing would improve the readability and flow of the manuscript.

In conclusion, the manuscript presents a well-justified and innovative approach to clustering high-dimensional biological data, and I recommend it for publication pending minor improvements to the text clarity.

Methods

Are the methods appropriate to the aims of the study, are they well described, and are necessary controls included? Choose an item.

Conclusions

Are the conclusions adequately supported by the data shown? Choose an item.

Reporting Standards

Does the manuscript adhere to the journal's guidelines on minimum standards of reporting? Choose an item.

Choose an item.

Statistics

Are you able to assess all statistics in the manuscript, including the appropriateness of statistical tests used? Choose an item.

Quality of Written English

Please indicate the quality of language in the manuscript: Choose an item.

Declaration of Competing Interests

Please complete a declaration of competing interests, considering the following questions:

- Have you in the past five years received reimbursements, fees, funding, or salary from an organisation that may in any way gain or lose financially from the publication of this manuscript, either now or in the future?
- Do you hold any stocks or shares in an organisation that may in any way gain or lose financially from the publication of this manuscript, either now or in the future?
- Do you hold or are you currently applying for any patents relating to the content of the manuscript?
- Have you received reimbursements, fees, funding, or salary from an organization that holds or has applied for patents relating to the content of the manuscript?
- Do you have any other financial competing interests?
- Do you have any non-financial competing interests in relation to this paper?

If you can answer no to all of the above, write 'I declare that I have no competing interests' below. If your reply is yes to any, please give details below.

I declare that I have no competing interests

I agree to the open peer review policy of the journal. I understand that my name will be included on my report to the authors and, if the manuscript is accepted for publication, my named report including any attachments I upload will be posted on the website along with the authors' responses. I agree for my report to be made available under an Open Access Creative Commons CC-BY license (http://creativecommons.org/licenses/by/4.0/). I understand that any comments which I do not wish to be included in my named report can be included as confidential comments to the editors, which will not be published.

Choose an item.

To further support our reviewers, we have joined with Publons, where you can gain additional credit to further highlight your hard work (see: https://publons.com/journal/530/gigascience). On publication of this paper, your review will be automatically added to Publons, you can then choose whether or not to claim your Publons credit. I understand this statement.

Yes Choose an item.
