# Supplementary material for: Lifting the curse from high-dimensional data: automated projection pursuit clustering for a variety of biological data modalities
Source: Gigascience. 2025 May 29;14:giaf052. doi: 10.1093/gigascience/giaf052 (PMC12121483; doi:10.1093/gigascience/giaf052)
Supplement: giaf052_Reviewer_4_Report_original_submission — Ruslan Rust -- 1/6/2025 [file giaf052_reviewer_4_report_original_submission.pdf]

Reviewer Report

Title: Lifting the curse from high-dimensional data: Automated projection pursuit clustering for the variety of biological data modalities

Version: Original Submission **Date:** 1/6/2025

Reviewer name: Ruslan Rust

Reviewer Comments to Author:

The authors develop a new clustering approach, automated projection pursuit (APP), for high-dimensional biological datasets. APP sequentially projects high-dimensional data into low-dimensional representations instead of operating in high-dimensional space (as is the case with other popular clustering approaches).
APP is validated on popular biological dataset applications, including flow and mass cytometry data, scRNA-seq, multiplex imaging data, and T-cell receptor repertoire data, and is partially benchmarked against other clustering methods.
Regarding the criteria from GigaScience Journal, the manuscript presents important findings and addresses an increasing problem in systems biology with large datasets. It also provides new biological datasets and fulfills the criteria of a research article by providing new scientific insights and conclusions.
In my opinion, in some parts, the manuscript is almost overwhelming with different applications and datasets that could be explained in more detail. Potentially, Figure 6 could even be split into a separate manuscript to streamline the different applications of APP. However, this can be addressed in the revised version.
I validated that all datasets and code are available. Some descriptions of the data could be improved, e.g., more detailed README files, etc. (see below). Perhaps the datasets could also be uploaded to permanent repositories in addition to the GitHub link to ensure permanent accessibility.
However, several aspects must be addressed (see below) to improve the quality of the manuscript, and I would recommend a major revision before the manuscript can be accepted for publication.
Introduction
The introduction is well-written and clearly explains the limitations of currently used high-dimensional clustering with the increasing size and dimensionality of big datasets.
The authors describe well their objective to combine the principles of projection pursuits and clustering into their Automated Projection Pursuit (APP) approach.While the limitations and objectives are clearly defined in the introduction, I believe the authors could expand on alternative, newer methods that people use for clustering.
The authors largely describe the historical initial work by Friedman et al., 1982; Huber, 1985, which is 40-50 years old. For instance, newer clustering algorithms might be relevant to include, along with mentioning their limitations. For example, the authors benchmark APP with algorithms such as HDBSCAN and K-means, but these are not introduced.
Additionally, mentioning graph-based clustering and dimensionality reduction techniques such as UMAP and t-SNE, and how they address challenges in high-dimensional clustering and have become standard

for scRNA-seq data analysis, might be helpful.
Since the authors compare APP to tools like Phenograph, this context could strengthen the introduction.Since much of the manuscript is about the validation of APP with real-world data, the authors may consider including a bit more information on why they chose these datasets and the different modalities (e.g., flow cytometry, scRNA-seq, multiplex imaging).
I appreciate the diversity of the data, but expanding a little on the rationale would be helpful.Additionally, the authors may clarify the novelty or major advantage of the APP clustering tool. For instance, the authors mention that APP is an alternative clustering approach, but they could perhaps elaborate on its major strengths or novelty in the last paragraph.
The last three paragraphs of the introduction discuss the findings of this manuscript. I would suggest shortening this part into a single last paragraph in the introduction.
Minor comments:
The authors could use a reference instead of a link in the introduction when referring to supervised UMAP.
It may not be ideal to use "APP" for the name of the software in the context of models of Alzheimer's disease, as most AD researchers associate APP with Amyloid Precursor Protein.

Methods
Overall, the methods are well described and also include the analysis procedures. All steps in the analysis appear on GitHub. I have just written a few comments that could improve clarity and details.
I checked that IRB protocols were approved by the review board.
Since the authors generated some of their own data and used publicly available datasets, it might be helpful to add a table with an overview of all datasets they used. It took me some time to understand which datasets were generated in this study and which ones were taken from a public database.
Perhaps the authors could explain why they decided to include their own data if the main purpose is to benchmark APP against other clustering software.
For the flow cytometry data, the authors could add the manufacturer, species, and dilution of the antibody.
More information about the antibodies and their dilution would also be beneficial for the multiplex imaging data.
The CyTOF methods are not properly described. Although the authors refer to previous studies, they should consider providing a bit more detail.
Why did the authors use Seurat v3? The current version is v5. Perhaps the authors could double-check whether their code is still executable with v5.
The description of the scRNA-seq analysis is quite brief; however, the coding data is available on GitHub. In case the authors have not done so, it would be helpful to deposit the data in a permanent database such as Zenodo (since the GitHub data could be altered or deleted).
All raw data and code appear to be available on GitHub. The authors could perhaps elaborate on the README files in the main folder to provide more detailed instructions and descriptions of the project.
Figure 1: The authors provide a data clustering workflow of APP. It helps to understand the underlying methodology of the APP algorithm and describes how it identifies projections, applies boundaries, and splits data to identify clusters. I have no concerns here.

Results
Fig 2:
In Figure 2, the authors compare APP performance to widely used clustering methods in flow cytometry of immune cells from WT and RAG KO mice.
The experimental design is very elegant, mixing the population of cells in different proportions to validate individual cell types, but I think this needs to be better explained in the results. Especially since most scientists reading this manuscript might have a bioinformatics background and may not be familiar with the mouse model and the experimental setup.
Also, the clustering algorithms that were used as a comparison have several parameters that can be adjusted. How were those chosen? Did any of them lead to better/worse clustering?
Perhaps in a comparison like this, it would be helpful to briefly mention the computational performance of the methods. For instance, how long did each algorithm take to run under which computer configuration?
It is not clear to me how they generated the UMAP in Fig 2C. Is this based on the APP clustering? And then were the labels transferred from the other clustering algorithms? A bit more explanation is needed.
Did the authors perform any statistical analysis on the quantified data? The plots look convincing but should be tested using appropriate statistical tests in Fig 2D and E.
The authors also include discussion within the results; if this aligns with the journal's guidelines, that is okay for me.
Fig 3:
In Fig 3, the authors describe APP's performance in clustering PBMCs from healthy donors and COVID-19 patients and benchmark the performance to Phenograph.
The authors should add statistical analysis to Fig 3 A-D and F.
The authors convincingly show that the label-transfer pipeline is very accurate when training on one healthy control sample, and that there were more discrepancies with COVID samples. I think the authors could expand a little more on the diverse responses of COVID patients and cellular shifts, which could also include cellular transition states. Also, intragroup variability is likely higher in COVID patients.
The authors suggest that "these findings highlight new mechanisms of SARS-CoV-2-induced immuno-modulation that underlie the COVID-19 immunopathology in hospitalized patients," which might be a slight overstatement. They detect a subpopulation of monocytes, which is interesting. However, there is no data indicating a new mechanism. Toning this statement down would solve the issue.
Fig 4:
In Figure 4, the authors evaluate APP's performance on scRNA-seq data from PBMCs and 5xFAD mice brain datasets.
The scRNA-seq clustering in Seurat depends on the parameters chosen. Did the authors use default parameters?
The authors claim that naÃ¯ve and memory CD4 T cells do not show any distinction in clustering in any pairwise dimensions explored by APP but refer to Supplementary Data without specifying where in the Supplementary Data this information can be found. Please add a specific reference to where this information can be found.
Did the authors examine CD45RA/PTPRC and CD62L/SELL levels in their PBMC RNA-seq data? These

markers are usually used to distinguish naÃ¯ve and memory CD4 T cells.
For the scRNA-seq dataset, it would be helpful to compare the performance in terms of the time required for clustering. scRNA-seq datasets can have millions of cells, and Seurat may face challenges with conventional analysis. How did APP perform compared to Louvain clustering in terms of speed? Could the authors also test it on a larger dataset?
Perhaps the authors could check whether the clustering algorithm in Seurat was updated between v3 and v5 (the most current version). If not, they could consider adding this information to the methods.
Fig 5:
In Figure 5, the authors test the application of APP on multiplexed imaging data and explore the cellular composition within the tissue of a human squamous lung carcinoma sample.
What do coordinate X and Y represent? Please add the labels (in Fig 5C-D).
There is almost no figure description for Fig 5. Please expand, e.g., add a scale bar and a description of the heatmap (what does 0-1 in the scale mean? Normalized expression?).
As the authors mentioned, they do not have the information on the ground truth in this dataset, and they also do not compare APP to alternative clustering algorithms for this dataset. What was the rationale for that?
The authors say: "Pathology and immunology experts independently assessed and confirmed the adequacy of APP clustering in characterizing and distinguishing meaningful cell populations within the slide tissue, particularly in the context of the human squamous lung carcinoma sample." I am not sure how helpful this assessment is, as it is hard to validate and anonymous, and I am also not sure how accurately a pathologist can assess the clustering of a rare cell type that might be misclassified. Would it be helpful if the authors examined the composition of similar cancerous tissues in scRNA-seq/spatial RNA-seq databases and confirmed the expression of the identified marker genes? If this is not possible, the authors could add this as a limitation.
Fig 6:
In Fig 6, the authors describe APP clustering in the context of uncovering patterns and relationships of TCR-antigen sequences. They used LLMs for TCR to capture patterns and used APP to interpret the LLM embeddings to identify sequence similarities and shared patterns.
Although I find this an interesting approach to partially unravel the decision-making of LLMs, in my opinion, this part of the results is slightly disconnected from the previous findings in Figs 1-5. The data in Fig 6 (and the associated Supplementary Figure) could almost represent a new manuscript. I am not saying it needs to be separated, but it may help to streamline the findings.
If the authors want to keep Fig 6, they should benchmark the use of APP with other clustering methods alongside the LLMs to determine whether the performance of APP is comparable to other clustering algorithms.
The authors generate two embeddings for Fig 6, the second embedding being a subset of approximately 4,000 unique TCRs with sequence information for both TCR CDR3Î± and CDR3Î². Could the authors add the rationale for doing this?
Also, similar to previous findings, it is important to know what parameters were used for APP and how robust the results are to changes in these parameters.
Suppl Figures:
Suppl Fig 1 shows an example of decision boundary search for 2D projections in APP. Perhaps the figure

legend could be expanded, but otherwise, no issues here.
Suppl Fig 2 shows the workflow for automated label transfer using UMAP to align cluster labels and quantify misclassification. No issues here.
Suppl Fig 3 shows an example of discrepancies between data topology and clustering decisions in manual gating. Since this is a UMAP, the numbers on the x and y axes are not particularly meaningful and can be removed. Otherwise, no issues here.
Suppl Fig 4 shows a representation of the label transfer pipeline comparing clustering algorithms. I am not sure why the authors used the word "cartoon." Perhaps they can clarifyâ€”this is real data from their clustering, right?
Suppl Fig 5 shows their gating strategy refinement for flow cytometry. No issues here.
Suppl Fig 6 shows a UMAP with misclassification by APP and Phenograph compared to manual gating annotations. The labeling is a bit difficult to read and not always clear to which cluster it refers. Also, the numbers on the x and y axes can be removed.
Suppl Fig 7 shows that cell populations are more prone to misclassification in healthy donors and COVID-19 samples with APP and Phenograph. No issues here.
Suppl Fig 8 shows the impact of data topology and ground truth labels on label transfer performance. No issues here.
Suppl Fig 9 shows a violin plot of marker gene expression and the heterogeneity in CD4 and CD8 clusters in the PBMC dataset.
Suppl Fig 10 shows sequence similarity and amino acid group properties in peptide and TCR embeddings. The figure feels a bit busy and overwhelming with data (true for subsequent supplementary figures as well). Perhaps the authors could decide whether all of the data is required to show the sequence similarity, and they should also subdivide the figure into more panels than a-c.
Suppl Fig 11 shows the absence of common binding motifs in TCR CDR3b sequences for similar epitopes. See comments on Suppl Fig 10.
Suppl Fig 12 shows cluster alignment differences between single-class and concatenated embeddings for peptides and TCRs. The labels are very small and can be enlarged. Otherwise, no issues here.
Suppl Fig 13 shows the enrichment of charged amino acids stabilizing TCR-pMHC interactions through hydrogen and ionic bonds. No issues here. a

Discussion

The authors summarize their findings and contextualize the main advantage of APP in the use of high-dimensional data and assigning cluster labels.
While APP's ability to assign cluster labels is highlighted, the authors could further discuss how the approach to projection is unique compared to modern methods like, for example, Phenograph.
Similar to my comment in the introduction, I think the authors should discuss APP in the context of modern clustering methods (which they used as benchmarks in the results, e.g., in scRNA-seq) and focus less on historical references.
For findings that did not have "ground truth" data (e.g., the multiplex imaging data), the authors should point out these limitations in the discussion.
For some of their interpretations, such as stating that they identified novel mechanisms, I would suggest that the authors clarify that follow-up studies with gain- or loss-of-function experiments need to be done to validate the in situ findings and strengthen these claims.

This is a more general comment, but regarding the use of APP and LLMs, the authors could perhaps discuss the limitation that LLM representations may or may not accurately capture the underlying biological process. For example, how do they account for possible biases in LLM embeddings?
Minor (and more a personal interest): Could APP also be applied to non-biological high-dimensional datasets?
Disclaimer:
My review was corrected for grammar and typos using GPT4 on December 22, 2024 with the phrase "Correct my text for grammar and typos only, do NOT correct or alter any of content".
Note:
I previously reviewed this paper previously in Research Hub and you can read these comments via the Research Hub review page here: https://www.researchhub.com/paper/6670845/lifting-the-curse-from-high-dimensional-data-automated-projection-pursuit-clustering-for-the-variety-of-biological-data-modalities/reviews#threadId=55525.
The original preprint linked to the Research Hub review is here: https://doi.org/10.1101/2024.04.18.589981.

Methods

Are the methods appropriate to the aims of the study, are they well described, and are necessary controls included? Choose an item.

Conclusions

Are the conclusions adequately supported by the data shown? Choose an item.

Reporting Standards

Does the manuscript adhere to the journal's guidelines on [minimum standards of reporting?](#) Choose an item.

Choose an item.

Statistics

Are you able to assess all statistics in the manuscript, including the appropriateness of statistical tests used? Choose an item.

Quality of Written English

Please indicate the quality of language in the manuscript: Choose an item.

Declaration of Competing Interests

Please complete a declaration of competing interests, considering the following questions:

- Have you in the past five years received reimbursements, fees, funding, or salary from an organisation that may in any way gain or lose financially from the publication of this manuscript, either now or in the future?
- Do you hold any stocks or shares in an organisation that may in any way gain or lose financially from the publication of this manuscript, either now or in the future?
- Do you hold or are you currently applying for any patents relating to the content of the manuscript?
- Have you received reimbursements, fees, funding, or salary from an organization that holds or has applied for patents relating to the content of the manuscript?
- Do you have any other financial competing interests?
- Do you have any non-financial competing interests in relation to this paper?

If you can answer no to all of the above, write 'I declare that I have no competing interests' below. If your reply is yes to any, please give details below.

I declare that I have no competing interests.

I agree to the open peer review policy of the journal. I understand that my name will be included on my report to the authors and, if the manuscript is accepted for publication, my named report including any attachments I upload will be posted on the website along with the authors' responses. I agree for my report to be made available under an Open Access Creative Commons CC-BY license (http://creativecommons.org/licenses/by/4.0/). I understand that any comments which I do not wish to be included in my named report can be included as confidential comments to the editors, which will not be published.

Choose an item.

To further support our reviewers, we have joined with Publons, where you can gain additional credit to further highlight your hard work (see: https://publons.com/journal/530/gigascience). On publication of this paper, your review will be automatically added to Publons, you can then choose whether or not to claim your Publons credit. I understand this statement.

Yes Choose an item.
